# Helical aromatic oligoamide foldamers as selective G-quadruplex ligands

**DOI:** 10.1093/nar/gkaf1365

**Published:** 2025-12-31

**Authors:** Alexander König, Vincent Laffilé, Stéphane Thore, Cameron D Mackereth, Liliya A Yatsunyk, Yann Ferrand, Eric Largy, Valérie Gabelica

**Affiliations:** University of Bordeaux, CNRS, INSERM, ARNA, UMR 5320, U1212, F-33600 Bordeaux, France; University of Bordeaux, CNRS, IPB, CBMN, UMR 5248, IECB, F-33600 Bordeaux, France; University of Bordeaux, CNRS, INSERM, ARNA, UMR 5320, U1212, F-33600 Bordeaux, France; University of Bordeaux, CNRS, INSERM, ARNA, UMR 5320, U1212, F-33600 Bordeaux, France; Department of Chemistry and Biochemistry, Swarthmore College, Swarthmore, PA 19081, United States; University of Bordeaux, CNRS, IPB, CBMN, UMR 5248, IECB, F-33600 Bordeaux, France; University of Bordeaux, CNRS, INSERM, ARNA, UMR 5320, U1212, F-33600 Bordeaux, France; University of Bordeaux, CNRS, INSERM, ARNA, UMR 5320, U1212, F-33600 Bordeaux, France; School of Pharmaceutical Sciences, University of Geneva, 1206 Geneva, Switzerland

## Abstract

We investigated the G-quadruplex (G4) binding selectivity of short aromatic oligoamide helical foldamers comprising quinoline (Q) and pyridine (P) units. We found that the foldamers bind with 1:1 and 2:1 stoichiometries and prefer parallel G4 structures, especially when the external G-quartets are sterically accessible. A crystal structure of the tetramer QQPQ with the parallel G4 formed by dTGGGTTGGGTTGGGTTGGGT shows two quinoline subunits interacting with an external G-quartet through π-stacking, and solution nuclear magnetic resonance (NMR) confirms that the foldamer targets the 3′ and 5′ ends of this G4. Foldamers can also selectively target sequence variants of the telomeric sequences containing adenine-to-thymine mutation in the loops. The conformational selectivity of foldamers originates from the bulkiness of oligomers with four or more subunits, which imposes steric restrictions on G4 binding. The flexibility provided by the pyridine subunits was also key to improve affinity. Mixed quinoline–pyridine foldamers are thus a promising class of selective G4 ligands, and their unique modular scaffold offers new avenues to further improve their affinity and selectivity.

## Graphical Abstract

**Graphical Abstract:**
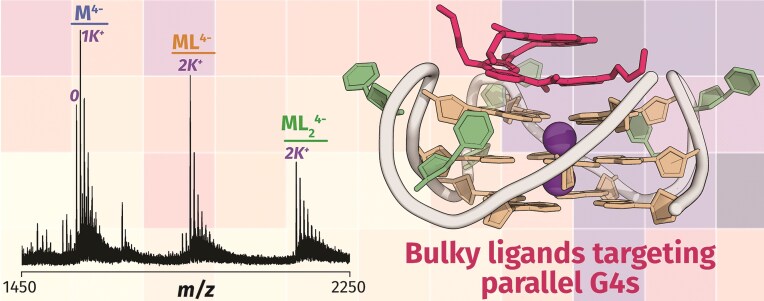

## Introduction

G-quadruplexes (G4s) are noncanonical DNA structures wherein four guanines associate into a quartet through Hoogsteen-type H-bonding [1]. G4 folding topologies are diverse and some sequences can form several energetically close G4 topologies [2–7]. The structure(s) adopted by a given DNA sequence can depend on the concentration of DNA strand, the type and concentration of cation [5, 8–10], or on the ionic strength and pH of the buffer [11, 12]. Over the past decades, the solution-phase structures of several G4s have been resolved [13–15].

G4-forming sequences are abundant in promoters and at the telomeres, opening avenues to develop G4-targeting ligands as antiproliferative agents or to regulate gene expression [16–22]. G4 ligands are generally flat, condensed heteroaromatic systems that allow efficient π-stacking on G-quartets while inhibiting double-stranded DNA (dsDNA) intercalation [23]. Cationic side chains are often added to improve water solubility and favor electrostatic interactions with the nucleic acid backbone [24]. However, while G4 selectivity over duplexes is often achieved, most ligands lack selectivity among different G4s [25, 26]. Ligands discriminating certain topologies [27–29], or topology subclasses [30–32], have already been reported, but rational design efforts have not yet yielded small molecules with significant selectivity for a specific G4 structure [33]. Molecular scaffolds that deviate from the condensed aromatic structure paradigm may be required to produce ligands with improved topology selectivity [30].

**Figure 1.**
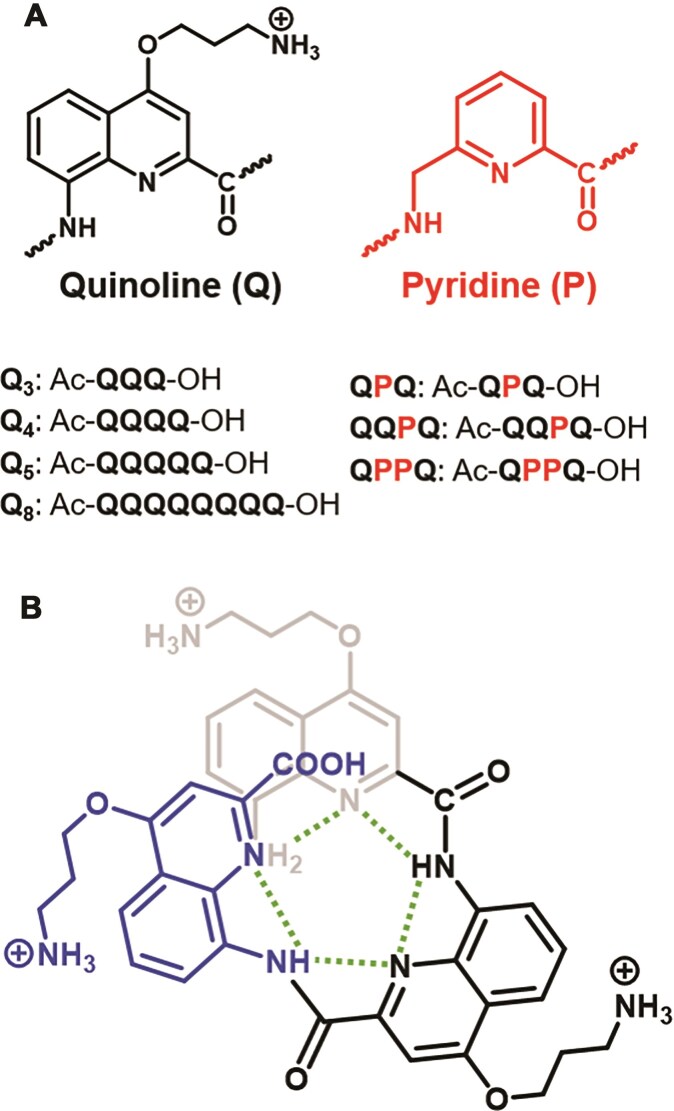
(**A**) Oligoquinoline foldamers used in this study, containing quinoline (Q) and pyridine (P) subunits [panel (B) shows Q_3_]. (**B**) Intramolecular H-bonds (green) sterically prohibit a planar structure, forcing the molecule into a (racemic) helical assembly.

Inspired by biopolymers, foldamers are synthetic oligomers self-organized into a defined folded structure [34]. Here we studied quinoline oligoamide foldamers that fold into a helix driven by intramolecular forces including electrostatic repulsions, intramolecular hydrogen bonds, conjugation and extensive aromatic stacking [35–37]. In the absence of any chirality inducers, helical foldamers exist as a racemic mixture of left- and right-handed helices [38]. Foldamers possess two characteristic traits of G4 ligands: (i) an aromatic heterocyclic core structure, which is derived from quinoline and (ii) extended, flexible side chains. Importantly, ligands containing pyridine and quinoline moieties linked by amide bonds, such as 360A and its close analogue Pyridostatin, have already shown excellent affinity and selectivity for G4s [39–43]. But unlike the ligands mentioned above, the foldamers studied here are not flat. Our foldamers have a positively charged side chain (derived from ornithine) to enhance water solubility (Fig. 1). We also introduced pyridine monomers devoid of any side chain to increase the flexibility of the helices.

The interaction between oligoquinoline foldamers and G4s has been studied before. They stabilize G4s, but have no effect on DNA duplexes, according to Förster resonance energy transfer (FRET)-melting studies [35, 36, 44]. Selectivity among different G4 sequences was observed, but not satisfyingly explored further in terms of structure, as the main aim was to demonstrate foldamers targeting G4s over other secondary structures. In a study involving FRET-melting and smFRET experiments, Müller and coworkers proposed the groove binding of their oligoquinoline foldamers [36]. In a subsequent study, Mandal *et al.* co-crystallized (G_4_T_4_G_4_)_2_ with a tetramer from this series, confirming the absence of binding to the tetrads [37]. However, only electrostatic interactions between the foldamer side chains and the G4 were evidenced, which does not appear sufficient to explain their affinity and specificity in the solution phase. The takeaway lessons from these previous studies are: (i) Negatively charged oligoquinoline foldamers do not interact with G4s, likely due to charge repulsion [36, 45]. Hence, aromatic foldamers designed as G4 ligands should carry positive charges, which are implemented through the side chain. (ii) The effect of oligoquinoline foldamer helicity on G4 binding is uncertain. It was probed by inducing the handedness quantitatively into a left (*M*) or right (*P*) configuration by covalently attaching either *(R)* or *(S)*-camphanic acid, respectively. However, introducing the camphanyl residue disrupts the foldamer-G4 interaction, indicating that the G4-foldamer interaction site is located at the *N*-terminus of the foldamer [45]. Helicenes derived from diazaoxatriangulenium (DAOTA) were partially separated using diastereoselective precipitation. The difference in *K*_D_ value for the *(M)* and *(P)* helices was less than factor 1.5 [46]. (iii) Oligoquinoline foldamers and antiparallel G4s can co-crystallize without any specific ligand-target interaction [37].

On the course of native mass spectrometry (MS) experiments for another project, we serendipitously rediscovered the high binding affinity of foldamer Q_4_ with the parallel-forming sequence 1XAV (from the *c-myc* promoter region), then large affinity differences with variants of the telomeric sequence. Here, we report the systematic investigation of a larger panel of sequences with several foldamers, followed by detailed biophysical and structural investigations to understand the determinants of sequence or topology preferences. The sequences were largely selected from our database listing resolved G4 structures in both nuclear magnetic resonance (NMR) and electrospray ionization mass spectrometry (ESI-MS) compatible conditions, allowing for straightforward structural interpretation [47].

## Experimental methods

### Foldamer synthesis

Seven foldamer sequences (Supplementary Figs S1 and S2) were prepared according to the synthetic schemes in the supporting information (Supplementary Figs S3–S6). NMR spectra, high-performance liquid chromatography (HPLC) traces and accurate masses for each foldamer are provided in Supplementary Figs S7–S20.

The aromatic oligoamides were synthesized using a microwave-assisted solid-phase synthesis approach. The matrix is a low loading Cl-MPA ProTide^®^ resin. The first monomer unit was attached to the resin using CsI/DIEA. Subsequently, each monomer was coupled to the oligomer chain iteratively through periodic cycles of deprotection. Coupling to the next monomer unit was performed using Appel conditions (PPh_3_/trichloroacetonitrile) with collidine as base. Finally, trifluoroacetic acid (TFA) was used to simultaneously cleave the oligomer from the resin and deprotect the Boc groups of the side chains to yield the corresponding ammonium salts. After filtration of the remaining solids, the TFA solution was evaporated then the residue was suspended in Et_2_O and centrifuged. The solid was finally dissolved in water and freeze-dried.

All sequences were purified using semi-preparative HPLC on a reverse phase C18 column (mobile phase: H_2_O/ACN/TFA). The purified foldamers were freeze-dried twice more to remove TFA traces. The final product was obtained as a yellow solid with a cotton-like texture, consisting of the foldamer with one trifluoroacetate counterion per ammonium group. Foldamer stock solutions were prepared by weighing in the purified product on a microbalance (Sartorius ME5) and dissolving them in UPLC/MS grade pure water.

### Oligonucleotides and sample preparation

All oligonucleotide sequences were purchased from Eurogentec (Belgium) and dissolved in UPLC/MS grade pure water (Biosolve Chimie, France) to 1 mM DNA. Stock solutions were annealed for 3 min at 85°C, then any residual Na^+^ ions were exchanged with 500 mM NH_4_OAc, which we then flushed out with water using cellulose-matrix 3K centrifugal filter units (MerckMillipore, Ireland). We validated the desalting method by analyzing a DNA stock solution before and after desalting (Supplementary Fig. S21). The concentration of each DNA stock solution was determined with a ultraviolet (UV) spectrophotometer (Uvikon XL Secomam), utilizing extinction coefficients at 260 nm that were calculated from the nucleotide sequence via nearest-neighbor method [48]. An overview of the sequences studied is provided in Table 1. To form G4, DNA solutions were doped with either potassium chloride from Sigma Aldrich and trimethylammonium acetate solution (TMAA) from ChemCruz or ammonium acetate solution (NH_4_OAc) from Sigma Aldrich (Saint-Quentin-Fallavier, France).

**Table 1.** DNA sequences used for the foldamer screening experiments

Name	PDB	Sequence	Morphology^a^	θ (20°C)^b^
1XAV	1XAV	dT**G**A**GGG**T**GGG**TA**GGG**T**GGG**TAA	Parallel G4	100%
222T		dT**GGG**TT**GGG**TT**GGG**TT**GGG**T	Polymorphic G4	98%
222T_mA		dT**GGG**TT**GGG**AA**GGG**TT**GGG**T	Polymorphic G4	92%
222T_mC		dT**GGG**TT**GGG**CC**GGG**TT**GGG**T	Polymorphic G4	96%
T30177TT	2M4P	dTT**G**T**GG**T**GGG**T**GGG**T**GGG**T	Parallel G4	100%
26CEB	2LPW	dAA**GGG**T**GGG**T**G**TAA**G**T**G**T**GGG**T**GGG**T	Parallel G4	100%
26CEB-mT		dAA**GGG**T**GGG**TTTTTTT**G**T**GGG**T**GGG**T	Parallel G4	100%
2KYP	2KYP	dC**GGG**C**GGG**C**G**CTA**GGG**A**GGG**T	Parallel G4	93%
2O3M	2O3M	dA**GGG**A**GGG**C**G**CT**GGG**A**GG**A**GGG**	Parallel G4	84%
TG4T	2O4F	(dT**GGGG**T)_4_	Parallel G4	N/A
21G		d**GGG**TTA**GGG**TTA**GGG**TTA**GGG**	Polymorphic G4	100%
5YEY	5YEY	d**GGG**TTA**GGG**TTA**GGG**TTT**GGG**	Antiparallel G4	100%
22GT	2KF8	d**GGG**TTA**GGG**TTA**GGG**TTA**GGG**T	Antiparallel G4	98%
22GT_18T		d**GGG**TTA**GGG**TTA**GGG**TTT**GGG**T	Antiparallel G4	100%
22CTA	2KM3	dA**GGG**CTA**GGG**CTA**GGG**CTA**GGG**	Antiparallel G4	88%
TBA	148D	d**GG**TT**GG**T**G**T**GG**TT**GG**	Antiparallel G4	90%
G4T4G4	4R47	(d**GGGG**TTTT**GGGG**)_2_	Antiparallel G4	N/A
26TTA	2JPZ	dTTA**GGG**TTA**GGG**TTA**GGG**TTA**GGG**TT	Hybrid G4	66%
Bcl2	2F8U	d**GGG**C**G**C**GGG**A**GG**AATT**GGG**C**GGG**	Hybrid G4	97%
24TTG	2GKU	dTT**GGG**TTA**GGG**TTA**GGG**TTA**GGG**A	Hybrid G4	99%
24TTG_20T		dTT**GGG**TTA**GGG**TTA**GGG**TTT**GGG**A	Hybrid G4	98%
23TAG	2JSM	dTA**GGG**TTA**GGG**TTA**GGG**TTA**GGG**	Hybrid G4	94%
2KPR	2KPR	d**GGG**T**GGGG**AA**GGGG**T**GGG**T	Hybrid G4	100%
21CCC		dCCCTAACCCTAACCCTAACCC	i-motif (pH 5.5) or single strand (pH 7)	100%
ds26		(dCAATCGGATCGAATTCGATCCGATTG)_2_	Duplex/Hairpin	100%
DK-33		(dCGTAAATTTACG)_2_	Duplex	100%
DK-66	1FQ2	(dCGCGAATTCGCG)_2_	Duplex	100%
DK-100		(dCGCGGGCCCGCG)_2_	Duplex	100%
ss24		dTGCCATGCTACTGAGATGACGCTA	Single strand	
24nonG4		dTGGGATGCGACAGAGAGGACGGGA	Single strand	
T24		dTTTTTTTTTTTTTTTTTTTTTTTT	Single strand	
A24		dAAAAAAAAAAAAAAAAAAAAAAAA	Single strand	
T6		dTTTTTT	Single strand	

a Most abundant secondary structure in MS sample conditions

b Fraction of folded DNA at 20°C in 1 mM KCl, based on UV melting data

### Circular Dichroism spectroscopy

CD measurements were performed on a JASCO J-815 spectrophotometer equipped with a Lauda RE 305 temperature control system with the following parameters: 220–500 nm scan range, 50 nm/min scanning speed, 0.2 nm data pitch, 2 nm bandwidth, 2 s data integration time, 22°C temperature in the sample holder, three accumulations. The samples, containing 10 µM of DNA, were placed in Suprasil quartz cuvettes (Hellma) with 2 or 10 mm pathlength.

Raw data was blank subtracted then converted to molar ellipticity $\Delta \varepsilon $ (M^−1^cm^−1^) with equation (1), where $\theta $ is the ellipticity (mdeg), $c$ the molar concentration of oligonucleotide (mol/l), and $l$ the path length (cm).


(1) \begin{eqnarray*}
\Delta \varepsilon = {\mathrm{\ }}\frac{{\theta {\mathrm{\ }}}}{{32980 \times c \times l{\mathrm{\ }}}}\
\end{eqnarray*}

We provide CD spectra in MS conditions for all intramolecular G4 sequences listed in Table 1. They have either been published previously [47], or are featured in the supporting information (Supplementary Figs S22–S29).

### Thermal denaturation

Thermal denaturation was monitored by UV absorption spectroscopy on a SAFAS UVmc2 double-beam spectrophotometer equipped with a high-performance Peltier temperature control unit. The melting ramps were 20→90°C (10°C/min), 90→4°C (0.2°C/min), 4→90°C (0.2°C/min), 90 s data reading interval, 0.5 s averaging time. The samples containing 10 µM of DNA were placed in 10 mm path length Suprasil quartz cuvettes (Hellma). The UV absorption was taken at 260, 295, and 335 nm. The 260 nm wavelength corresponds to the DNA duplex, the 295 nm one to the G4 and the absorption at 335 nm serves as an internal reference to correct for instrumental artifacts. In addition, the buffer alone (100 mM TMAA and 0.5/1 mM KCl) was measured for blank correction.

Raw data, at 260 nm for dsDNA and 295 nm for G4s, was blank subtracted, and corrected with the 335 nm data, then converted to folded fraction $\theta $ using equation (2), where $L0( T )$ and $L1( T )$ are the baseline values of the unfolded and folded species, respectively [49, 50].


(2) \begin{eqnarray*}
\theta \left( T \right) = {\mathrm{\ }}\frac{{L0\left( T \right) - A\left( T \right)}}{{L0\left( T \right) - L1\left( T \right)}}
\end{eqnarray*}

The melting temperature T_M_ is the temperature for which θ = 0.5. We provide ${\theta }\,({20^\circ {\mathrm{C}}})$ for all intramolecular G4, i-motifs and duplex forming sequences, in MS conditions in Table 1. Melting curves have either been published previously [47], or are featured in the supporting information (Supplementary Figs S22–S27).

### Native MS: instrumental setup

Native MS experiments were conducted on an Agilent 6560 IMS-QTOF. The instrumental settings were optimized to preserve noncovalent DNA structures, albeit making a compromise between signal intensity and softness of the ion transfer (Supplementary Table S1) [51]. Samples were infused at 3 µL/min for 11 min, working foldamer-by-foldamer to avoid cross-contaminations. The instrument is equipped with a drift tube for ion mobility spectrometry (IMS). The ions pass through a tube, filled with helium, and we measure the time between ions being released into the drift tube until arriving at the detector. This time is the arrival time t_A_ is related to the reduced mobility *K*_0_, whose correlation to the collisional cross section (CCS) is described in the Mason–Schamp equation (equation 3).


(3) \begin{eqnarray*}
CCS = \frac{3}{{16}}\frac{{ze}}{{{N}_0{K}_0}}\sqrt {\frac{{2\pi }}{{\mu {k}_BT}}}
\end{eqnarray*}

Where *e* is the charge of the electron, *k_B_* is the Boltzmann constant, and *N_0_* is the gas number density (Loschmidt number) at *T*_0_ = 275.15 K and *p*_0_ = 1 atm. The temperature *T* and the reduced mass µ [µ = (*m*_ion_*m*_He_)/(*m*_ion_+ *m*_He_)] remain practically constant within the experimental conditions (details in Supplementary Table S1). The reduced mobility *K_0_* and charge state *z* differ for every ion species. We obtained the reduced mobility by measuring the arrival time at several drift voltages (Supplementary Figs S30 and S31) and performing a linear fit according to equation (4).


(4) \begin{eqnarray*}
{t}_A = {t}_0 + \frac{{{L}^2p{T}_0}}{{{K}_0{p}_0T}}\frac{1}{{\Delta V}}
\end{eqnarray*}

where *t_0_* is the dead time, *L* is the length of the drift tube (78.1 cm), *p* and *T* are the pressure and temperature inside the drift tube (which are recorded for each experiment) and *K_0_* is the reduced mobility of the ion.

We validated the instrumental settings before each series of measurements using [(dTG_4_T)_4•_(NH_4_)_3_]^z−^ as an external calibrant. We accepted the instrumental settings when the experimental CCS values were within 2% of error to our previously published CCS values (787.5 Å² [5-] ion, 735.7 Å² [4-] ion) [52]. The supporting information contains a step-by-step tutorial for how to obtain CCS values and reconstruct CCS distributions (Supplementary Figs S30 and S31 and accompanying text).

### Ligand screening with native MS

The panel (Table 1) includes 23 G4-forming sequences featuring different strand stoichiometries (1, 2, 4), numbers of G-quartets (2–4), topologies (parallel, antiparallel, hybrid), and origins (e.g. oncogene promoter, telomeres, aptamers). The panel also includes control sequences of alternative secondary DNA structures (duplex, i-motif, single strand). We screened the DNA panel against four different foldamer sequences: QPQ, QQPQ, QPPQ, and Q_4_. Screening samples contained 10 µM DNA, 20 µM foldamer, 4 µM dT_6_, 0.5 mM KCl and 100 mM TMAA (pH 6.8) in water. 150 mM ammonium acetate (pH 6.8) was used for the sequences T_6_, T_24_, ss24, DK-66, and 21CCC. The multi-stranded G4s TG_4_T and G_4_T_4_G_4_ were screened in both TMAA/KCl and ammonium acetate.

First, each stoichiometry of complex is directly inferred from the mass of the complex. Then, the concentration of DNA species of any stoichiometry (M*) was determined using Equation 5, where [*M*]_0_ is the total concentration of DNA in the sample and *I* is the intensity of the different species, obtained by integrating all signal within a specified *m/z* window (monomeric DNA: $M$, ligand: $L$) [53]. The mass spectra in the supporting information show the *m/z* selection windows. The concentration of unbound foldamer is determined by difference (Eq. 6), where [*L*]_0_ is the total concentration of foldamer in the sample. The dissociation constants *K*_D1_ and *K*_D2_ values for the 1:1 and 2:1 (*L:M*) complexes were then determined from Equations 7 and 8. The fraction of bound DNA (Eq. 9) is a more concise parameter to present ligand screening results.


(5) \begin{eqnarray*}
\left[ {{M}^*} \right] = \ {\left[ M \right]}_0\ \frac{{I\left( {{M}^*} \right)}}{{I\left( M \right) + I\left( {ML} \right) + I\left( {M{L}_2} \right)}}
\end{eqnarray*}

(6) \begin{eqnarray*}
\left[ {\boldsymbol{L}} \right] = {\left[ {\boldsymbol{L}} \right]}_0 - {\left[ {\boldsymbol{L}} \right]}_{{\boldsymbol{\mathrm{ bound}}}} = {\left[ {\boldsymbol{L}} \right]}_0 - \left[ {{\boldsymbol{ML}}} \right] - 2\left[ {{\boldsymbol{M}}{{\boldsymbol{L}}}_2} \right]\\[-8pt]
\end{eqnarray*}

(7) \begin{eqnarray*}
\ ML \rightleftharpoons M + L;{K}_{\mathrm{ D1}} = \frac{{[M][L]}}{{ML}}\\[-4pt]
\end{eqnarray*}

(8) \begin{eqnarray*}
M{L}_2 \rightleftharpoons ML + L;{K}_{\mathrm{ D2}} = \frac{{[ML][L]}}{{[M{L}_2]}}\\[-4pt]
\end{eqnarray*}

(9) \begin{eqnarray*}
\text{fraction}\ \text{of}\ \text{bound}\ \text{DNA} = \frac{{[ML] + [M{L}_2]}}{{[M] + [ML] + [M{L}_2]}}
\end{eqnarray*}

The difference in *K*_D1_ and *K*_D2_ highlights ligand cooperativity. We define a positive cooperativity when *K*_D2_ < 4 *K*_D1_ and negative cooperativity when *K*_D2_ > 4 *K*_D1_ [54].

### ESI-MS titrations

The six sequences 1XAV, 222T, 21G, 5YEY, T24 and ss24 were titrated with seven foldamers: QPQ, QPPQ, QQPQ, QQQ (Q_3_), QQQQ (Q_4_), QQQQQ (Q_5_), and QQQQQQQQ (Q_8_). The MS titration experiments follow two main objectives: (i) Obtain average *K*_D_ values from a set of measurements alongside with a standard deviation, and (ii) study the effect of foldamer length on G4 affinity to aid rational ligand design. We kept the DNA concentration at 10 µM and measured at seven different foldamer concentrations: 0, 5, 10, 15, 20, 30, and 40 µM.

The data processing consists of three steps:

1. Noise correction. Three times the standard deviation of the noise was subtracted from integrated DNA signals. The ligand screening data was noise corrected as well.
2. Response analysis. Response factors depend on the gas-phase transmission yield during electrospray ionization, which can be different for the M, ML, and ML_2_ species. We quantify changes in response by adding dT_6_ as an internal calibrant [55]. The ratio of DNA to dT_6_ intensity as a function of foldamer concentration was monitored and, from changes in that ratio, we derived the effect of foldamer concentration on DNA response (Supplementary Fig. S32). Note that the foldamers do not interact with dT_6_.
3. Dynamic fitting with the DynaFit software (BioKin, MA, USA) [56, 57] which derives a mathematical model from the chemical Equations 7 and 8 and matches it to the experimental data with minimal residual error (Supplementary Fig. S33). Response factors of the complexes (ML, ML_2_) were allowed to vary to reflect effects observed at step 2. *K*_D1_ and *K*_D2_ values were extracted from the fitted data.

### Determination of the number of consecutive G-quartets in ligand-free and ligand-bound forms

K^+^ ions bind to G4 forming sequences in two ways: in-between G-quartets (specific) or elsewhere (unspecific). Unspecific K^+^ ions distribution follows a discrete probability, which can be approximated as a Poisson distribution [58]. Specific K^+^ ions in G4s typically have much higher binding affinities than unspecific ones and have well-defined stoichiometries. An adduct with *n* K^+^ ions is considered specific when it is significantly more populated than its adjacent *n + 1* and *n-1* adducts.

**Figure 2.**
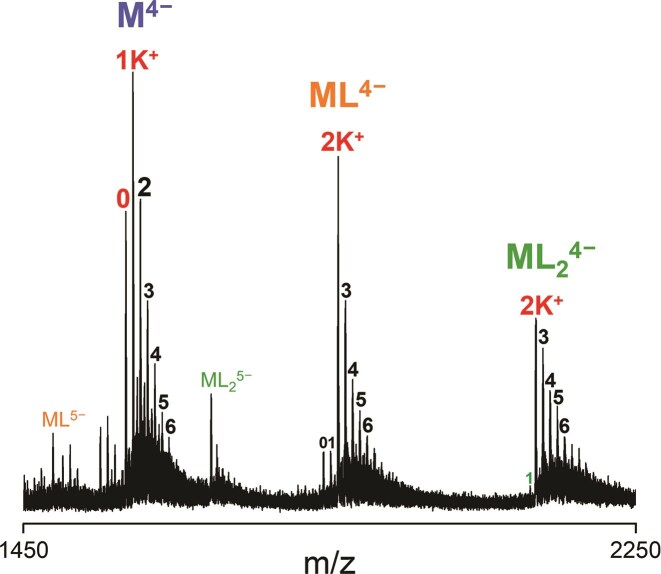
Mass spectrum of 10 µM 222T_mA (dT**G_3_**TT**G_3_**AA**G_3_**TT**G_3_**T) with 20 µM Q_4_ in 0.5 mM KCl and 100 mM TMAA. Labeled species are: Free DNA (M), 1:1 complex (ML) and 2:1 complex (ML_2_), specific K^+^ adducts (red), nonspecific K^+^ adducts (black).

For example, in Fig. 2, the main adduct of the free DNA is 1 K^+^, which corresponds not only to a 2-quartet G4 (antiparallel) [5], but also some unfolded DNA stand with nonspecific potassium binding. The high relative intensity of the unfolded peak (0 K^+^) is an indicator for an unspecific K^+^ adduct distribution blended within all adduct peaks. The main adduct of the complex is 2 K^+^, which corresponds to a 3-quartet parallel G4. The absence of 0 and 1 K^+^ adducts indicates ligand selectivity towards 3-quartet parallel G4s over 2-quartet antiparallel G4s and unfolded DNA.

### X-ray crystallography and structure determination

Crystallization was performed in a sitting-drop well plate (SWISSCI MRC 2 Lens Crystallisation Plate). Droplets were obtained by mixing 200 nL of a solution containing 400 µM DNA (222T), 900 µM foldamer (QQPQ), and 20 mM potassium cacodylate (pH 7.0) with an equivalent volume of crystallization screening solutions from various commercial kits. The original condition was the no. 41 of the (Hampton Research) and was composed of 100 mM KCl, 15 mM MgCl_2_, 50 mM Tris (pH 7.5), and 10% PEG-550. We obtained clear, cuboid crystals that were 10–20 µm in size. The crystals were transferred into a cryoprotective solution containing 100 mM KCl, 15 mM MgCl_2_, 50 mM Tris (pH 7.5), 20% PEG-550, and 20% ethylene glycol. Finally, the crystals were flash frozen at −196°C and analyzed at the SOLEIL synchrotron (Saint-Aubin, France).

Several data sets were collected at the French synchrotron Soleil on the beamline Proxima I to a maximum resolution of 2.51 Å. The raw images were processed with XDS [59]. The structure was solved using the Phaser-Molecular Replacement procedure from the software package Phenix [60]. The structure was solved using the G4 core from the previously solved structure of the 222T sequence (PDB: 6P45) [61]. We tested various models with and without the connecting nucleotide to help remove model bias. We improved the G4 model by several rounds of manual rebuilding and added the QQPQ foldamer once the electron density from the G4 was filled. QQPQ foldamer restrains were generated using the webserver from GlobalPhasing [62], and added to the refinement procedure in Phenix. The final model included two potassium ions located between the G-quartets of the same G4 and one potassium ion located between the G-quartets of two dimerizing G4s coordinated by the guanine bases, several magnesium ions and 18 nucleotides of the G4 sequence (missing only the first and the last thymine nucleotide). The summary of data collection and refinement statistics is presented in Supplementary Table S5. Figures were prepared using ChimeraX [63].

### NMR spectroscopy

NMR spectra were recorded on a 700 MHz Bruker Avance Neo spectrometer equipped with a triple resonance gradient standard probe, or a 800 MHz Bruker Avance Neo spectrometer equipped with a triple resonance cryoprobe. Topspin version 4.1 (Bruker BioSpin) was used for data collection. Processing and analysis for 1D spectra also used Topspin, whereas the 2D spectra were processed using NMRPipe [64], followed by analysis with Sparky (T.D. Goddard and D.G. Kneller, University of California). The parallel and monomorphic G4 sequence T95-2T (dT_2_G_3_TG_3_TG_3_TG_3_T, PDB: 2LK7) was used as a substitute for 222T which displayed poor quality NMR spectra [65]. Identification of key foldamer-binding thymine bases was aided by using three mutated sequences, each of which replaced one thymine with (deoxy)uracil: 2LK7_1U (d**U**TG_3_TG_3_TG_3_TG_3_T), 2LK7_2U (dT**U**G_3_TG_3_TG_3_TG_3_T) and 2LK7_18U (dT_2_G_3_TG_3_TG_3_TG_3_**U**). The 2LK7 and 5YEY NMR samples contained 100 µM DNA in 10 mM potassium phosphate buffer (pH 7, contains 16 mM K^+^) in 90%/10% H_2_O/D_2_O without or with added QQPQ. The 2D ^1^H,^1^H-NOESY used a mixing time of 200 ms, and the ^1^H,^1^H-TOCSY used a mixing time of 60 ms, with both measured at 278 K. Every DNA sequence was measured with and without QQPQ. 1D-NMR spectra of QQPQ alone were measured in d6-DMSO instead of H_2_O/D_2_O and potassium phosphate.

### Quantum chemical and molecular dynamics simulations

Details of methods for torsion scan, conformer search, force field modifications for the foldamer, complex preparation, MD preparation, molecular dynamics and data analysis are described in the supporting information pages S131–S133.

## Results and discussion

### Foldamer specificity for different nucleic acid secondary structures

**Figure 3.**
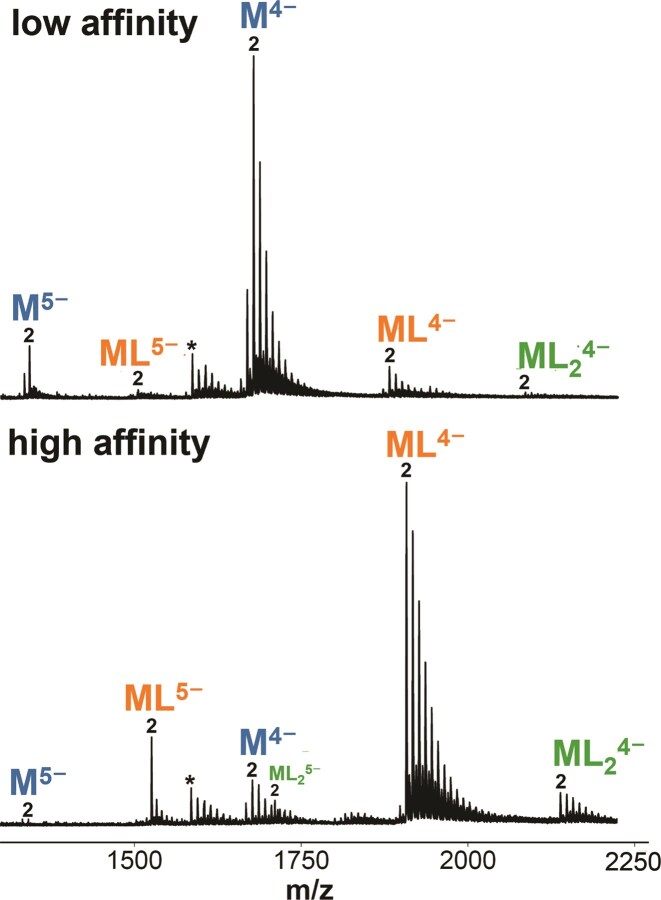
Mass spectra of 10 µM 5YEY (d**G_3_**TTA**G_3_**TTA**G_3_**TTT**G_3_**) with **top**: 20 µM QPPQ. (*K*_D1_ = 75 µM, *K*_D2_ = 40 µM), and **bottom**: 20 µM QQPQ. (*K*_D1_ = 1.5 µM, *K*_D2_ = 22 µM). Sample matrix contains: 0.5 mM KCl and 100 mM TMAA (pH 6.8). Labeled species are: Unbound DNA (M), 1:1 complex (ML), 2:1 complex (ML_2_), 2K^+^ adduct (2), depurinated DNA (*).

To obtain a first ranking of foldamer binding affinities, we recorded native ESI-MS spectra at always the same DNA:foldamer concentration ratio. Although the electrolyte concentrations are not identical to physiological ones, using conditions in which the G4s are less stable has the advantage of better revealing the equilibria at play and the differences between the molecular systems. MS has also the unique advantage of revealing, in the same experiments, details on the stoichiometries of the complexes. For example, in Fig. 3 we can visually compare the mass spectra of foldamers QPPQ and QQPQ with vastly different binding affinities for the same DNA strand 5YEY. The relative abundance of complex (ML) compared to free DNA (M) readily shows that QQPQ binds to the sequence 5YEY with much higher affinity than QPPQ. The calculated *K*_D_ values differ by more than one order of magnitude (Supplementary Table S2).

The low intensity of 2:1 complex compared to the 1:1 complex indicates that the 5YEY G4 only has one accessible binding site. In contrast, foldamer Q_4_ binds to the parallel G4 222T at two binding sites (see Fig. 2).

We carried out this general screening with four foldamers (Q_4_, QQPQ, QPPQ, and QPQ), and 34 DNA sequences that include well-characterized G4 structures and other DNA secondary structures as controls (i-motif, duplexes, and single-stranded DNA). Figure 4A presents the ligand affinity results as a heatmap. The detailed data, including K_D_ values, are in Supplementary Table S2 and the mass spectra (showing the foldamer and K^+^ stoichiometries) are shown in Supplementary Figs S34–S69. Overall, the data indicate that foldamers have a strong preference for parallel G4s and bind very little to DNA controls. Among different foldamers, QQPQ has the most varied affinities and selectivities.

**Figure 4.**
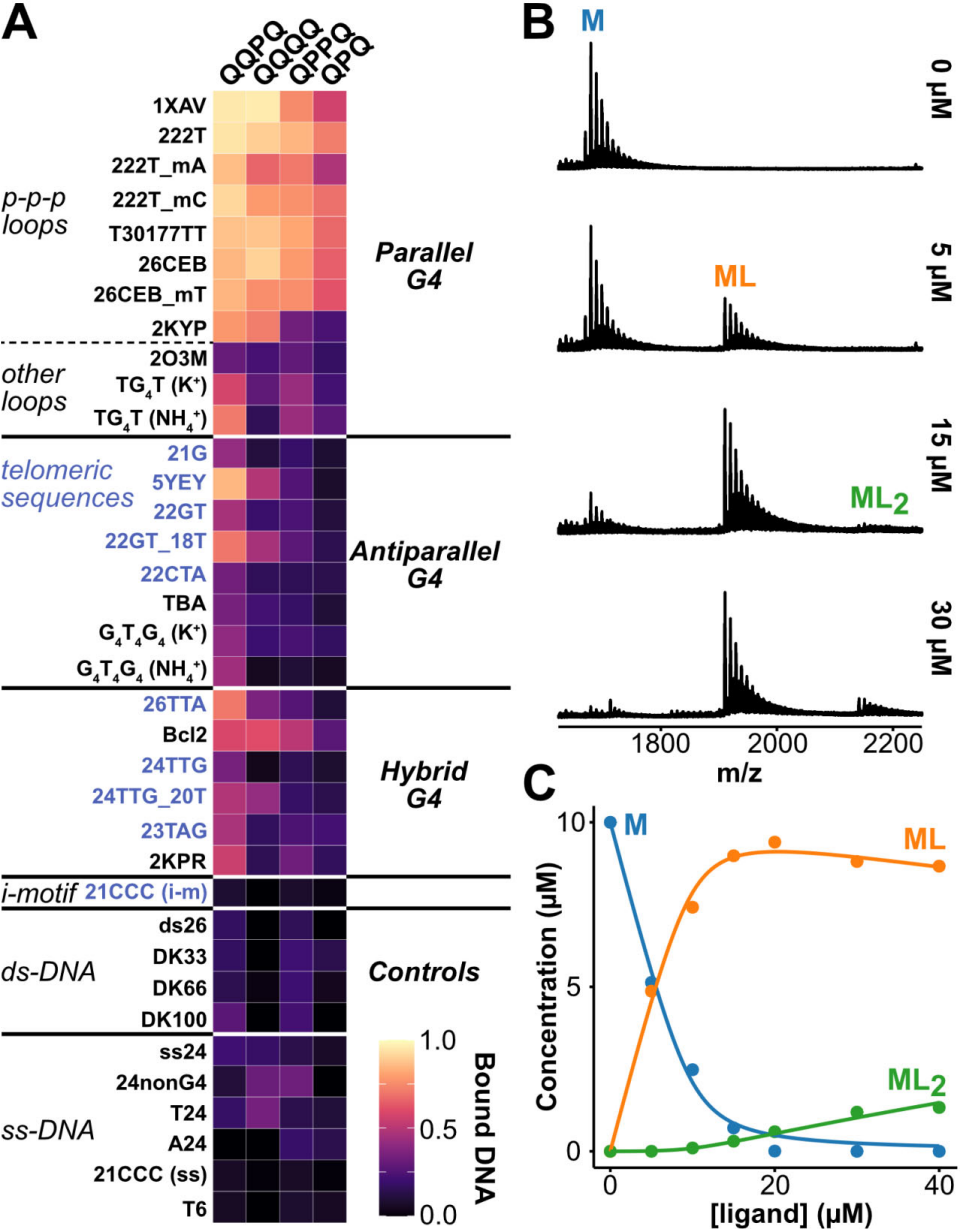
(**A**) Heatmap showing the fraction of DNA bound for all DNA/foldamer combinations, calculated from mass spectra. Sample conditions are: 10 µM DNA, 20 µM foldamer, 0.5 mM KCl, 100 mM TMAA (pH 6.8). DNA sequences are listed in Table 1. Telomeric-related sequences are highlighted in blue. (**B**) Titration data for 5YEY and QQPQ, showing an extract of the raw mass spectra. (**C**) Processed data. The dynamic fitting curves match the experimental datapoints and return *K*_D1_ = 0.54 ± 0.1 µM, *K*_D2_ = 200 ± 70 µM.

All four studied foldamers bind parallel G4s with *K*_D_ values in the low µM range (Supplementary Table S2). But not all parallel G4s are equivalent. First, 222T_mA and 2KYP are partially unfolded in 0.5 mM K^+^. Therefore, there is a mixture of targetable structures (folded G4) and nontargetable structures (unfolded species). This explains that the fraction of bound DNA is lower compared to the other (fully folded) sequences. Next, the G-quartets of 2O3M and TG4T are less exposed than those of common parallel G4s with three propeller loops. 2O3M has a lateral and a snapback loop can obstruct the two terminal G-quartets. TG4T has eight flanking thymines that can form a sparsely populated T4 tetramer, which also protects the G4 from 5′-5′-dimerization [66]. We therefore hypothesize that foldamers bind to the external G-quartets, but that loops/bases covering the terminal quartets will impede foldamer binding for steric reasons.

QPQ and QPPQ are highly selective to parallel G4s with three propeller loops but have moderate affinities. These foldamers are more likely crescent-shaped than helical, because the quinoline-oligomer chain is not long enough (it takes five Q units for two helical turns) [34]. QQPQ and Q_4_ are more stable helices; as indicated by the network of intramolecular H-bonds for QQPQ by NOESY (see NMR section). Their affinity to parallel G4s is three to four times higher.

Apart from parallel G4s, the QQPQ foldamer tightly binds to 5YEY (*K*_D1_ = 1.5 µM), but also to TG4T, 22GT_18T, and 26TTA (*K*_D1_ ≈ 5 µM). 5YEY and 22GT_18T are telomeric mutants with a TTA→TTT mutation in the third loop, which induces a switch from a 2-quartet to a 3-quartet antiparallel G4 [67]. QQPQ and Q_4_ are sensitive to this structural switch: The affinities to the mutants is 5–10 times higher than to the ‘wild-type’ sequences (compare: 21G/5YEY, 22GT/22GT_18T, 24TTG/24TTG_20T). 26TTA is a hybrid-2 G4 that is targeted by QQPQ but no other foldamer in this panel. We cannot assess whether QQPQ is selective towards hybrid-2 over hybrid-1 topology.

None of the foldamers interact well with antiparallel G4s, where the G-quartets are usually covered by base triads and/or diagonal loops on both sides (21G, 22GT, 22CTA, TBA, G4T4G4). This finding confirms that foldamers selectively target G4s whose configuration allows exposed G-quartets. The low affinity towards alternative DNA structures (i-motif, duplex, single strand) underlines the G4 specificity of foldamers, except for very specific cases described below. The three-dimensional shape of foldamers prevents duplex intercalation, as they are too bulky to lodge in between base pairs [46]. Among the control DNA sequences, the foldamers were irresponsive to changes in G-richness, molecularity or strand length.

### Characterization of affinities with ESI-MS titrations

To further characterize binding affinities and stoichiometries, we selected six DNA sequences (two single strands, two parallel G4s, and two derivatives of the telomeric sequences that differ by a single base), and seven foldamers: QPQ, QQPQ, QPPQ, Q_3_, Q_4_, Q_5_ and Q_8_. Figure 4B shows titration data for 5YEY and QQPQ. All *K*_D_ values are listed in Supplementary Table S3, and estimated response factors of each DNA:Foldamer complex are in Supplementary Table S4. The full titration datasets are in Supplementary Figs S70–S110.

The control sequence ss24 is a single-stranded 24-mer with 25% of each nucleobase. None of the foldamers target this sequence (*K*_D1_ > 100 µM) save for Q_3_, which has significant populations of 1:1 complex, 2:1 complex, and 3:1 complex (Supplementary Fig. S98). Q_3_ is thus the least G4-specific ligand in the panel. T24 is another single-stranded control sequence that does not interact with QPQ, QQPQ, or QPPQ (*K*_D_ > 100 µM), but it attracted our attention during the screening with Q_4_. Intriguingly, it forms high-affinity 2:1 complex with Q_3_, Q_4_, and Q_5_ (*K*_D2_ < 1 µM), including a 4:1 complex with Q_3_. Although our foldamers are racemic and display no CD signal on their own, the induced CD in the presence of T24 matches the CD signature of a right-handed foldamer helix (Supplementary Fig. S111). Our hypothesis is that T24 and Q_n_ foldamers associate into a right-handed double helix, wherein the foldamer is dimerized in order to accommodate the full length of the T24 strand. No other oligonucleotide target led to induced CD of the foldamers, for which the binding of both their enantiomers are likely similar and very dynamic (see X-ray crystallography and MD results, below). We also tried the RNA counterpart U24 (Supplementary Fig. S112), because U-rich motifs play a role in RNA expression [68, 69], but there was no induced CD in U24 upon adding Q_n_ foldamers.

The parallel G4s 1XAV and 222T show the highest affinity. All foldamers form high-affinity 1:1 complex with 222T (*K*_D1_ < 10 µM). The strongest binding partner for 1XAV is Q_4_ (*K*_D1_ = 0.075 ± 0.059 µM), the weakest one is Q_8_ (*K*_D1_ = 28 ± 6.7 µM). All foldamers form 2:1 complex (*K*_D2_ ≈ 1 µM), except for Q_8_ and QPQ. Q_3_ is the only foldamer to form a 3:1 complex, but based on our results with a control sequence ss24, the interaction might not be G4 specific.

Finally, the two telomeric sequences, 21G and 5YEY, are identical, save one nucleotide in the third loop (A in 21G and T in 5YEY). This mutation, however, changes the polymorphic 21G to the antiparallel 5YEY. Q_5_, Q_4_, and QQPQ form a stable 1:1 complex with 5YEY (*K*_D1_ < 10 µM), but not 21G. Q_4_ is the most selective foldamer, with a 20-fold change in *K*_D1_ between 21G and 5YEY. We also compared the sequences 22GT (antiparallel with 2 quartets) and 24TTG (very stable hybrid-1 G4) and the corresponding sequences with the same A$\rightarrow$T mutation (22GT_18T and 24TTG_20T), and observed the same increase of affinity for the sequence variant containing the thymine (Fig. 4A).

Overall, the ESI-MS titrations indicate that foldamers have the highest affinity (in the low/sub-µM range) towards parallel G4s (1XAV and 222T), alongside a few notable exceptions (5YEY, T24). Foldamer length is important and there seems to be a ‘sweet spot’ at about 4–5 monomers. Having only three monomers comes with a decline in affinity (QPQ) or G4 specificity (Q_3_). Having over five monomers leads to no affinity increase, but loss of DNA signal that scales with increasing number of Q monomers (Supplementary Fig. S32). The reason is that each Q monomer adds a positive charge to the foldamer, which reduces charge repulsion among DNA polyanions, promoting DNA aggregation. We confirm this hypothesis from (i) seeing precipitation in concentrated DNA solution when an excess of foldamer is added , and (ii) loss of CD signature for a stable (i.e. resistant to disruption) parallel G4 when Q_8_ is added (Supplementary Fig. S113).

### Preferred topologies and foldamer-induced topology switching

**Figure 5.**
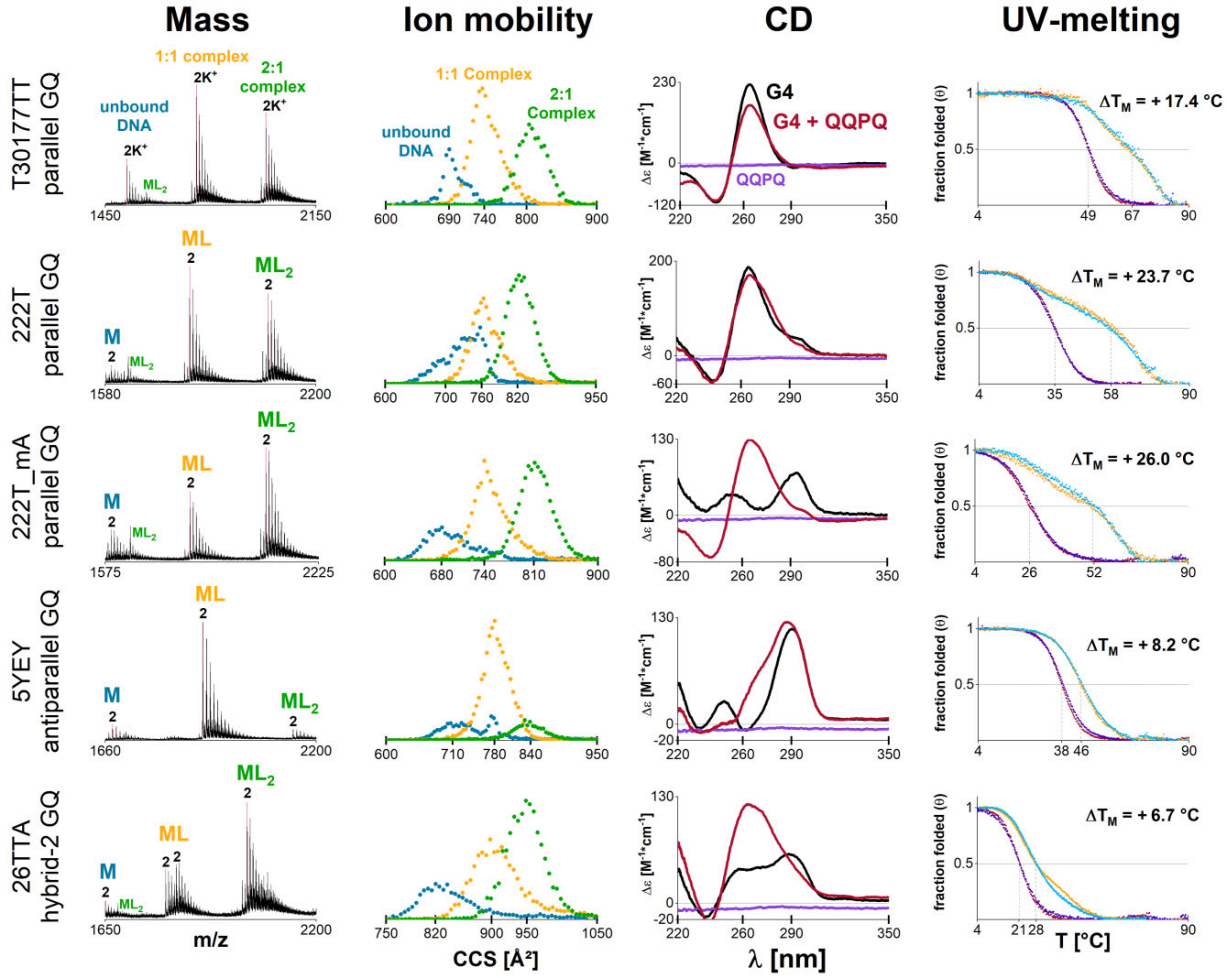
The effect of foldamer QQPQ on five different G4s. Samples contain 10 µM DNA, 20 µM QQPQ, 0.5 mM KCl, 100 mM TMAA (pH 6.8). First column: Mass spectra showing the 4- charge state (5- for 26TTA). Second column: CCS distributions extracted from the 2K^+^ adduct of each species, highlighted with a red line in the corresponding mass spectrum (in column 1). Third column: CD spectra of the complex (red) and control spectra with free DNA (black) and free foldamer (purple). Fourth column: UV-melting curves with foldamer (heating: orange; cooling: cyan) and without foldamer (heating: crimson, cooling: navy).

Here we focused on a single most promising foldamer, QQPQ and five G4 DNA with differing topologies. We selected QQPQ because (i) it was the foldamer with the highest G4 affinity and (ii) we were intrigued by its ability to selectively target some telomeric repeat sequences. We selected telomeric G4 26TTA which adopts hybrid-2 topology and 5YEY, which is antiparallel. We also picked T30177TT as a stable parallel-stranded G4; 222T and 222T_mA which are polymorphic in 0.5 mM K^+^ with a mix of 3-quartet parallel and 2-quartet antiparallel G4 [5]. The equilibrium of 222T in our experimental conditions is shifted towards the parallel form, while the folding of 222T_mA is shifted towards the antiparallel form. We characterized all complexes by MS, IMS, CD, and thermal stability, and present selected results in Fig. 5.

MS provides a quick readout on ligand stoichiometry, potassium binding stoichiometry and the quantity of each species. The number of specific K^+^ ions correlates to the number of G-quartets. IMS lets us track changes in structure even when there is no change in mass. The ions’ CCS depends on the ion size, shape and charge state (within constant experimental settings). Adding ligands is increasing the size of the complex and is expected to lead to a CCS increment. In addition, significant conformational differences for a given size are expected to lead to significant differences in ion mobility, resulting in different CCS distributions. Thus, the shape of the CCS distribution for each ligand stoichiometry indicates how many topologies could be present in solution. CD signatures of DNA alone and with foldamer informs on how the foldamer affects the G4 topology. Finally, the difference in melting temperature with and without foldamer (Δ*T*_m_) provides a quick snapshot of how potent a ligand is at stabilizing the G4 structure. When there are sufficiently populated species with significantly different melting temperatures, multiple melting transitions are visible.

For T30177TT, 2 K^+^ is the dominant adduct in the mass spectra. It has a parallel CD signature and is 100% folded at 25°C. We are thus dealing with a stable, 3-quartet parallel G4. Adding foldamer does not change the K^+^ adduct distribution, the shape of the CCS distribution or the CD signature, meaning that foldamer binding does not significantly change the G4 structure. The slight drop in CD intensity is likely due to foldamer-induced DNA aggregation (Supplementary Fig. S113). The melting temperature of T30177TT increases by 17°C in the presence of a 2-fold excess of QQPQ, but two distinct melting transitions appear. We know from previous temperature-controlled MS studies on G4 ligands that the 1:1 complex usually melts before the 2:1 complex [70]. This situation could apply here as well.

222T and 222T_mA are polymorphic in 0.5 mM K^+^. The CCS distributions of free DNA are therefore the sum of multiple Gaussian functions, each representing a different set of conformers. Upon foldamer binding, the CCS distributions are less convoluted (i.e. they look more like a single Gaussian distribution), highlighting that the foldamer selectively targets certain conformers within the conformational ensemble of polymorphic sequences. Figure 5 shows that the complexes (ML/ML_2_) have 2 specific K^+^ ions, therefore the complex must be a 3-quartet G4. QQPQ shifts the CD spectra towards a parallel signature (222T_mA has a remarkable signature switch). The foldamer targets parallel G4 and stabilizes it with Δ*T*_m_ ≈ +26°C). Similar to T30177TT, the DNA/foldamer system has two separated, broadened melting transitions which likely correspond to unfolding of 1:1 and 2:1 complexes.

26TTA mirrors the behavior of 222T/222T_mA: The K^+^ adduct distribution shifts towards 2K^+^, a parallel CD signature emerges and the UV melting transition broadens in the presence of QQPQ. The foldamer promotes a structural rearrangement of 26TTA, probably from hybrid to mostly parallel topology. As a result, the complex has two binding sites.

The antiparallel 5YEY has only one accessible binding site, as the intensity of 1:1 complex is significantly higher than that of 2:1 complex in the mass spectrum (see also the titration in Supplementary Fig. S87). The foldamer affects G4 folding, causing a CD band at 260 nm to increase. The CD signature indicates that the 1:1 complex could be a hybrid G4, but the CCS distributions give no conclusive evidence whether there is only one hybrid species or multiple species with different topologies. The affinity of the 1:1 complex between 5YEY and QQPQ (*K*_D1_ = 0.54 ± 0.1 µM) is comparable to that observed for parallel G4s. The melting curve is steeper and shifts less (Δ*T*_m_ = +8.2°C) compared to those for parallel G4s. That implies that the complex formation with 5YEY is more enthalpy-driven [50].

### Origin of selectivity for parallel G4s

#### X-ray crystallography

**Figure 6.**
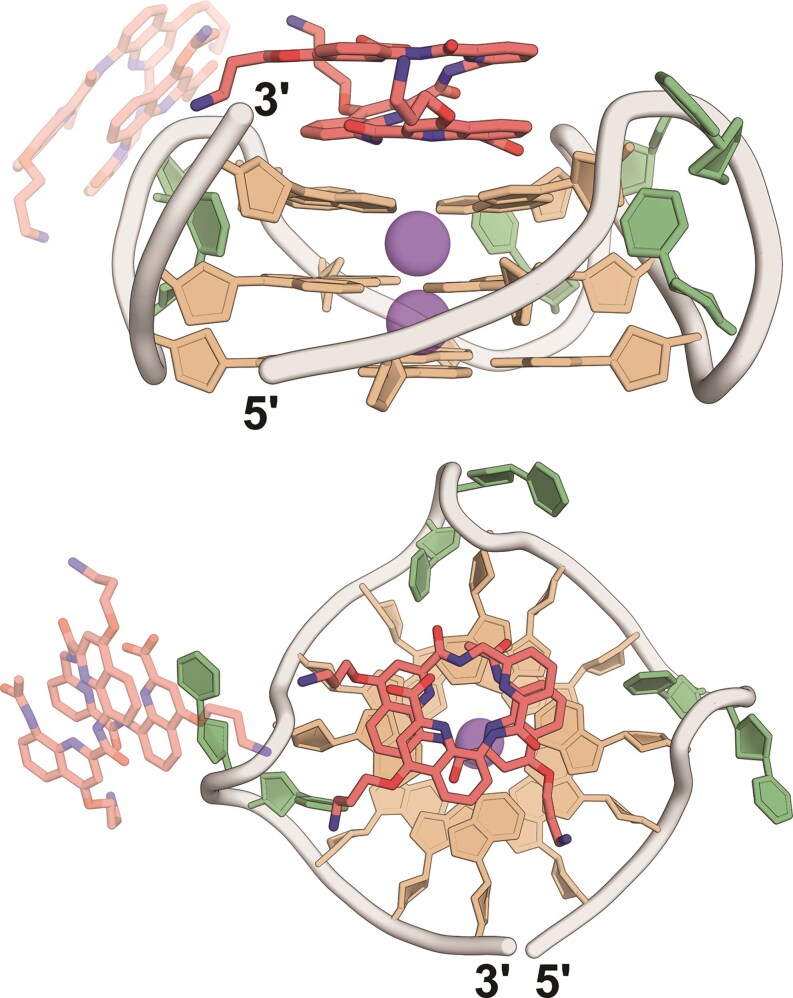
X-ray crystal structure of G4 222T (dTG_3_TTG_3_TTG_3_TTG_3_T) and foldamer QQPQ (PDB entry code: 8QN2) from the side (top) and from above (bottom). Color coding: two QQPQ molecules in red (the binding site not existing in solution phase is set to 50% transparency), guanine in tan, thymine in green, potassium in purple. The crystallization matrix contains 100 mM KCl, 15 mM MgCl_2_, 50 mM Tris (pH 7.5), 10% PEG-550, and a 9:4 ratio of foldamer:DNA.

We used X-ray crystallography to test the end-stacking hypothesis based on the CD and native MS results. Molecular crowding induces a bias for the parallel topology [71, 72], so we selected the parallel G4 sequences 1XAV and 222T to maintain the solution-phase topology and promote crystallization with QQPQ or Q_4_. We also studied the 5YEY/QQPQ and T24/Q_4_ complexes due to their specific binding properties that could provide additional mechanistic insights. We only obtained crystals for the 222T/QQPQ complex, whose structure is shown in Fig. 6. Atomic and diffraction data have been deposited in the PDB under the accession code: 8QN2. Crystal and structure refinement information is deposited in Supplementary Table S5. Electron density maps are shown in Supplementary Figs S114–S116.

222T adopts a parallel-stranded topology and forms a 5′-5′ crystallographic dimer. Two QQPQ foldamers interact with one 222T molecule. The first QQPQ has two quinolines π-stacking onto the 3′ G-quartet. The QQ unit is flattened at the binding site to allow more contact area for π-stacking. We suspect that the P unit adds flexibility through its methylene group, which can freely rotate. The flexibility is needed to reduce molecular tension in the foldamer helix caused by the QQ pair flattening. Such molecular architecture explains why QQPQ has the highest G4 affinity: It has two Qs for π-stacking, followed by a P which acts as a step, after which the molecule can resume its helical shape. The top view illustrates how the diameter of the foldamer helix matches the size of a G-quartet. The propeller loops are not limiting the foldamer’s access to the G-quartets and therefore do not interfere with foldamer binding.

According to our ESI-MS titrations, the 222T/QQPQ complex is saturated in a 3-fold excess of foldamer, where it forms a 2:1 complex (Supplementary Fig. S80). We expected to have one ligand on each end of the G4. However, the 5′ interface is utilized for G4 dimer formation and the second foldamer instead stacks onto a thymine base of a TT loop (Fig. 6). Parallel G4s tend to dimerize in concentrated solution because of their exposed G-quartets. The preferred interface for dimerization is 5′ to 5′ end [73, 74]. The foldamer sitting atop the thymine establishes a contact with a neighboring unit cell (Supplementary Fig. S117). Such crystal contacts are needed to promote crystal formation and growth, but they can only persist in the solid phase.

The electron density map (standard 2mFo-DFc at the 1 sigma level) of the G4 is well-resolved (Supplementary Fig. S114), but the electron density of the foldamer is less defined (high B-factors, see Supplementary Fig. S116), suggesting high dynamics. Several factors can contribute to these dynamics: (i) rotational freedom along the helical axis, (ii) exchanges between left- and right-handed helix, since the foldamer is racemic, (iii) the foldamer latching onto the G-quartet from its C-terminus or N-terminus, and (iv) conformational freedom of the foldamer side chains. We tested models where either the C- or N-terminus of QQPQ interacts with the 3′ G-quartet (Supplementary Fig. S118) and found no significant change in refinement statistics that would suggest any C/N-terminal arrangement being more favorable than the others. So even though the crystal structure depicts a static image of the foldamer-DNA-complex, dynamic exchanges are expected in solution.

In summary, we have validated one binding site being the 3′ G-quartet and the binding mode being π-stacking but need supporting evidence for the second binding site and the foldamer orientation.

#### NMR spectroscopy

In order to complement the X-ray structural studies with observations in the solution state, we opted to use NMR spectroscopy to investigate foldamer binding to G4 DNA. Due to difficulties in obtaining well-resolved NMR spectra with 222T, we selected the similar 2LK7 (dTT**G_3_**T**G_3_**T**G_3_**T**G_3_**T). The structure of 2LK7 resembles 222T, but is more dynamically constrained (1-nt loops) and protected from dimerization (TT motif at 5′) [65]. In addition, QQPQ also targets 2LK7 with sub-µM affinity (Supplementary Fig. S119 and Supplementary Table S3). All 1D and 2D NMR spectra of 2LK7/QQPQ are gathered in the supporting information (Supplementary Figs S120–S135), with key findings summarized in Fig. 7.

**Figure 7.**
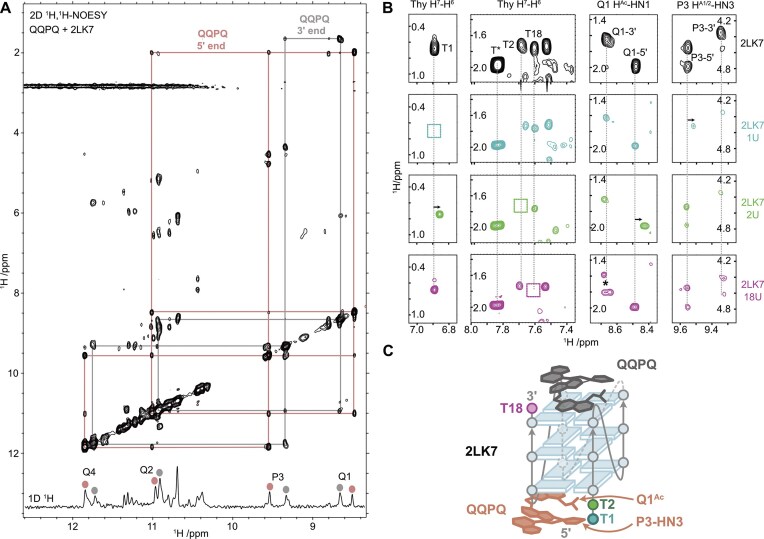
(**A**) 2D ^1^H,^1^H-NOESY (200 ms mixing time) of 2LK7 (dTTG_3_TG_3_TG_3_TG_3_T) 100 µM, 10 mM KP buffer, pH 7.0, in 90/10 H2O/D2O) in the presence of QQPQ (300 µM), showing two NOE crosspeak networks connecting the foldamer amide protons (NH). 1D NMR imino region (bottom insert) annotated with the two foldamer species amides. Cycle and proton naming conventions are given in Supplementary Fig. S120. (**B**) A series of selected regions from the ^1^H,^1^H-NOESY of QQPQ bound to 2LK7, 2LK7-1U, 2LK7-2U and 2LK7-18U. Vertical lines indicate the original thymine H6, QQPQ Q1 NH1, and QQPQ P3 NH3 chemical shifts. Boxes highlight the absence of peaks observed when the thymidine is replaced with deoxyuridine. The full spectrum is shown in Supplementary Fig. S135. (**C**) Scheme of the 2:1 QQPQ:2LK7 complex, where QQPQ is shown in salmon and gray for the 5′ and 3′ binding sites, respectively. The T1, T2 and T18 residues are colored as in panel (B).

From the NMR spectra, we were first able to assign the amide protons of the foldamer (designated *NH1* to *NH4* in Supplementary Fig. S120) from a ^1^H-^1^H NOESY spectrum of the 2LK7/QQPQ complex (Fig. 7A). Instead of a single bound foldamer species, we observed two NOESY-connected systems. The fact that the NOE crosspeaks are visible confirms that the foldamer in both cases has an overall folded helical arrangement with the amide protons in close proximity and protected from solvent exchange via hydrogen bonds. However, the presence of two foldamer species further complicated an already complex 2LK7/QQPQ NMR spectra and we were unable to perform sufficient chemical shift assignment to determine atomic details of the complex.

As an alternate approach, we decided to probe the structure by observing the chemical shift perturbation upon specific removal of three different 2LK7 thymidine methyl groups by replacement with deoxyuridine (2LK7-1U, 2LK7-2U, and 2LK7-18U). Selected regions of the ^1^H-^1^H NOESY spectrum 2LK7/QQPQ illustrates the initial position of thymine methyl group NOE crosspeaks as well as foldamer amides from Q1 (*NH1*) and P3 (*NH3*) (Fig. 7B; top row, black spectrum). Comparison to the corresponding regions for 2LK7-1U/QQPQ confirms the loss of the T1 methyl crosspeak, with a small shift of the neighboring T2 methyl as well as one of the P3 foldamer species (Fig. 7B, second row). Similarly, the spectrum for 2LK7-2U/QQPQ confirms the loss of the T2 methyl group, with a small shift in neighboring T1 and this time a different amide, Q1, from the foldamer (Fig. 7B, third row). Taken together, this places one of the bound foldamer species on the 5′ face of 2LK7, with the N-terminus of QQPQ at the interface (Fig. 7C). The spectrum of 2LK7-18U/QQPQ confirms the loss of the T18 methyl group, with a mix of peaks replacing the Q1 amide crosspeak of the second foldamer species (Fig. 7B, fourth row). These findings place the second foldamer species on the 3′ face of 2LK7, and again likely with the N-terminus of QQPQ at the interface. The presence of multiple crosspeaks may arise from conformational heterogeneity of the bound foldamer position, or implicate the T18 methyl group as a critical component of the interaction. Finally, all three loop thymines (T6, T10, T14) are unaffected by removing any of the methyl groups, and also do not change upon QQPQ binding (Supplementary Fig. S135). This finding is consistent with the quadruplex loops playing no direct role in foldamer binding. In summary, the characterization of 2LK7/QQPQ in the solution state supports a model in which two foldamers can bind on opposite faces of a single quadruplex molecule, in agreement with the 2:1 complex observed in the mass spectra (Supplementary Fig. S119).

### Specificity for variants of the telomeric sequence

**Figure 8.**
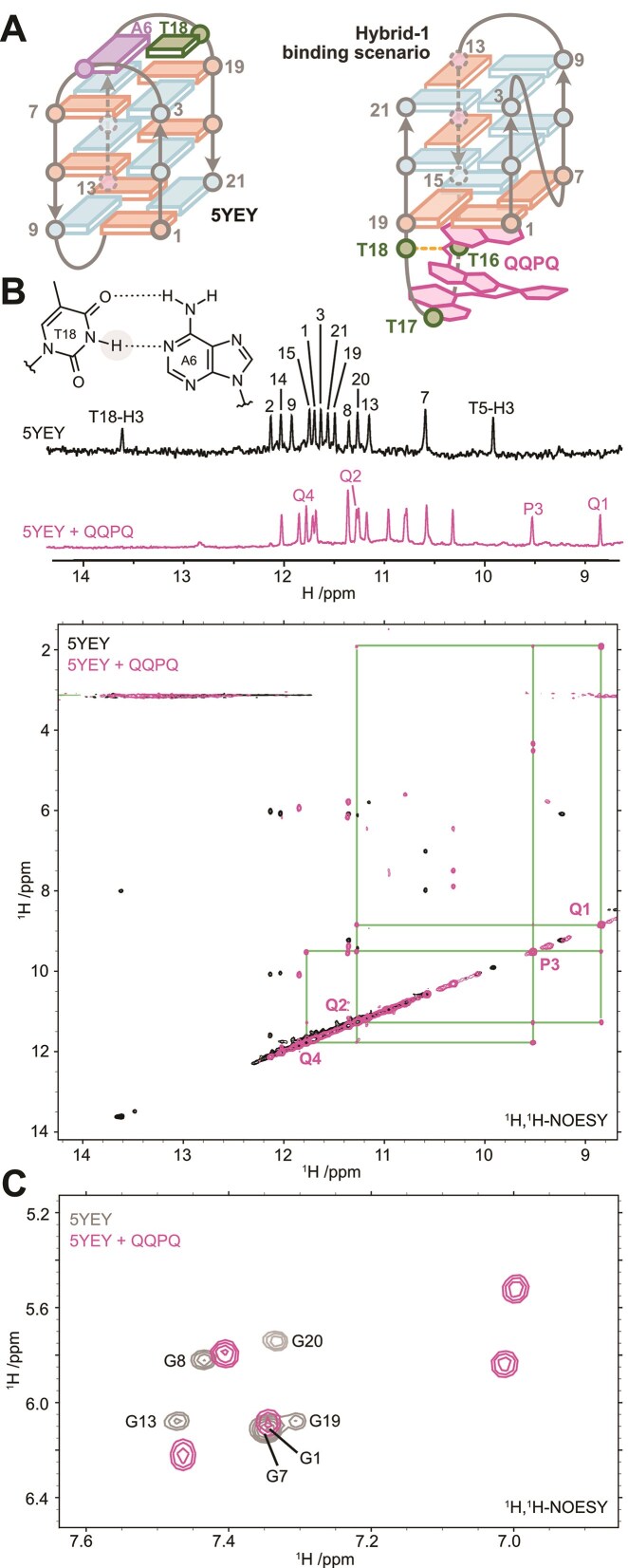
(**A**) Binding of QQPQ to 5YEY (dG_3_TTAG_3_TTAG_3_TTTG_3_) displaces the equilibrium towards a different fold, likely a hybrid-1 topology, where the A6:T18 base pair is disrupted (syn and anti guanine conformations are shown in blue and salmon, respectively, with thymines in green, adenines in purple and QQPQ in pink). (**B**) Top: Imino region of the 1D ^1^H NMR spectra of 5YEY (100 µM) in absence (black) or presence (pink; 100 µM) of QQPQ, in 10 mM KP buffer (pH 7.0), 90%/10% H_2_O/D_2_O. Bottom: Corresponding 2D ^1^H-^1^H-NOESY annotated with the single NOE crosspeak network connecting the foldamer amide protons (NH). (**C**) 2D ^1^H,^1^H-NOESY showing the crosspeaks between H1’ and H8 of guanines with contour level raised to show only the more intense syn crosspeaks in the absence (gray) or presence (pink) of QQPQ. The six unbound 5YEY syn-G H1’-H8 NOE crosspeaks are labelled via comparison to the deposited chemical shifts (BMRB accession number 36116) [75]. The five syn-G H1’-H8 NOE crosspeaks for QQPQ-bound 5YEY were not assigned.

In the above characterization of the foldamer-bound G4s based on the telomeric sequence, we observed intriguing specificities for 5YEY, for sequences wherein the third loop is TTT, and also for 26TTA. This latter G4 is not very stable in MS-compatible K^+^ solutions, and therefore the quadruplex can possibly switch its conformation towards a parallel-stranded topology. For 5YEY, our data indicate that QQPQ induces significant base stacking changes in 5YEY (Fig. 5). We were thus curious about the structure formed by the 5YEY-QQPQ complex. Unfortunately, we could not crystalize the complex, but we were able to probe the solution states of the free and bound 5YEY by NMR spectroscopy (Fig. 8). For these experiments, a 1:1 QQPQ:5YEY ratio was maintained, as higher ligand concentrations resulted in precipitation.

NOESY data indicate that only one foldamer NOE network is present (Fig. 8B), which reaffirms the 1:1 complex seen in our ESI-MS results (Fig. 5). The NOE crosspeak network and NMR chemical shifts match well with the QQPQ 5′ network in Fig. 7A, where the foldamer was bound at the 5′ end. Although this observation does not prove that in 5YEY the binding site is also located on the 5′ end, it is consistent with this interpretation.

The 5YEY structure contains an A:T base pair formed between A6 in the first loop and the mutated T18 in the third loop (Fig. 8A and B) [75]. A clear indicator of the A:T base pair in the NMR spectrum is the observation of the H-bonded H3 amide proton of T18 within the chemical shift range typical of Watson–Crick base-paired thymine (Fig. 8B, top spectrum). In the presence of QQPQ, this distinctive peak disappears from this characteristic region (Fig. 8B, bottom spectrum), indicating that the 5YEY/QQPQ complex no longer contains the A:T base pair. Our MS data show 2 specific K^+^ ions for both free 5YEY and the 5YEY/QQPQ complex, therefore both free and bound 5YEY have three G-quartets, but both the circular dichroism spectrum and the ion mobility data suggest that a topology switch could have occurred. Finally, we also recall that the only non-G4 forming sequence to which foldamers significantly bind is the polythymine dT_24_.

We can imagine two scenarios of QQPQ binding to 5YEY. The first scenario is that the G4 topology of 5YEY remains unchanged. The A:T base pair dissociates to make space for the foldamer helix, allowing it to π-stack onto the 5′-opposing G-quartet. However, foldamer binding by stacking on the quartet on the same side of two lateral loops is difficult to reconcile with the observation that, in parallel G4s, steric hindrance on the terminal quartets diminishes the foldamer affinity. Given that the number of *syn* guanines decreases in the presence of QQPQ (Fig. 8C), a scenario in which the G4 undergoes structural rearrangement is more likely. Time-dependent CD spectroscopy shows the formation of G-quartet stacking typical of hybrid structures, requiring 5 h to complete (Supplementary Fig. S136), which supports a full unfolding of the oligonucleotide before refolding. Hybrid structures typically have a lateral loop on each side of the G-stack core, but recalling the non-negligible affinity of foldamers for polythymines (such as dT_24_), we suggest that the G-quartet on the side of thymine loops (and T or TT overhangs in 22GT_18T and 24TTG_20T) could be the foldamer binding site (Fig. 8A).

### Molecular dynamics of the complexes

Crystallography experiments indicate that the bound QQPQ remains quite dynamic, showing no clear preference for a specific enantiomer and binding through either its N- or C-terminus. Additionally, these experiments did not allow observation of binding at the 5′-end of the 222T due to its dimerization, nor the peculiar binding to 5YEY. To gain further insights into these aspects, we conducted GPU-enabled one-microsecond molecular dynamics (MD) simulations in 100 mM KCl, using the OL21 Amber force field with the Amber software package and subsequent analysis with the bio3d R package (see methods in supporting information) [76–87]. Force field parameters for QQPQ were derived from quantum mechanical (QM) geometry optimization, followed by two-stage Restrained Electrostatic Potential atomic charge calculations [88–107]. A QM-level scan of the amide bond dihedral angles confirmed the energetic preference for the helical conformation in water (Supplementary Fig. S137). Further exploration via global geometry optimization with the GOAT algorithm implemented in Orca did not identify any significantly structural difference among the 133 most stable conformers (Supplementary Table S6 and Supplementary Fig. S138) [108–111]. The 1:1 complex from the crystal structure served as a reference, with 222T as a monomer and without the second foldamer interacting with a neighboring unit cell. Based on this reference complex, three other 1:1 complexes were prepared wherein QQPQ binds through its C-terminus (3CM) or as the (P) enantiomer (3NP and 3CP). Additionally, four 2:1 complexes were prepared to study the 5′-end binding: 3NP/5NP, 3NP/5CP, 3NM/5NM, and 3NM/5CM (Supplementary Fig. S139).

MD simulations show that 222T can accommodate QQPQ on both end-quartets without causing steric hindrance or structural modification to either partner (Supplementary Figs S140–S143). In particular, QQPQ retains its helicity. Both enantiomers bind through their N- and C-termini without clear preference, consistent with the extensive binding and absence of induced CD. Principal component analysis followed by *k*-means clustering of the structures from the trajectories show that, for all models, QQPQ is positioned off-center relative to the tetrad, optimizing the stacking overlap given its smaller helical radius (Supplementary Figs S144–S155). The two aromatic rings nearest the tetrad (two Q or one Q and one P for N- and C-terminal binding, respectively) are frequently co-planar to enhance π-stacking interactions, though not always. This variability likely reflects the intrinsic helicity of QQPQ, characteristic of this foldamer class and supported by both crystallographic data and QM optimizations. The side chains can bind to the backbone and, to a lower extent, sugars (Supplementary Figs S156–S159). When QQPQ is bound at the 3′-end, the side chains can establish H-bonds with the O4 and O2 acceptors of loop and terminal thymines, but this is almost never the case at the 5′-end (Supplementary Fig. S160).

**Figure 9.**
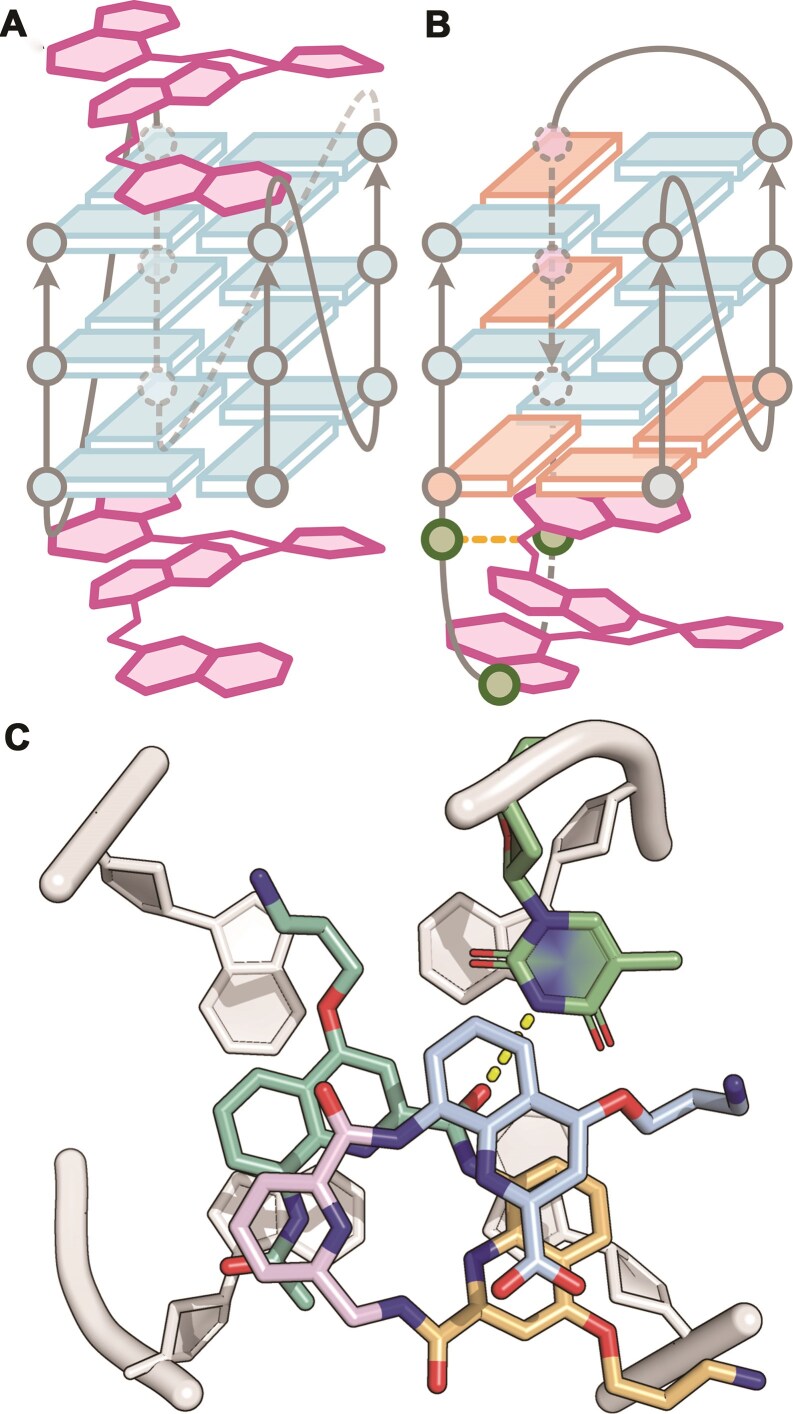
Scheme of the deduced foldamer ligand binding modes. (**A**) Stacking on terminal quartets of a parallel sequence (e.g. 222T), when they are accessible. (**B**) Stacking on a terminal quartet of a nonparallel sequence (e.g. 5YEY), with loop thymines in proximity. (**C**) A snapshot of molecular dynamics started from the crystal structure 8QN2, showing a transient hydrogen bond between an outward pointing amide carbonyl of the foldamer and the N3–H group of a thymine.

The dynamics of QQPQ is responsible for most of the trajectories’ variance (Supplementary Figs S145 and S146). A notable finding is that QQPQ can rotate along its helical axis (Supplementary Figs S148–S155 and S161–S163), sometimes completing a full rotation within a microsecond of simulation (Supplementary Fig. S164), while keeping optimal interplanar angles for π-stacking (Supplementary Figs S165–S167). Additionally, the side chains exhibit significant dynamics and do not form long-lasting interactions with DNA (Supplementary Figs S156 and S157). These observations likely account for the poor electron density of QQPQ in crystallography. Similarly, the 5′ and 3′ dT are highly dynamic, which explains their absence from the crystal data. However, there were a few instances where their N3 H-bound to the amide oxygen of QQPQ, supporting a particular affinity for thymines that has been noted previously (Fig. 9). This is exemplified in the 3NM/5CM system, where QQPQ binds at the 5′-end by forming a hydrogen bond between its O6 atom and the N3 of dT1 (Supplementary Fig. S168A; 250 ns). This interaction is notably stable, persisting for over 630 ns (Supplementary Fig. S168B), and consequently restricting the rotational freedom of QQPQ, after which the interaction is disrupted and QQPQ rotates (Supplementary Fig. S163; see 3NM/5CM: Q4/G12). Disruption of this specific interaction re-establishes the rotational freedom of QQPQ, yielding different binding arrangements (Supplementary Fig. S168A; 965 ns).

Finally, we investigated the binding of QQPQ to 5YEY. A complex was assembled from the hybrid 1 structure of 2JSM, adjusted to match the sequence of 5YEY and capped with QQPQ above the 5′-quartet. The MD trajectory was particularly stable (Supplementary Fig. S169) and provided two key observations supporting the conversion of 5YEY to a hybrid 1 topology with the disruption of the A6:T18 base pair. First, the 5′-quartet of 5YEY can accommodate QQPQ, as it is only occupied by the T16-T17-T18 lateral loop, which uses minimal space in the absence of the A6:T18 base pair (Supplementary Fig. S170A). Second, the disruption of this base pair allows for the formation of a stable hydrogen bond between the N3 atom of T18 and the amide connecting the N-terminus quinolines of QQPQ (Supplementary Fig. S170A and B). Again, this binding mode is similar to that of the 5′ T1 with 222T described above (Supplementary Fig. S168), and is consistent with the proximity of QQPQ and T2, when the former stacks on the 5′-end of 2LK7 (Fig. 7C). Finally, T17 stacks over the C-terminus quinoline of QQPQ, further stabilizing the complex (Supplementary Fig. S170A).

The sequence and topology preference of oligoamide quinoline/pyridine containing foldamers can be summarized in Fig. 9. The foldamers can stack on terminal G-quartets, preferably when they are sterically accessible. If the two termini are accessible, 2:1 complexes can form (Fig. 9A). Exceptionally, binding to some sequences forming nonparallel stranded structures can also occur (e.g. Fig. 9B, proposed topology for the 1:1 complex between the 5YEY sequence and QQPQ), provided that there are thymines in the loop, because thymines can form hydrogen bonds with the outward-pointing amide carbonyl groups of the foldamer (Fig. 9C, a snapshot extracted from MD simulations).

## Conclusions

Our comprehensive survey of the interaction between G4s and small oligoamide quinoline/pyridine foldamers reveals exciting opportunities to develop sequence- and structure-selective ligands. These foldamers do not target double-stranded DNA, single strands or i-motifs. They selectively target parallel G4s, forming 1:1 and 2:1 complex with sub-μM *K*_D_ values. The crystal structure reveals that two quinoline units in the foldamer helix interact with an exposed G-quartet by π-stacking. The ‘bulkiness’ of the foldamer helix makes it selective for parallel G4 topology with unobstructed 3′ and 5′ G-quartets. The helix clashes with nucleobases surrounding the G-quartet, making it sensitive to loops and flanking nucleotides. We also found that thymines in the loops favor the interaction of foldamers with some specific sequences, offering further opportunities to fine-tune the selectivity.

We believe that through rational design, the structure of the foldamer helix can be adjusted to adapt itself to the terminal G-quartet but also to the surrounding nucleobases. For example, we showed that adding flexibility to the foldamer by incorporating pyridine-based monomers did increase the affinity while preserving G4 specificity. Another intriguing starting point is the particularly high affinity of the QQPQ foldamer to the 5YEY antiparallel variant of the telomeric sequence, which calls for more in-depth structural studies to understand how foldamers could target nonparallel G4s.

As foldamers are made by solid-phase synthesis, other variants can be easily conceived and synthesized. We showed that combinations of four monomer ensured high affinity and some selectivity. Some obvious next steps would be to conceive foldamer compounds with other subunits to modulate the stacking (first two subunits), the flexibility (third subunit), and to explore a variety of sidechains. Overall, our study provides fundamental guidance to those looking to use foldamers as a highly promising ligand scaffold that could be molded to selectively target a G4 target of interest.

## Supplementary Material

gkaf1365_Supplemental_File

## Data Availability

The data underlying this article are available in Zenodo at https://zenodo.org/records/17251445.
