# Supplementary material for: Helical aromatic oligoamide foldamers as selective G-quadruplex ligands
Source: Nucleic Acids Res. 2025 Dec 31;53(22):gkaf1365. doi: 10.1093/nar/gkaf1365 (PMC12754783; doi:10.1093/nar/gkaf1365)
Supplement: gkaf1365_Supplemental_File [file gkaf1365_supplemental_file.pdf]

Helical aromatic oligoamide foldamers as selective G-quadruplex ligands

Alexander König,[1] Vincent Laffilé,[2] Stéphane Thore,[1] Cameron D. Mackereth,[1] Liliya Yatsunyk,[3] Yann Ferrand,[2] Eric Largy,[1*] Valérie Gabelica[1,4]*

[1] Univ. Bordeaux, CNRS, INSERM, ARNA, UMR 5320, U1212, F-33600 Bordeaux, France (eric.largy@u-bordeaux.fr)

[2] Univ. Bordeaux, CNRS, IPB, CBMN, UMR 5248, IECB, F-33600 Bordeaux, France

[3] Department of Chemistry and Biochemistry, Swarthmore College, Swarthmore, PA 19081, United States

[4] School of Pharmaceutical Sciences, University of Geneva, Geneva, Switzerland (valerie.gabelica@unige.ch)

Supporting Information

Table of Contents

Synthesis and characterization of the foldamers 4
Materials and methods 4
Nuclear Magnetic Resonance 4
High Performance Liquid Chromatography 4
Mass spectrometry analyses 4
Methods for chemical synthesis 4
General method for oligomer synthesis 4
Synthesis of Q3,Q4,Q5 and Q8 6
Synthesis of QPQ 7
Synthesis of QQPQ 8
Synthesis of QPPQ 9
Characterization of Q3 10
Characterization of Q4 11
Characterization of Q5 12
Characterization of Q8 13
Characterization of QPQ 14
Characterization of QQPQ 15
Characterization of QPPQ 16
Desalting procedure 17
Circular dichroism 18
CD/UV-melting curves of mutated G-quadruplex sequences 18
CD spectra of multi-stranded G-quadruplexes in NH_4^+ and K^+ 22
Native ESI-MS 23
Methods 23
Parameters of the ESI-IMS-QTOF instrument 23
Generating CCS distributions from ATD distributions 24
Data processing of native ESI-MS titrations 26
Ligand screening 28
Comprehensive list of DNA/Ligand species concentrations and their K_D values 28
Mass spectra 31
ESI-MS titrations 67
K_D values and response factor estimates 67
Mass spectra 69
T24 induces CD on Q_n-type foldamers 110
Foldamer-induced disruption of G-quadruplex 112
X-ray crystallography 113

1D and 2D NMR spectra 117
CD kinetics of QQPQ-induced topology interconversion of 5YEY 130
Quantum chemical and molecular dynamics calculations 131
Methods 131
Torsion scan 131
Conformer search 131
Force field modifications for the foldamer 131
Complex preparation 131
MD preparation 132
Molecular dynamics 132
Data analysis 132
Interplanar angle calculations 133
PCA and cluster centroid determination 133
Results 134
Torsion scan 134
QQPQ/222T 137
Structures mid-production 137
RMSD 138
Tetrad stability 140
Principal component analysis 142
PCA clusters 146
Side-chain H-bonding 154
Stacking distances 159
QQPQ rotation 162
QQPQ stacking 163
Thymine binding 166
QQPQ/5YEY 167
RMSD 167
QQPQ/5YEY T18 interactions 168
References 169

Synthesis and characterization of the foldamers

Materials and methods

Nuclear Magnetic Resonance

1D NMR spectra of oligomers were recorded on a Bruker Avance NEO NMR spectrometer (Bruker BioSpin) operating at 700,15 MHz for ^1H observation, equipped with a 5mm TXI probe with a gradient. All NMR experiments were performed at 273 K. Chemical shift values are given in ppm with reference to residual signals of solvent DMSO-d_6 (δ=2.50). All coupling constants (J) are given in Hertz and ^1H NMR splitting patterns with observed first order coupling are designated as singlet (s), broad singlet (brs), doublet (d), triplet (t) or multiplet (m).

High Performance Liquid Chromatography

HPLC analyses and purification were performed on a reverse phase C8 column on Jasco Extrema analytical and preparative systems. Mobile phases were composed of milli-Q water + 0.1% TFA (solvent A) and Acetonitrile + 0.1% TFA (solvent B). Analyses were done using the following gradient: 0 min: 100% A, 0% B – 2 min: 100% A, 0% B – 12 min: 0% A, 100% B – 15 min: 0% A, 100% B. Purifications were performed using the gradient 0 min: 100% A, 0% B – 2 min: 100% A, 0% B – 32 min: 0% A, 100% B – 35 min: 0% A, 100% B.

Mass spectrometry analyses

MS characterizations were performed on an Agilent Technologies 6230 TOF LC/MS spectrometer. The instrument is equipped with an ESI source and experiment were recorded in positive mode. The spray voltage was maintained at 3500 V and capillary temperature set at 300 °C. Samples were introduced by injection through a 20 µL sample loop into a 600 µL.min^{-1} flow of acetonitrile from the LC pump.

Methods for chemical synthesis

Commercial reagents were purchased from Sigma-Aldrich, Alfa-Aesar or TCI and used without further purification. Low-loading ProTide resin was purchased from CEM. Chloroform ($CHCl_3$), Triethylamine (TEA) and *N*,*N*-diisopropylethylamine (DIEA) were distilled over calcium hydride prior to use. Dry organic solvents: Tetrahydrofuran (THF) and Dichloromethane (DCM), used for solution and solid phase synthesis, were dispensed from a solvent purification system that passes solvents through packed column of dry neutral alumina. Milli-Q water was delivered from a PureLab Prima 7/15/20 system.

General method for oligomer synthesis

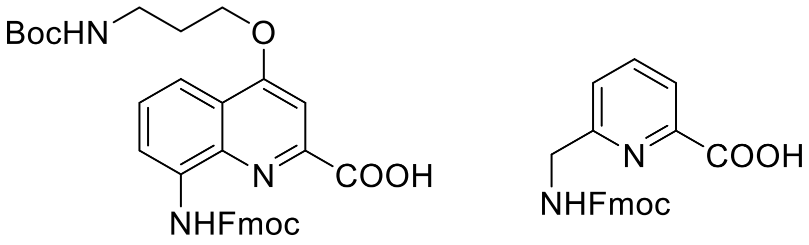

Q^{orn} monomer **P monomer**

Figure S1 Structures of monomers ***Q^{Orn}*** *and* ***P****. These monomers were synthetized as Fmoc-N-protected and acid free form according to the reported procedure.*[1]

Resin grafting: On *LL* ProTide resin, the first **Q**^{Orn} monomer (3 equiv.) was grafted using CsI (5 equiv.) and DIEA (6 equiv.) in dry DMF. Reaction mixture was vigorously shaking overnight. After reaction, the resin was filtered and washed three times with DMF and dichloromethane.

Fmoc deprotection: Grafted resin was washed twice with DMF, suspended in a 20% piperidine in DMF solution (4mL) and slowly stirred for 3 minutes. Resin was then filtered, washed twice with DMF and suspended again in a 20% piperidine in DMF solution and stirred for 7 minutes. The resin was then filtered and washed three times with DMF and dry THF.

***In-situ* coupling procedure**: *For coupling on the aromatic amines of the **Q**^{Orn} monomer.* Resin was suspended in dry THF and collidine (9 equiv.) was added. A solution of monomer (3 equiv.), PPh_3 (8 equiv.) and trichloroacetonitrile (TCAN, 9 equiv.) in dry $CHCl_3$ was added on the resin. The reaction was assisted by micro-waves (25 W, 50°C) for 15 minutes and repeated once. After reaction, the resin was filtered and washed with dry THF and DMF.

HBTU coupling: *For coupling on the aliphatic amines of the **P** monomer*. Resin was suspended in dry DMF. Monomer (3 equiv.) and HBTU (2.9 equiv.) as powder were added followed by DIEA (6 equiv.). The reaction was assisted by micro-waves (50 W, 50°C) for 10 minutes and repeated once. After reaction, the resin was filtered and washed with DMF.

Resin cleavage: Resin was washed three times with DMF and dichloromethane and was suspended in a solution of TFA/TIPS/H_2O 95:2.5:2.5 (*v/v/v*). The mixture was vigorously stirred for 4 hours. The resin was filtered, and the filtrate was evaporated under reduced pressure. The residual solid was suspended in Et_2O and centrifugated at 4°C for 5 minutes. Et_2O was removed and the yellow solid was dried under vacuum and then freeze-dried in water.

Preparative HPLC purifications: Crude compounds were purified using solvents A and B. The following gradient was used: 0 min: 100% A, 0% B – 2 min: 100% A, 0% B – 22 min: 0% A, 100% B – 27 min: 0% A, 100% B. Collected fractions were analyzed by analytic HPLC and the relevant ones were combined and freeze-dried twice to remove the excess of TFA.

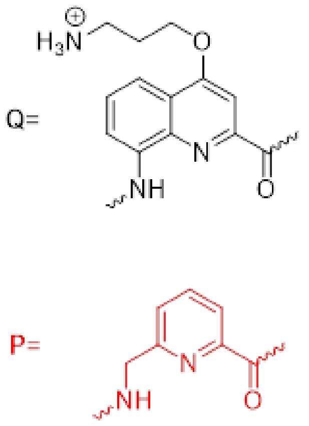

1: Ac-QQQ-OH
2: Ac-QQQQ-OH
3: Ac-QQQQQ-OH
4: Ac-QQQQQQQQ-OH

5: Ac-QPQ-OH
6: Ac-QQPQ-OH
7: Ac-QPPQ-OH

Figure S2. Synthesized oligomers

Synthesis of Q3,Q4,Q5 and Q8

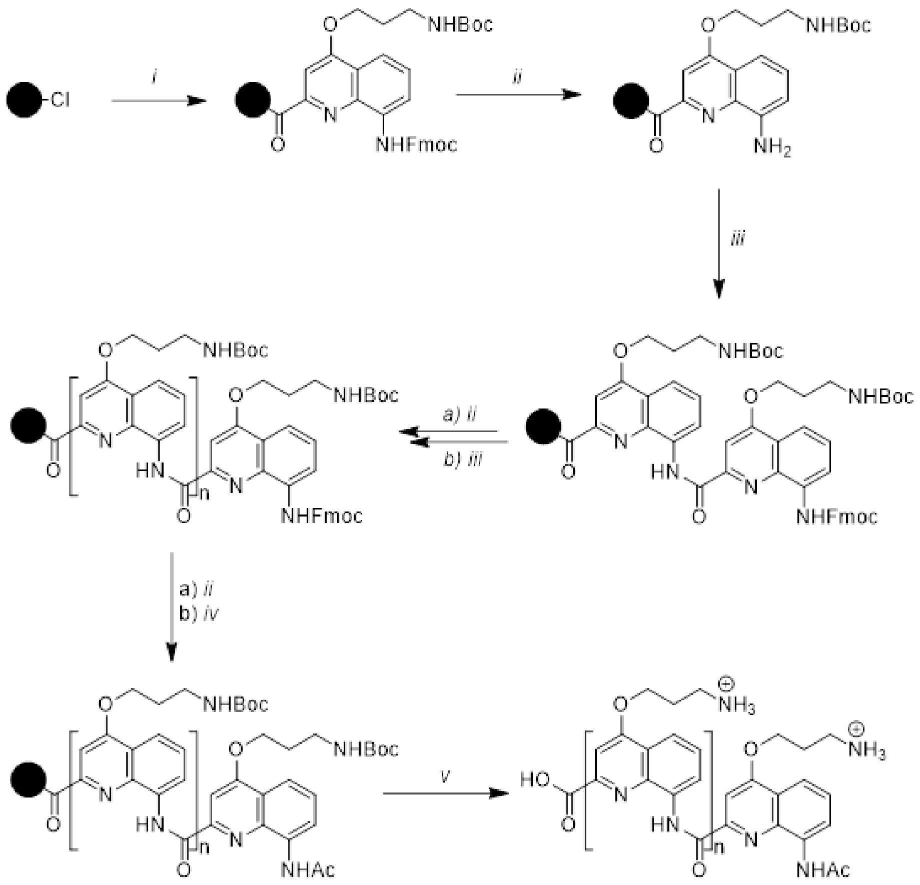

1: n=2 ; **2**: n=3 ; **3**: n=4 ; **4**: n=7

Figure S3. Solid phase synthesis of oligomers ***1*** *to* ***4****. i)* ***Q***^{Orn} *monomer, CsI, DIEA, dry DMF. ii) 2 times, piperidine/DMF 2:8 (v/v), iii) 2 times,* ***Q***^{Orn} *monomer,* PPh_3*, TCAN, collidine, THF/*$CHCl_3$*. These two last steps are repeated until the desired length is obtained. iv) 2 times, acetyl chloride, DIEA, THF. v) TFA/TIPS/*H_2O *95:2.5:2.5 (v/v/v)*

Synthesis of QPQ

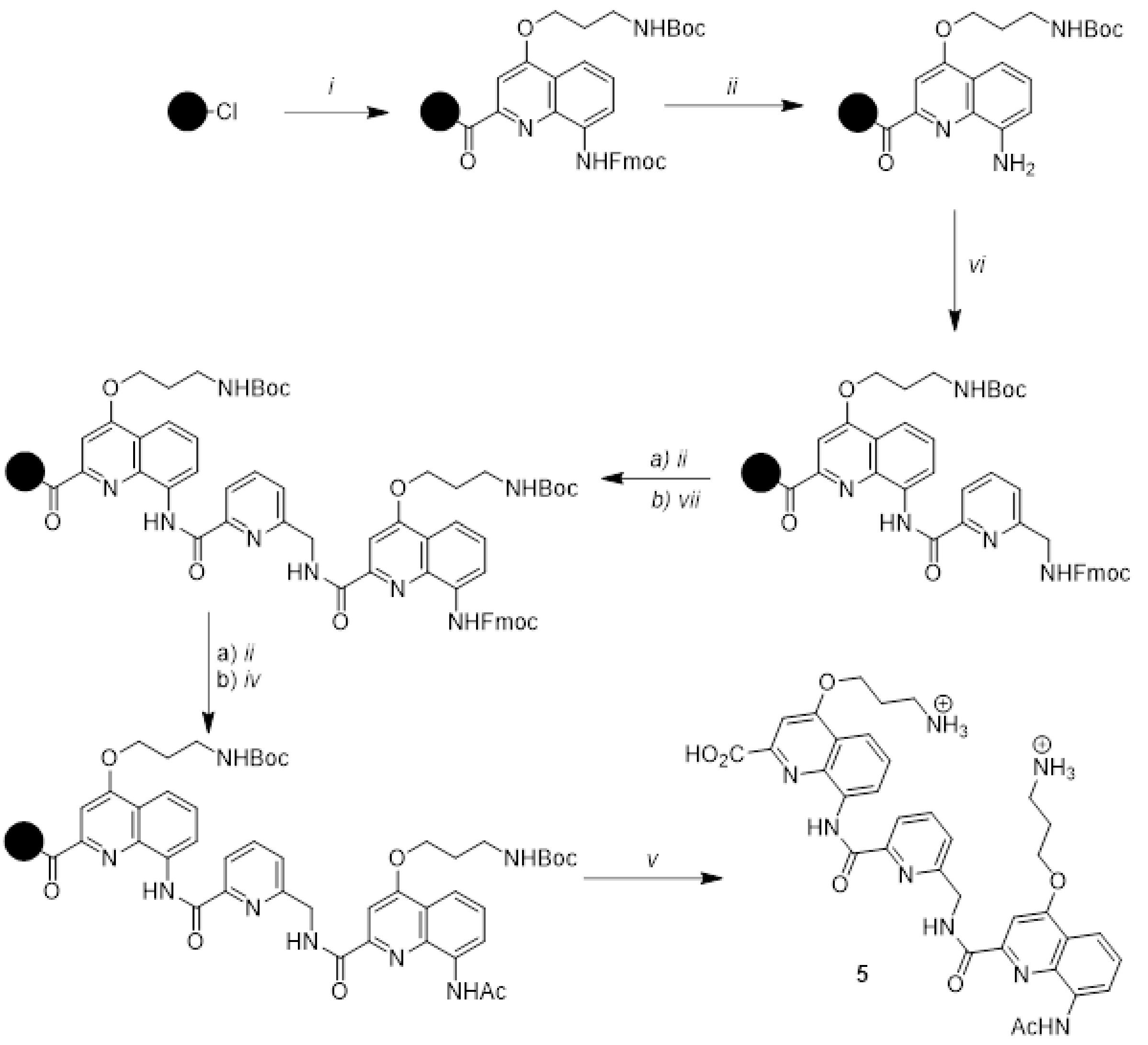

*Figure S4. Solid phase synthesis of oligomer **5**. vi) 2 times, **P** monomer, PPh_3, TCAN, collidine, THF/$CHCl_3$. vii) 2 times, **Q^{Orn}** monomer, HBTU, DIEA, dry DMF.*

Synthesis of QQPQ

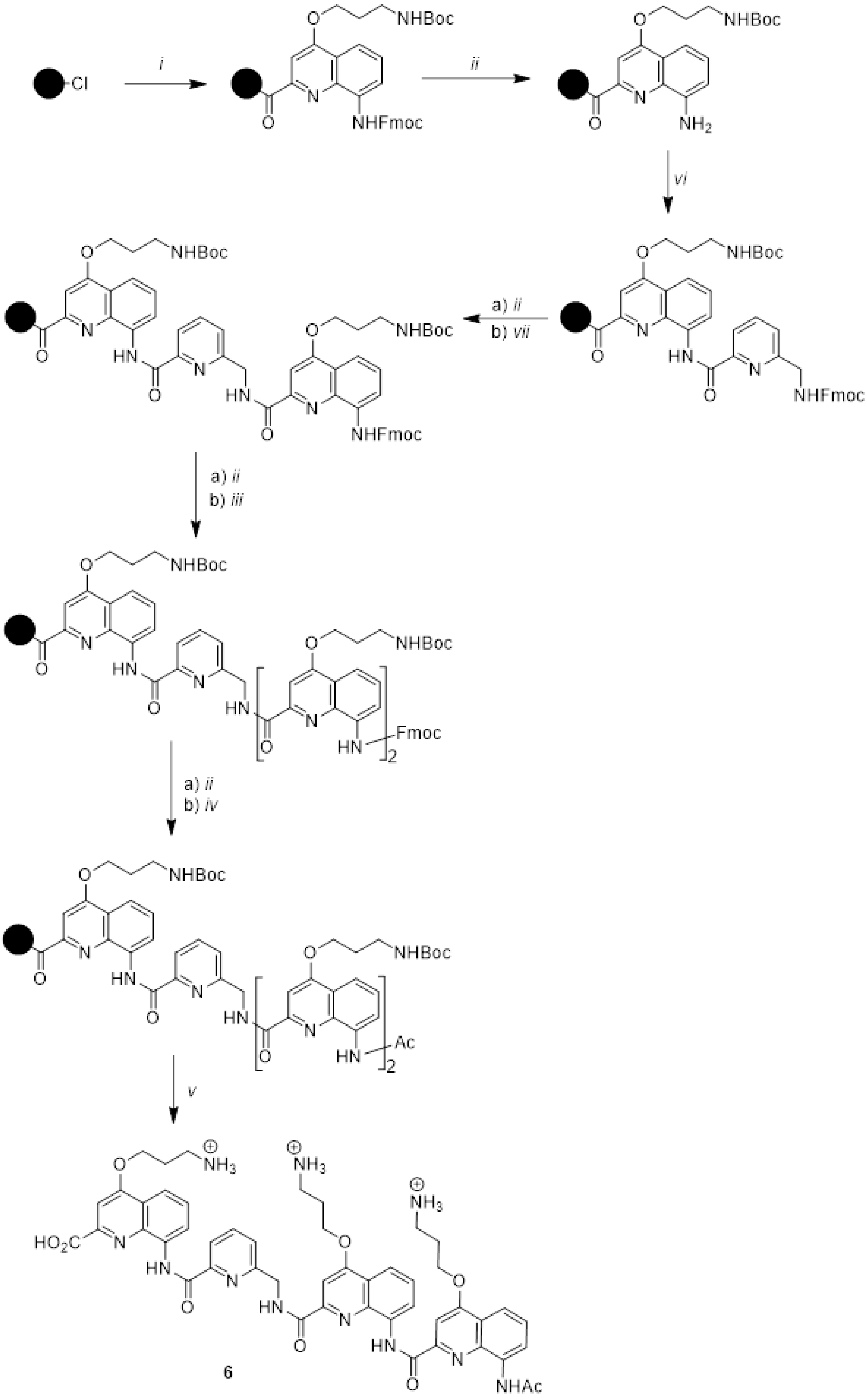

6

*Figure S5. Solid phase synthesis of oligomer **6.***

Synthesis of QPPQ

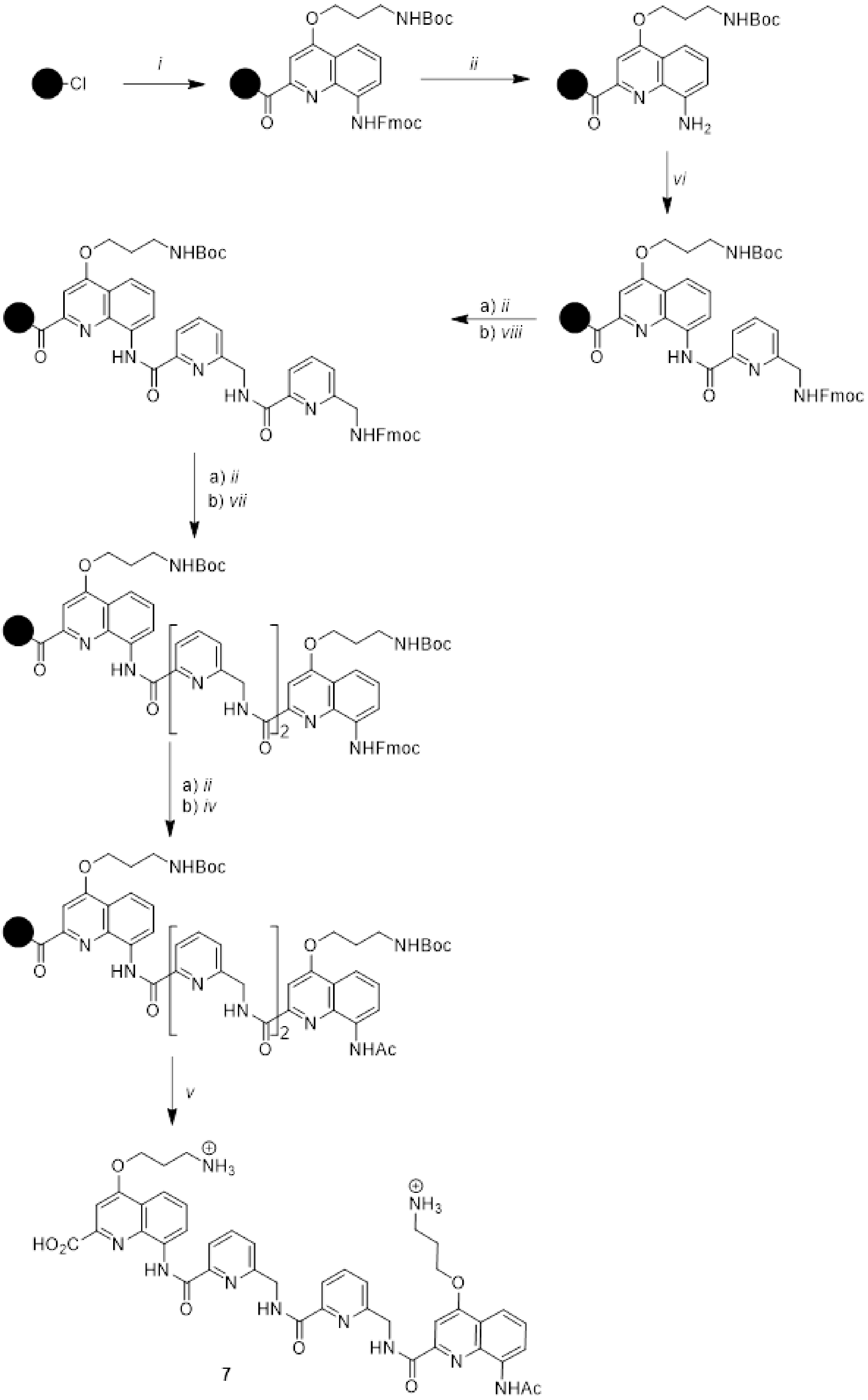

*Figure S6. Solid phase synthesis of oligomer **7**. viii) 2 times, **P** monomer, HBTU, DIEA, dry DMF.*

Characterization of Q3

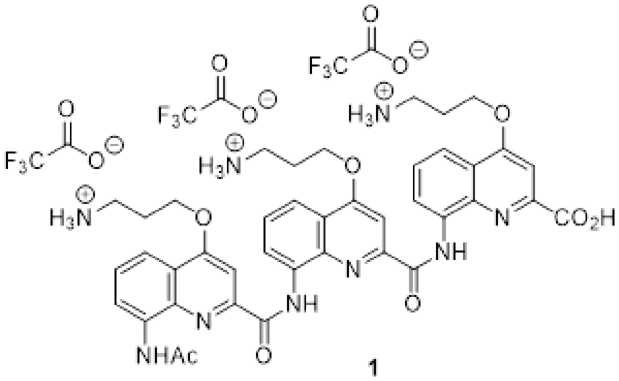

1

Trimer **1** was synthetized as a trifluoroacetate salt on 8 µmol scale following the *in-situ* activation procedure. The target compound was obtained as a light-yellow solid after purification by preparative HPLC (4 mg, 63% yield). ^{1}H NMR (700 MHz, DMSO-d6). δ 12.45 (brs, 1H), 12.27 (s, 1H), 12.16 (s, 1H), 9.23 (s, 1H), 8.91 (d, 1H, $^3J_{H\text{-}H}$=7.5 Hz), 8.85 (d, 1H, $^3J_{H\text{-}H}$=7.4 Hz), 8.03 (d, 1H, $^3J_{H\text{-}H}$=8.2 Hz), 7.76-7.87 (m, 9H), 7.71-7.74 (m, 2H), 7.66-7.70 (m, 2H), 7.28 (t, 1H, $^3J_{H\text{-}H}$=8.0 Hz), 6.58 (s, 1H), 4.57 (t, 2H, $^3J_{H\text{-}H}$=5.8 Hz), 4.54 (t, 2H, $^3J_{H\text{-}H}$=5.5 Hz), 4.12 (t, 2H, $^3J_{H\text{-}H}$=5.5 Hz), 3.04-3.16 (m, 6H), 2.21-2.27 (m, 4H), 2.15-2.20 (m, 2H), 1.68 (s, 2H). HRMS (ESI$^+$) m/z [M+H]$^+$ 790.3303 (calc. 790.3307 for $C_{41}H_{44}O_8N_9^+$).

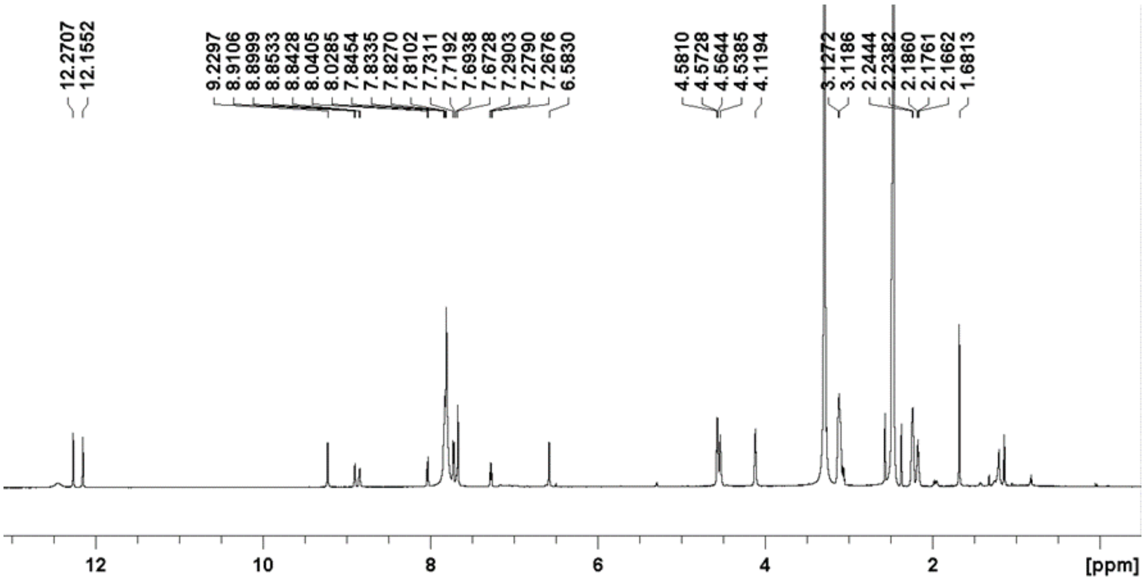

*Figure S7. ^{1}H NMR spectrum of oligomer **1**, measured in DMSO-d6 at 25°C.*

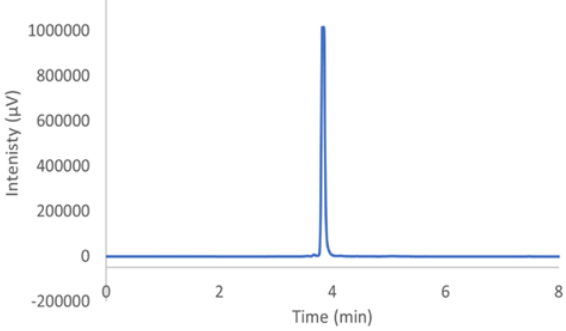

*Figure S8. HPLC trace of oligomer **1**. Analysis was performed using solvents A and B with the gradient: 0 min: 100% A, 0% B – 2 min: 100% A, 0% B – 12 min: 0% A, 100% B – 15min: 0% A, 100% B.*

Characterization of Q4

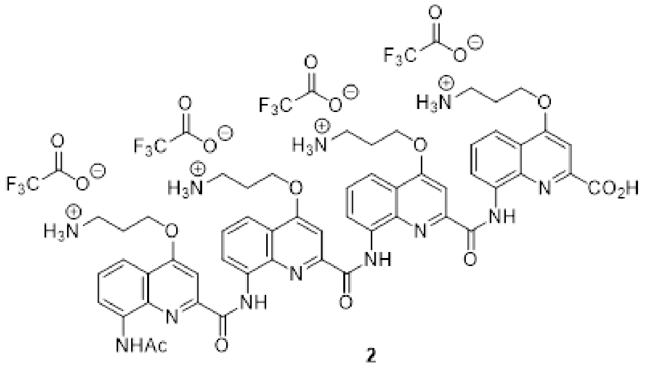

2

Tetramer **2** was synthetized as a trifluoroacetate salt on 16 μmol scale following the *in-situ* activation procedure. The target compound was obtained as a light-yellow solid after purification by preparative HPLC (16 mg, 67% yield). ^{1}H NMR (700 MHz, DMSO-d6). δ 12.53 (brs, 1H), 12.20 (s, 1H), 11.77 (s, 1H), 11.69 (s, 1H), 9.08 (s, 1H), 8.97 (d, 1H, 3JH-H=7.3 Hz), 8.43 (brs, 1H), 7.81-7.99 (m, 12H), 7.75-7.80 (m, 2H), 7.74 (s, 1H), 7.44 (brs, 1H), 7.37 (t, 1H, 3JH-H=7.8 Hz), 7.26 (s, 1H), 6.75 (s, 1H), 6.66 (brs, 1H), 4.54-4.59 (m, 4H), 4.34 (brs, 1H), 4.23 (brs, 1H), 4.16 (brs, 2H), 3.07-3.22 (m, 6H), 2.20-2.46 (m, 8H), 1.69 (s, 3H). HRMS (ESI$^+$) m/z 1033,4316 [M+H]$^+$ (calc. 1033,4315 for $C_{54}H_{57}O_{10}N_{12}^+$).

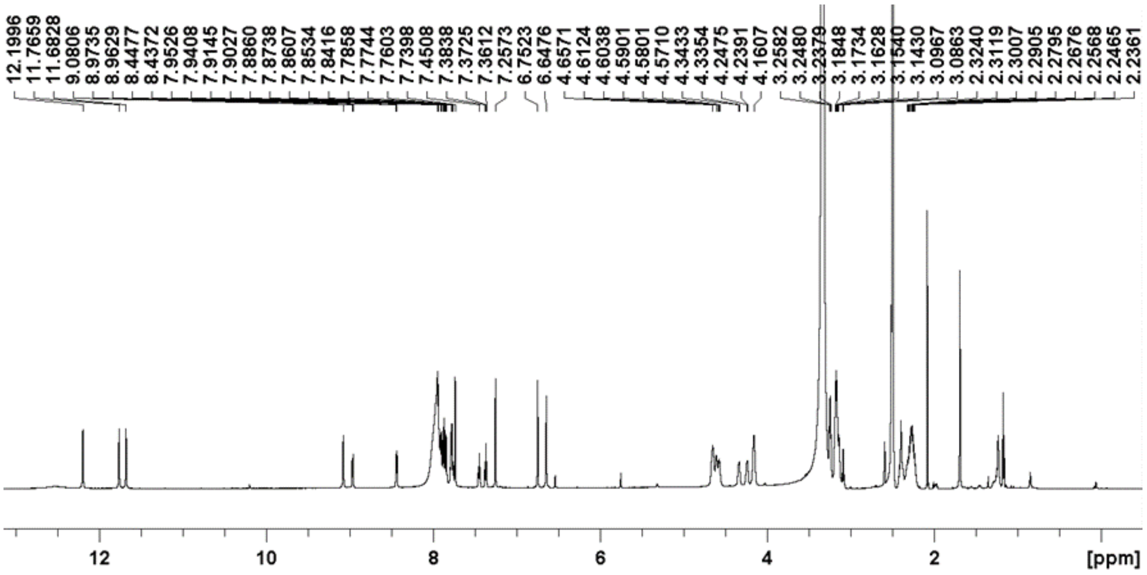

*Figure S9. ^{1}H NMR spectrum of tetramer **2**, measured in DMSO-d6 at 25°C on 700MHz Bruker Avance NEO spectrometer (TXI probe (^{1}H, ^{13}C, ^{15}N, ^{2}H), 5mm, z-gradients).*

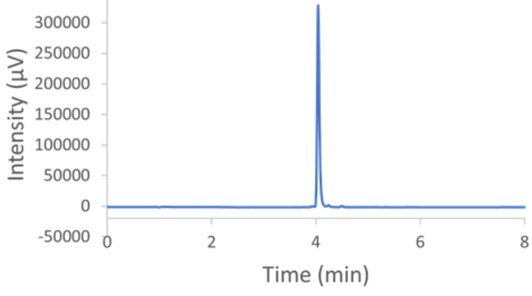

*Figure S10. HPLC trace of oligomer **2**. Analysis was performed using solvents A and B with the gradient: 0 min: 100% A, 0% B – 2 min: 100% A, 0% B – 12 min: 0% A, 100% B – 15 min: 0% A, 100% B.*

Characterization of Q5

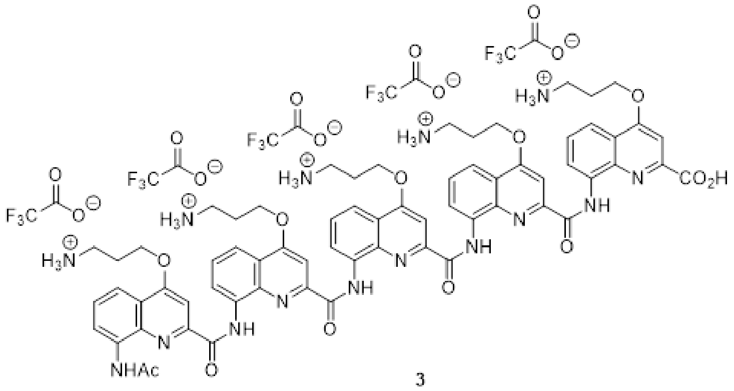

3

Pentamer **3** was synthetized as a trifluoroacetate salt on 10 µmol scale following the *in-situ* activation procedure. The target compound was obtained as a light-yellow solid after purification by preparative HPLC (7.5 mg, 59% yield). ^{1}H NMR (700 MHz, DMSO-d6). δ 12.21 (brs, 1H), 11.74 (s, 1H), 11.68 (s, 1H), 11.64 (s, 1H), 11.52 (s, 1H), 8.87 (s, 1H), 8.50 (d, 2H, $^3J_{H\text{-}H}$), 7.4 Hz), 7.88-8.06 (m, 17H), 7.78-7.86 (m, 3H), 7.75 (d, 1H, $^3J_{H\text{-}H}$=8.2 Hz), 7.67 (d, 1H, $^3J_{H\text{-}H}$=7.3 Hz), 7.51 (t, 1H, $^3J_{H\text{-}H}$=7.8 Hz), 7.38 (t, 1H, $^3J_{H\text{-}H}$=7.8 Hz), 7.33 (t, 1H, $^3J_{H\text{-}H}$=7.8 Hz), 7.30 (s, 1H), 7.20 (s, 1H), 6.79 (s, 1H), 6.67 (s, 1H), 6.52 (s, 1H), 4.67-4.73 (m, 2H), 3.08-3.28 (m, 13H), 2.17-2.46 (m, 13H), 1.36 (s, 2H). HRMS (ESI$^+$) m/z [M+H]$^+$ 1276.5369 (calc. 1276.5323 for $C_{67}H_{70}O_{12}N_{15}^+$).

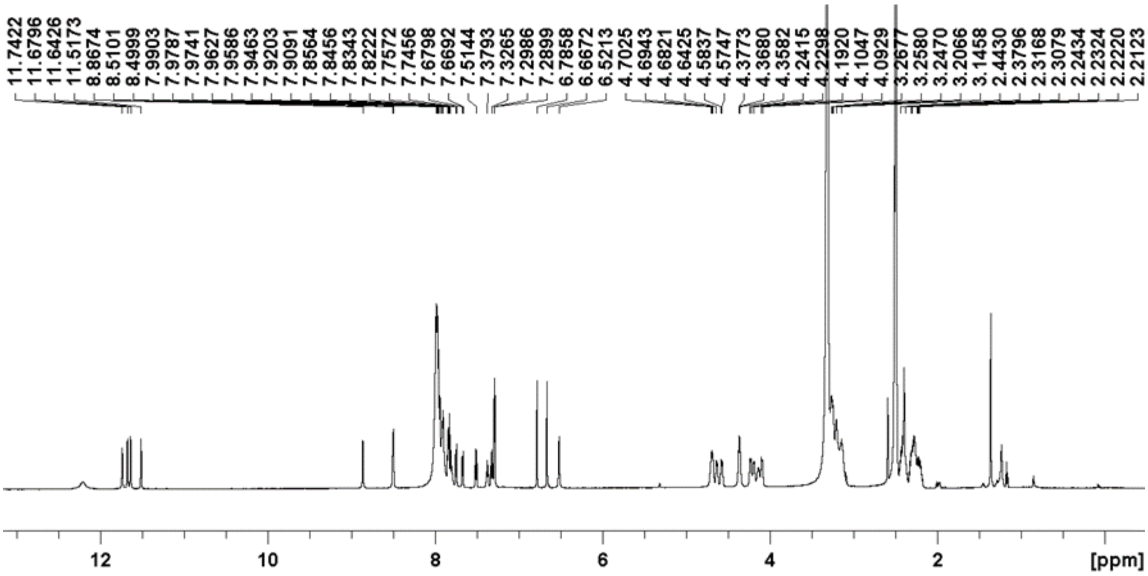

*Figure S11. ^{1}H NMR spectrum of pentamer **3**, measured in DMSO-d6 at 25°C on 700MHz Bruker Avance NEO spectrometer (TXI probe (^{1}H, ^{13}C, ^{15}N, ^{2}H), 5mm, z-gradients).*

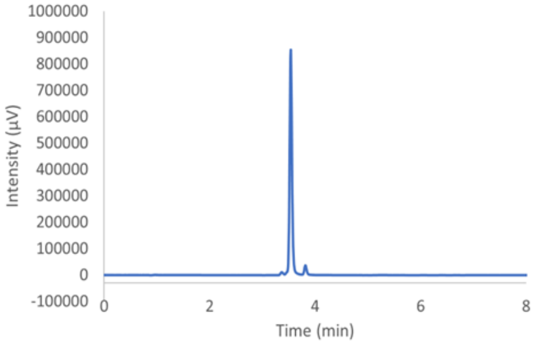

*Figure S12. HPLC trace of oligomer **3**. Analysis was performed using solvents A and B with the gradient: 0 min: 100% A, 0% B – 2 min: 100% A, 0% B – 12 min: 0% A, 100% B – 15 min: 0% A, 100% B.*

Characterization of Q8

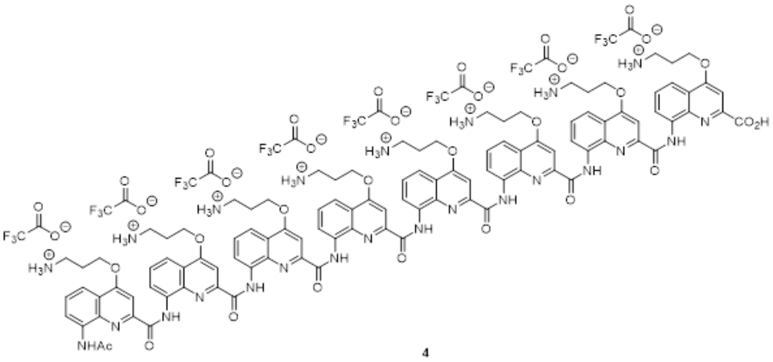

4

Octamer **4** was synthesized as a trifluoroacetate salt on 10 µmol scale following the *in-situ* activation procedure. The target compound was obtained as a light-yellow solid after purification by preparative HPLC (11.5 mg, 57% yield). ^{1}H NMR (700 MHz, DMSO-d6). δ 11.93 (brs, 1H), 11.28 (s, 1H), 11.25 (s, 1H), 11.14 (s, 1H), 11.04 (s, 1H), 11.03 (s, 1H), 10.94 (s, 1H), 10.88 (s, 1H), 8.91 (brs, 1H), 8.44 (s, 1H), 8.07-8.27 (m, 11H), 7.83-8.04 (m, 12H), 7.74-7.81 (m, 3H), 7.67 (d, 2H, $^3J_{H-H}$=7.9 Hz), 7.44-7.51 (m, 3H), 7.31-7.43 (m, 4H), 7.17 (t, 1H, $^3J_{H-H}$=8.3 Hz), 7.12 (t, 1H, $^3J_{H-H}$=8.1 Hz), 7.02 (d, 1H, $^3J_{H-H}$=7.3Hz), 6.96 (s, 1H), 6.85 (s, 1H), 6.58 (s, 1H), 6.53 (s, 1H), 6.36 (s, 1H), 6.30 (s, 1H), 6.17 (s, 1H), 5.91 (s, 1H), 4.36-4.51 (m, 5H), 4.12-4.34 (m, 9H), 3.97-4.07 (m, 3H), 3.85-3.90 (m, 1H), 2.96-3.19 (m, 16H), 1.95-2.35 (m, 15H), 1.24 (s, 3H). HRMS (ESI$^+$) m/z [M+H]$^+$ 2005.8351 (calc. 2005.8346 for $C_{106}H_{109}O_{18}N_{24}^+$).

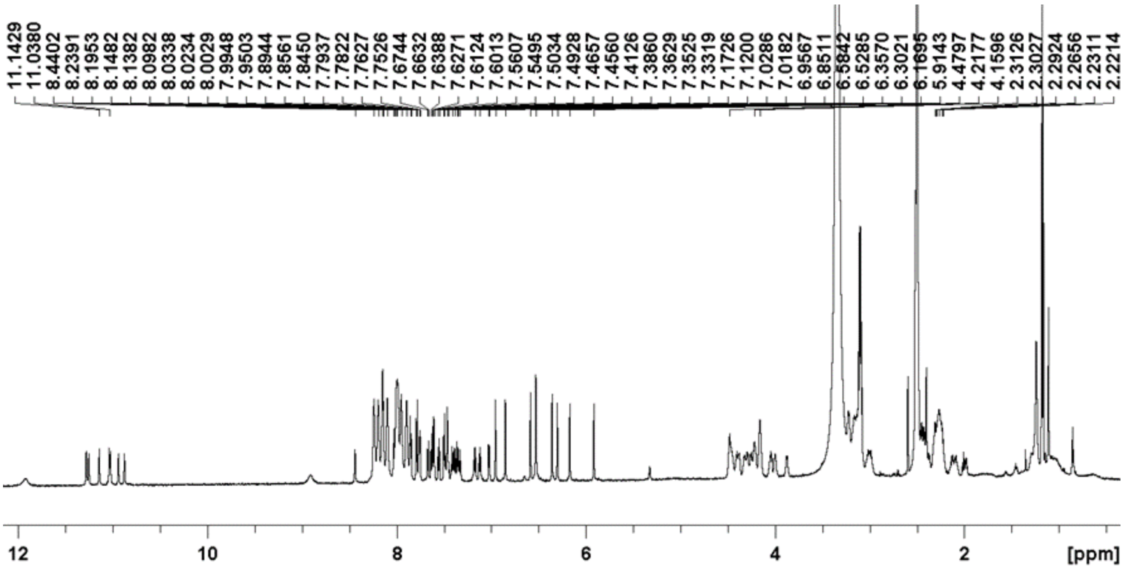

*Figure S13. ^{1}H NMR spectrum of octamer **4**, measured in DMSO-d6 at 25°C on 700MHz Bruker Avance NEO spectrometer (TXI probe (^{1}H, ^{13}C, ^{15}N, ^{2}H), 5mm, z-gradients).*

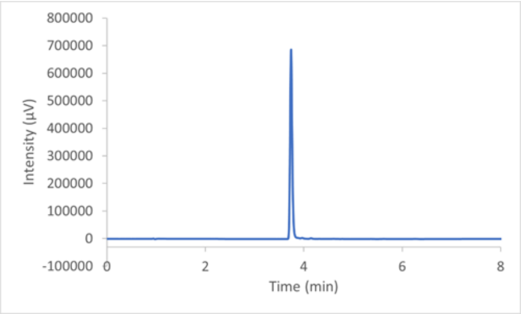

*Figure S14. HPLC trace of oligomer **4**. Analysis was performed using solvents A and B with the gradient: 0 min: 100% A, 0% B – 2 min: 100% A, 0% B – 12 min: 0% A, 100% B – 15 min: 0% A, 100% B.*

Characterization of QPQ

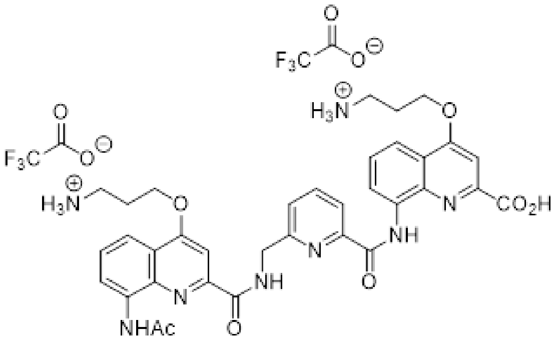

5

Trimer **5** was synthetized as a trifluoroacetate salt on 16 µmol scale following *in-situ* activation procedure for coupling on $\mathbf{Q^{Orn}}$ monomer and HBTU coupling procedure for coupling on **P** monomer. The target compound was obtained as a white solid after purification by preparative HPLC (7mg, 48% yield). ^{1}H NMR (700 MHz, DMSO-d6). δ 13.61 (brs, 1H), 12.60 (s, 1H), 10.35 (t, 1H, 3JH-H=6.2 Hz), 10.25 (s, 1H), 8.91 (d, 1H, 3JH-H=7.2 Hz), 8.74 (d, 1H, 3JH-H=7.7 Hz), 8.08-8.17 (m, 2H), 7.69-7.97 (m, 8H), 7.60-7.67 (m, 3H), 6.55 (s, 1H), 4.99 (d, 2H, 3JH-H=6.1 Hz), 4.43-4.50 (m, 4H), 3.06-3.15 (m, 5H), 2.29 (s, 2H), 2.16-2.25 (m, 4H), 1.24 (s, 3H). HRMS (ESI$^+$) m/z [M+H]$^+$ 681.2777 (calc. 681.2780 for $C_{35}H_{37}O_7N_8^+$).

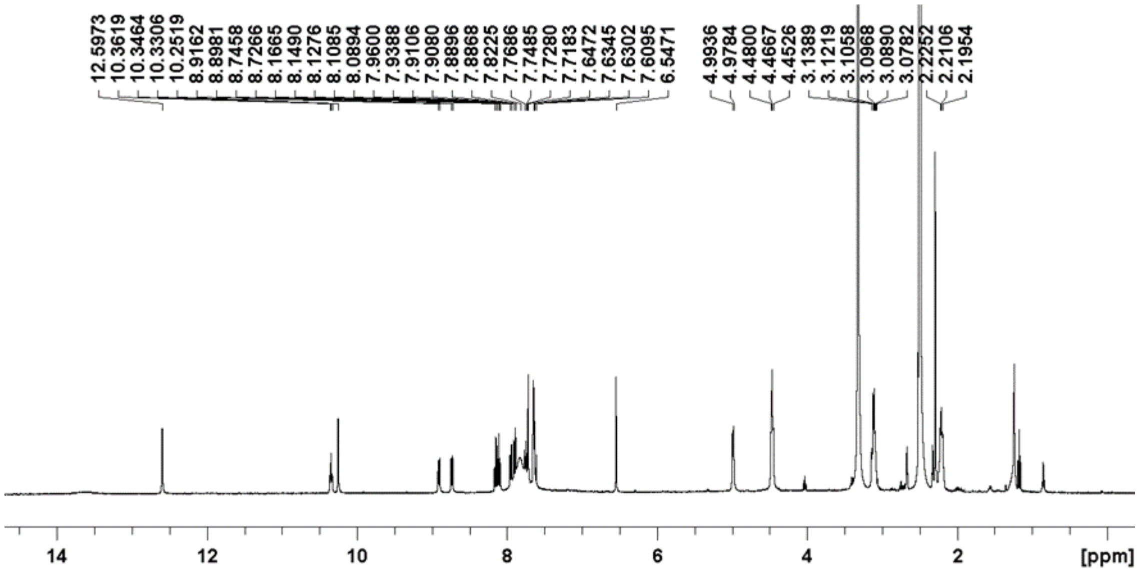

*Figure S15. ^{1}H NMR spectrum of trimer **5**, measured in DMSO-d6 at 25°C on 700MHz Bruker Avance NEO spectrometer (TXI probe (^{1}H, ^{13}C, ^{15}N, ^{2}H), 5mm, z-gradients).*

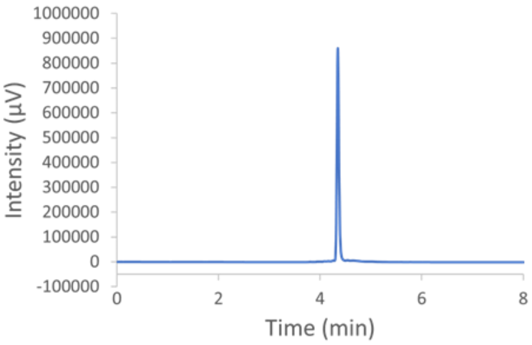

*Figure S16. HPLC trace of oligomer **5**. Analysis was performed using solvents A and B with the gradient: 0 min: 100% A, 0% B – 2 min: 100% A, 0% B – 12 min: 0% A, 100% B – 15 min: 0% A, 100% B.*

Characterization of QQPQ

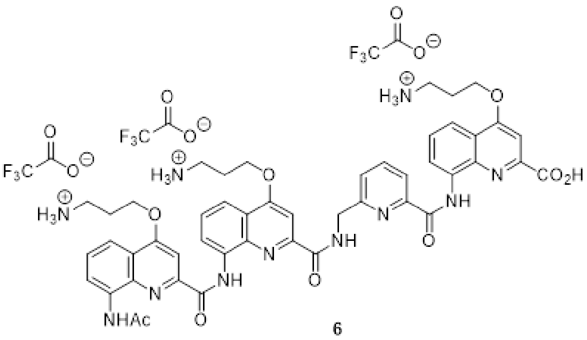

6

Tetramer **6** was synthetized as a trifluoroacetate salt on 16 µmol scale following *in-situ* activation procedure for coupling on **Q**^{Orn} monomer and HBTU coupling procedure for coupling on **P** monomer. The target compound was obtained as a white solid after purification by preparative HPLC (13 mg, 64% yield). ^{1}H NMR (700 MHz, DMSO-d6). δ 11.82 (brs, 1H), 11.56 (s, 1H), 9.52 (brs, 1H), 9.30 (s, 1H), 8.31 (d, 1H, $^3J_{H-H}$=7.4 Hz), 7.77-8.14 (m, 10H), 7.61-7.71 (m, 2H), 7.75 (t, 1H, $^3J_{H-H}$=7.9 Hz), 7.43 (t, 1H, 3JH-H=8.0 Hz), 7.31 (d, 1H, $^3J_{H-H}$=7.4 Hz), 7.11 (s, 1H), 6.74 (s, 1H), 6.57 (s, 1H), 5.72 (s, 1H), 4.78 (brs, 2H), 4.53-4.56 (m, 2H), 4.48-4.52 (m, 2H), 4.18-4.22 (m, 2H), 3.06-3.25 (m, 7H), 2.16-2.38 (m, 5H). HRMS (ESI$^+$) m/z [M+H]$^+$ 924.3048 (calc. 924.3787 for $C_{48}H_{50}O_9N_{11}^+$).

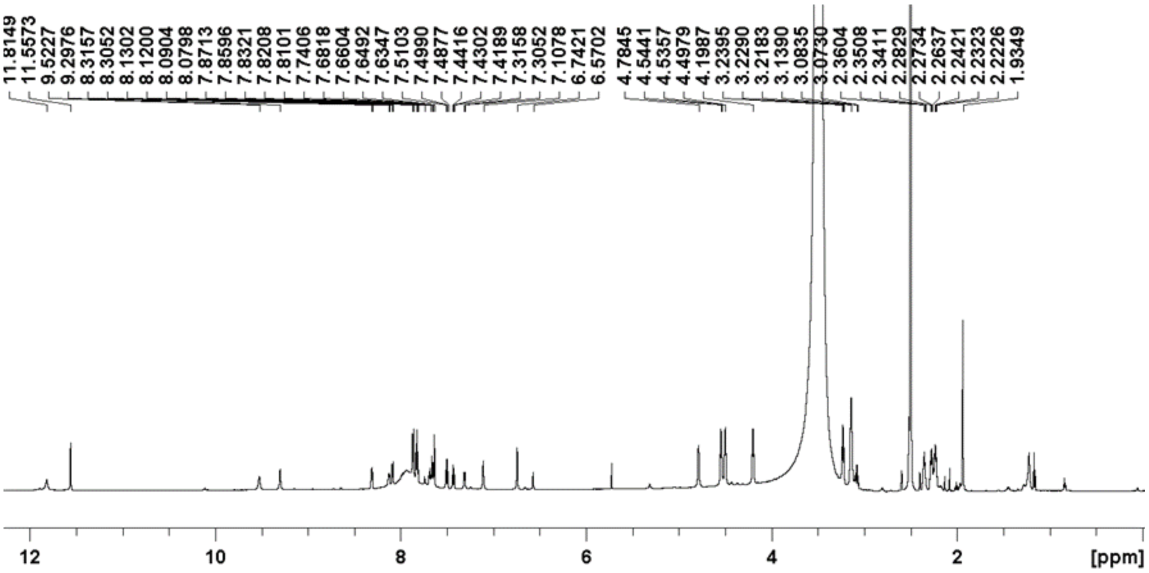

*Figure S17. ^{1}H NMR spectrum of tetramer **6**, measured in DMSO-d6 at 25°C on 700MHz Bruker Avance NEO spectrometer (TXI probe (^{1}H, ^{13}C, ^{15}N, ^{2}H), 5mm, z-gradients).*

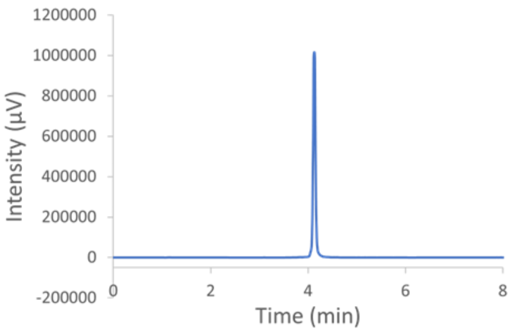

*Figure S18. HPLC trace of oligomer **6**. Analysis was performed using solvents A and B with the gradient: 0 min: 100% A, 0% B – 2 min: 100% A, 0% B – 12 min: 0% A, 100% B – 15 min: 0% A, 100% B.*

Characterization of QPPQ

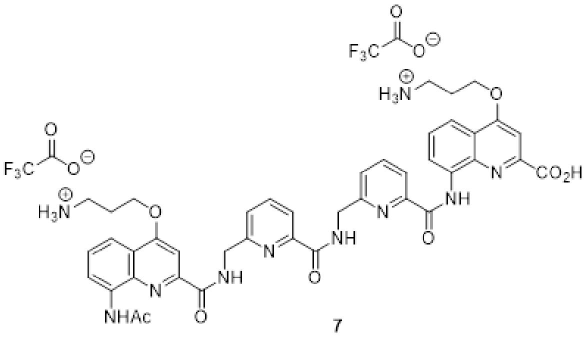

7

Tetramer **7** was synthetized as a trifluoroacetate salt on 16 µmol scale following *in-situ* activation procedure for coupling on **Q**^{Orn} monomer and HBTU coupling procedure for coupling on **P** monomer. The target compound was obtained as a white solid after purification by preparative HPLC (11 mg, 66% yield). ^{1}H NMR (300 MHz, DMSO-d6). δ 12.60 (s, 1H), 10.09 (brs, 1H), 9.61 (brs, 1H), 8.91 (d, 1H, $^{3}J_{H-H}$=7.7 Hz),7.92-8.15 (m, 7H), 7.64-7.82 (m, 9H), 7.48-7.60 (m, 4H), 7.21-7.36 (m, 4H), 6.84 (d, 1H, $^{3}J_{H-H}$=7.2 Hz), 4.87 (d, 2H, $^{3}J_{H-H}$=6.2 Hz), 4.73 (d, 2H, $^{3}J_{H-H}$=6.1 Hz), 4.43-4.52 (m, 2H), 4.34-4.42 (m, 3H), 2.96-3.15 (m, 6H), 1.50-2.69 (m, 8H). HRMS (ESI^{+}) m/z [M+H]$^{+}$ 815.3253 (calc. 815.3260 for $C_{42}H_{43}O_{8}N_{10}^{+}$).

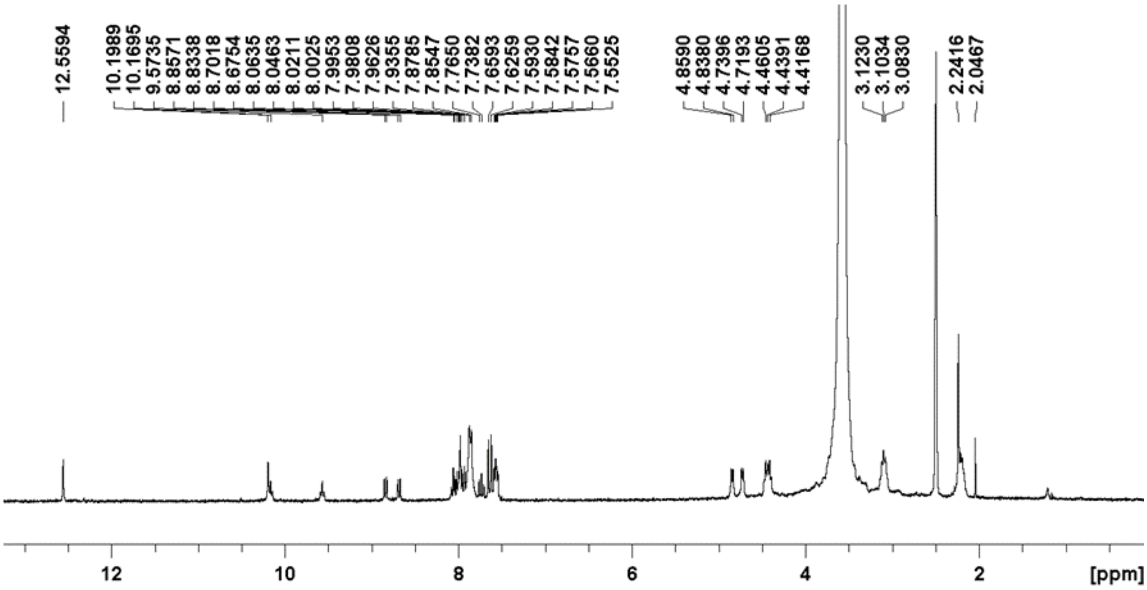

*Figure S19. ^{1}H NMR spectrum of tetramer **7**, measured in DMSO-d6 at 25°C on 300MHz Bruker Avance NEO spectrometer (TXI probe (^{1}H, ^{13}C, ^{15}N, ^{2}H), 5mm, z-gradients).*

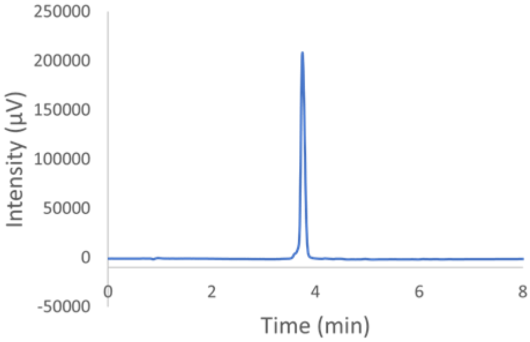

*Figure S20. HPLC trace of oligomer **7**. Analysis was performed using solvents A and B with the gradient: 0 min: 100% A, 0% B – 2 min: 100% A, 0% B – 12 min: 0% A, 100% B – 15 min: 0% A, 100% B.*

Desalting procedure

Traces of Na^+ in the oligonucleotide stock give rise to visible Na^+ adducts in the mass spectrum, causing loss of resolution and S/N ratio. We therefore strip our stock solutions off of Na^+ using centrifugal filters (Amicon Ultracel 3K, Millipore). The filters contain a cellulose matrix that will hold back the DNA macromolecule, while allowing the washing solution to pass through via centrifugation.

Prior to desalting, the oligonucleotides are dissolved in water and annealed at 85°C for 2-3 min. Then, the first segment of the desalting process is exchanging the non-volatile Na^+ ion with volatile NH_4^+. The filter unit is filled with a washing solution of 500 mM Ammonium acetate and placed in the centrifuge for 15 minutes at 15,000 rpm. This process is repeated four times. The second segment is diluting with pure water to gradually flush out the NH_4^+ ions. Some NH_4^+ ions will remain electrostatically bound to the DNA strand, but they will detach during the MS ionization process, given their volatility. This washing process is repeated six times. The third segment is the recovery of the desalted DNA stock solution by placing the filter upside-down in a fresh tube and pushing it out at 1,000 rpm for 3 minutes.

A proof of concept for the desalting method is illustrated in Figure S21.

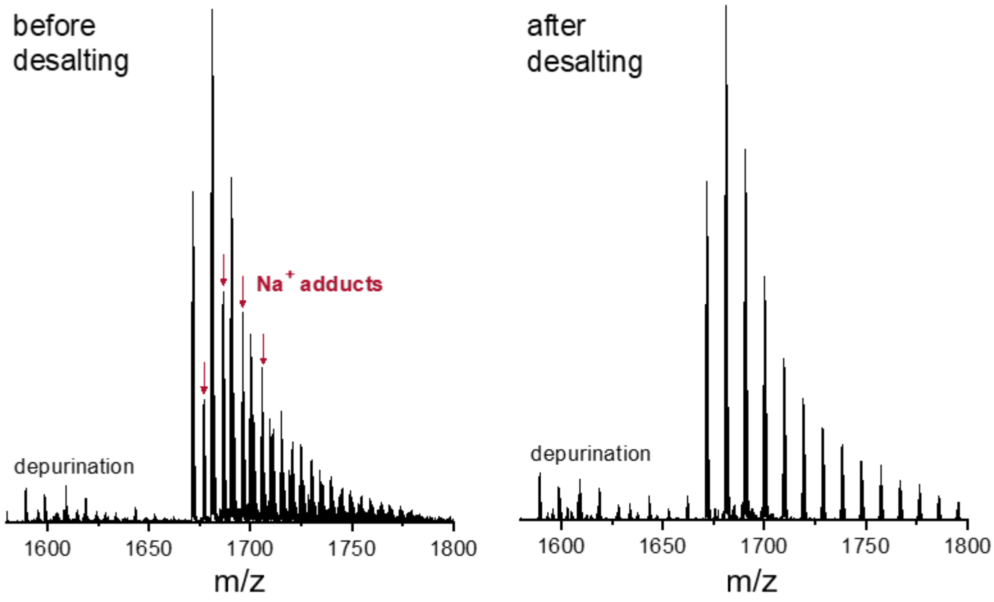

*Figure S21. Mass spectrum of DNA sequence 21G (d**G**$_3$TTA**G**$_3$TTA**G**$_3$TTA**G**$_3$) before (left) and after (right) desalting, zoomed in on the 4- charge state. Sample conditions: 10 µM DNA, 1 mM KCl, 100 mM TMAA in H_2O.*

Circular dichroism

CD/UV-melting curves of mutated G-quadruplex sequences

This section is for the mutant sequences 222T-mA, 222T-mC, 26CEB-mT, 22GT-18T and 24TTG-20T that were introduced into the foldamer screening panel to probe the foldamers' sensitivity to loop-induced G4 topology switches. 22GT-18T and 24TTG-20T are telomeric repeats where the third loop has a TTA to TTT mutation. Removing the adenine disrupts the formation of AGA triads and induces a topology switch.[2] 222T-mA, 222T-mC and 26CEB-mT are mutated in the middle loop. The effect of mutating the middle loop is unknown for these sequences. We also provide spectra for 'bcl2' since we were unsure of its G-quadruplex stability in low K^+ concentration.

For our six undocumented sequences we provide CD spectra and UV melting curves. One dataset in 1 mM KCl, to be orthogonal with our previously established database.[23] Another dataset in 0.5 mM KCl, which is the concentration for the ligand screening. In second approach, this dataset gives insight for how much cutting the K^+ concentration in half affects G-quadruplex stability.

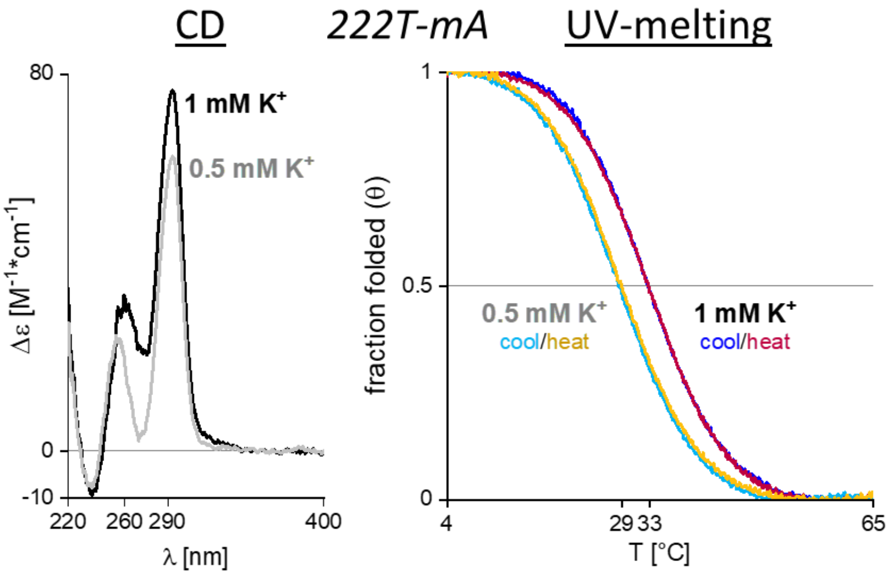

Figure S22. ***Left***: *CD spectra of 222T-mA (dTG_3TTG_3AAG_3TTG_3T) in 1 mM (black) and 0.5 mM KCl (grey).* ***Right***: *UV-melting curves in 1 mM (blue/red) and 0.5 mM (cyan/orange) KCl. Samples contain 10 µM DNA and 100 mM TMAA (pH 6.8).*

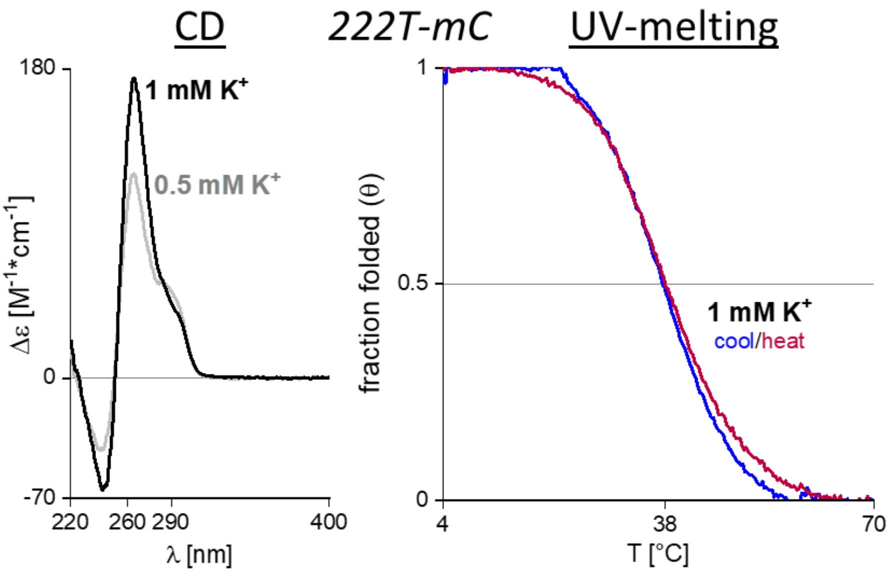

*Figure S23. **Left**: CD spectra of 222T-mC (dT$\mathbf{G_3}$TT$\mathbf{G_3}$CC$\mathbf{G_3}$TT$\mathbf{G_3}$T) in 1 mM (black) and 0.5 mM KCl (grey). **Right**: UV-melting curve in 1 mM (blue/red) KCl. No data could be obtained in 0.5 mM KCl. Samples contain 10 µM DNA and 100 mM TMAA (pH 6.8).*

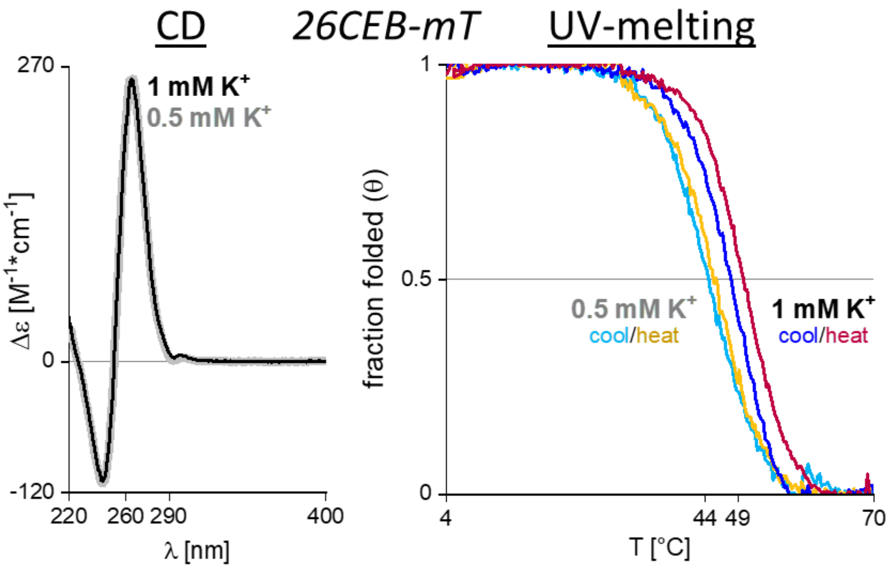

*Figure S24. CD spectra of 26CEB-mT (dAA$\mathbf{G_3}$T$\mathbf{G_3}$TTTTTTT**G**T$\mathbf{G_3}$T$\mathbf{G_3}$T) in 1 mM (black) and 0.5 mM KCl (grey). **Right**: UV-melting curve in 1 mM (blue/red) KCl. Samples contain 10 µM DNA and 100 mM TMAA (pH 6.8).*

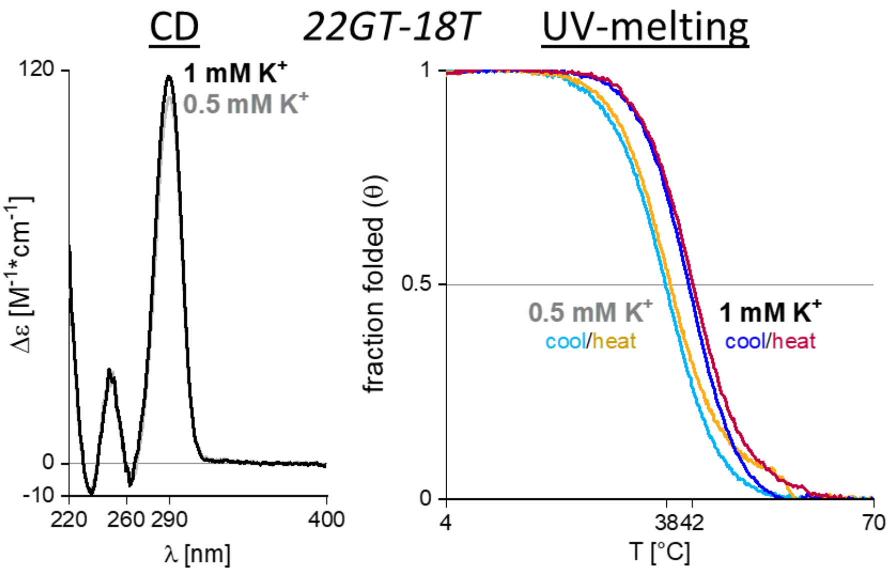

*Figure S25. CD spectra of 22GT-18T (d**G**$_3$TTA**G**$_3$TTA**G**$_3$TTT**G**$_3$T) in 1 mM (black) and 0.5 mM KCl (grey).* ***Right****: UV-melting curves in 1 mM (blue/red) and 0.5 mM (cyan/orange) KCl. Samples contain 10 µM DNA and 100 mM TMAA (pH 6.8).*

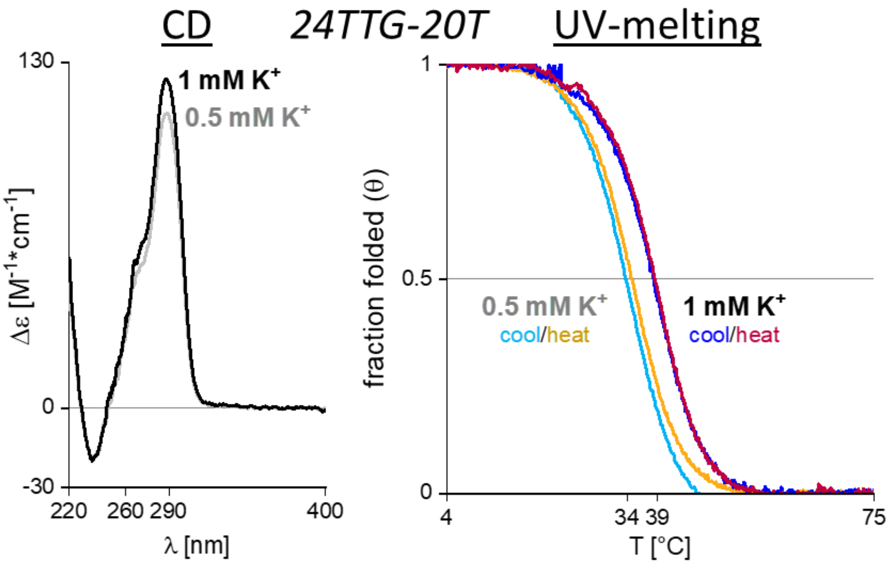

*Figure S26. CD spectra of 24TTG-20T (dTT**G**$_3$TTA**G**$_3$TTA**G**$_3$TTT**G**$_3$A) in 1 mM (black) and 0.5 mM KCl (grey).* ***Right****: UV-melting curves in 1 mM (blue/red) and 0.5 mM (cyan/orange) KCl. Samples contain 10 µM DNA and 100 mM TMAA (pH 6.8).*

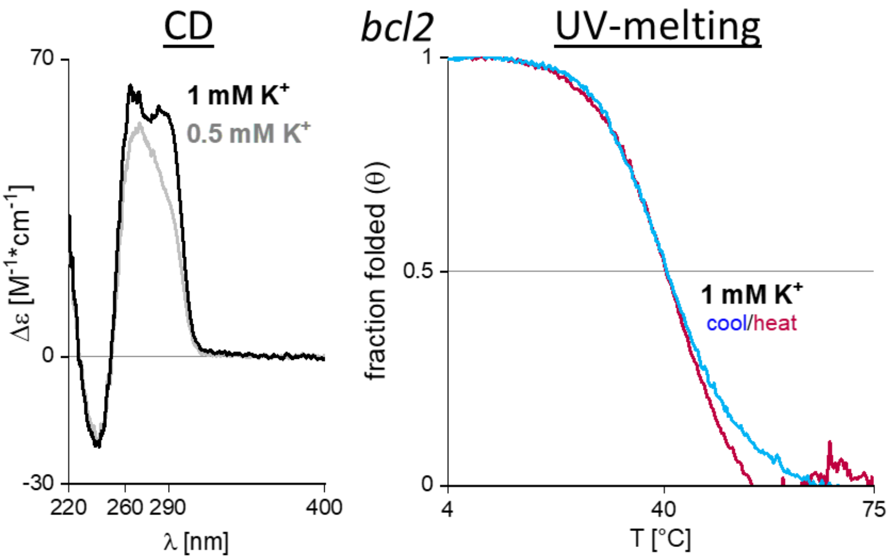

*Figure S27. CD spectra of bcl2 (d$\mathbf{G_3}$C**G**C$\mathbf{G_3}$A**GG**AATT$\mathbf{G_3}$C$\mathbf{G_3}$) in 1 mM (black) and 0.5 mM KCl (grey). **Right**: UV-melting curve in 1 mM (blue/red) KCl. No data could be obtained in 0.5 mM KCl due to lack of a low temperature baseline. Samples contain 10 µM DNA and 100 mM TMAA (pH 6.8).*

Figure S28 lets us directly compare the CD signatures of the mutated sequences with their 'wild-type' versions.

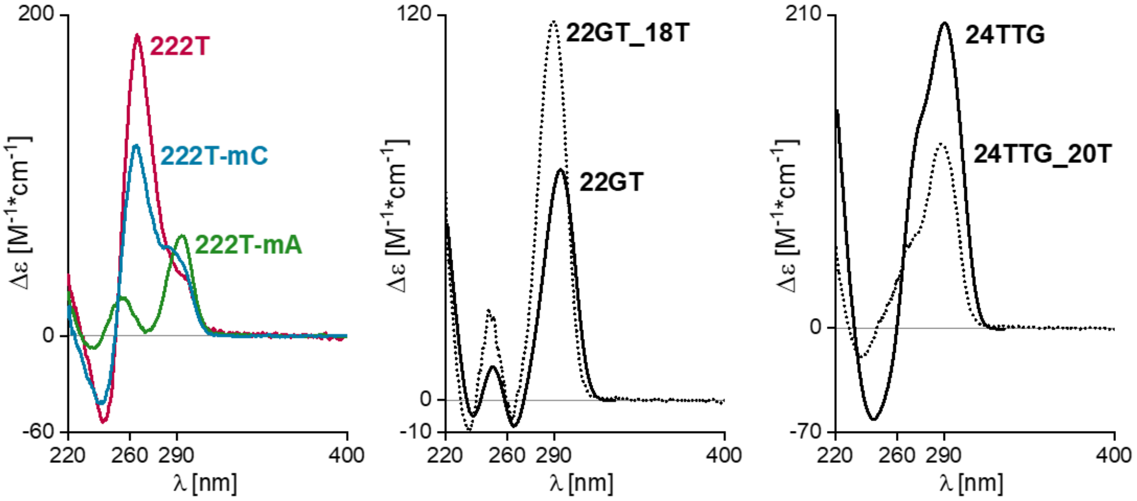

*Figure S28. Comparing CD signatures of 'wild-type' and mutant sequences. **Left**: The 222T sequence motif (dT$\mathbf{G_3}$TT$\mathbf{G_3}$<u>TT</u>$\mathbf{G_3}$TT$\mathbf{G_3}$T) in 0.5 mM KCl **Middle**: the 22GT sequence motif (d$\mathbf{G_3}$TTA$\mathbf{G_3}$TTA$\mathbf{G_3}$<u>TTA</u>$\mathbf{G_3}$T) in 1 mM KCl **Right**: The 24TTG sequence motif (dTT$\mathbf{G_3}$TTA$\mathbf{G_3}$TTA$\mathbf{G_3}$<u>TTA</u>$\mathbf{G_3}$A) in 1 mM KCl. Samples contain 10 µM DNA, 0.5 or 1 mM KCl and 100 mM TMAA (pH 6.8). 'wild-type' signatures of 22GT and 24TTG are taken from previously published data.[3]*

CD spectra of multi-stranded G-quadruplexes in NH_4^+ and K^+

The screening panel contains tetramolecular G-quadruplex $(dTG_4T)_4$ and bimolecular G-quadruplex $(dG_4T_4G_4)_2$. We know that these quadruplexes take weeks/months to fold completely in solution containing low concentrations of potassium.[4] We expected the formation to be notoriously slow in 0.5 mM K^+, which is why we measured another sample in 150 mM NH_4^+, hoping to speed up the formation kinetics and obtain quantitative amounts of G-quadruplex.

To our surprise, we noticed slight changes in ligand binding when the cation was switched (see Figure 4). We gathered CD spectra of both sequences in K^+ and NH_4^+ (Figure S29) after having let the samples rest for 10/11 months. The CD signatures do not change enough to indicate that switching from K^+ to NH_4^+ induces a change in topology. Since the true concentration of folded G-quadruplex is uncertain, we did not convert the ellipticity to Δε.

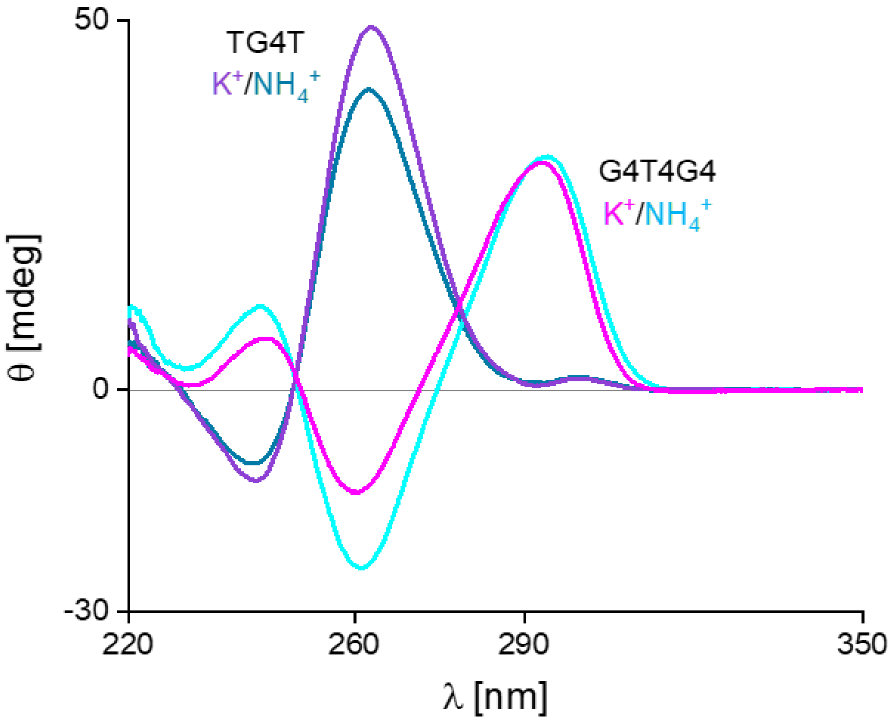

Figure S29. CD spectra of multi-stranded G-quadruplexes TG4T and G4T4G4 in different cation buffers. Samples contain: 10 µM G4T4G4/20 µM TG4T, 150 mM ammonium acetate (pH 6.8) or 0.5 mM KCl and 100 mM TMAA (pH 6.8).

Native ESI-MS

Methods

Parameters of the ESI-IMS-QTOF instrument

Table S1. Instrumental settings of the Agilent 6560 IMS-QTOF, optimized for native MS on oligonucleotides.

AREA	PARAMETER	VALUE	UNIT	AREA	PARAMETER	VALUE	UNIT
**Cell**	Col. Cell Flow	20	psig	**Ion Funnel**	Funnel RF HP	200	Volts
	Col. Cell Energy	0	Voltage		Funnel Delta LP	100	Volts
	Cell Exit	12.1	Volts		Funnel Delta HP	150	Volts
	Cell Entrance	-20	Volts		Funnel DC	50	Volts
	Hex dV	3	Volts	**Optics 1**	Lens 2	-16.5	Volts
	Hex DC Entrance	-20	Volts		Lens 1	-23	Volts
	Hex RF	550	Volts		Oct1 DC	-25	Volts
	Col. Cell Gas	Nitrogen		**Optics 2**	Bot Slit	41.35	Volts
**IM**	Trap Funnel Pressure	3.725	Torr		Top Slit	41.2	Volts
**IM Drift Tube**	DT Voltage	-210	Volts		Vertical Q	-12.9	Volts
	DT Entrance Voltage	-650	Volts		Horizontal Q	1.5	Volts
	DT Pressure	3.895	Torr		Slicer	10	Volts
	DT Temperature	24.3	°C		Ion Focus	-10	Volts
**IM Front Funnel**	HP Funnel Pressure	3.1	Torr		Oct 2 RF Vpp	600	Volts
	Trap Funnel RF	89	Volts		Oct2 DC	-14.6	Volts
	Trap Funnel Exit	-10.8	Volts	**Source**	Corona	65.9	uA
	HP Funnel Delta	-118	Volts		Gas Temp	280	°C
	HP Funnel RF	90	Volts		Nebulizer	12	psig
	Trap Funnel Delta	-121	Volts		Drying Gas	2	l/min
	HP Funnel Exit	0	Volts		Sheath Gas Flow	0.8	l/min
**IM Rear Funnel**	Rear Funnel RF	179	Volts		Ion Polarity	(negative)	
	Rear Funnel Exit	-35.4	Volts		Nozzle Voltage	0	Volts
	Rear Funnel Entrance	-199	Volts		Vcap	3500	Volts
	IM Hex RF	599	Volts		Oct 1 RF Vpp	750	Volts
	IM Hex Entrance	-31.8	Volts		Skimmer	-27	Volts
**IM Trap Funnel**	Exit Grid 2 Low	-72	Volts		Fragmentor	320	Volts
	Exit Grid 2 Delta	-63	Volts	**TOF**	Mirror Back	-1250	Volts
	Exit Grid 1 Low	-69	Volts		Mirror Mid	1675	Volts
	Exit Grid 1 Delta	-64	Volts		Mirror Front	7000	Volts
	Exit	-67	Volts		Acc Focus	1950	Volts
	Entrance Grid Low	-72	Volts		Puller Offset	-32	Volts
	Entrance Grid Delta	-69.9	Volts		Puller	700	Volts
	Entrance	-69	Volts		Pusher	-1200	Volts
**Ion Funnel**	Funnel RF LP	100	Volts		Min Range	62080	ns

Generating CCS distributions from ATD distributions

The instrument measures the arrival time (t_A) as the time between the release of ions from the trapping funnel until their detection at the TOF mass analyzer. The ion spends a portion of that time in the drift tube (drift time: t_D) and the other portion inside the rest of the instrument (dead time: t_0). We separate those contributions by operating the drift tube at different drift voltages. Figure S30 shows the shift of arrival time distribution (ATD) at different drift voltages (ΔV) from our external calibrant, which is a solution of 40 mM dTG_4T in 150 mM ammonium acetate.

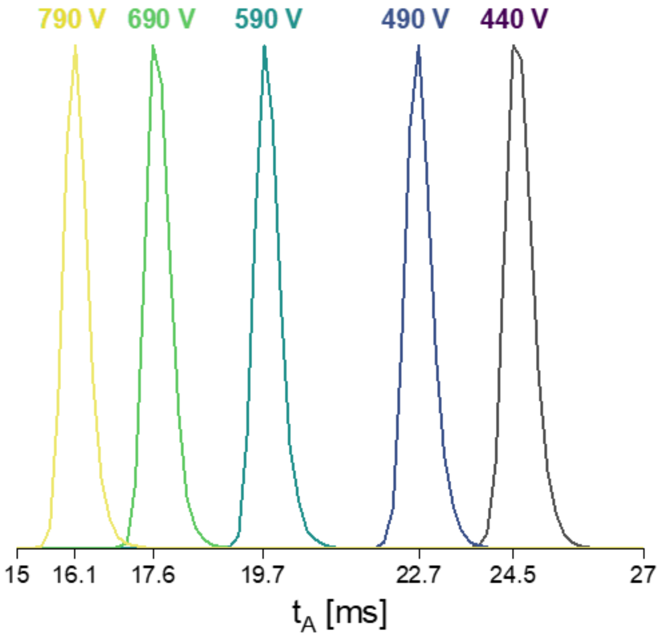

Figure S30. Arrival time distributions of the G-quadruplex species $[(dTG_4T)_4{*}3NH_4^+]^{5-}$ at different drift voltages.

The drift time t_D is dependent on the drift voltage ΔV, whereas the dead time is not.

$$t_D = \frac{L^2 * p * T_0}{K_0 * \Delta V * p_0 * T}$$

Where L is the length of the drift tube (78.1 cm), p_0/T_0 are the standard pressure/temperature (1 bar, 273 K), p/T are the pressure/temperature inside the drift tube (which are recorded for each experiment) and K_0 is the reduced mobility of the ion species (In this case: $[(dTG_4T)_4{*}3NH_4^+]^{5-}$). Based on this, we can formulate the correlation between arrival time and drift voltage as a linear function.

$$t_A = t_0 + t_D = t_0 + \frac{L^2 * p * T_0}{K_0 * p_0 * T} * \frac{1}{\Delta V}$$

The plot is shown in Figure S31. We extract the dead time from the y-intercept and the reduced ion mobility from the slope. We convert the reduced ion mobility K_0 to the momentum transfer cross section Ω (commonly referred to as the collisional cross section *CCS*) using the Mason-Schamp equation. Note that this equation follows the assumption of a static drift gas and low-field conditions.

$$CCS \cong \Omega = \frac{3}{16} * \frac{z * e}{N_0 * K_0} * \sqrt{\frac{2\pi}{\mu * k_B * T}}$$

The equation features several constants: e, k_B and N_0 (Loschmidt constant). The temperature T and the reduced mass μ ($\mu = (M_{ion}{*}M_{He})/(M_{ion}+M_{He})$) remain practically constant within the

experimental conditions. The reduced mobility K_0 and charge state z will be different for every ion species.

This method produces a single CCS-value which corresponds to the peak maxima of the arrival time distributions. For the calibrant TG_4T, our CCS-values are 788.7 Å^2 for the 5- ion (Figure S31) and 740.4 Å^2 for the 4- ion (not shown). These values are in good agreement with previously published experimental results (787.5 Å^2 [5-], 735.7 Å^2 [4-]).[5]

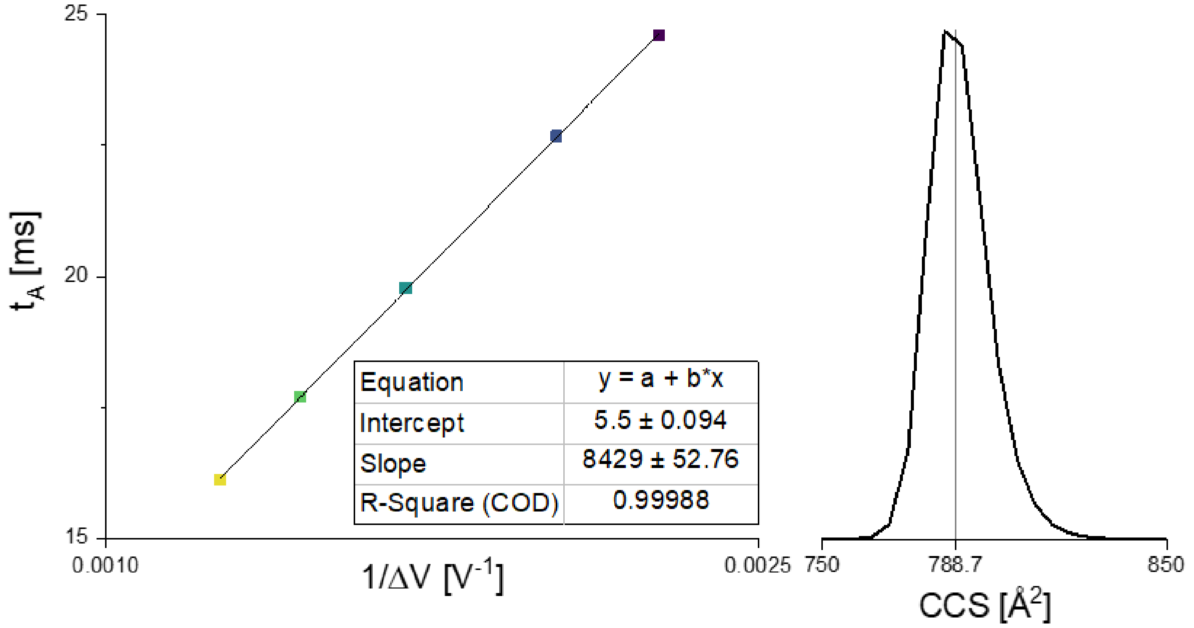

Figure S31 ***Left****: Obtaining drift time and reduced ion mobility by linearly fitting the arrival time at different drift voltages.* ***Right****: CCS distribution of $[(dTG_4T)_4{*}3NH_4^+]^{5-}$, calculated from the reduced ion mobility using the Mason-Schamp equation.*

We generate CCS distributions by converting the arrival time t_A to the respective CCS value using the ratio of CCS value and arrival time at peak maximum as a proportionality factor.

$$CCS = t_A * \frac{CCS_{max}}{t_{A,max}}$$

Conceptually, any of the five ATDs shown in Figure S30 can be converted using this method. To be as close as possible to the low-field limit, we consistently choose the ATD at the lowest drift voltage.

Data processing of native ESI-MS titrations

Using dT_6 as an internal calibrant we quantify the change in DNA response.[6] Figure S32 shows how the ratio of collective DNA signal vs. dT_6 signal evolves as a function of ligand concentration. The estimated response factors are listed in table. Based on the estimates we allowed the response factors for the complex signals to fluctuate between 0.8 and 1.1 during dynamic fitting.

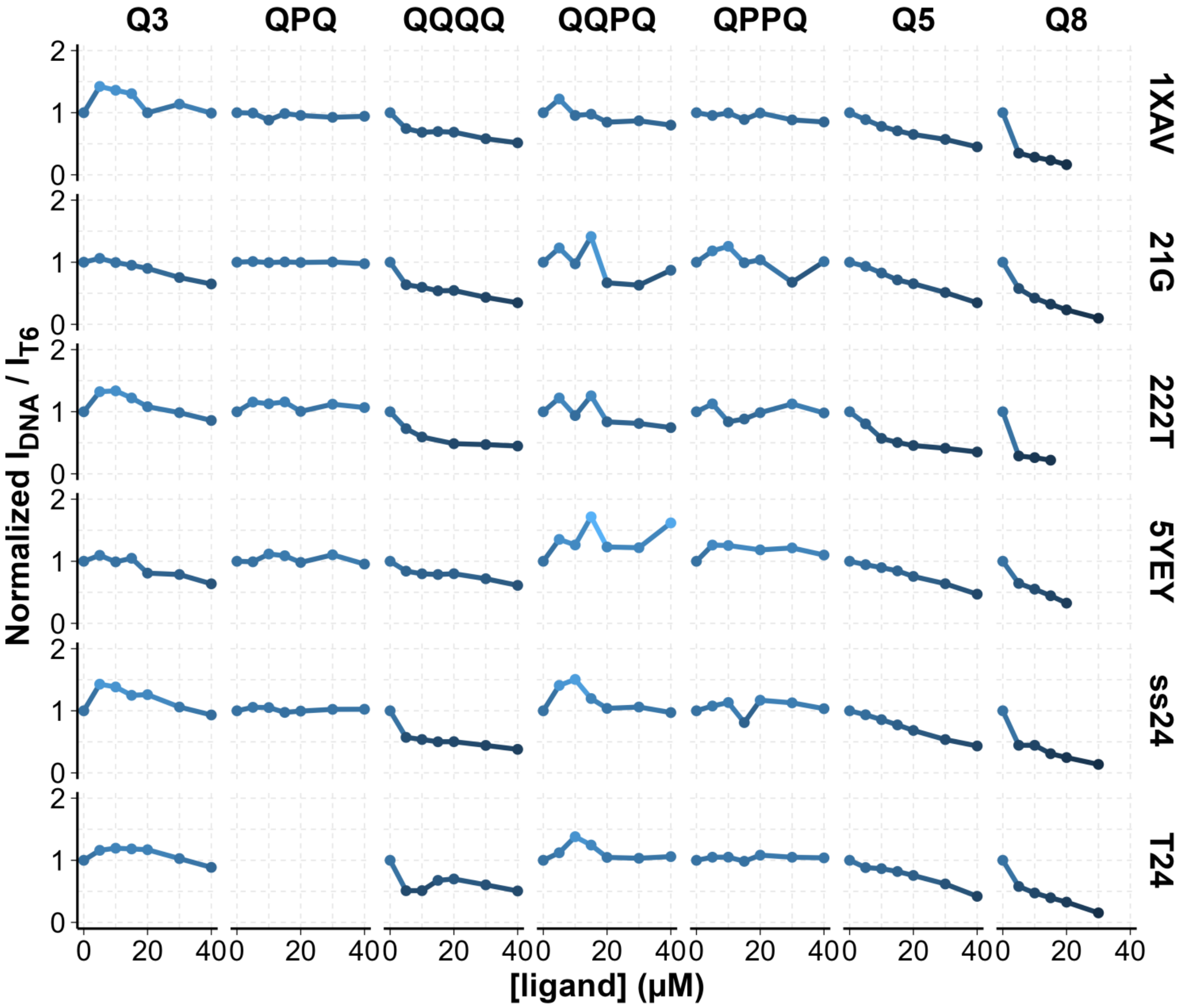

Figure S32. The sum of all DNA signal intensities vs. the signal of calibrant dT_6. The first datapoint is normalized to 1.

A declining curve means that adding ligand negatively impacts the detection of DNA ions. This leads to an ever-decreasing S/N ratio until the DNA species fall under the limit of detection. The latter happened with Q_8, which is why the titration datasets with Q_8 are incomplete.

The titration data points, corrected for noise and response, are shown in Figure S33, alongside the dynamic fits from which we obtained refined K_D values. All K_D values are listed in Table S3.

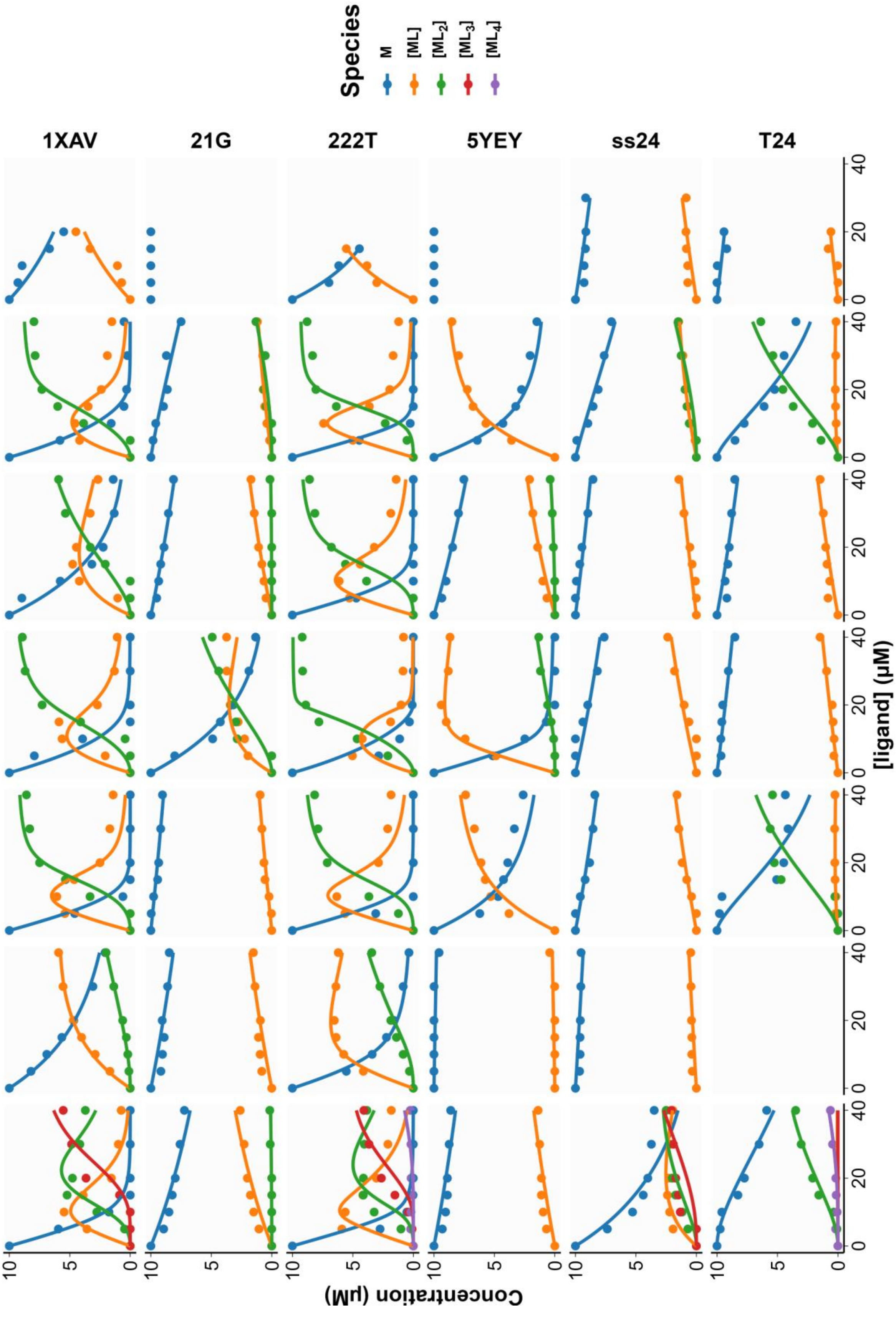

Figure S33. Data points obtained from processing the ESI-MS titration mass spectra. K_D values are calculated by dynamically fitting the experimental data based on the complex formation equilibria. Curves show the dynamic fits. Samples contain 10 µM DNA, 0-40 µM ligand, 4 µM dT_6, 0.5 mM KCl and 100 mM TMAA (pH 6.8).

Ligand screening

Comprehensive list of DNA/Ligand species concentrations and their K_D values

Table S2. Data extracted from the ligand screening, featuring: Concentration of unbound DNA (M), 1:1 complex (ML) and 2:1 complex (ML_2), fraction of DNA bound (fb), complex dissociation constants K_{D1} and K_{D2} as well as ligand cooperativity. Sample conditions were: 10 µM DNA, 20 µM ligand, 0.5 mM KCl, 100 mM TMAA (pH 6.8).

DNA	Ligand	M [µM]	ML [µM]	ML_2 [µM]	fb	K_{D1} [µM]	K_{D2} [µM]	Coop.[1]
**1XAV**	QQPQ	0.4	3.7	5.9	*0.96*	**0.5**	**1.5**	positive
**1XAV**	QQQQ	0.3	3.2	6.5	*0.97*	**0.3**	**0.9**	positive
**1XAV**	QPPQ	2.5	4.6	3.0	*0.75*	**5.2**	**7.4**	positive
**1XAV**	QPQ	4.7	4.2	1.1	*0.53*	**15**	**27**	positive
**222T**	QQPQ	0.4	4.3	5.3	*0.96*	**0.5**	**2.1**	negative
**222T**	QQQQ	0.8	3.9	5.4	*0.92*	**1.1**	**2.0**	positive
**222T**	QPPQ	1.4	5.0	3.6	*0.86*	**2.3**	**5.4**	positive
**222T**	QPQ	2.6	5.9	1.5	*0.74*	**4.8**	**22**	negative
**222T_mA**	QQPQ	1.2	2.6	6.3	*0.88*	**2.2**	**1.0**	positive
**222T_mA**	QQQQ	3.6	3.0	3.4	*0.64*	**12**	**4.5**	positive
**222T_mA**	QPPQ	3.0	3.0	4.0	*0.70*	**8.9**	**3.4**	positive
**222T_mA**	QPQ	5.5	3.0	1.5	*0.45*	**25**	**14**	positive
**222T_mC**	QQPQ	0.5	2.9	6.5	*0.95*	**0.7**	**0.9**	positive
**222T_mC**	QQQQ	1.9	4.0	4.1	*0.81*	**3.7**	**3.7**	positive
**222T_mC**	QPPQ	2.2	3.7	4.1	*0.78*	**4.9**	**3.6**	positive
**222T_mC**	QPQ	3.0	5.1	1.9	*0.70*	**6.6**	**15**	positive
**T30177TT**	QQPQ	0.9	4.7	4.4	*0.91*	**1.2**	**3.4**	positive
**T30177TT**	QQQQ	0.7	4.5	4.7	*0.93*	**1.0**	**2.9**	positive
**T30177TT**	QPPQ	1.5	6.2	2.4	*0.85*	**2.1**	**12**	negative
**T30177TT**	QPQ	2.9	6.2	0.8	*0.71*	**5.7**	**47**	negative
**26CEB**	QQPQ	1.2	8.2	0.5	*0.88*	**1.6**	**83**	negative
**26CEB**	QQQQ	0.7	8.4	0.9	*0.93*	**0.8**	**45**	negative
**26CEB**	QPPQ	1.8	7.7	0.5	*0.82*	**2.6**	**95**	negative
**26CEB**	QPQ	3.3	6.4	0.3	*0.67*	**6.6**	**136**	negative
**26CEB_mT**	QQPQ	1.2	7.9	0.9	*0.88*	**1.5**	**43**	negative
**26CEB_mT**	QQQQ	2.2	6.2	1.6	*0.78*	**3.8**	**21**	negative
**26CEB_mT**	QPPQ	2.1	7.3	0.7	*0.79*	**3.3**	**63**	negative
**26CEB_mT**	QPQ	3.6	6.0	0.4	*0.64*	**8.1**	**103**	negative
**2KYP**	QQPQ	2.2	2.4	5.4	*0.78*	**6.2**	**1.5**	positive
**2KYP**	QQQQ	2.7	6.6	0.7	*0.73*	**4.9**	**57**	negative
**2KYP**	QPPQ	7.1	1.7	1.2	*0.29*	**66**	**11**	positive
**2KYP**	QPQ	8.0	1.7	0.3	*0.20*	**84**	**47**	positive
**2O3M**	QQPQ	7.2	1.6	1.2	*0.28*	**71**	**11**	positive
**2O3M**	QQQQ	7.8	2.2	0	*0.22*	**64**	**0**	
**2O3M**	QPPQ	7.5	2.2	0.3	*0.25*	**58**	**58**	positive
**2O3M**	QPQ	8.6	1.4	0	*0.14*	**119**	**0**	
**TG4T_K**	QQPQ	4.2	5.0	0.8	*0.58*	**11**	**43**	positive
**TG4T_K**	QQQQ	7.3	2.7	0	*0.27*	**46**	**0**	
**TG4T_K**	QPPQ	5.7	3.8	0.5	*0.43*	**23**	**60**	positive
**TG4T_K**	QPQ	8.0	2.0	0	*0.20*	**70**	**0**	
**TG4T_NH4**	QQPQ	2.8	6.2	1.0	*0.72*	**5.3**	**37**	negative
**TG4T_NH4**	QQQQ	8.9	1.1	0	*0.11*	**149**	**0**	
**TG4T_NH4**	QPPQ	5.8	3.9	0.3	*0.42*	**23**	**101**	negative

[1] Positive, if $4*K_{D1} > K_{D2}$. Negative, if $4*K_{D1} < K_{D2}$. Assuming independent and equivalent binding sites.[7]

**TG4T_NH4**	QPQ	7.5	2.3	0.2	*0.25*	**58**	**114**	positive
**21G**	QQPQ	5.9	2.6	1.5	*0.41*	**32**	**13**	positive
**21G**	QQQQ	9.1	0.9	0	*0.09*	**197**	**0**	
**21G**	QPPQ	8.4	1.3	0.2	*0.16*	**116**	**53**	positive
**21G**	QPQ	9.3	0.7	0	*0.07*	**250**	**0**	
**5YEY**	QQPQ	1.2	7.3	1.6	*0.88*	**1.5**	**22**	negative
**5YEY**	QQQQ	5.0	5.0	0	*0.50*	**15**	**0**	
**5YEY**	QPPQ	7.8	1.8	0.4	*0.22*	**75**	**40**	positive
**5YEY**	QPQ	9.4	0.6	0	*0.06*	**309**	**0**	
**22GT**	QQPQ	5.5	3.1	1.4	*0.45*	**25**	**16**	positive
**22GT**	QQQQ	8.5	1.5	0	*0.15*	**104**	**0**	
**22GT**	QPPQ	8.0	1.6	0.4	*0.20*	**89**	**36**	positive
**22GT**	QPQ	9.1	0.9	0	*0.09*	**196**	**0**	
**22GT_18T**	QQPQ	2.6	6.5	0.9	*0.74*	**4.6**	**41**	negative
**22GT_18T**	QQQQ	5.4	4.6	0	*0.46*	**18**	**0**	
**22GT_18T**	QPPQ	7.7	1.9	0.4	*0.23*	**69**	**45**	positive
**22GT_18T**	QPQ	8.8	1.2	0.0	*0.12*	**143**	**0**	
**22CTA**	QQPQ	7.0	2.0	1.0	*0.30*	**57**	**16**	positive
**22CTA**	QQQQ	8.6	1.4	0	*0.14*	**114**	**0**	
**22CTA**	QPPQ	8.6	1.4	0	*0.14*	**116**	**0**	
**22CTA**	QPQ	8.8	1.2	0	*0.12*	**135**	**0**	
**TBA**	QQPQ	6.7	3.3	0	*0.33*	**34**	**0**	
**TBA**	QQQQ	8.1	1.9	0	*0.19*	**78**	**0**	
**TBA**	QPPQ	8.6	1.4	0	*0.14*	**118**	**0**	
**TBA**	QPQ	9.3	0.7	0	*0.07*	**252**	**0**	
**G4T4G4_K**	QQPQ	6.1	3.3	0.6	*0.39*	**28**	**39**	positive
**G4T4G4_K**	QQQQ	8.2	1.8	0	*0.18*	**81**	**0**	
**G4T4G4_K**	QPPQ	7.9	2.1	0	*0.21*	**67**	**0**	
**G4T4G4_K**	QPQ	8.4	1.6	0	*0.16*	**99**	**0**	
**G4T4G4_NH4**	QQPQ	5.8	3.7	0.5	*0.42*	**24**	**53**	positive
**G4T4G4_NH4**	QQQQ	9.7	0.3	0	*0.03*	**562**	**0**	
**G4T4G4_NH4**	QPPQ	9.2	0.8	0	*0.08*	**225**	**0**	
**G4T4G4_NH4**	QPQ	9.6	0.4	0	*0.04*	**470**	**0**	
**26TTA**[2]	QQPQ	2.1	3.8	3.3	*0.71*	**4.1**	**4**	positive
**26TTA**	QQQQ	6.8	2.2	1.0	*0.32*	**48**	**18**	positive
**26TTA**	QPPQ	7.7	1.9	0.4	*0.23*	**70**	**40**	positive
**26TTA**	QPQ	9.1	0.9	0	*0.09*	**204**	**0**	
**Bcl2**	QQPQ	4.4	3.4	2.1	*0.56*	**16**	**9.8**	positive
**Bcl2**	QQQQ	4.0	4.0	1.9	*0.60*	**12**	**13**	positive
**Bcl2**	QPPQ	5.3	3.6	1.1	*0.47*	**21**	**24**	positive
**Bcl2**	QPQ	7.5	2.5	0	*0.25*	**52**	**0**	
**24TTG**	QQPQ	7.1	1.1	1.8	*0.29*	**102**	**4.6**	positive
**24TTG**	QQQQ	9.8	0.2	0	*0.02*	**809**	**0**	
**24TTG**	QPPQ	8.8	1.2	0	*0.12*	**145**	**0**	
**24TTG**	QPQ	9.4	0.6	0	*0.06*	**284**	**0**	
**24TTG_20T**	QQPQ	5.2	3.6	1.2	*0.48*	**20**	**22**	positive
**24TTG_20T**	QQQQ	6.0	4.0	0	*0.40*	**24**	**0**	
**24TTG_20T**	QPPQ	8.3	1.7	0	*0.17*	**92**	**0**	
**24TTG_20T**	QPQ	8.9	1.1	0	*0.11*	**153**	**0**	positive
**23TAG**	QQPQ	5.7	3.4	1.0	*0.43*	**25**	**25**	positive
**23TAG**	QQQQ	8.9	1.1	0	*0.11*	**148**	**0**	

[2] This is the only instance where we observed a 3:1 complex. $[ML_3]$ = 0.8 μM, K_{D3} = 22 μM (negative coop.).

**23TAG**	QPPQ	8.1	1.9	0	*0.19*	**75**	**0**	
**23TAG**	QPQ	8.3	1.7	0	*0.17*	**93**	**0**	
**2KPR**	QQPQ	4.6	1.9	3.5	*0.54*	**26**	**3**	positive
**2KPR**	QQQQ	8.7	1.3	0	*0.13*	**131**	**0**	
**2KPR**	QPPQ	7.0	2.0	1.0	*0.30*	**56**	**17**	positive
**2KPR**	QPQ	8.6	1.1	0.3	*0.14*	**144**	**37**	positive
**21CCC (i-m)**	QQPQ	9.4	0.6	0	*0.06*	**294**	**0**	
**21CCC (i-m)**	QQQQ	10	0	0	*0.00*	**0**	**0**	
**21CCC (i-m)**	QPPQ	9.4	0.6	0	*0.06*	**290**	**0**	
**21CCC (i-m)**	QPQ	9.8	0.2	0	*0.02*	**1280**	**0**	
**ds26**	QQPQ	8.7	1.3	0	*0.13*	**120**	**0**	
**ds26**	QQQQ	10	0	0	*0.00*	**0**	**0**	
**ds26**	QPPQ	8.8	0	1.2	*0.12*	**0**	**0**	
**ds26**	QPQ	10	0	0	*0.00*	**0**	**0**	
**DK33**	QQPQ	8.5	1.5	0	*0.15*	**107**	**0**	
**DK33**	QQQQ	10	0	0	*0.00*	**0**	**0**	
**DK33**	QPPQ	8.1	1.9	0	*0.19*	**80**	**0**	
**DK33**	QPQ	8.8	1.2	0	*0.12*	**141**	**0**	
**DK66**	QQPQ	8.7	1.3	0	*0.13*	**123**	**0**	
**DK66**	QQQQ	10	0	0	*0.00*	**3981**	**0**	
**DK66**	QPPQ	8.3	1.7	0	*0.17*	**88**	**0**	
**DK66**	QPQ	9.7	0.3	0	*0.03*	**586**	**0**	
**DK100**	QQPQ	7.7	2.3	0	*0.23*	**58**	**0**	
**DK100**	QQQQ	10	0.0	0	*0.00*	**0**	**0**	
**DK100**	QPPQ	8.1	1.9	0	*0.19*	**77**	**0**	
**DK100**	QPQ	10	0	0	*0.00*	**0**	**0**	
**ss24**	QQPQ	8.2	1.8	0	*0.18*	**83**	**0**	
**ss24**	QQQQ	8.7	1.3	0	*0.13*	**126**	**0**	
**ss24**	QPPQ	9.1	0.9	0	*0.09*	**182**	**0**	
**ss24**	QPQ	9.7	0.3	0	*0.03*	**626**	**0**	
**24nonG4**	QQPQ	9.2	0.8	0	*0.08*	**208**	**0**	
**24nonG4**	QQQQ	7.2	2.8	0	*0.28*	**44**	**0**	
**24nonG4**	QPPQ	7.5	2.5	0	*0.25*	**51**	**0**	
**24nonG4**	QPQ	10	0	0	*0.00*	**0**	**0**	
**T24**	QQPQ	8.3	1.5	0.2	*0.17*	**102**	**65**	positive
**T24**	QQQQ	6.8	0.4	2.8	*0.32*	**235**	**1.0**	positive
**T24**	QPPQ	8.8	1.1	0.1	*0.12*	**149**	**195**	positive
**T24**	QPQ	9.2	0.8	0	*0.08*	**210**	**0**	
**A24**	QQPQ	10	0	0	*0.00*	**0**	**0**	
**A24**	QQQQ	10	0	0	*0.00*	**0**	**0**	
**A24**	QPPQ	8.3	1.7	0	*0.17*	**92**	**0**	
**A24**	QPQ	8.8	1.2	0	*0.12*	**138**	**0**	
**21CCC (ss)**	QQPQ	9.5	0.5	0	*0.05*	**354**	**0**	
**21CCC (ss)**	QQQQ	10	0	0	*0.00*	**4064**	**0**	
**21CCC (ss)**	QPPQ	9.5	0.5	0	*0.05*	**359**	**0**	
**21CCC (ss)**	QPQ	9.9	0.1	0	*0.01*	**3365**	**0**	
**T6**	QQPQ	9.7	0.3	0	*0.03*	**722**	**0**	
**T6**	QQQQ	10	0	0	*0.00*	**0**	**0**	
**T6**	QPPQ	9.7	0.3	0	*0.03*	**725**	**0**	
**T6**	QPQ	9.8	0.2	0	*0.02*	**1150**	**0**	

Mass spectra

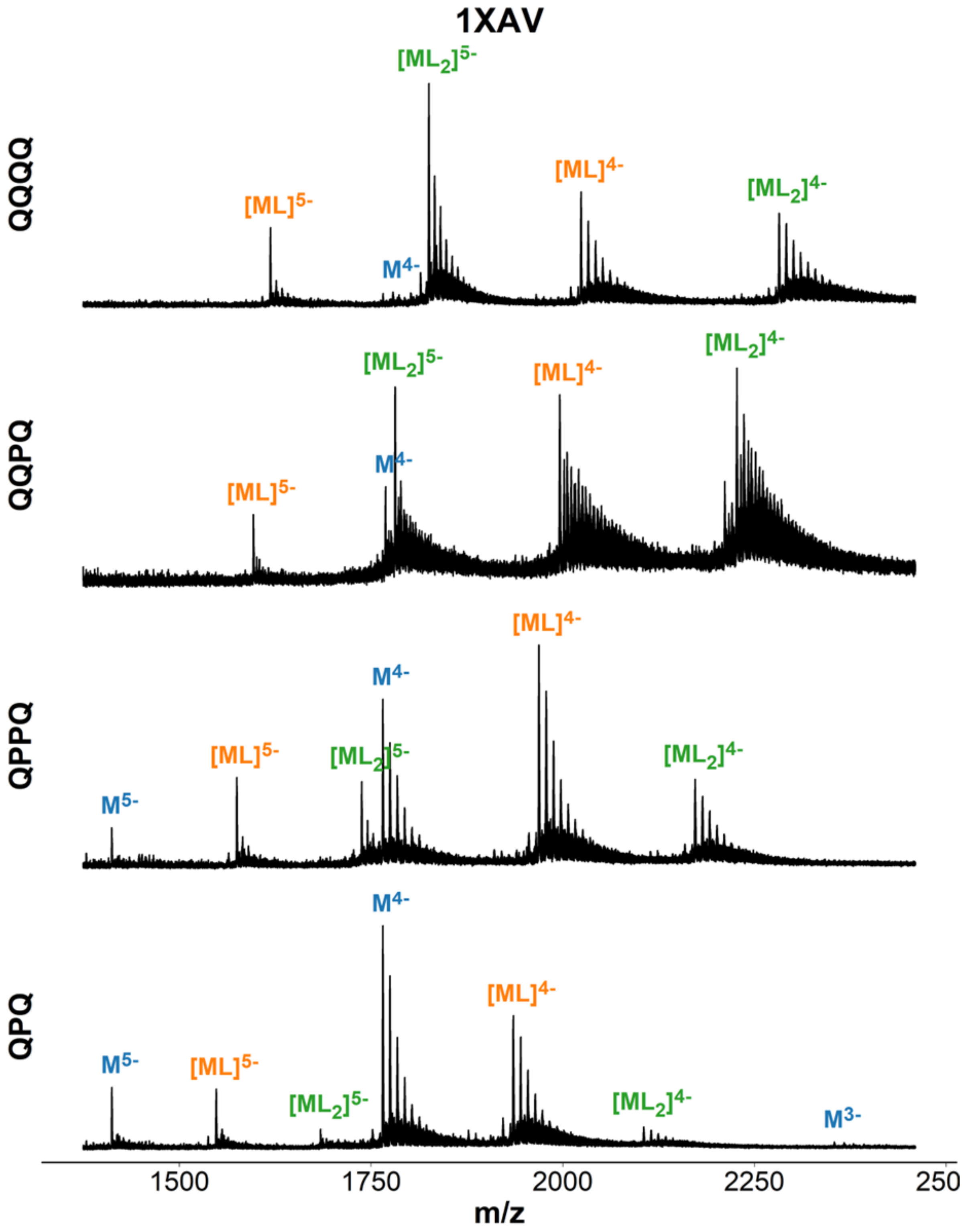

*Figure S34. Mass spectra of 1XAV (dT**GA**G**GG**T**GGG**TA**GGG**T**GGG**TAA) in presence of ligand. Samples contain 10 µM DNA, 20 µM ligand, 0.5 mM KCl, 100 mM TMAA (pH 6.8).*

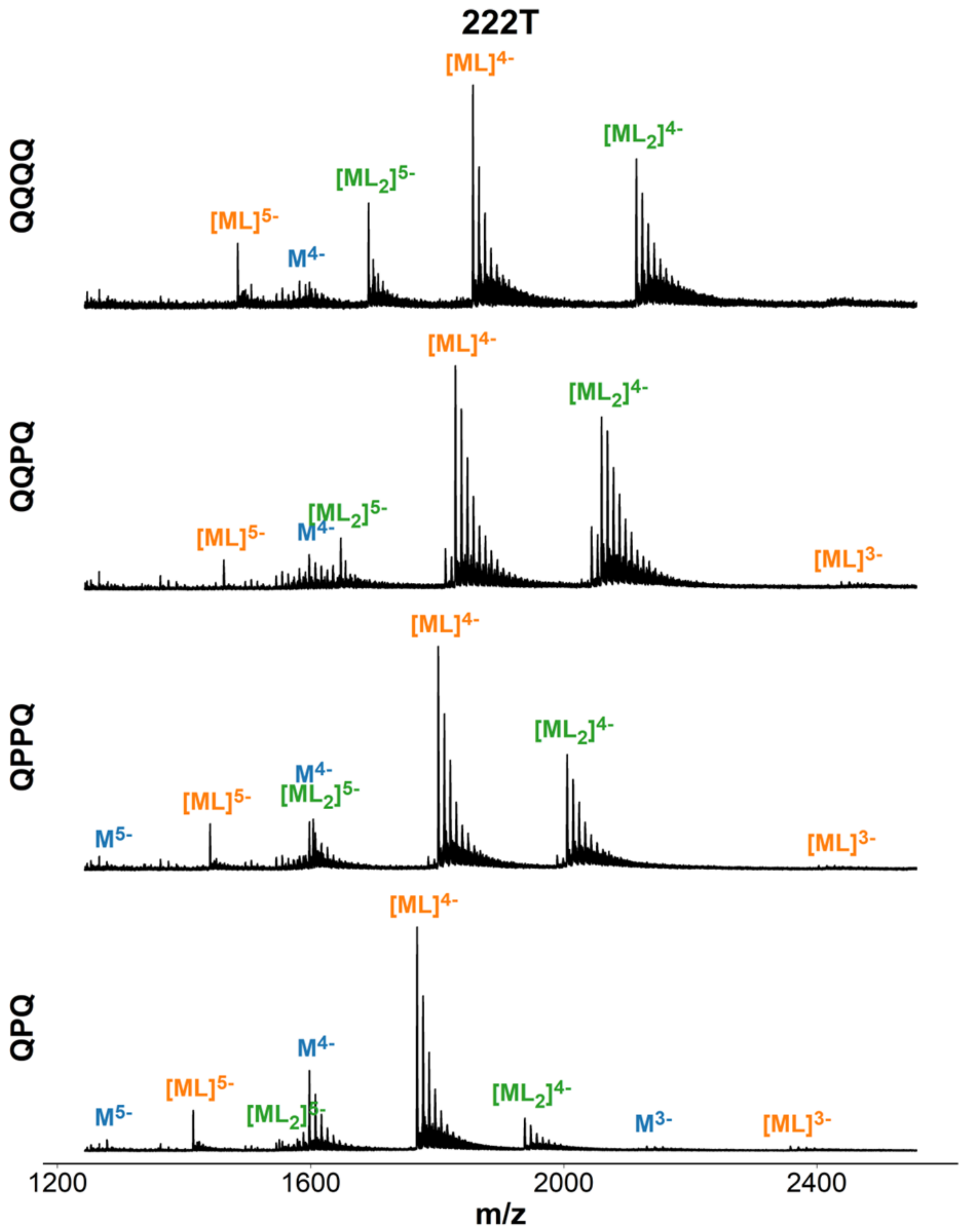

*Figure S35. Mass spectra of 222T (dT**GGG**TT**GGG**TT**GGG**TT**GGG**T) in presence of ligand. Samples contain 10 µM DNA, 20 µM ligand, 0.5 mM KCl, 100 mM TMAA (pH 6.8).*

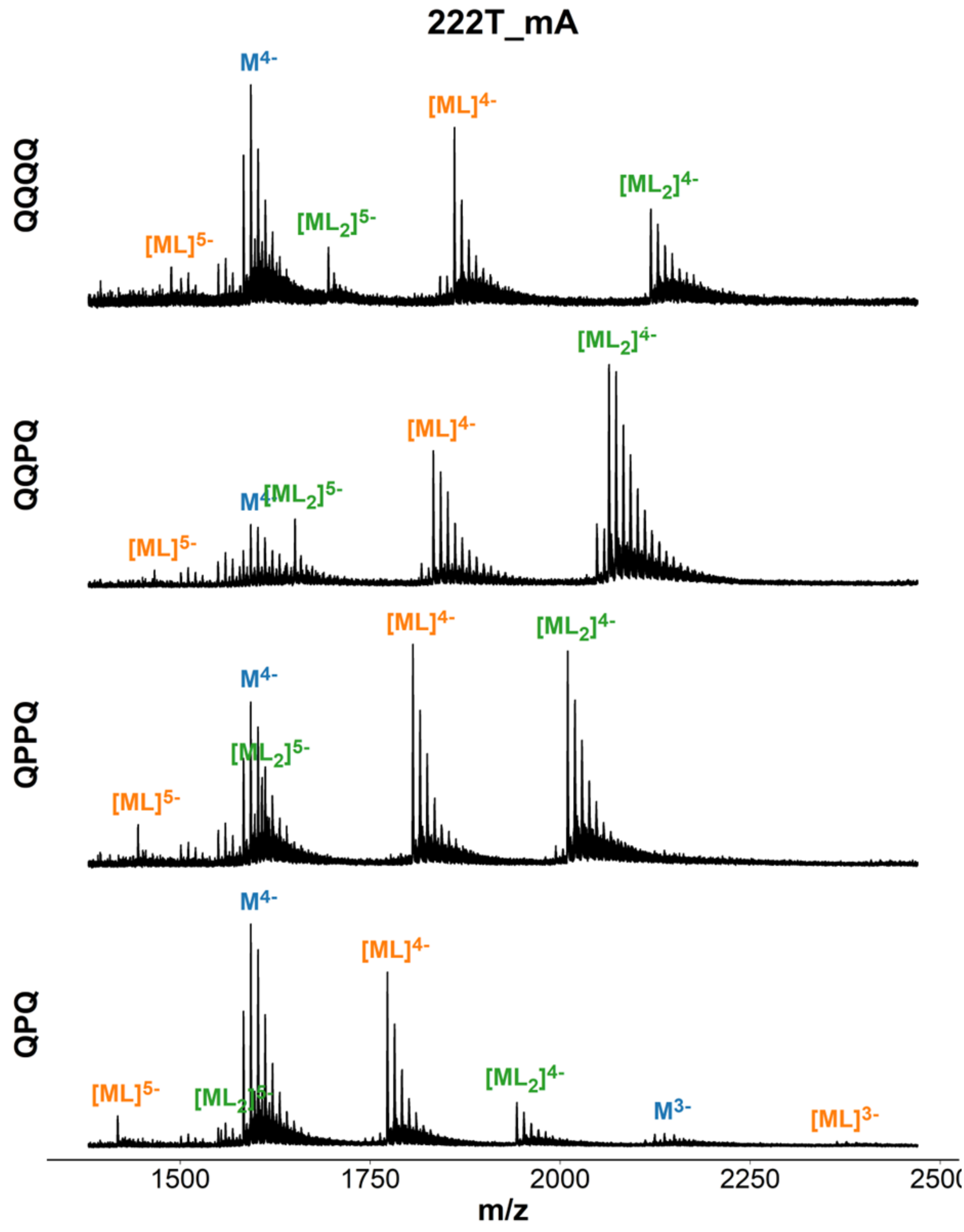

*Figure S36. Mass spectra of 222T-mA (dT**GGG**TT**GGG**AA**GGG**TT**GGG**T) in presence of ligand. Samples contain 10 µM DNA, 20 µM ligand, 0.5 mM KCl, 100 mM TMAA (pH 6.8).*

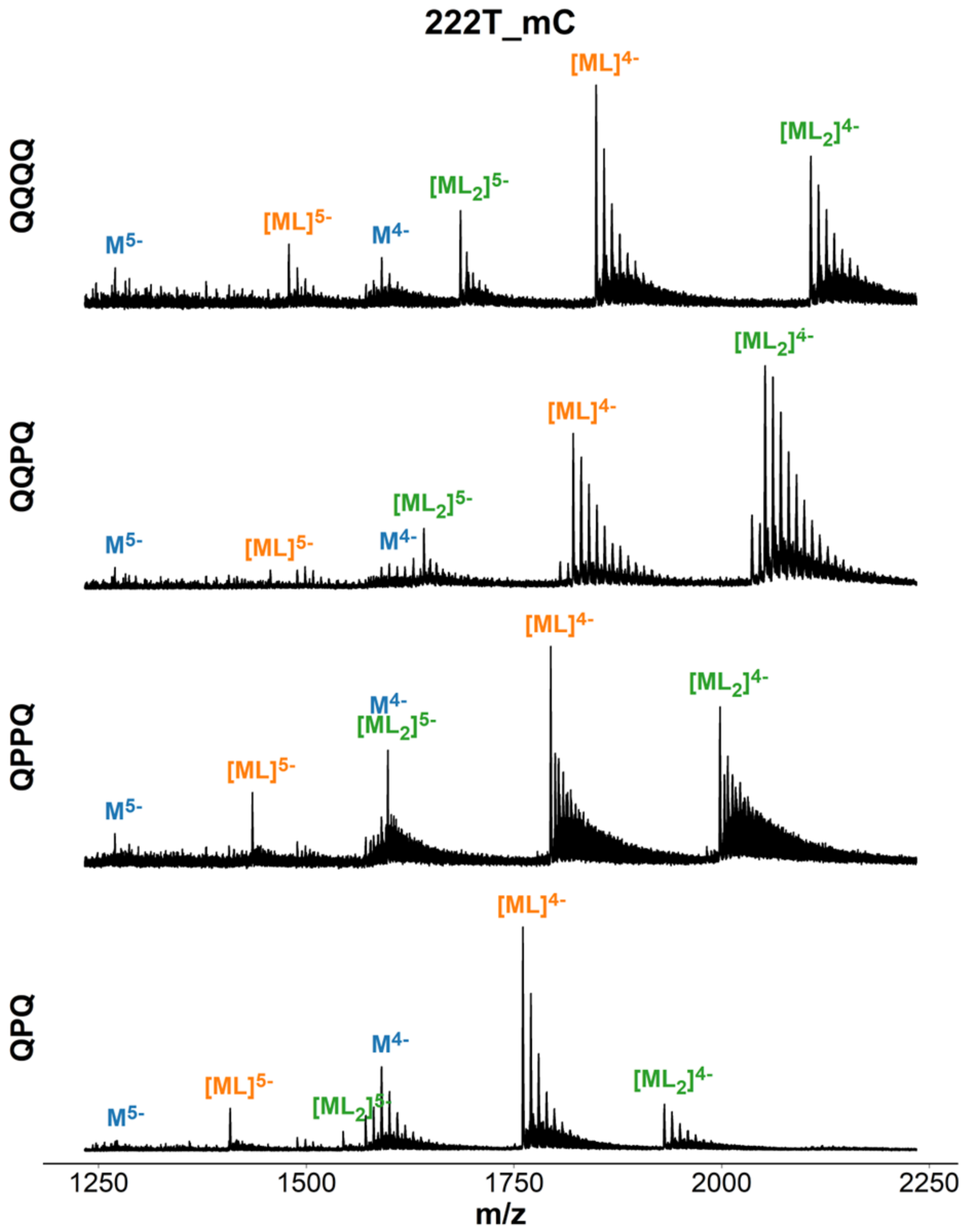

*Figure S37. Mass spectra of 222T-mC (dT**GGG**TT**GGG**CC**GGG**TT**GGG**T) in presence of ligand. Samples contain 10 µM DNA, 20 µM ligand, 0.5 mM KCl, 100 mM TMAA (pH 6.8).*

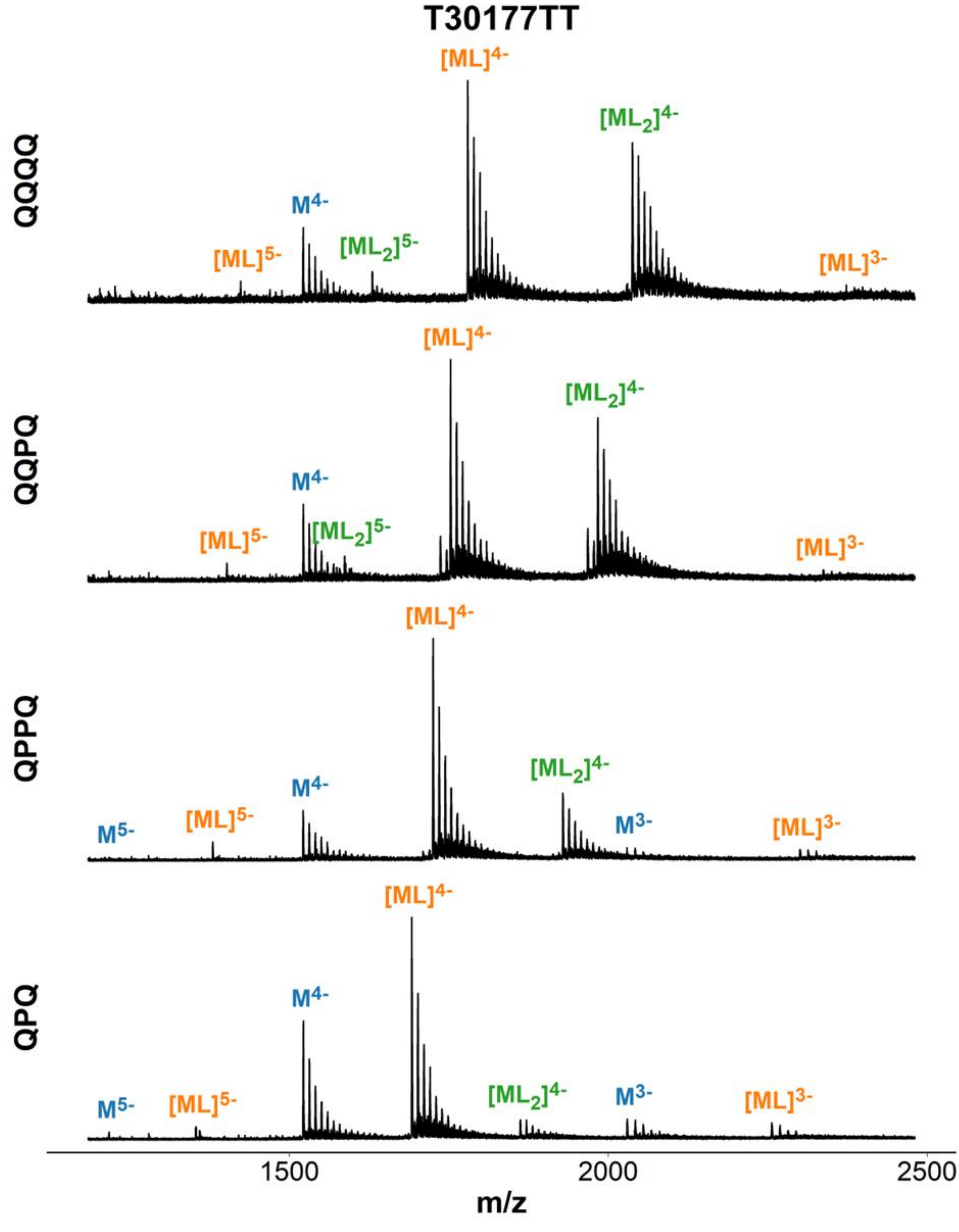

*Figure S38. Mass spectra of T30177TT (dTT**G**T**GG**T**GGG**T**GGG**T**GGG**T) in presence of ligand. Samples contain 10 µM DNA, 20 µM ligand, 0.5 mM KCl, 100 mM TMAA (pH 6.8).*

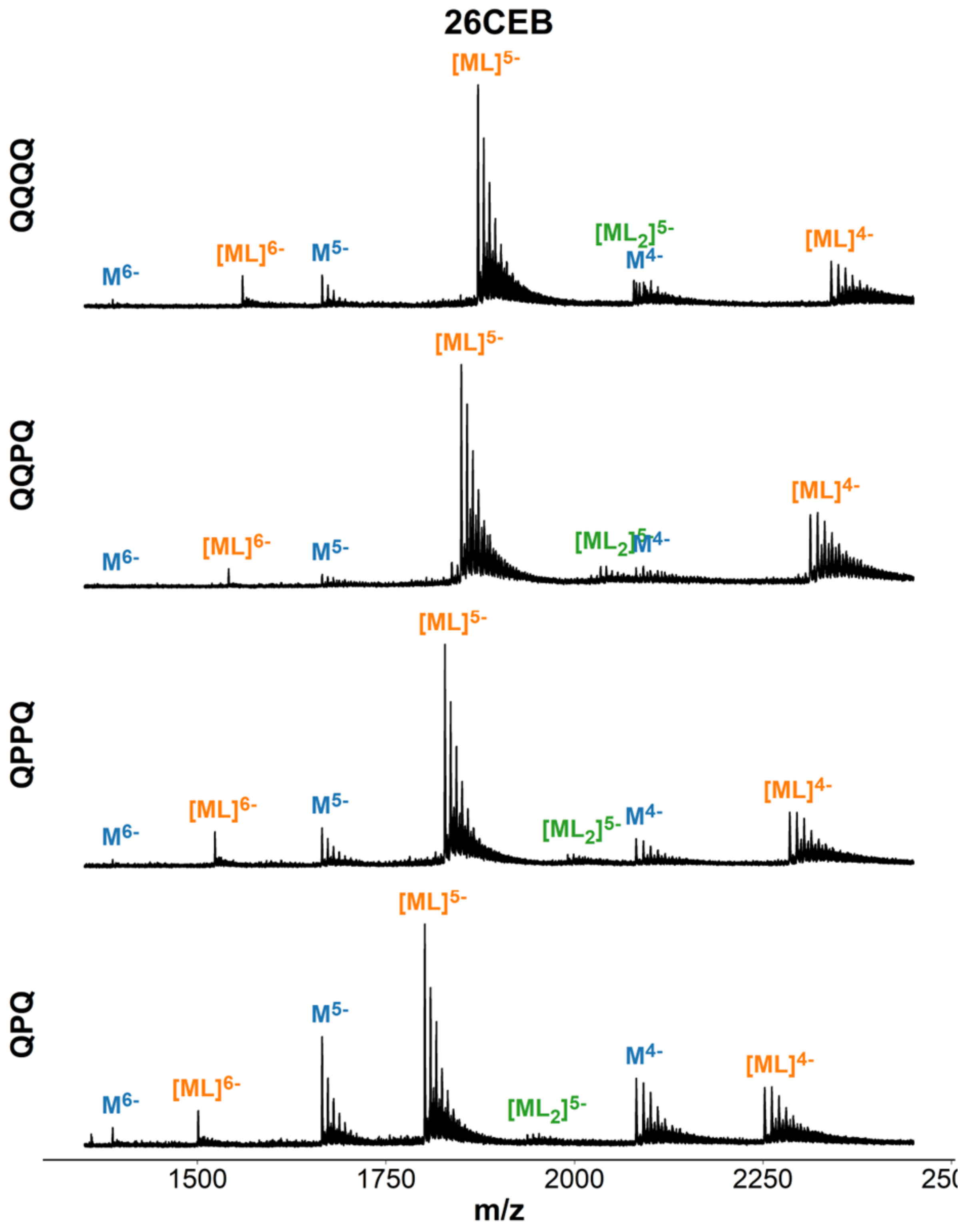

*Figure S39. Mass spectra of 26CEB (dAA**GGG**T**GGG**T**G**TAA**G**T**G**T**GGG**T**GGG**T) in presence of ligand. Samples contain 10 µM DNA, 20 µM ligand, 0.5 mM KCl, 100 mM TMAA (pH 6.8).*

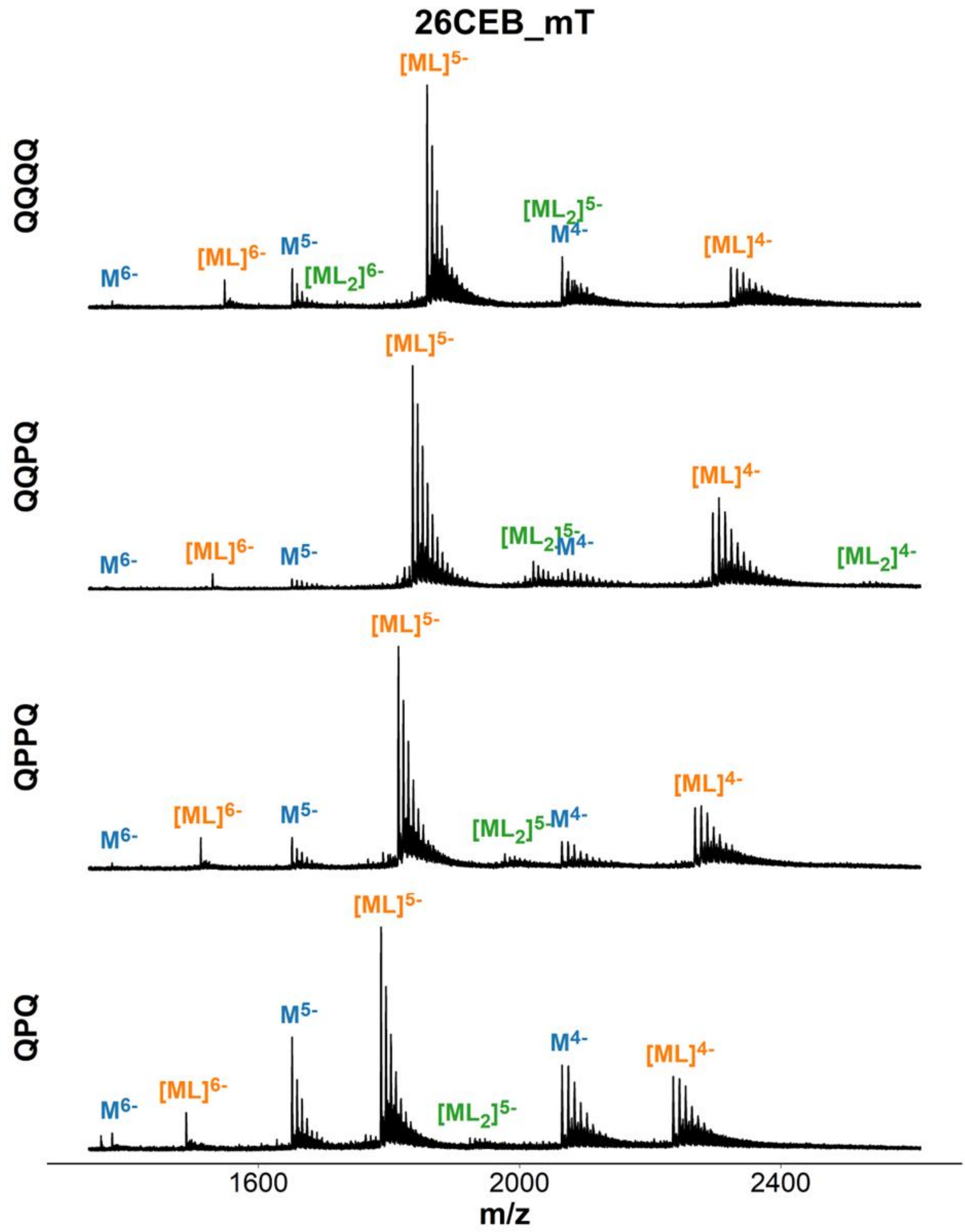

*Figure S40. Mass spectra of 26CEB-mT (dAA**GGG**T**GGG**TTTTTTT**G**T**GGG**T**GGG**T) in presence of ligand. Samples contain 10 µM DNA, 20 µM ligand, 0.5 mM KCl, 100 mM TMAA (pH 6.8).*

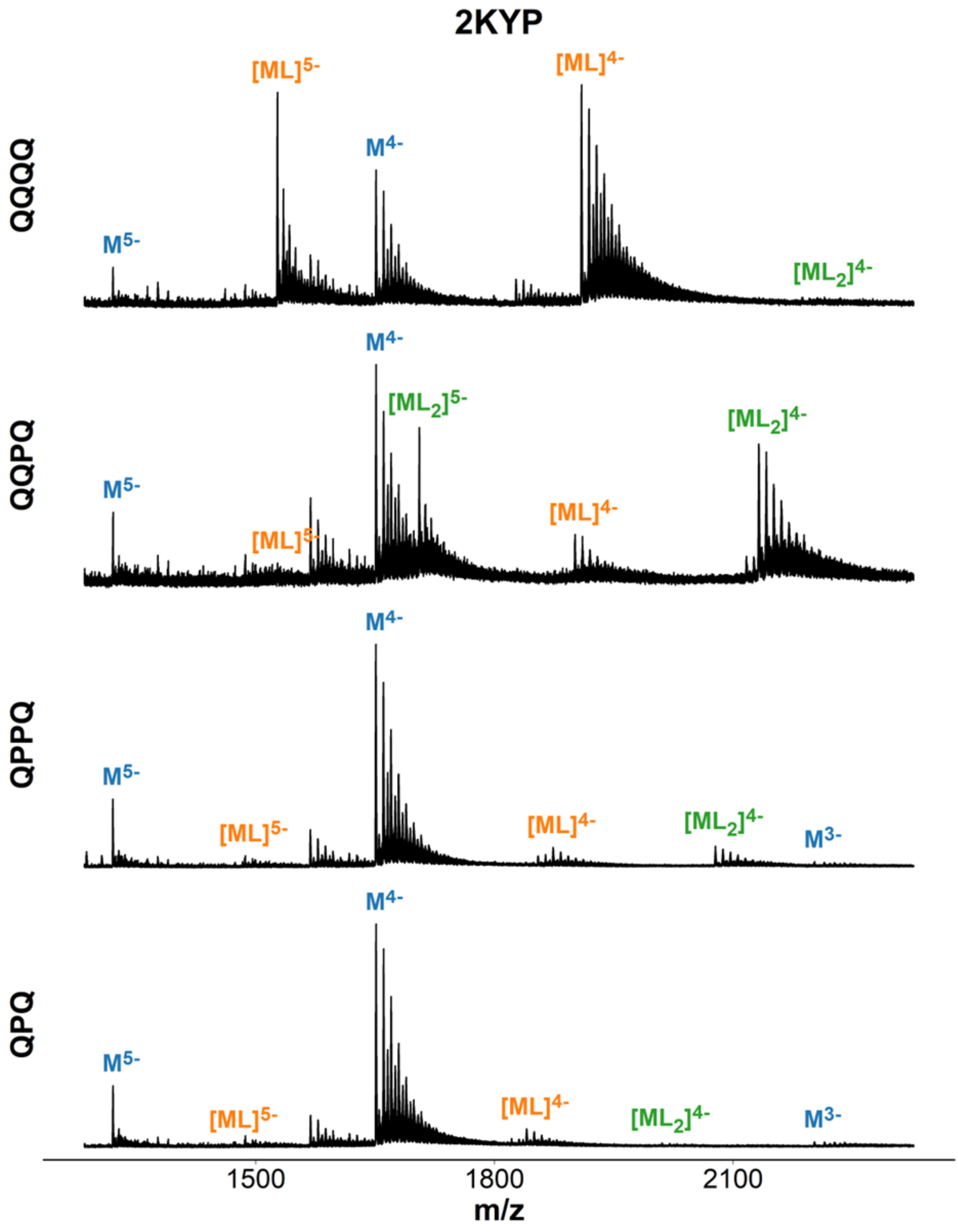

*Figure S41. Mass spectra of 2KYP (dC**GGG**C**GGG**C**G**CTA**GGG**A**GGG**T) in presence of ligand. Samples contain 10 µM DNA, 20 µM ligand, 0.5 mM KCl, 100 mM TMAA (pH 6.8).*

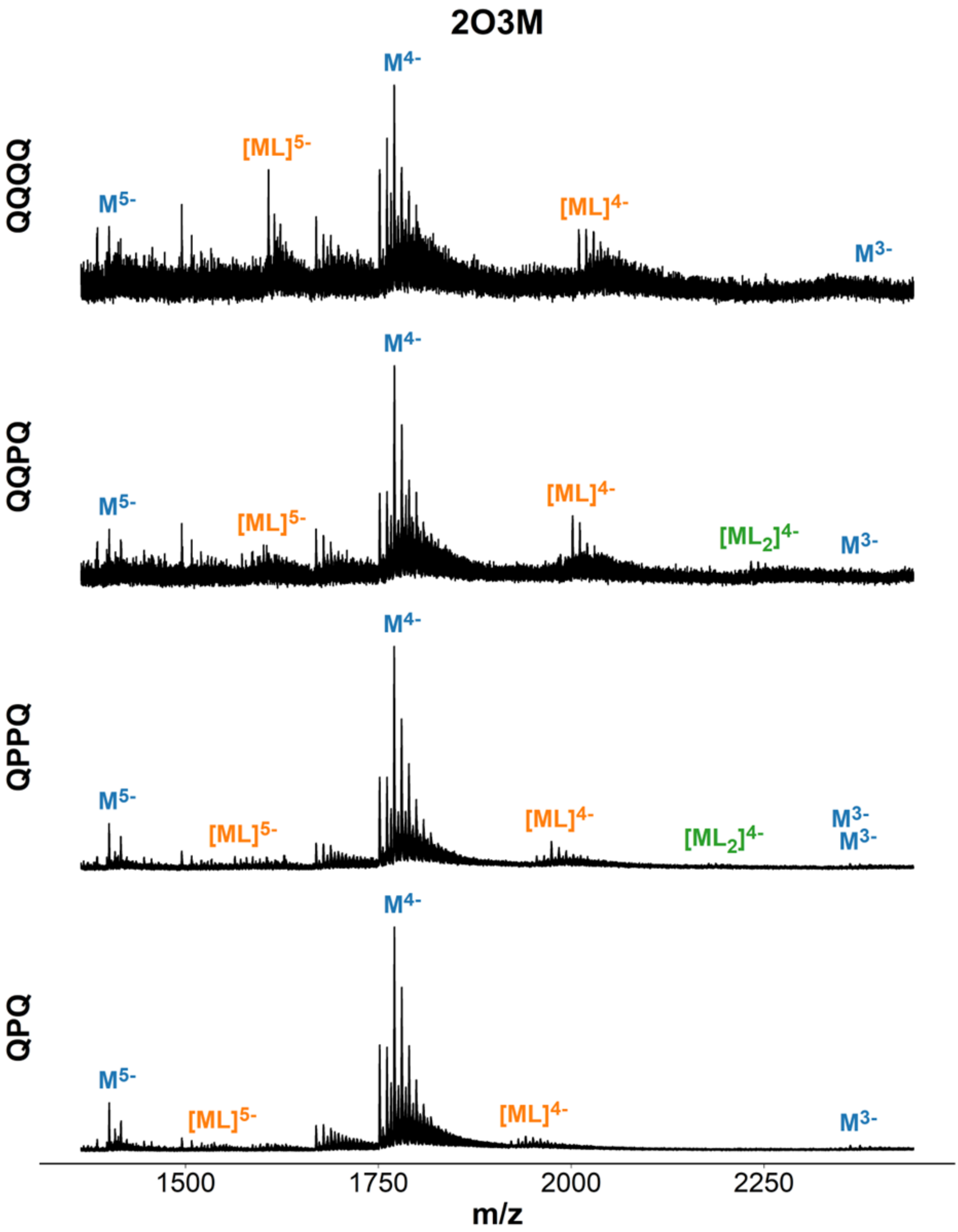

*Figure S42. Mass spectra of 2O3M (dA**GGG**A**GGG**C**G**CT**GGG**A**GG**A**GGG**) in presence of ligand. Samples contain 10 µM DNA, 20 µM ligand, 0.5 mM KCl, 100 mM TMAA (pH 6.8).*

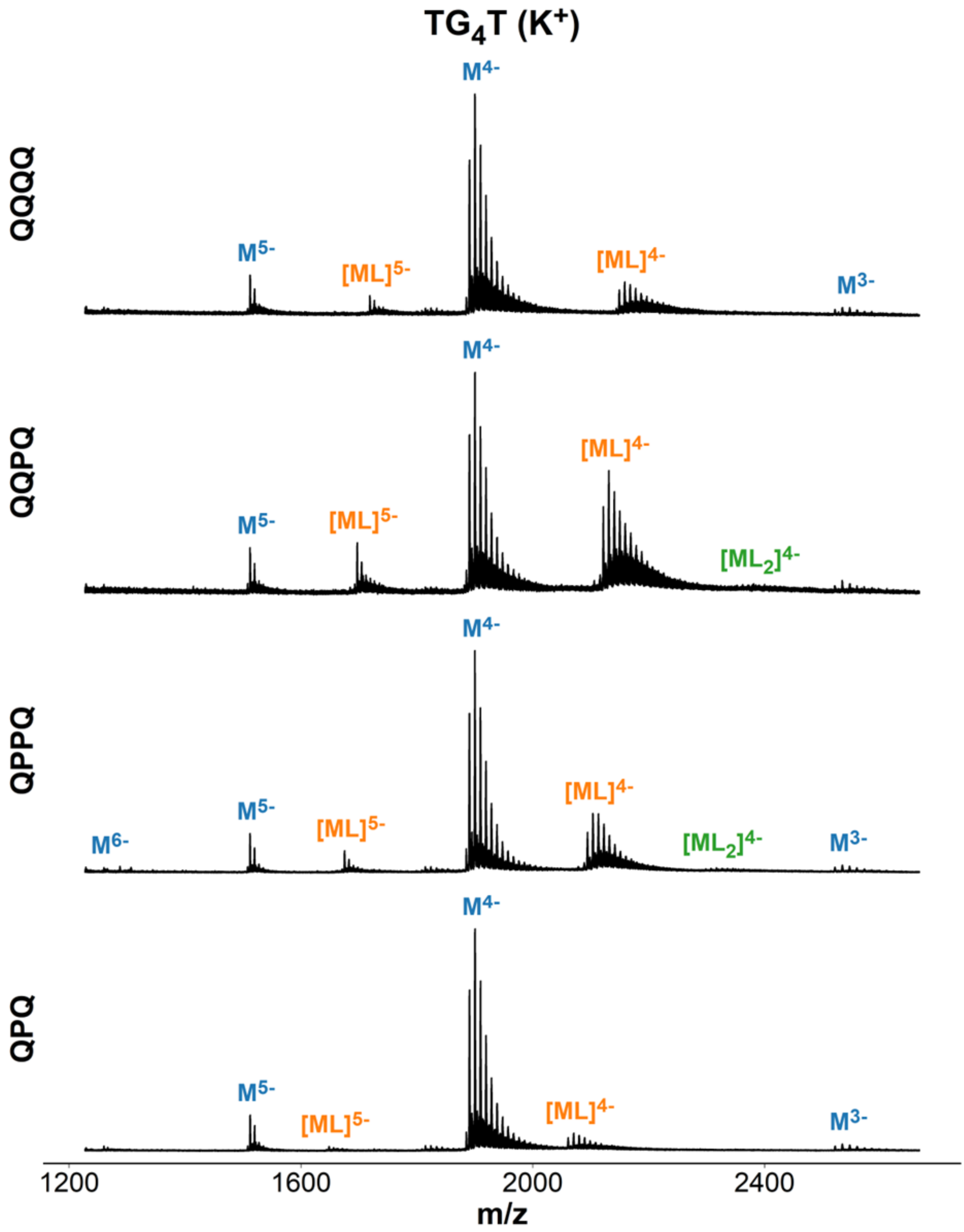

*Figure S43. Mass spectra of TG4T ([dT**GGGG**T)$_4$) in presence of ligand. Samples contain 40 µM DNA, 20 µM ligand, 0.5 mM KCl, 100 mM TMAA (pH 6.8).*

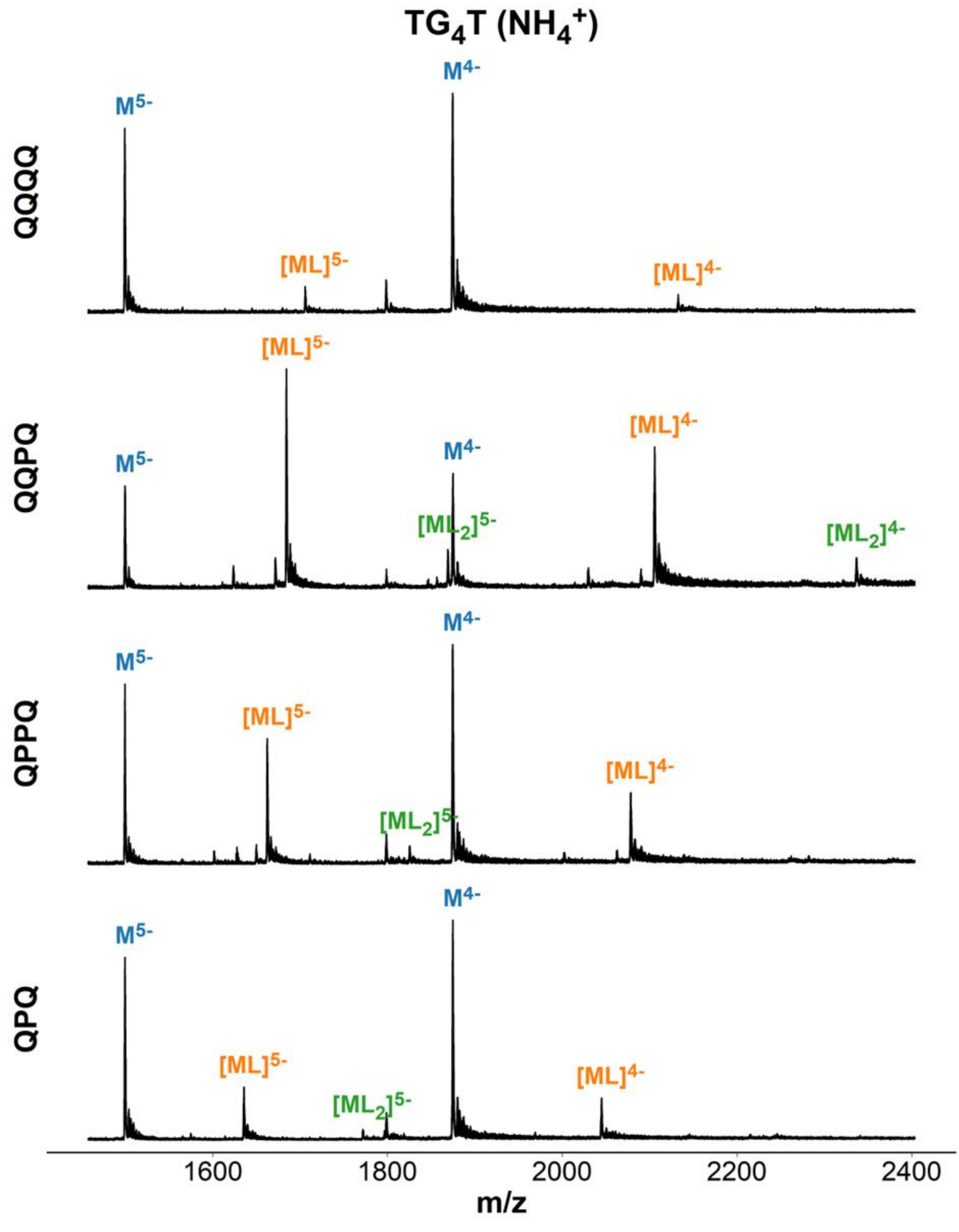

*Figure S44. Mass spectra of TG4T ([dT**GGGG**T)$_4$) in presence of ligand. Samples contain 40 µM DNA, 20 µM ligand, 150 mM ammonium acetate (pH 6.8).*

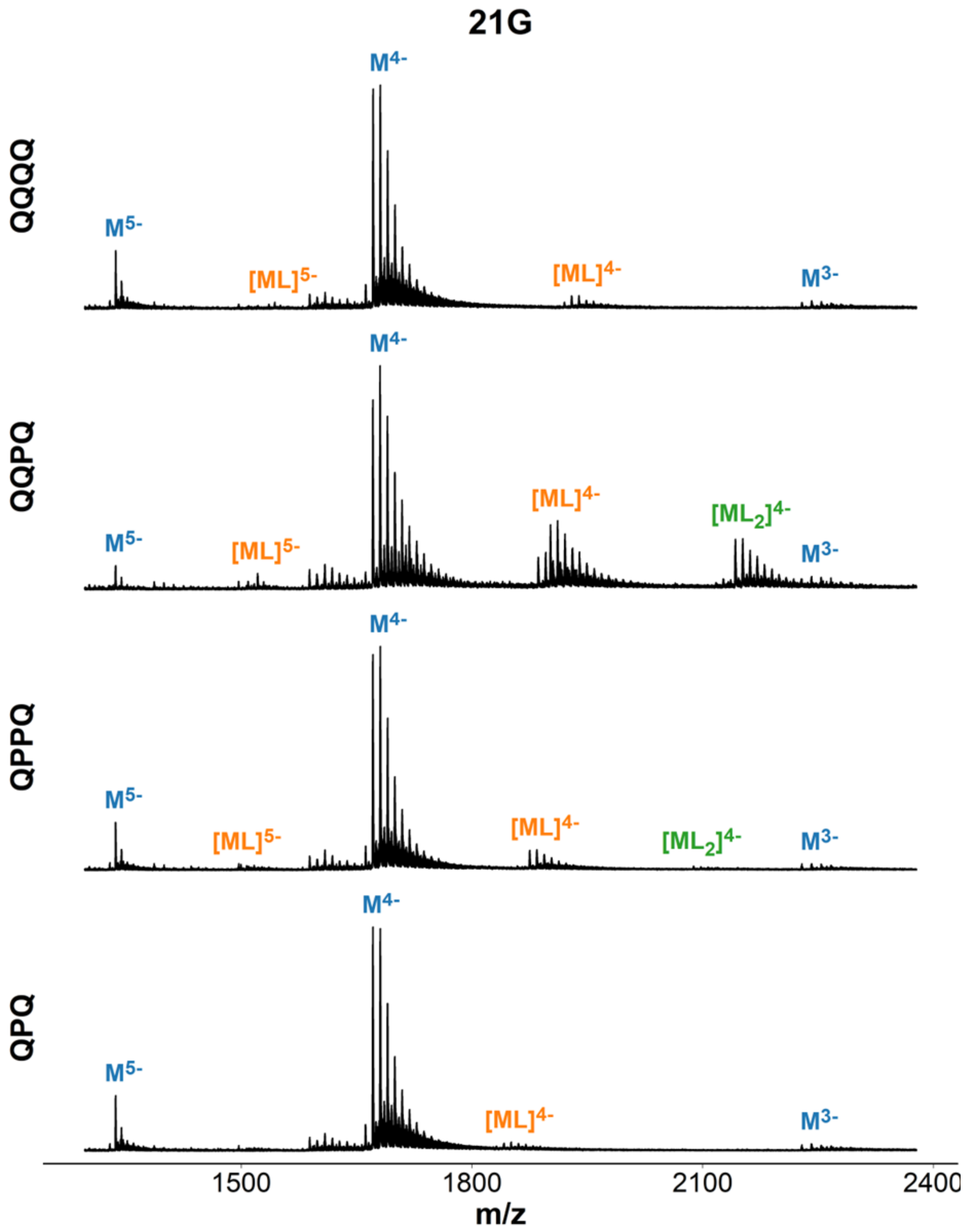

*Figure S45. Mass spectra of 21G (d**GGG**TTA**GGG**TTA**GGG**TTA**GGG**) in presence of ligand. Samples contain 10 µM DNA, 20 µM ligand, 0.5 mM KCl, 100 mM TMAA (pH 6.8).*

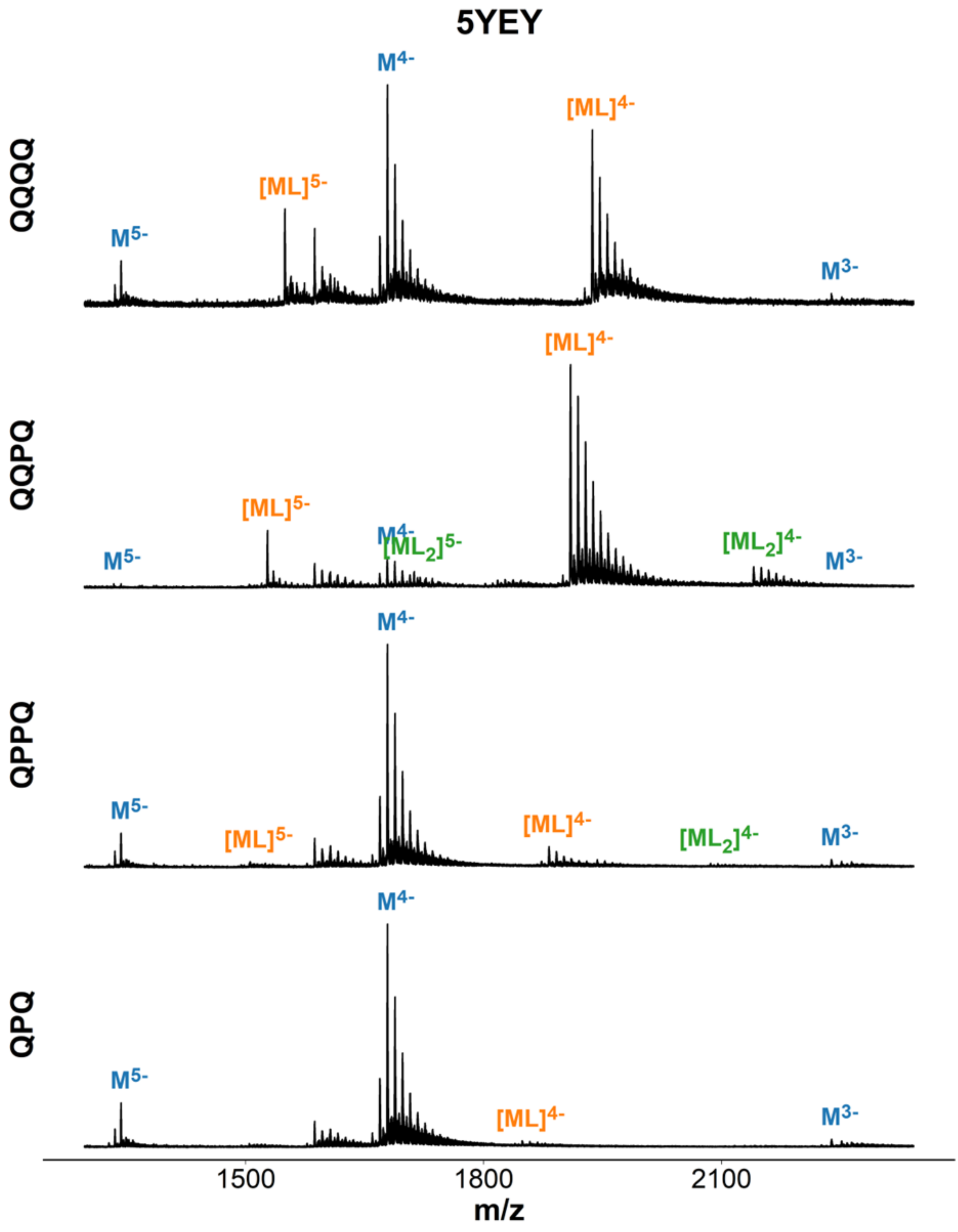

*Figure S46. Mass spectra of 5YEY (d**GGG**TTA**GGG**TTA**GGG**TTT**GGG**) in presence of ligand. Samples contain 10 µM DNA, 20 µM ligand, 0.5 mM KCl, 100 mM TMAA (pH 6.8).*

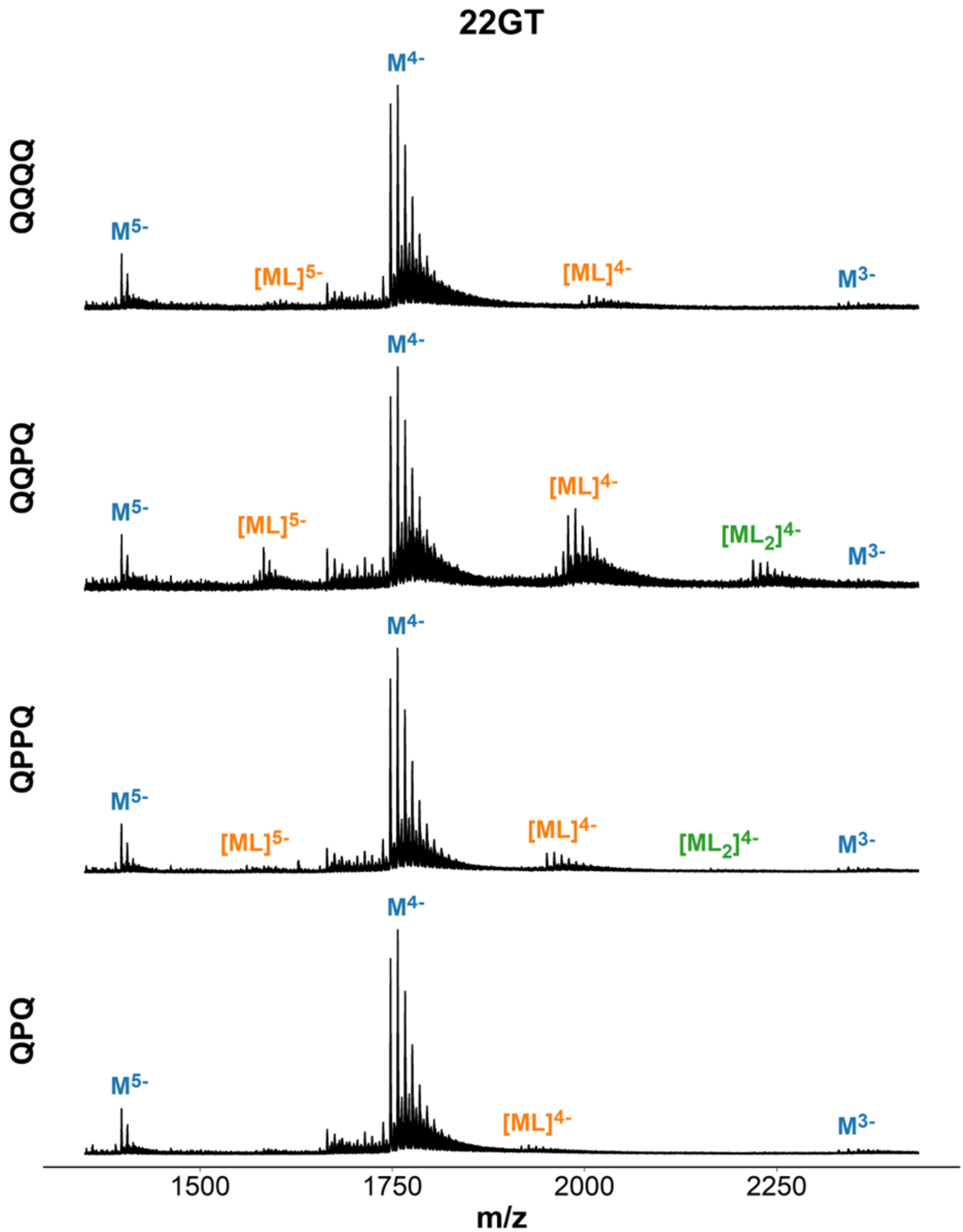

*Figure S47. Mass spectra of 22GT (d**GGG**TTA**GGG**TTA**GGG**TTA**GGG**T) in presence of ligand. Samples contain 10 µM DNA, 20 µM ligand, 0.5 mM KCl, 100 mM TMAA (pH 6.8).*

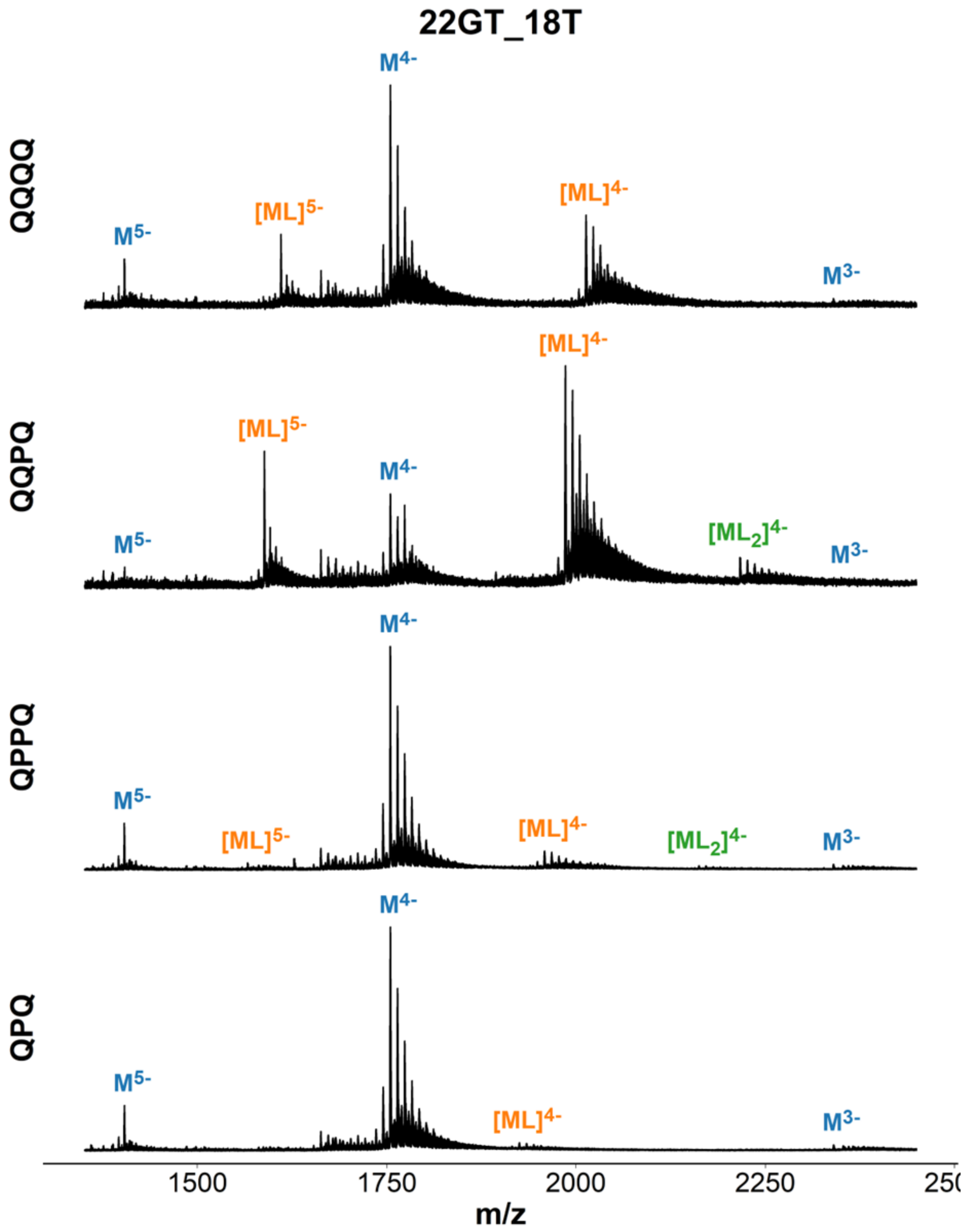

*Figure S48. Mass spectra of 22GT-18T (d**GGG**TTA**GGG**TTA**GGG**TTT**GGG**T) in presence of ligand. Samples contain 10 µM DNA, 20 µM ligand, 0.5 mM KCl, 100 mM TMAA (pH 6.8).*

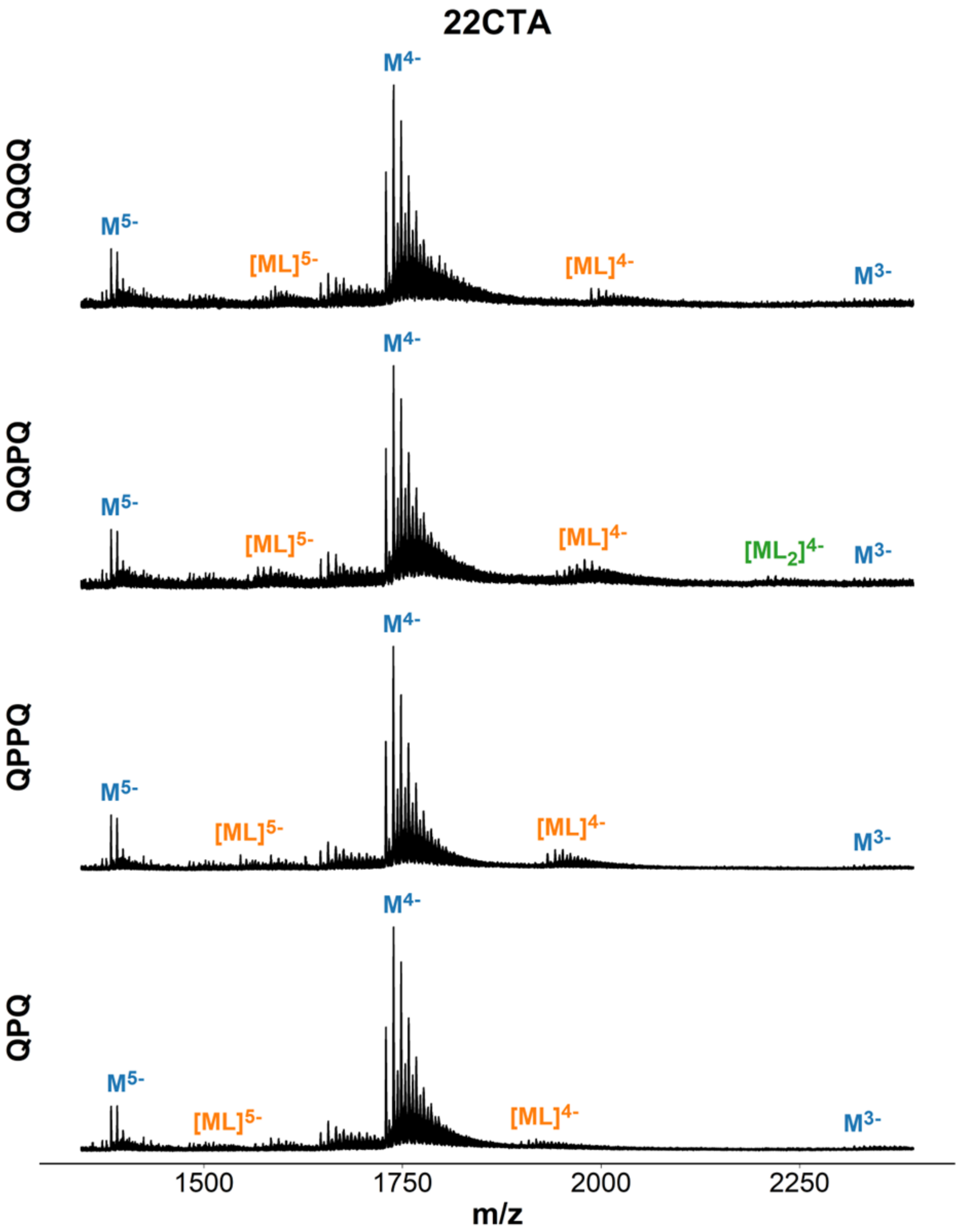

*Figure S49. Mass spectra of 22CTA (dA**GGG**CTA**GGG**CTA**GGG**CTA**GGG**) in presence of ligand. Samples contain 10 µM DNA, 20 µM ligand, 0.5 mM KCl, 100 mM TMAA (pH 6.8).*

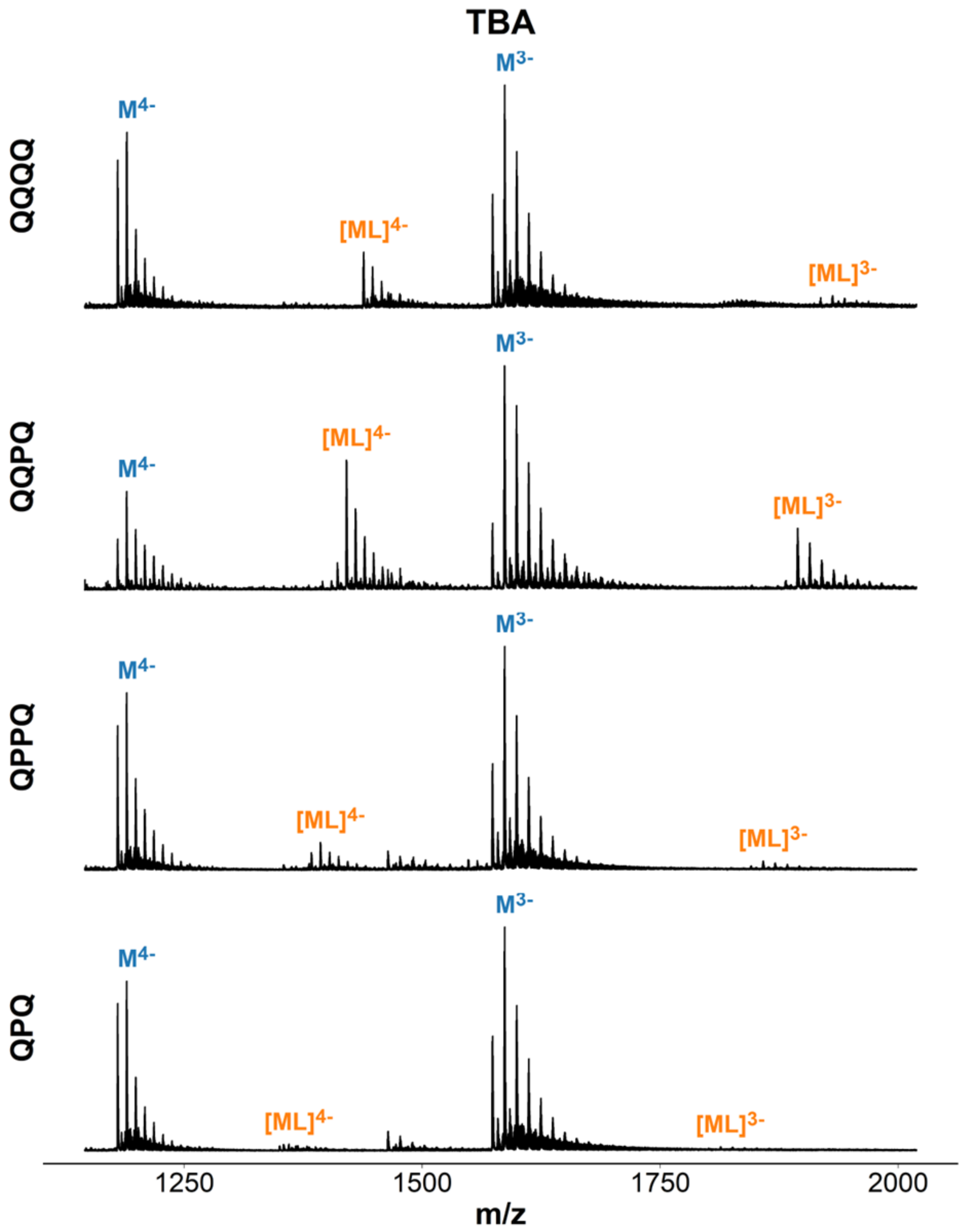

*Figure S50. Mass spectra of TBA (d**GG**TT**GG**T**G**T**GG**TT**GG**) in presence of ligand. Samples contain 10 µM DNA, 20 µM ligand, 0.5 mM KCl, 100 mM TMAA (pH 6.8).*

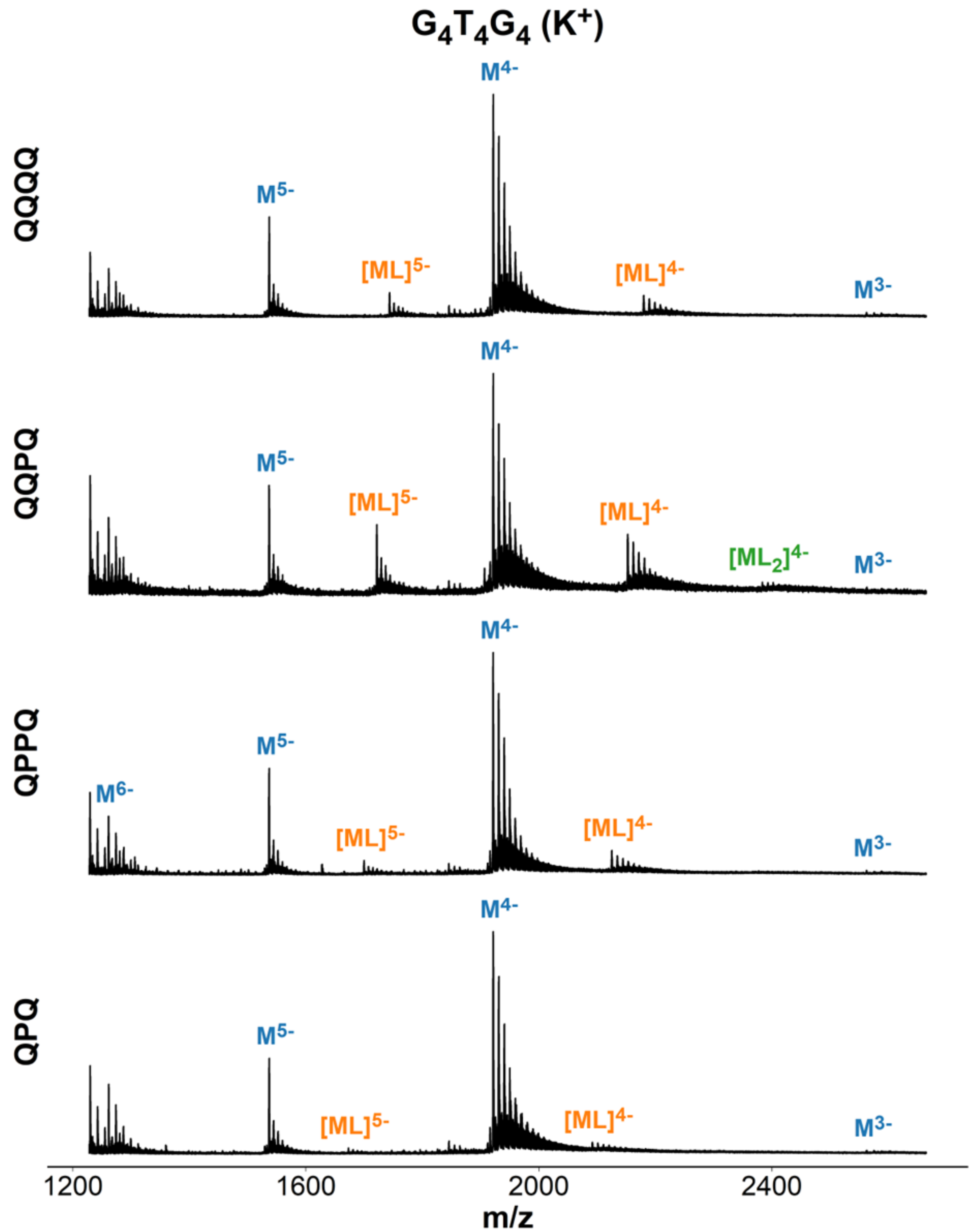

*Figure S51. Mass spectra of G4T4G4 ([d**GGGG**TTTT**GGGG**]2) in presence of ligand. Samples contain 20 µM DNA, 20 µM ligand, 0.5 mM KCl, 100 mM TMAA (pH 6.8).*

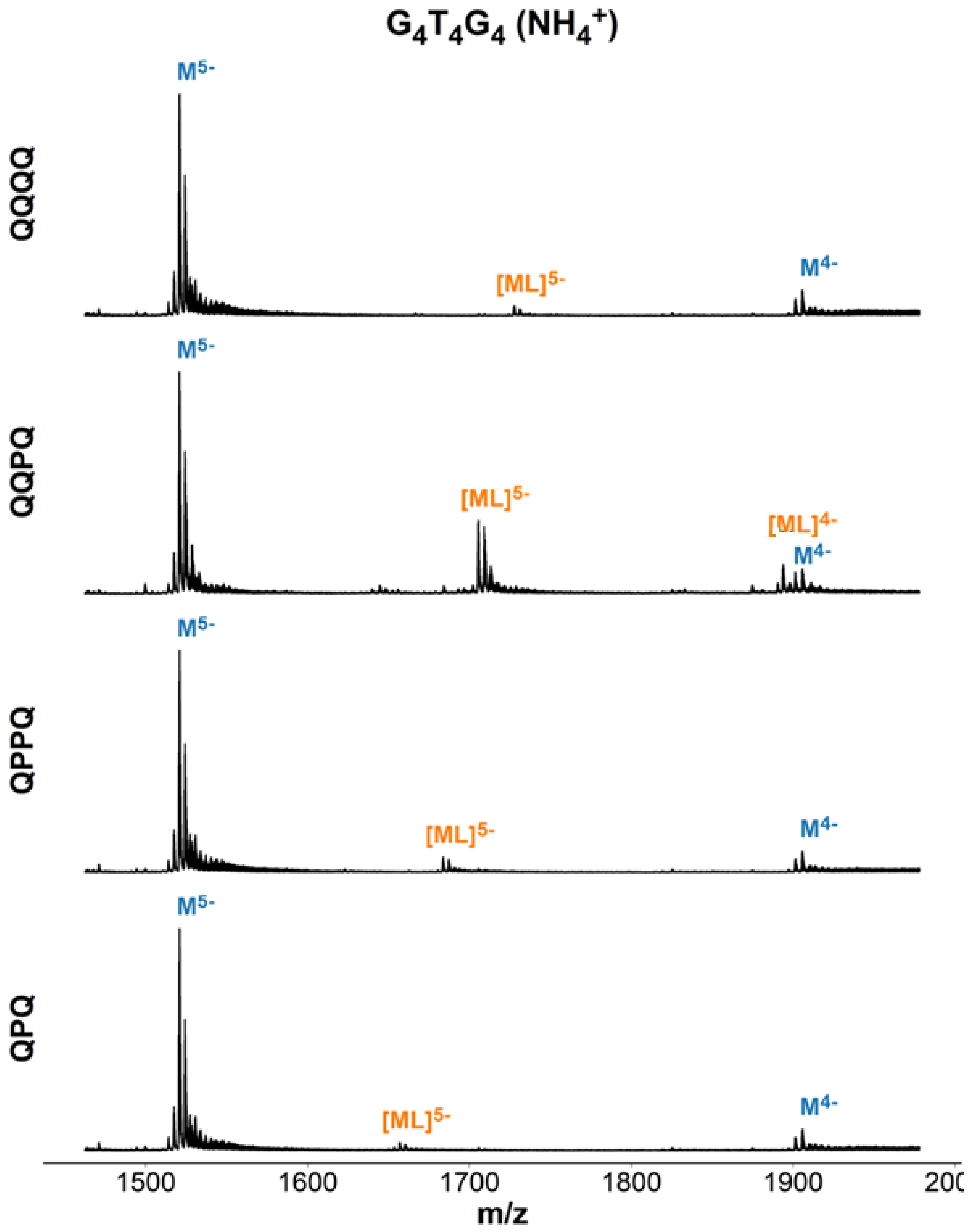

*Figure S52. Mass spectra of G4T4G4 ([d**GGGG**TTTT**GGGG**]₂) in presence of ligand. Samples contain 20 µM DNA, 20 µM ligand, 150 mM ammonium acetate (pH 6.8).*

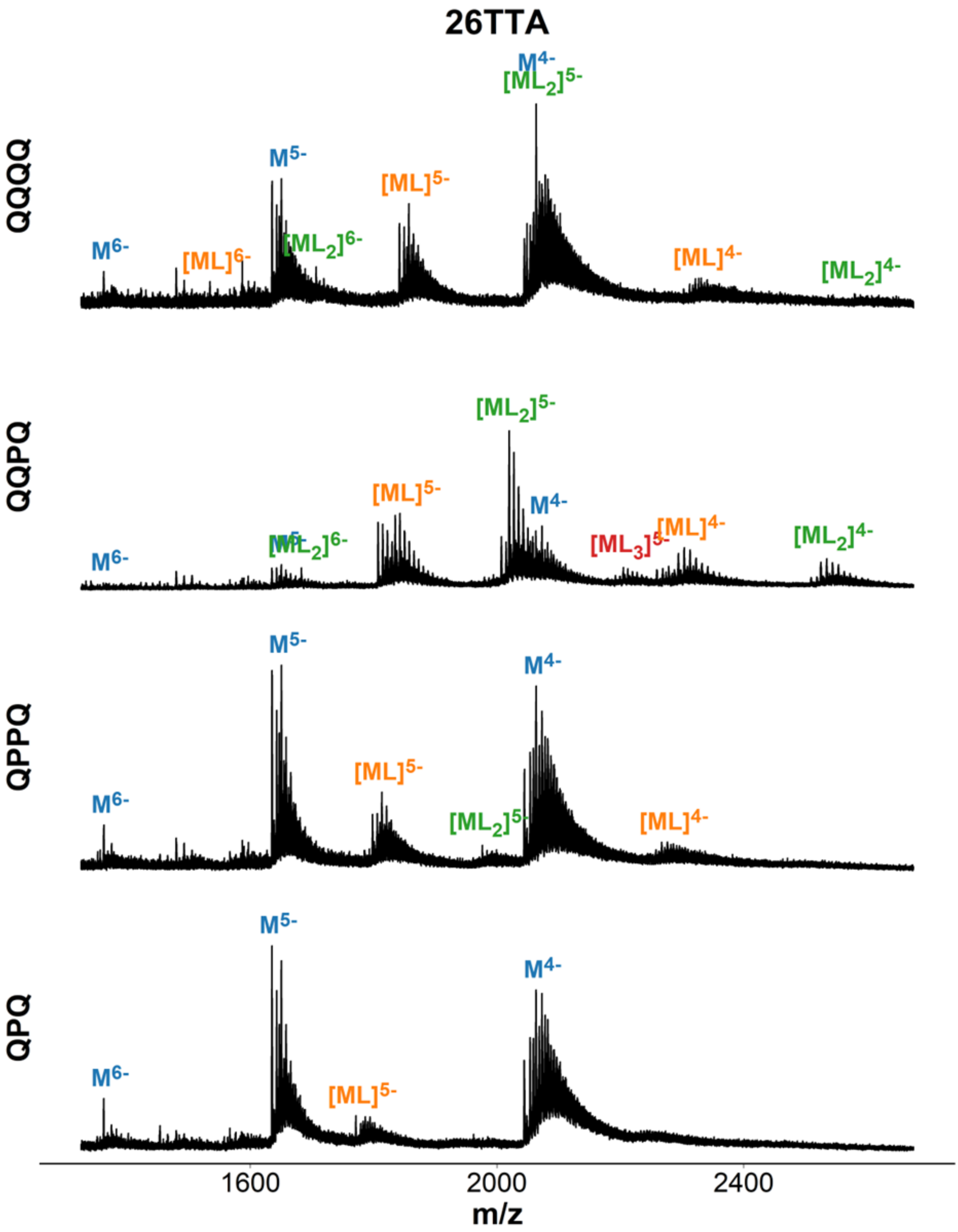

*Figure S53. Mass spectra of 26TTA (dTTA**GGG**TTA**GGG**TTA**GGG**TTA**GGG**TT) in presence of ligand. Samples contain 10 µM DNA, 20 µM ligand, 0.5 mM KCl, 100 mM TMAA (pH 6.8).*

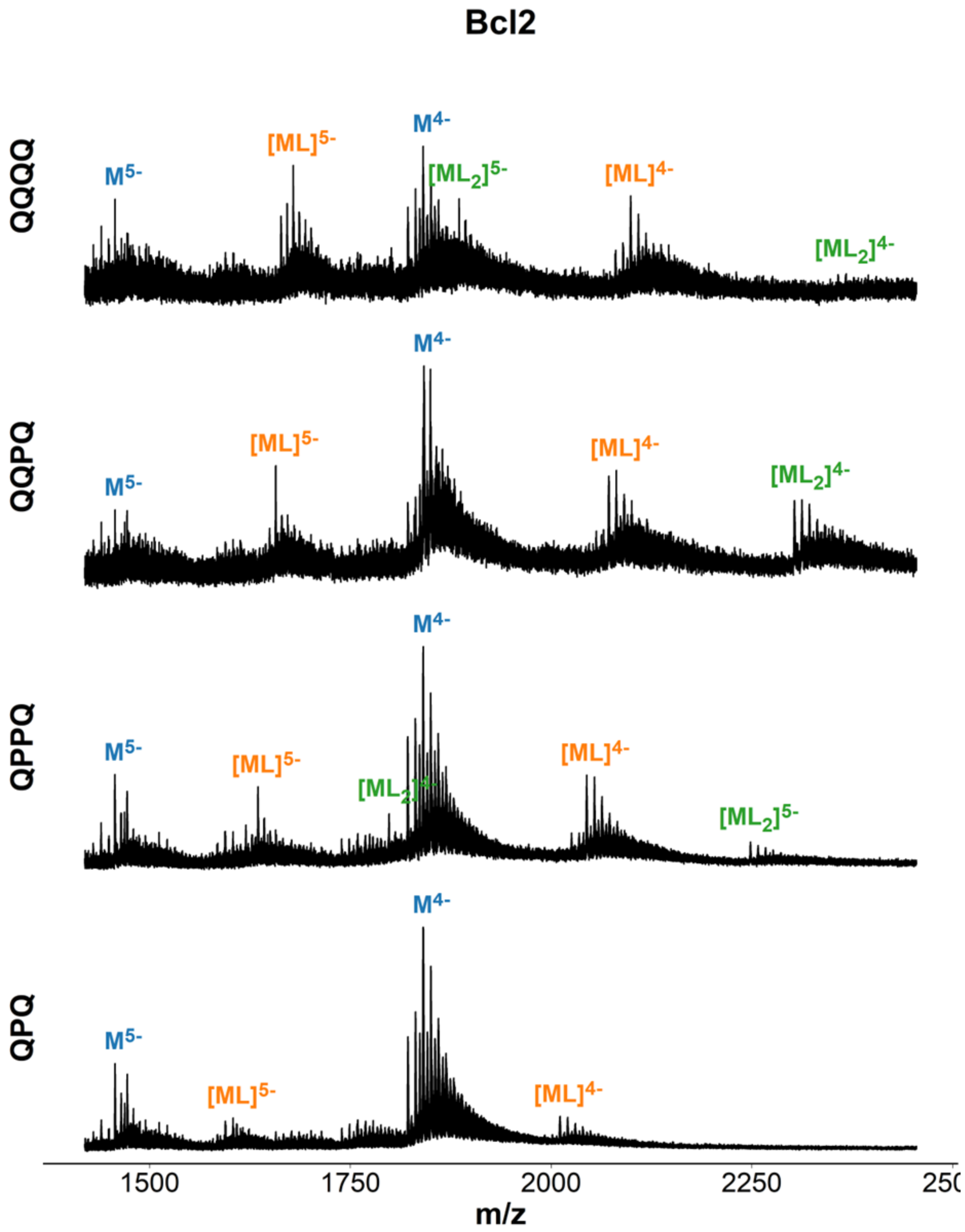

*Figure S54. Mass spectra of bcl2 (d**GGG**C**G**C**GGG**A**GG**AATT**GGG**C**GGG**) in presence of ligand. Samples contain 10 µM DNA, 20 µM ligand, 0.5 mM KCl, 100 mM TMAA (pH 6.8).*

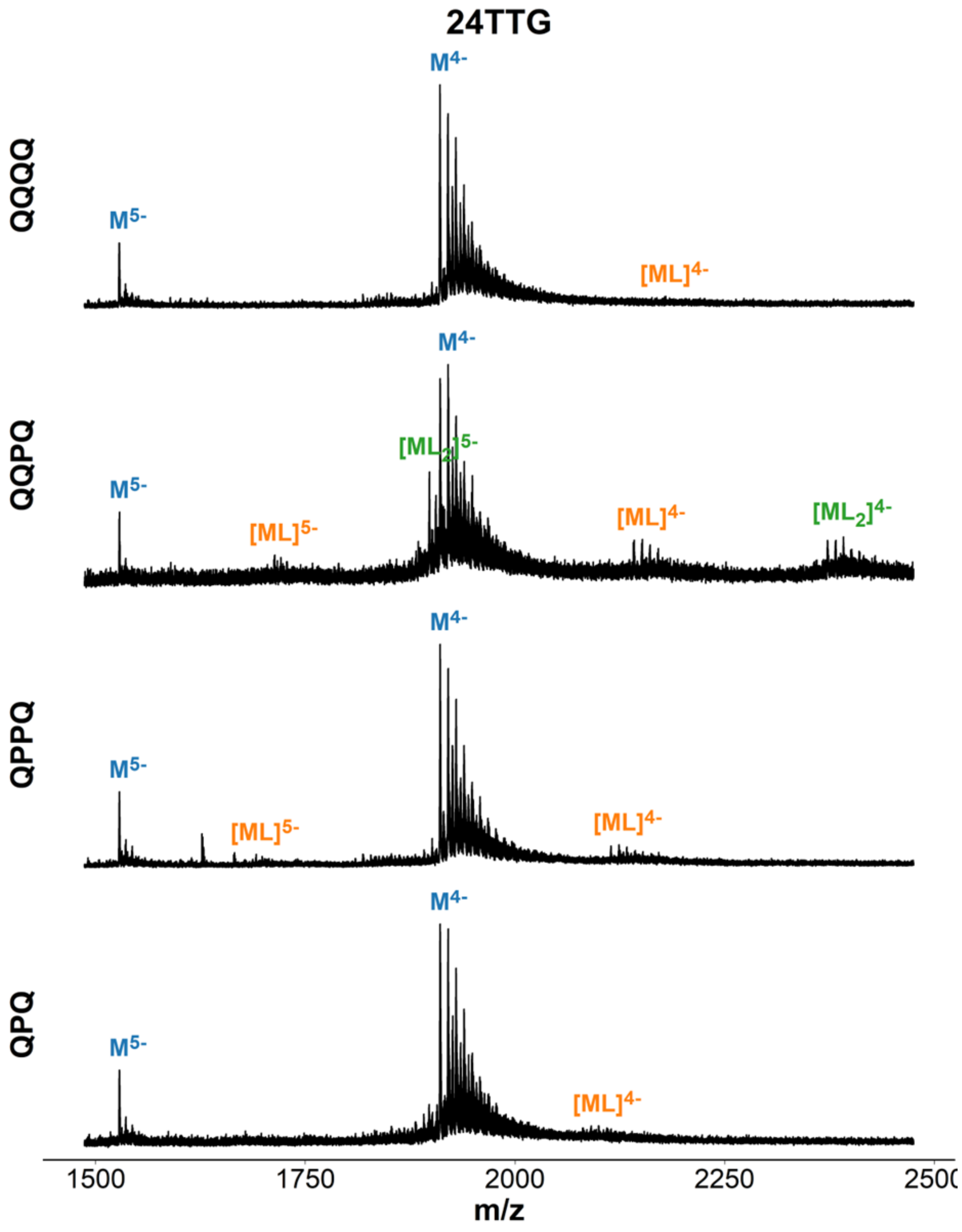

*Figure S55. Mass spectra of 24TTG (dTT**GGG**TTA**GGG**TTA**GGG**TTA**GGG**A) in presence of ligand. Samples contain 10 µM DNA, 20 µM ligand, 0.5 mM KCl, 100 mM TMAA (pH 6.8).*

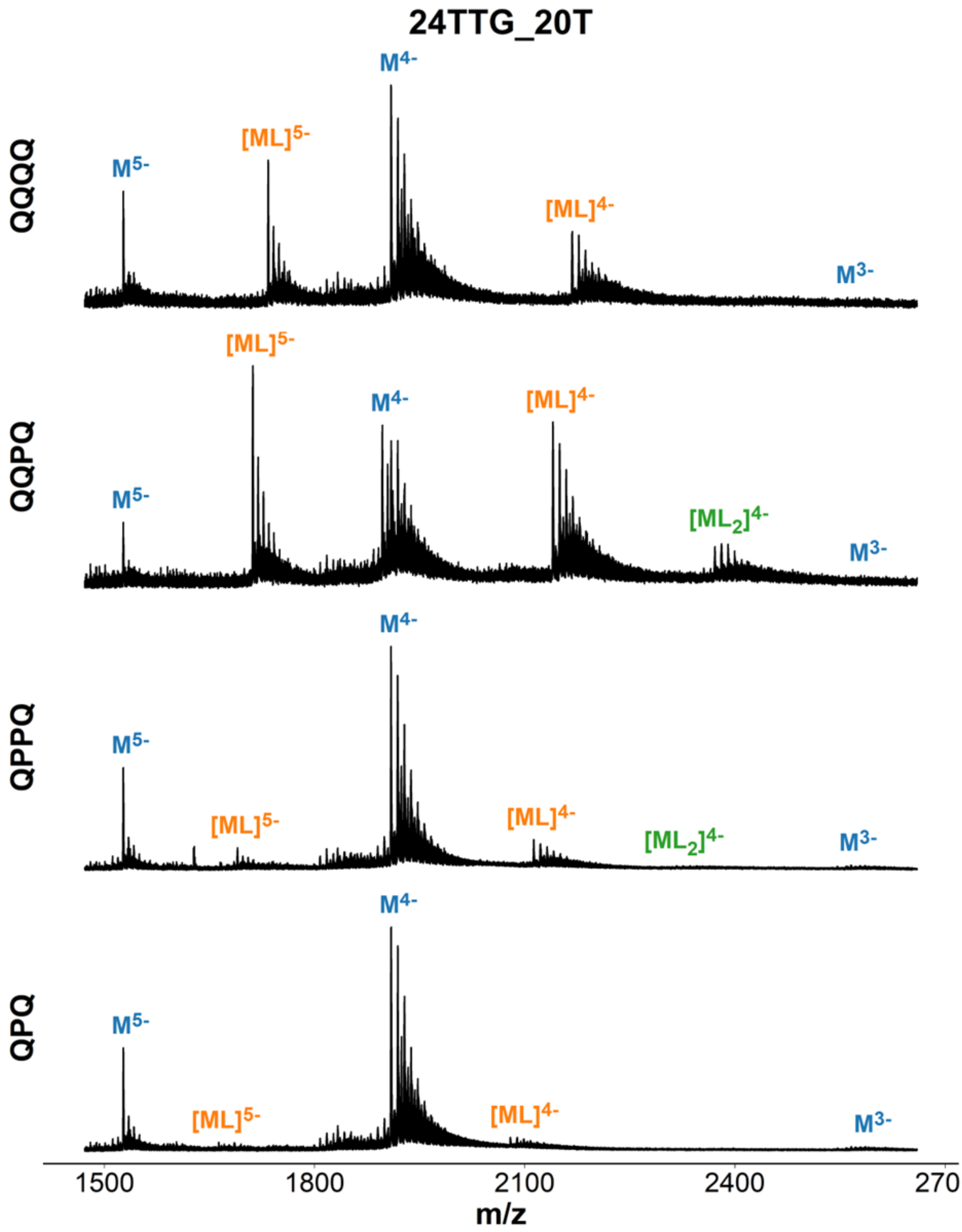

*Figure S56. Mass spectra of 24TTG-20T (dTT**GGG**TTA**GGG**TTA**GGG**TTT**GGG**A) in presence of ligand. Samples contain 10 µM DNA, 20 µM ligand, 0.5 mM KCl, 100 mM TMAA (pH 6.8).*

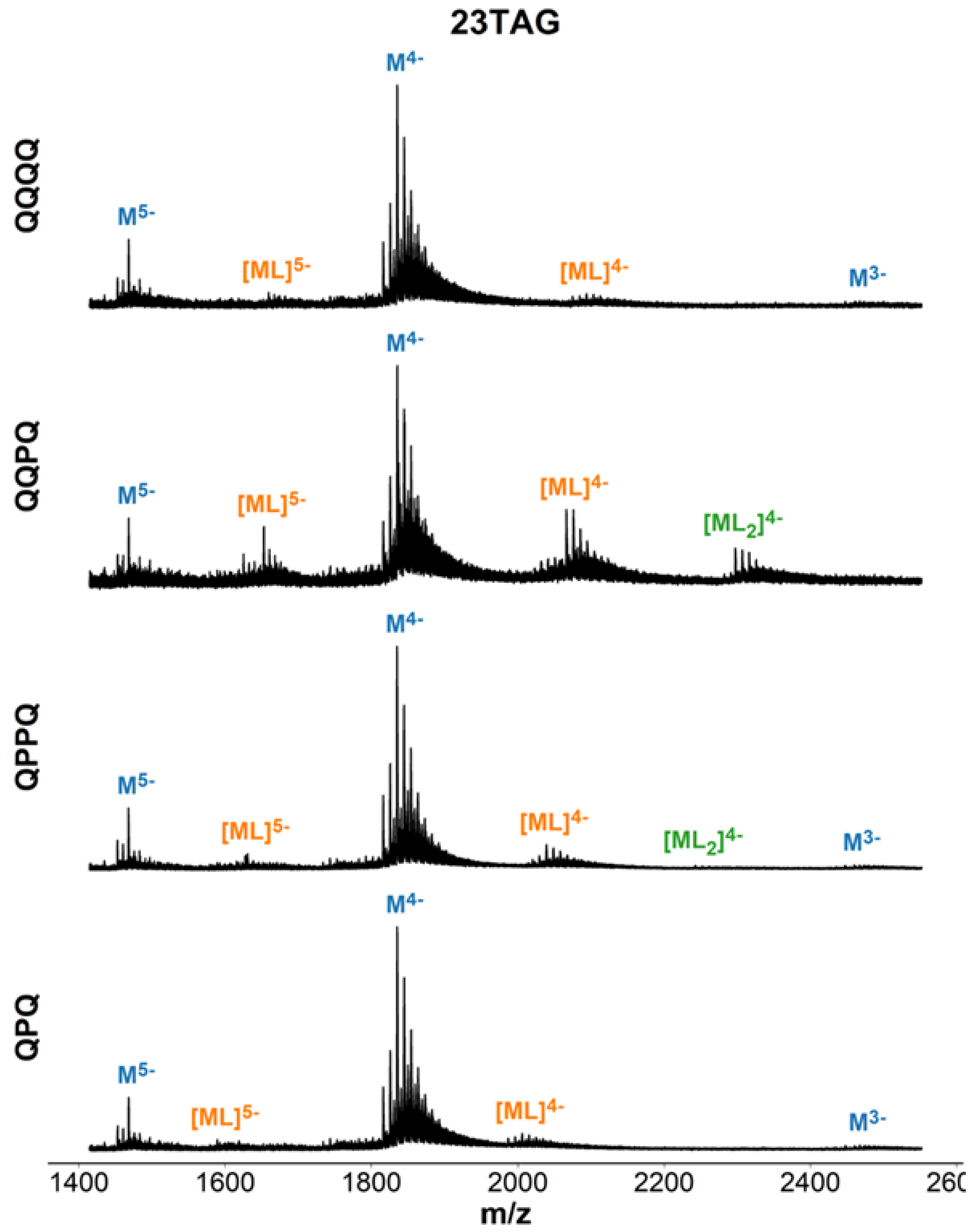

*Figure S57. Mass spectra of 23TAG (dTA**GGG**TTA**GGG**TTA**GGG**TTA**GGG**) in presence of ligand. Samples contain 10 µM DNA, 20 µM ligand, 0.5 mM KCl, 100 mM TMAA (pH 6.8).*

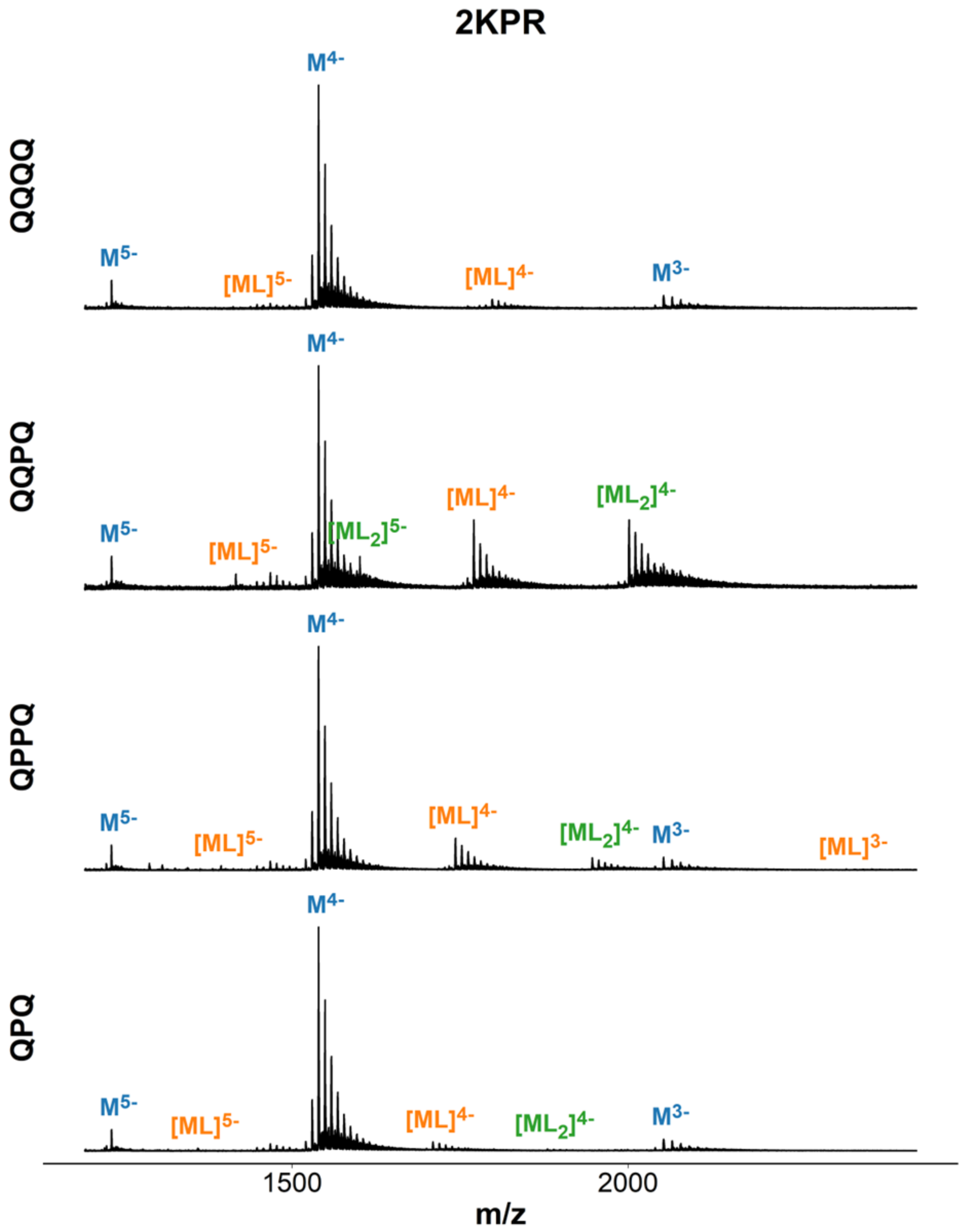

*Figure S58. Mass spectra of 2KPR (d**GGG**T**GGGG**AA**GGGG**T**GGG**T) in presence of ligand. Samples contain 10 µM DNA, 20 µM ligand, 0.5 mM KCl, 100 mM TMAA (pH 6.8).*

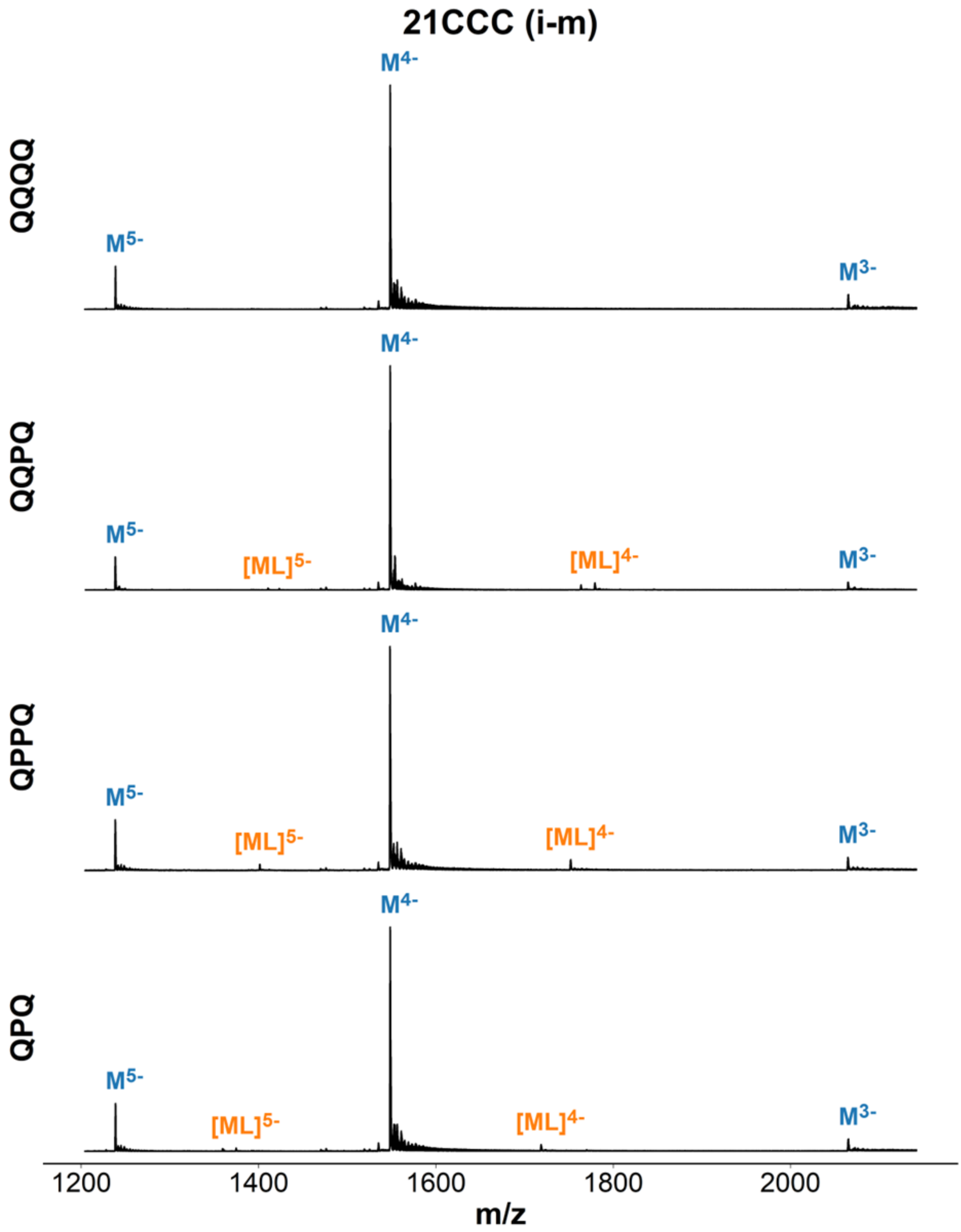

*Figure S59. Mass spectra of 21CCC (d**CCC**TAA**CCC**TAA**CCC**TAA**CCC**) in presence of ligand. Samples contain 10 µM DNA, 20 µM ligand, 150 mM ammonium acetate (pH 5.5).*

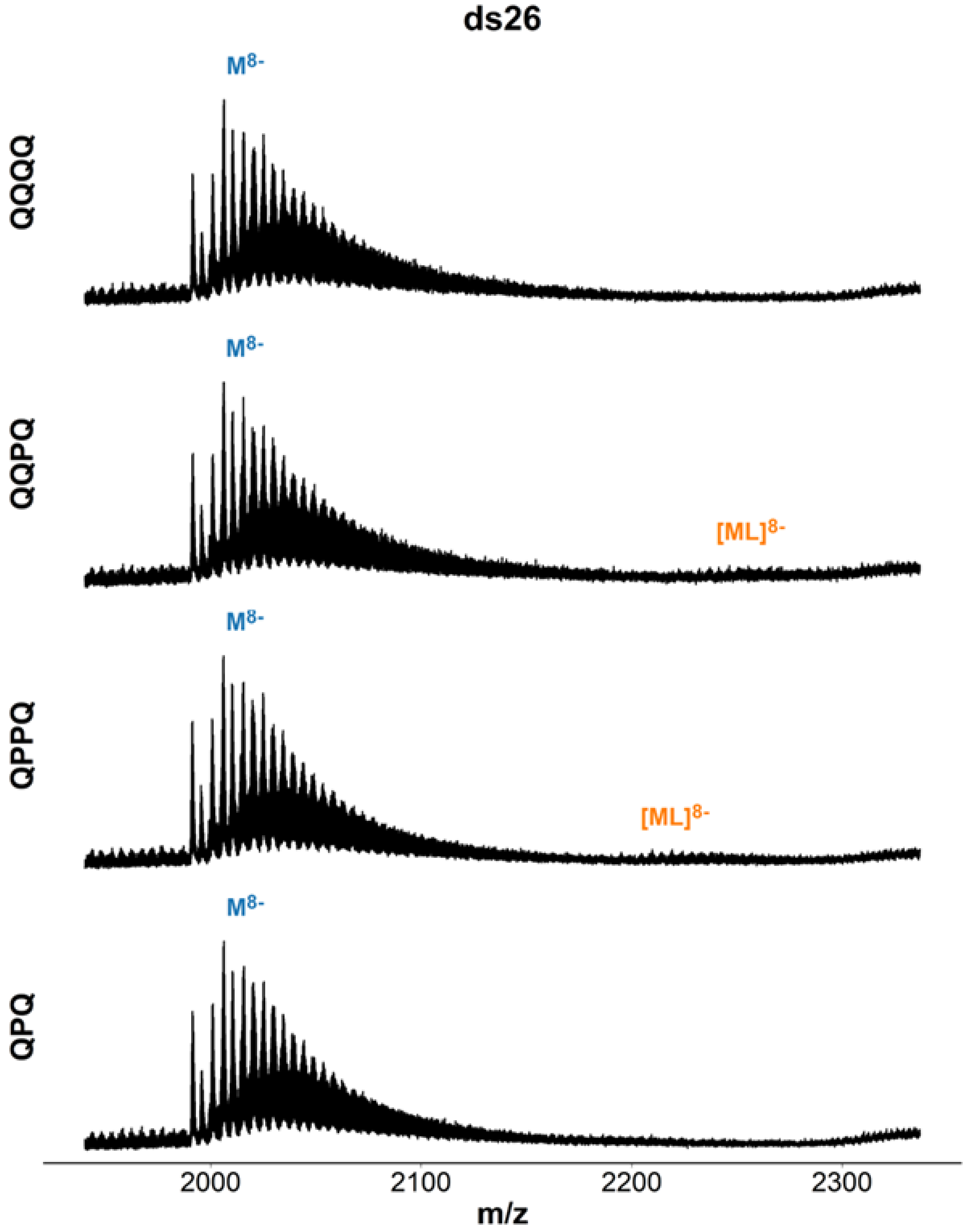

Figure S60. Mass spectra of ds26 ([dCAATCGGATCGAATTCGATCCGATTG]$_2$) in presence of ligand. Samples contain 20 µM DNA, 20 µM ligand, 0.5 mM KCl, 100 mM TMAA (pH 6.8).

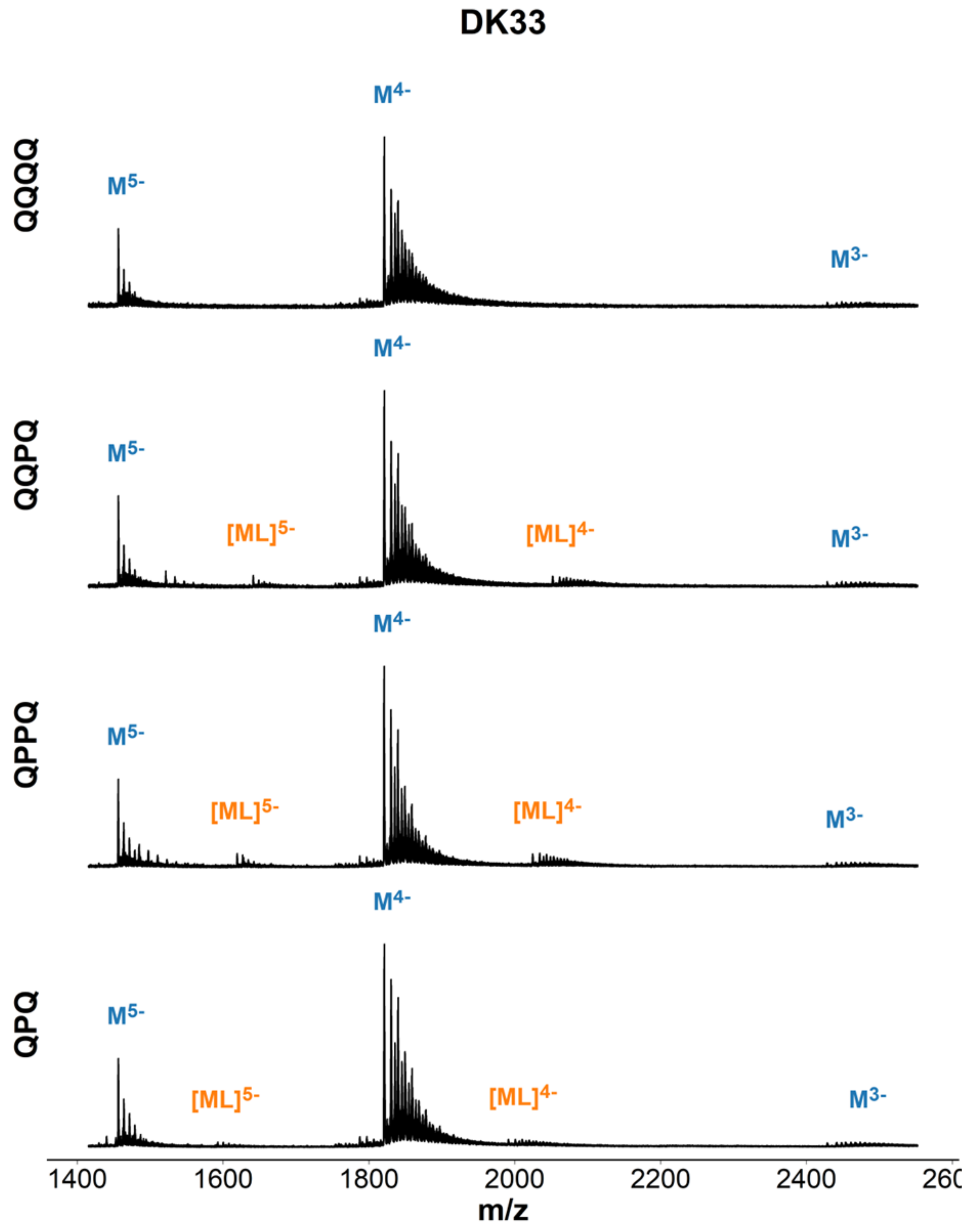

Figure S61. Mass spectra of DK-33 ([dCGTAAATTTACG]$_2$) in presence of ligand. Samples contain 20 µM DNA, 20 µM ligand, 0.5 mM KCl, 100 mM TMAA (pH 6.8).

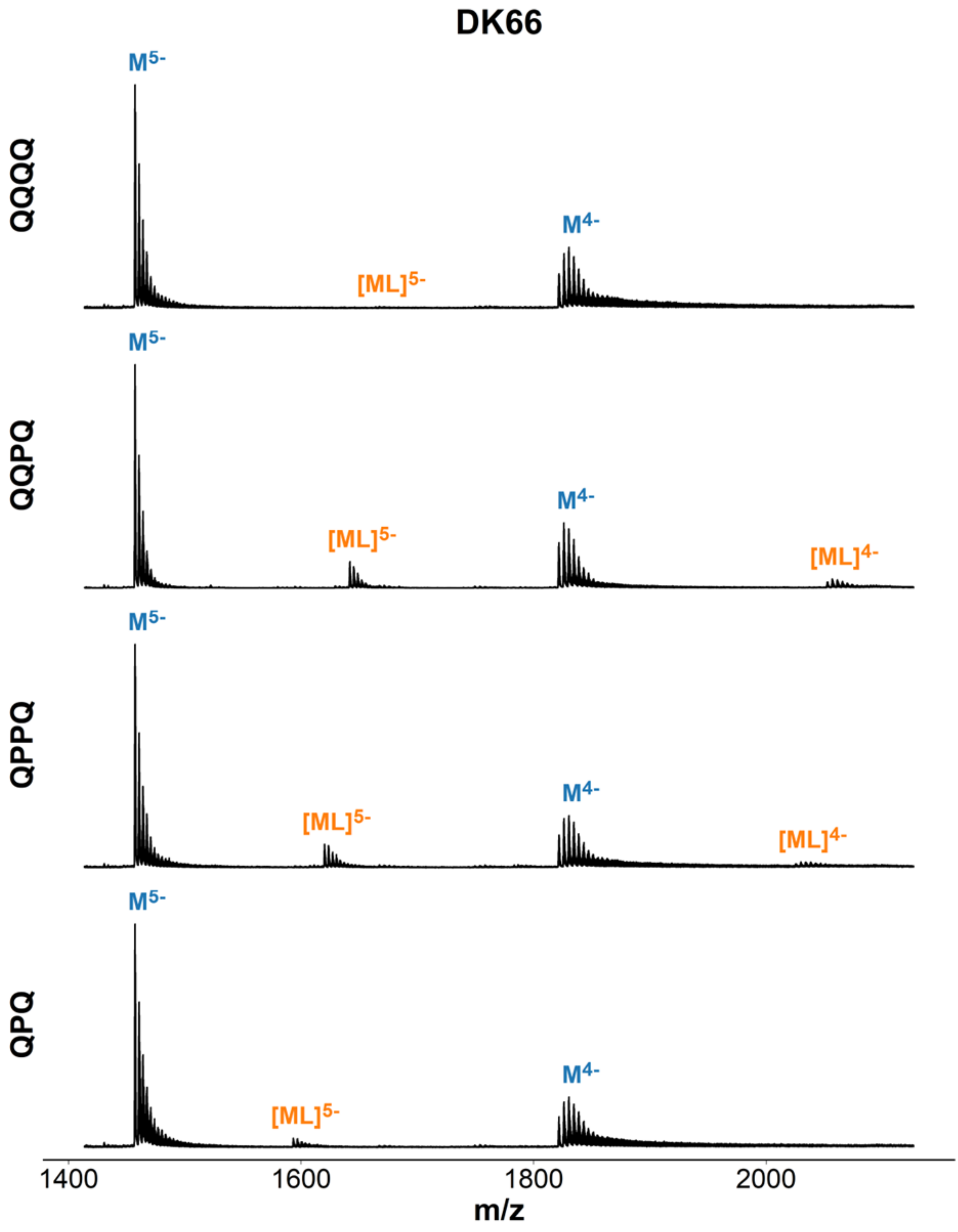

Figure S62. Mass spectra of DK-66 ([dCGCGAATTCGCG]$_2$) in presence of ligand. Samples contain 20 µM DNA, 20 µM ligand, 150 mM ammonium acetate (pH 6.8).

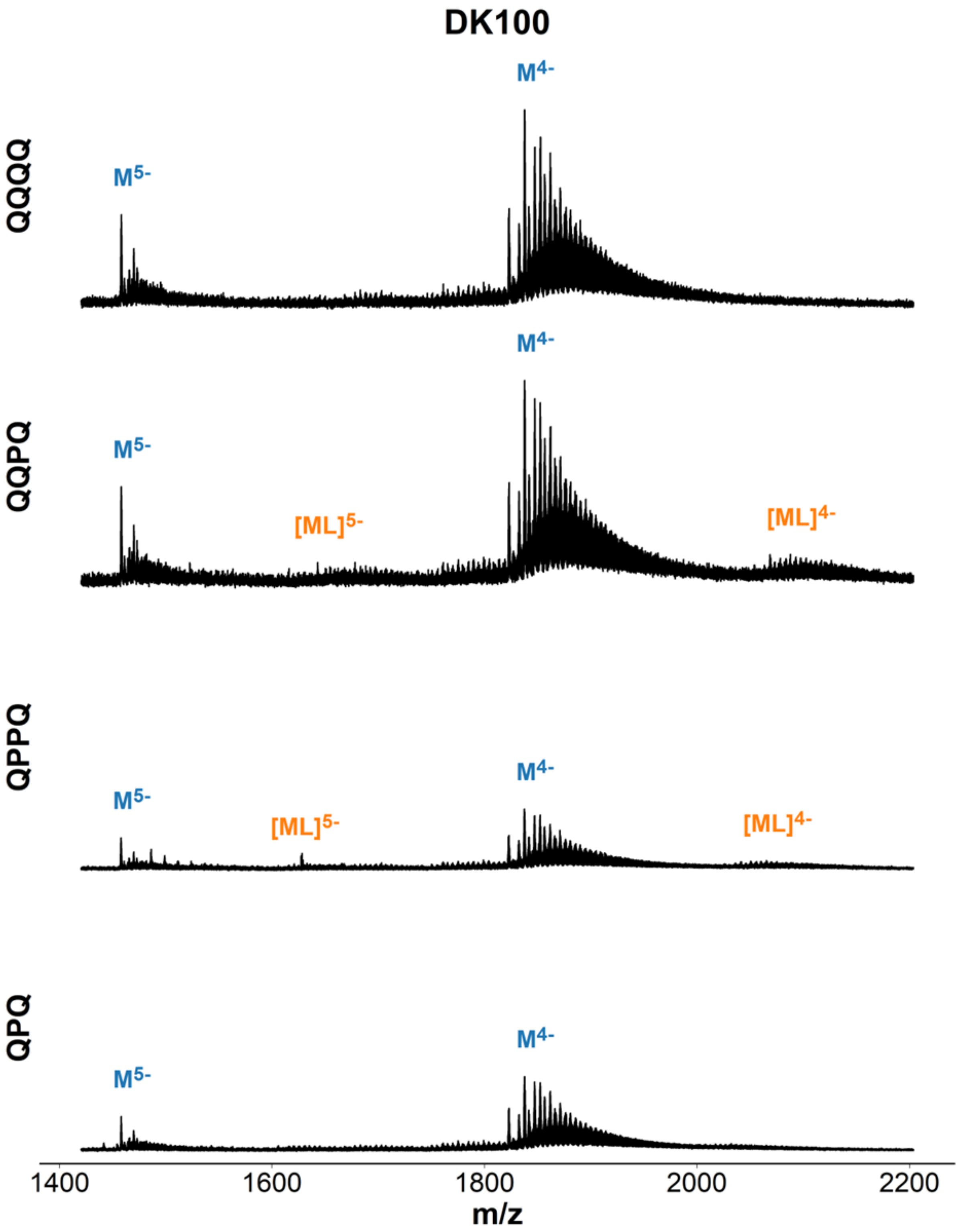

Figure S63. Mass spectra of DK-100 ([dCGCGGGCCCGCG]$_2$) in presence of ligand. Samples contain 20 µM DNA, 20 µM ligand, 0.5 mM KCl, 100 mM TMAA (pH 6.8).

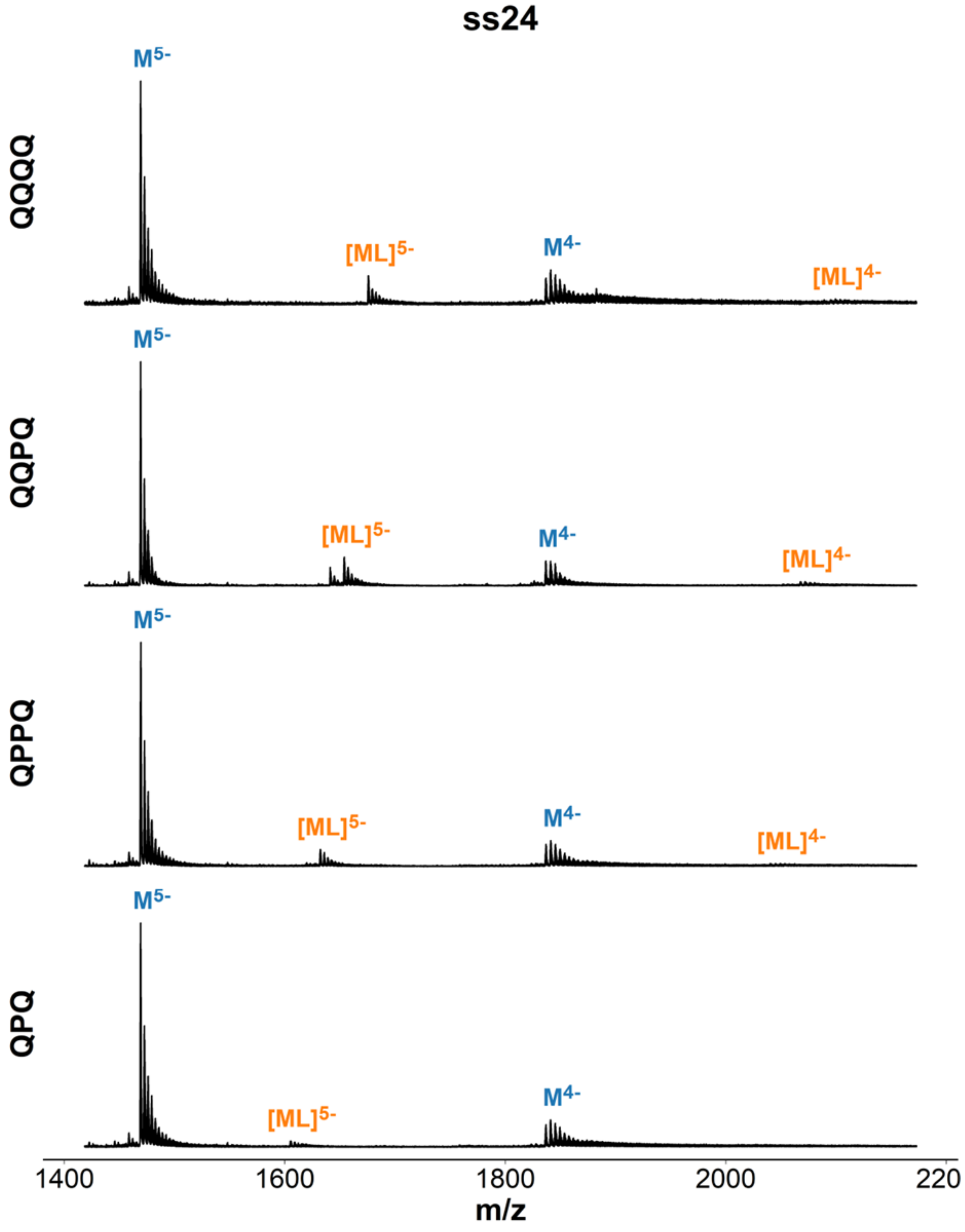

Figure S64. Mass spectra of ss24 (dTGCCATGCTACTGAGATGACGCTA) in presence of ligand. Samples contain 10 µM DNA, 20 µM ligand, 150 mM ammonium acetate (pH 6.8).

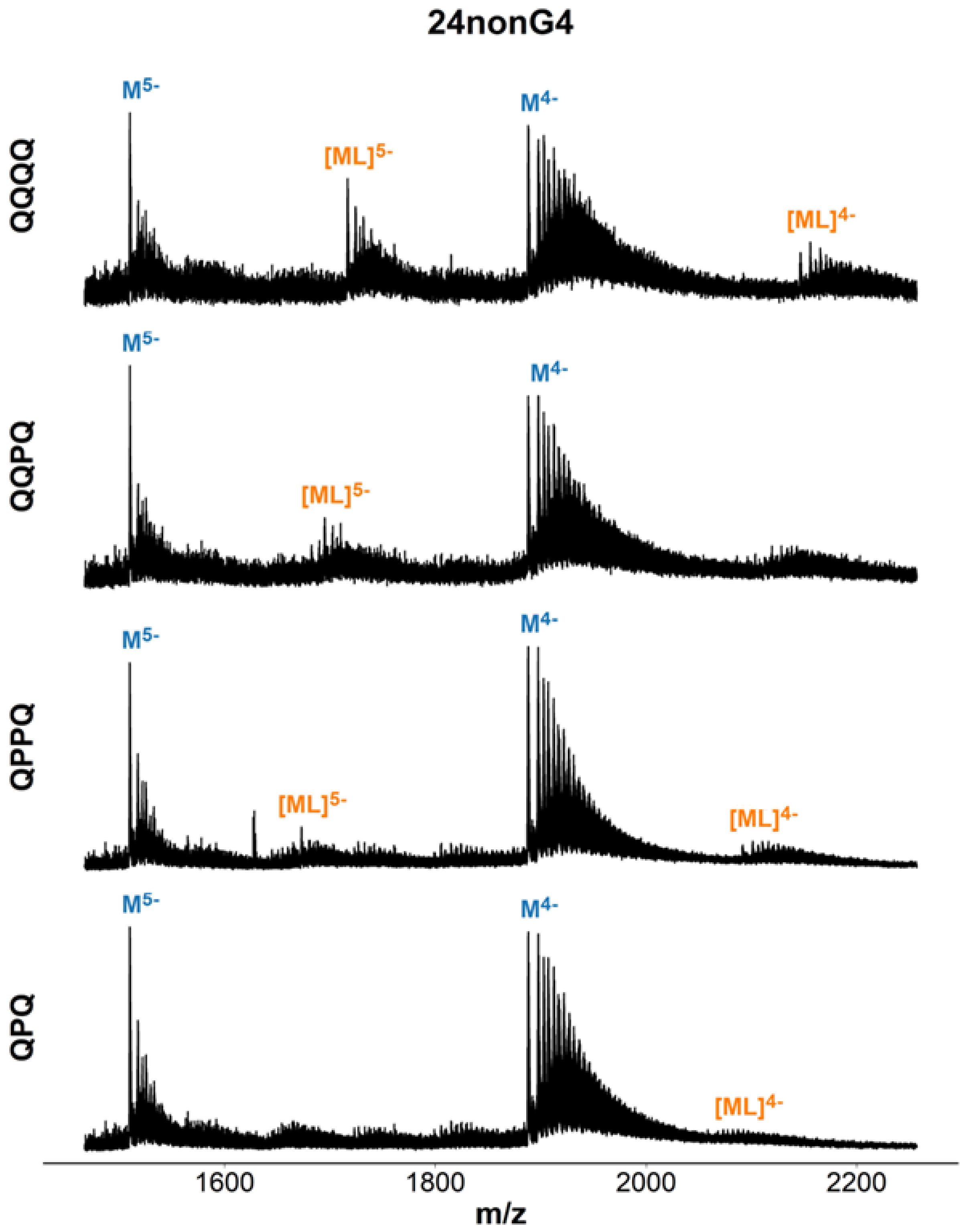

Figure S65. Mass spectra of 24nonG4 (dTGGGATGCGACAGAGAGGACGGGA) in presence of ligand. Samples contain 10 µM DNA, 20 µM ligand, 0.5 mM KCl, 100 mM TMAA (pH 6.8).

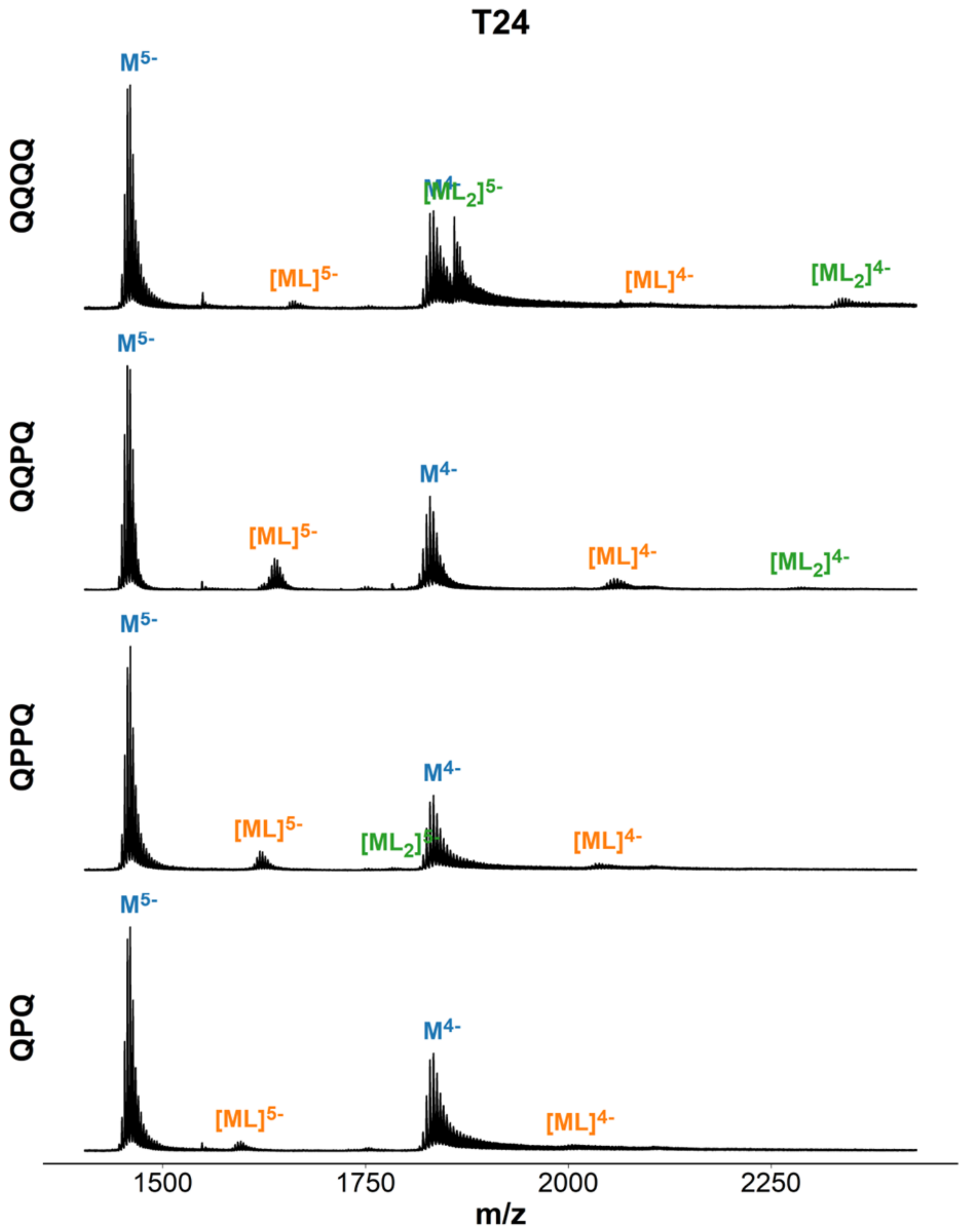

Figure S66. Mass spectra of T24 (dTTTTTTTTTTTTTTTTTTTTTTTTT) in presence of ligand. Samples contain 10 µM DNA, 20 µM ligand, 150 mM ammonium acetate (pH 6.8).

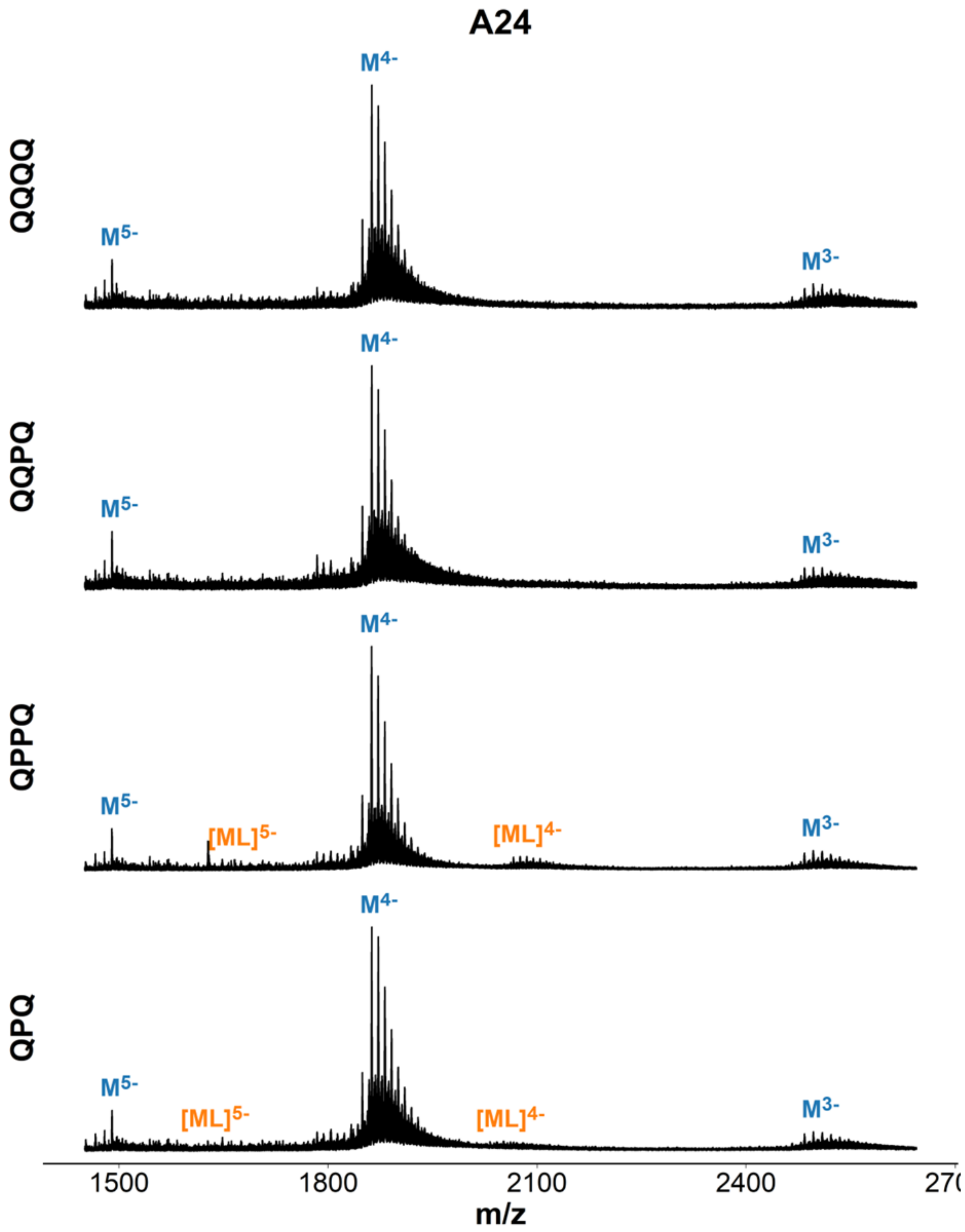

Figure S67. Mass spectra of A24(dAAAAAAAAAAAAAAAAAAAAAAAA) in presence of ligand. Samples contain 10 µM DNA, 20 µM ligand, 0.5 mM KCl, 100 mM TMAA (pH 6.8).

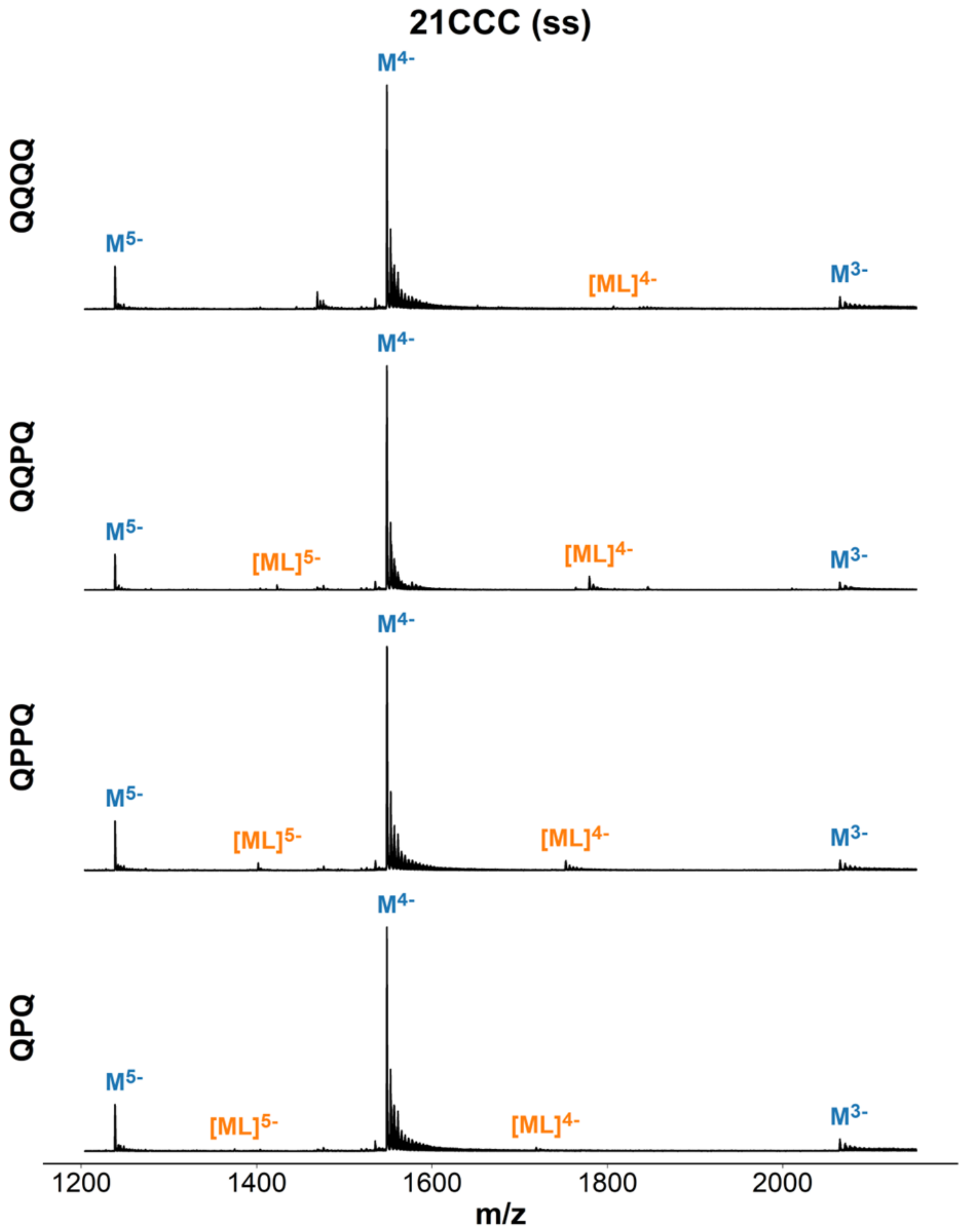

Figure S68. Mass spectra of 21CCC (dCCCTAACCCTAACCCTAACCC) in presence of ligand. Samples contain 10 µM DNA, 20 µM ligand, 150 mM ammonium acetate (pH 6.8).

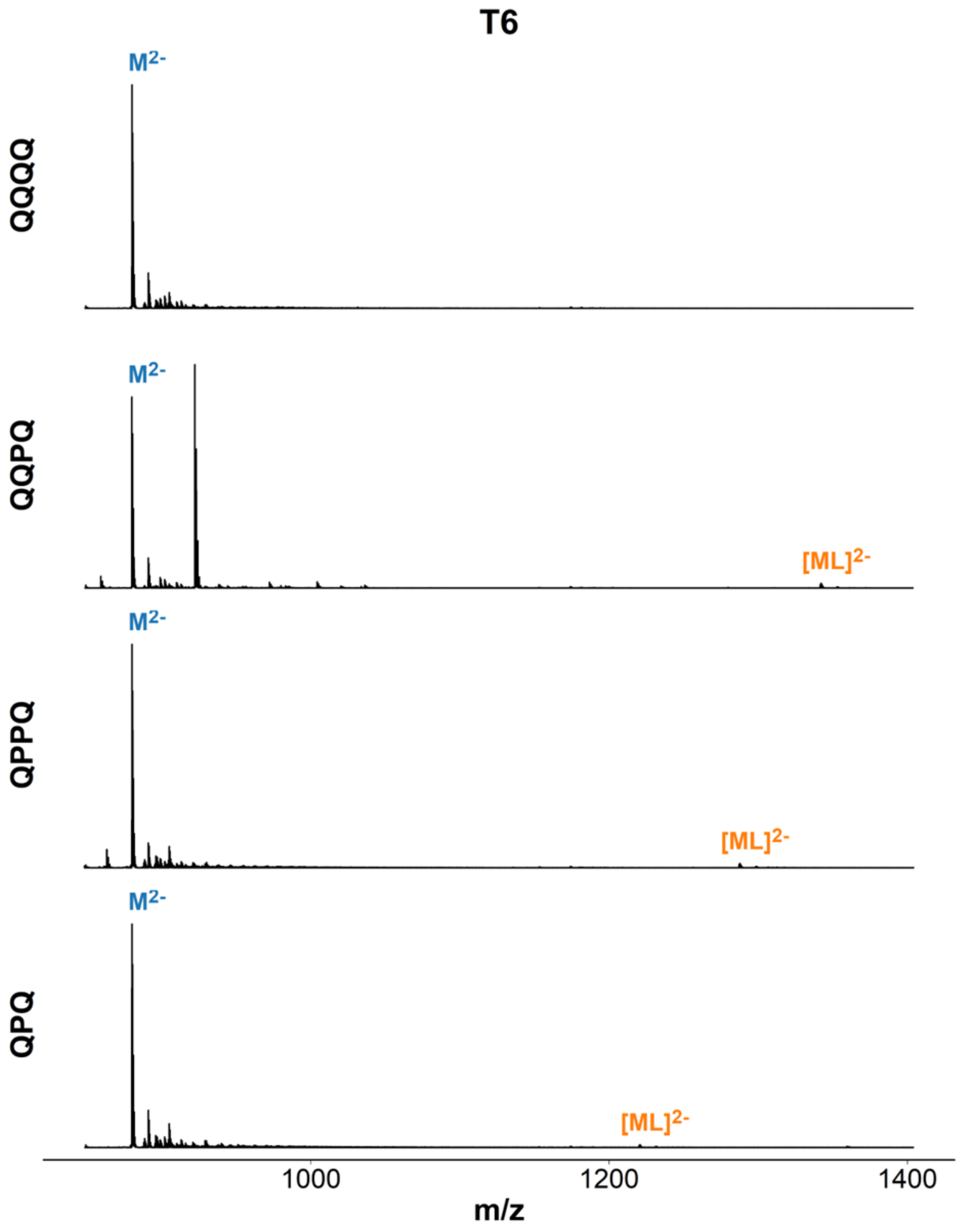

Figure S69. Mass spectra of T6 (dTTTTTT) in presence of ligand. Samples contain 10 µM DNA, 20 µM ligand, 150 mM ammonium acetate (pH 6.8).

ESI-MS titrations

K_D values and response factor estimates

Table S3. All K_D values obtained from ESI-MS titration of 6 DNA sequences with 7 foldamer ligands. K_D < 5 µM are highlighted in blue, K_D > 100 µM are faded out.

Dissociation constant estimates (µM)

	K_{D1}		K_{D2}		K_{D3}		K_{D4}	
	ESTIMATE	STD.DEV.	ESTIMATE	STD.DEV.	ESTIMATE	STD.DEV.	ESTIMATE	STD.DEV.
**2LK7**								
**QQPQ**	0.18	*0.079*	2.1	*0.69*				
**1XAV**								
**Q3**	0.11	*0.074*	1.2	*0.94*	4.1	*2.5*		
**QPQ**	13	*0.75*	110	*43*				
**QQQQ**	0.075	*0.059*	0.83	*0.49*				
**QQPQ**	0.41	*0.29*	1.9	*1.2*				
**QPPQ**	6.9	*2*	9.7	*5.7*				
**Q5**	0.25	*0.2*	0.7	*0.53*				
**Q8**	28	*6.7*						
**222T**								
**Q3**	0.1	*0.1*	2.8	*3.1*	6.1	*11*	64	*440*
**QPQ**	1.8	*0.25*	50	*13*				
**QQQQ**	0.088	*0.11*	1.6	*1.1*				
**QQPQ**	0.027	*0.081*	0.064	*0.19*				
**QPPQ**	0.1	*0.093*	1.4	*0.85*				
**Q5**	0.011	*0.014*	0.4	*0.35*				
**Q8**	7.8	*1.1*						
**5YEY**								
**Q3**	180	*28*						
**QPQ**	1900	*780*						
**QQQQ**	6.6	*1.3*						
**QQPQ**	0.54	*0.098*	200	*70*				
**QPPQ**	110	*23*	310	*760*				
**Q5**	3.9	*0.37*						
**21G**								
**Q3**	78	*19*	1500	*21000*				
**QPQ**	170	*26*						
**QQQQ**	330	*23*						
**QQPQ**	8.6	*2.3*	14	*9.2*				
**QPPQ**	160	*20*	1000	*4500*				
**Q5**	210	*74*	47	*45*				
**SS24**								
**Q3**	12	*6.2*	28	*87*	15	*63*		
**QPQ**	580	*110*						
**QQQQ**	180	*14*						
**QQPQ**	150	*21*						
**QPPQ**	270	*30*						
**Q5**	130	*42*	44	*41*				
**Q8**	210	*45*						
**T24**								
**Q3**	3400	*8200*	0.36	*0.91*	260000	*1.2e+08*	0.0092	*4.2*
**QQQQ**	350	*3400*	0.53	*5.5*				
**QQPQ**	270	*24*						
**QPPQ**	190	*25*						
**Q5**	180	*490*	1	*3*				
**Q8**	290	*87*						

Table S4. Estimated response factors of complex species ML, ML_2, ML_3, ML_4 relative to the unbound DNA (M with R = 1) using dT_6 as an internal calibrant. The calculation method was previously described.[6]

Response factors estimates

	K_{D1}		K_{D2}		K_{D3}		K_{D4}	
	ESTIMATE	*STD.DEV.*	*ESTIMATE*	*STD.DEV.*	*ESTIMATE*	*STD.DEV.*	*ESTIMATE*	*STD.DEV.*
**2LK7**								
**QQPQ**	0.987	0.099	0.968	0.071				
**1XAV**								
**Q3**	0.79	0.14	1.2	0.39	0.85	0.15		
**QPQ**	0.985	0.058	1.2	0.37				
**QQQQ**	1	0.15	0.95	0.067				
**QQPQ**	1	0.22	1	0.11				
**QPPQ**	1.1	0.31	0.91	0.2				
**Q5**	1.1	0.24	0.905	0.073				
**Q8**	1	0.2						
**222T**								
**Q3**	0.85	0.18	1.2	0.87	0.8	1.1	0.6	3.5
**QPQ**	0.944	0.052	1.1	0.2				
**QQQQ**	1	0.24	0.94	0.11				
**QQPQ**	1	0.36	0.998	0.088				
**QPPQ**	1	0.17	0.974	0.092				
**Q5**	0.95	0.13	0.948	0.065				
**Q8**	0.997	0.077						
**5YEY**								
**Q3**	0.97	0.18						
**QPQ**	1	0.58						
**QQQQ**	0.944	0.069						
**QQPQ**	1	0.028	1.2	0.39				
**QPPQ**	0.96	0.21	1	2.7				
**Q5**	0.974	0.026						
**21G**								
**Q3**	0.96	0.27	1	17				
**QPQ**	0.98	0.18						
**QQQQ**	0.997	0.087						
**QQPQ**	0.91	0.26	0.99	0.28				
**QPPQ**	0.99	0.13	1	5.3				
**Q5**	0.84	0.32	1.2	0.73				
**SS24**								
**Q3**	0.71	0.41	1	3.2	0.8	1.2		
**QPQ**	0.99	0.26						
**QQQQ**	0.997	0.088						
**QQPQ**	1	0.16						
**QPPQ**	1	0.14						
**Q5**	0.76	0.28	1.2	0.69				
**Q8**	0.98	0.26						
**T24**								
**Q3**			1.1	0.2			0.64	0.44
**QQQQ**	1	12	0.9	0.32				
**QQPQ**	1	0.11						
**QPPQ**	0.98	0.16						
**Q5**	0.6	1.9	0.95	0.18				
**Q8**	1	0.39						

Mass spectra

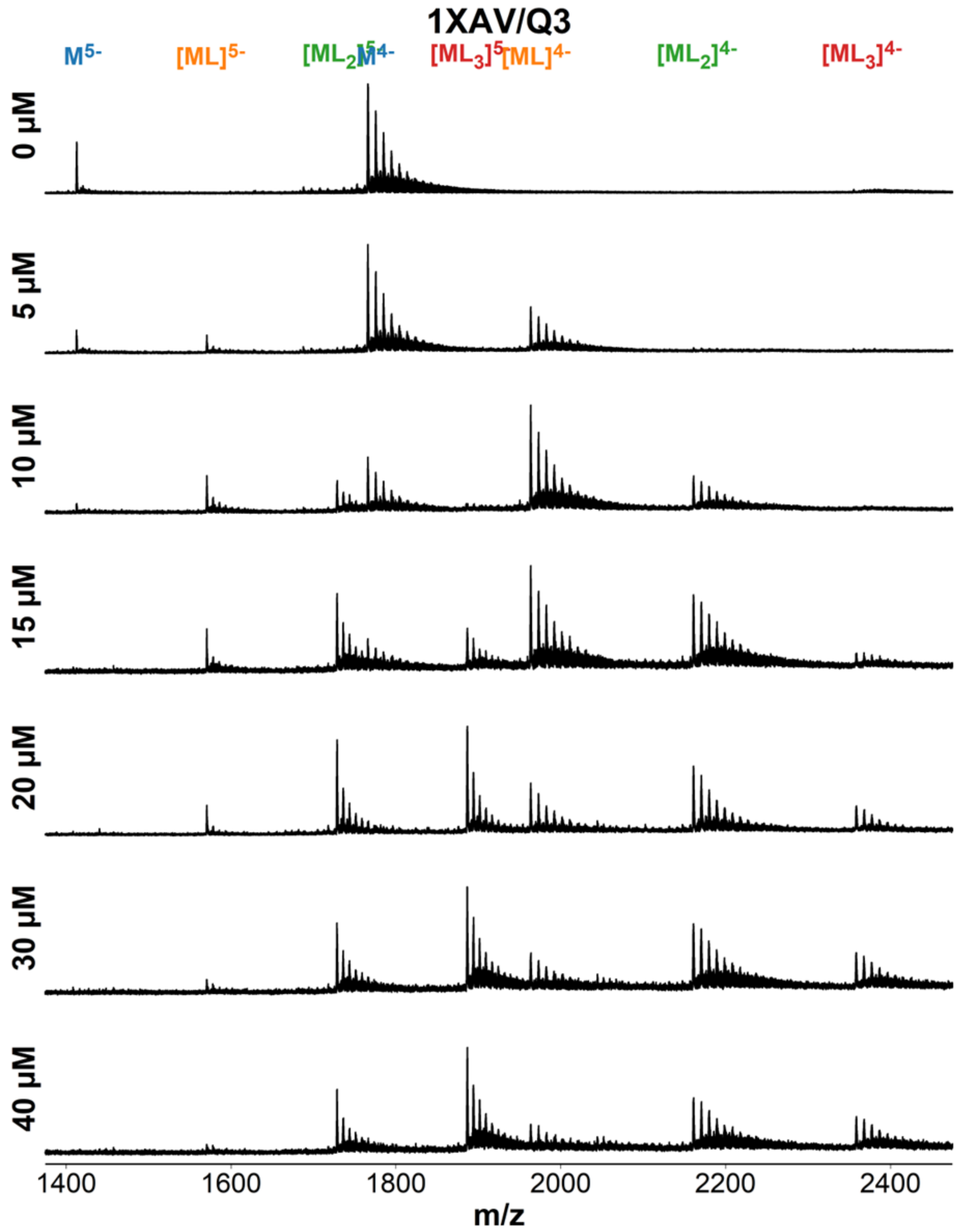

*Figure S70. ESI-MS titration of 1XAV (dT**GAGGGTGGG**TA**GGGTGGG**TAA) with foldamer QQQ. Samples contain 10 µM DNA, 0-40 µM ligand, 0.5 mM KCl, 100 mM TMAA (pH 6.8).*

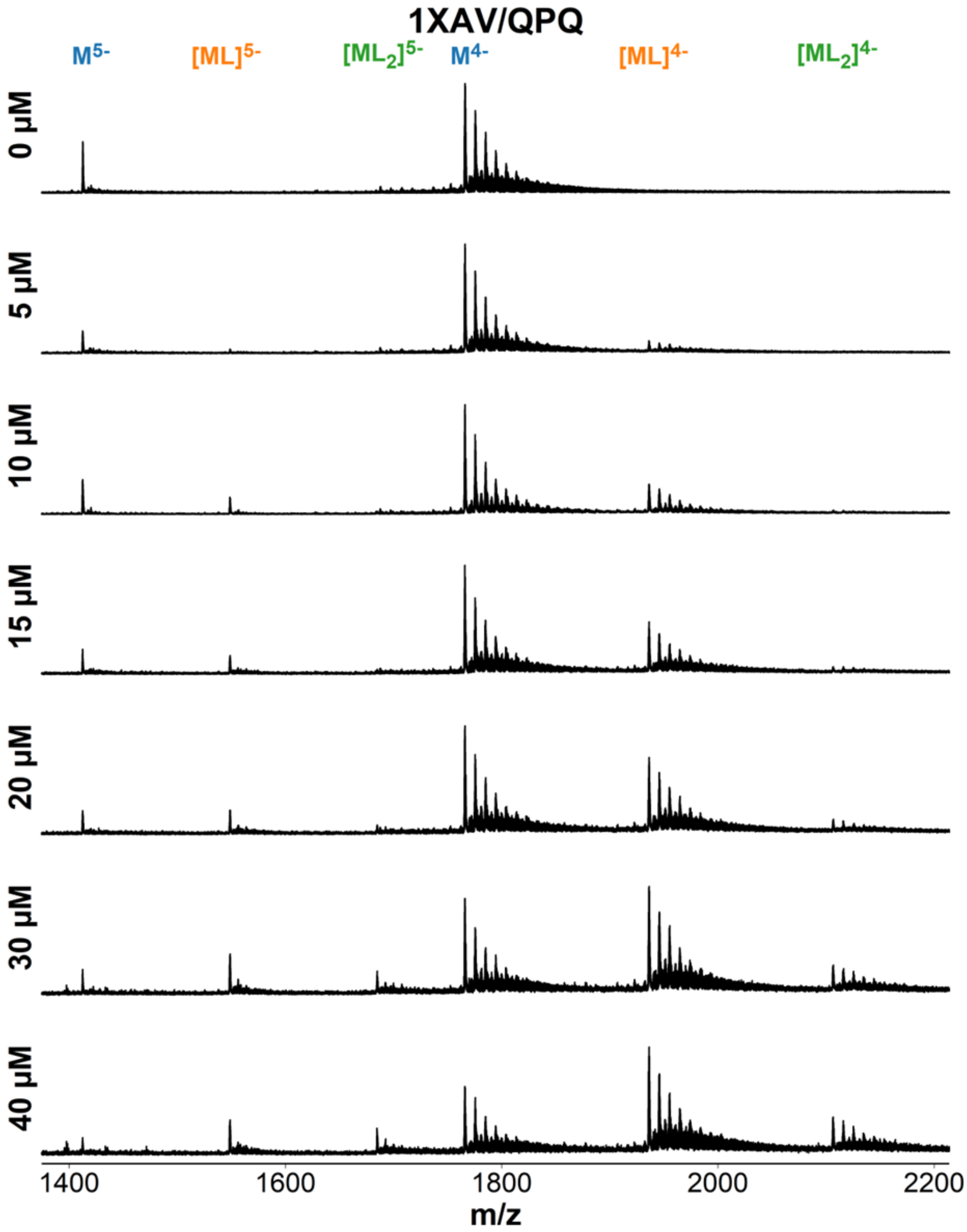

*Figure S71. ESI-MS titration of 1XAV (dT**GAGGG**T**GGG**TA**GGG**T**GGG**TAA) with foldamer QPQ. Samples contain 10 µM DNA, 0-40 µM ligand, 0.5 mM KCl, 100 mM TMAA (pH 6.8).*

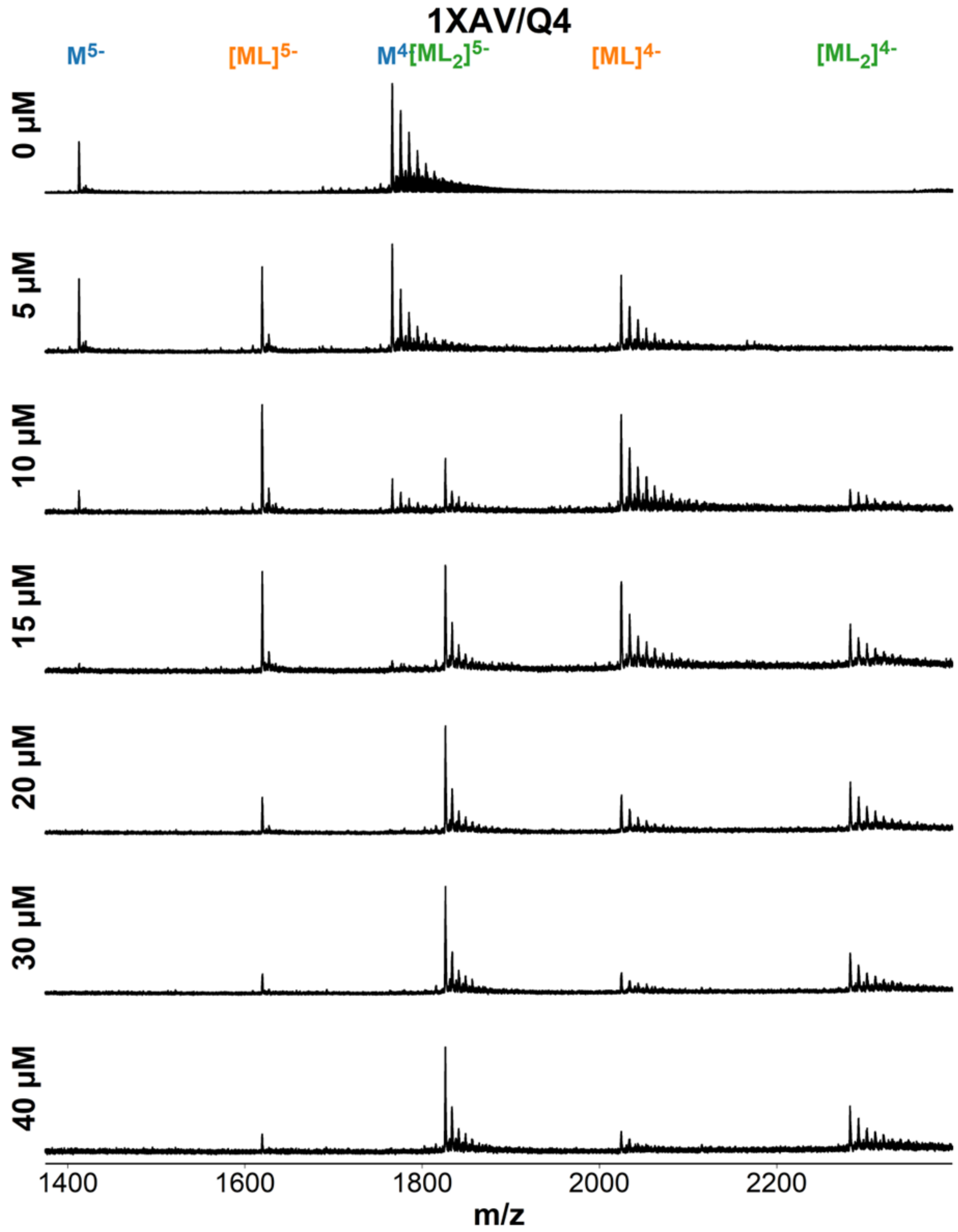

*Figure S72. ESI-MS titration of 1XAV (dT**G**A**GGG**T**GGG**TA**GGG**T**GGG**TAA) with foldamer QQQQ. Samples contain 10 µM DNA, 0-40 µM ligand, 0.5 mM KCl, 100 mM TMAA (pH 6.8).*

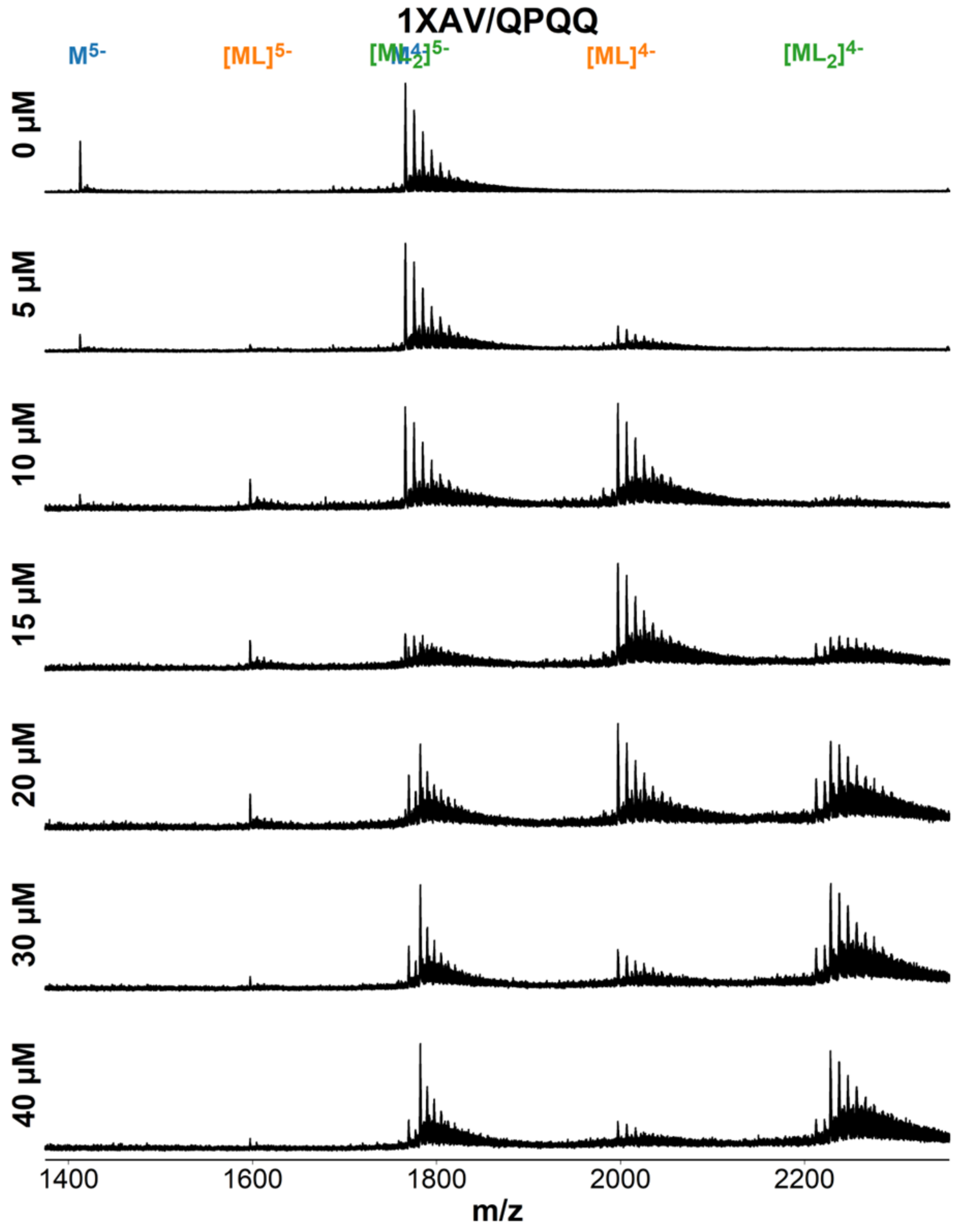

*Figure S73. ESI-MS titration of 1XAV (dT**GA**GGG**T**GGG**TA**GGG**T**GGG**TAA) with foldamer QQPQ. Samples contain 10 µM DNA, 0-40 µM ligand, 0.5 mM KCl, 100 mM TMAA (pH 6.8).*

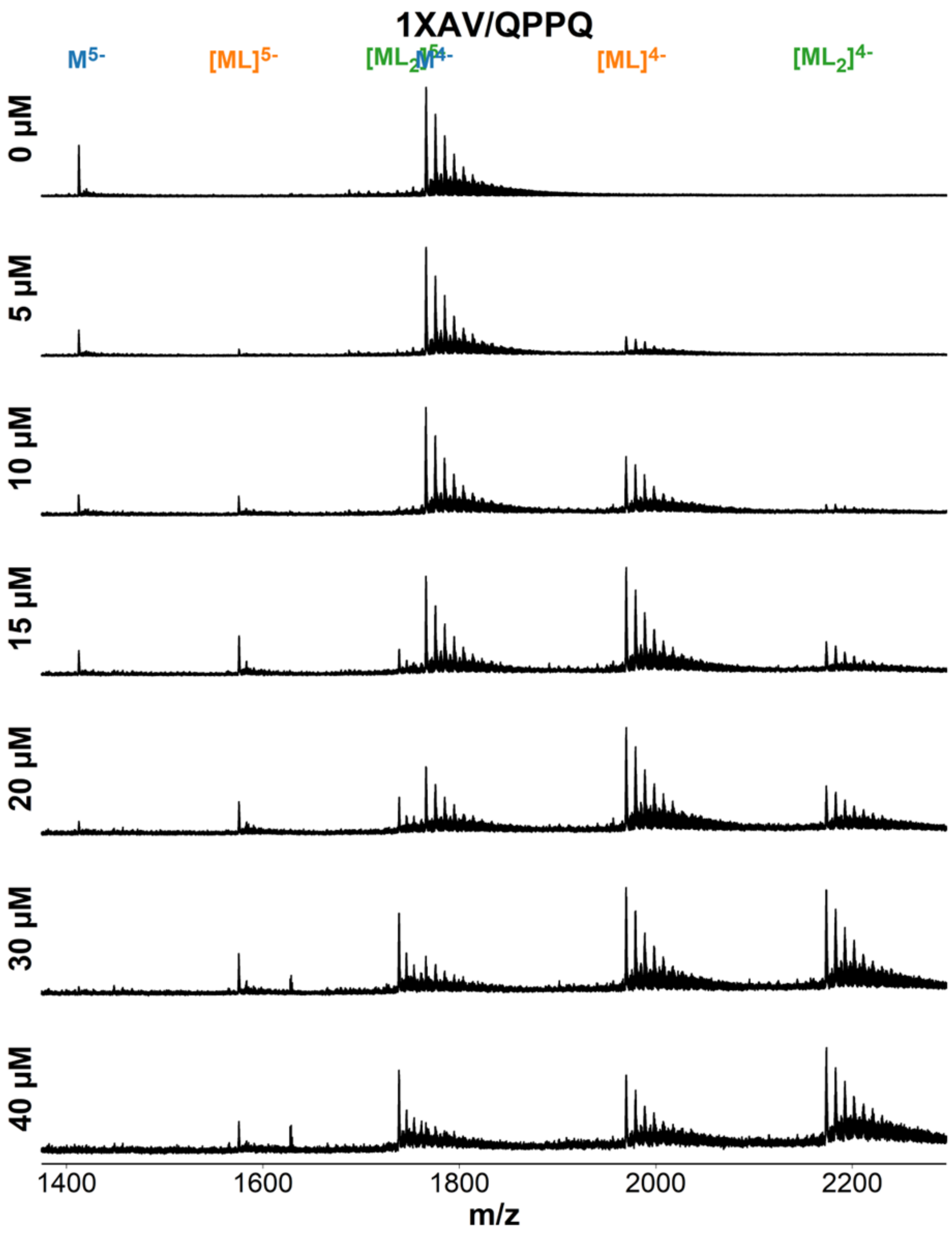

*Figure S74. ESI-MS titration of 1XAV (dT**GA**GGG**T**GGG**TA**GGG**T**GGG**TAA**) with foldamer QPPQ. Samples contain 10 µM DNA, 0-40 µM ligand, 0.5 mM KCl, 100 mM TMAA (pH 6.8).*

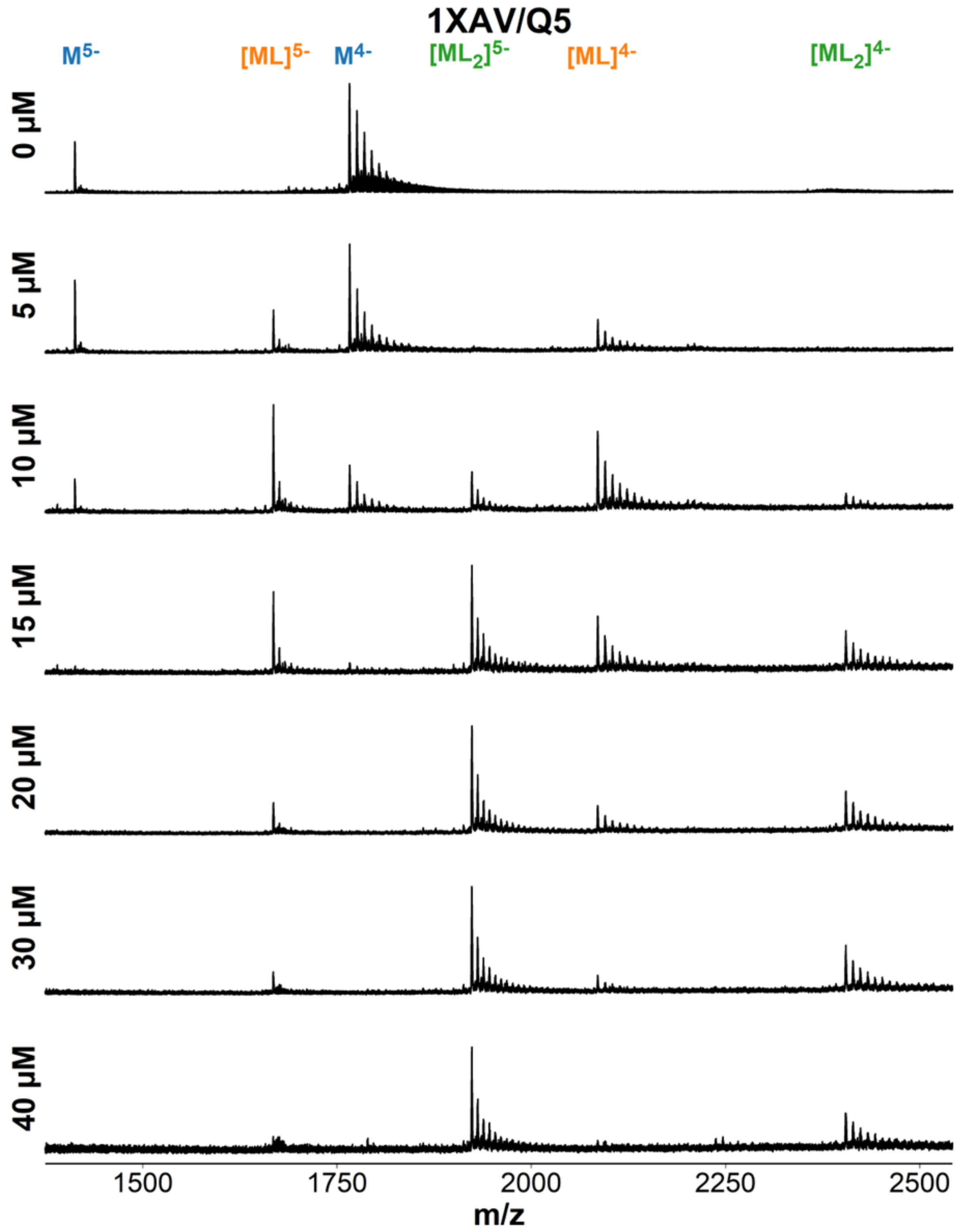

*Figure S75. ESI-MS titration of 1XAV (dT**G**A**GGG**T**GGG**TA**GGG**T**GGG**TAA) with foldamer QQQQQ. Samples contain 10 µM DNA, 0-40 µM ligand, 0.5 mM KCl, 100 mM TMAA (pH 6.8).*

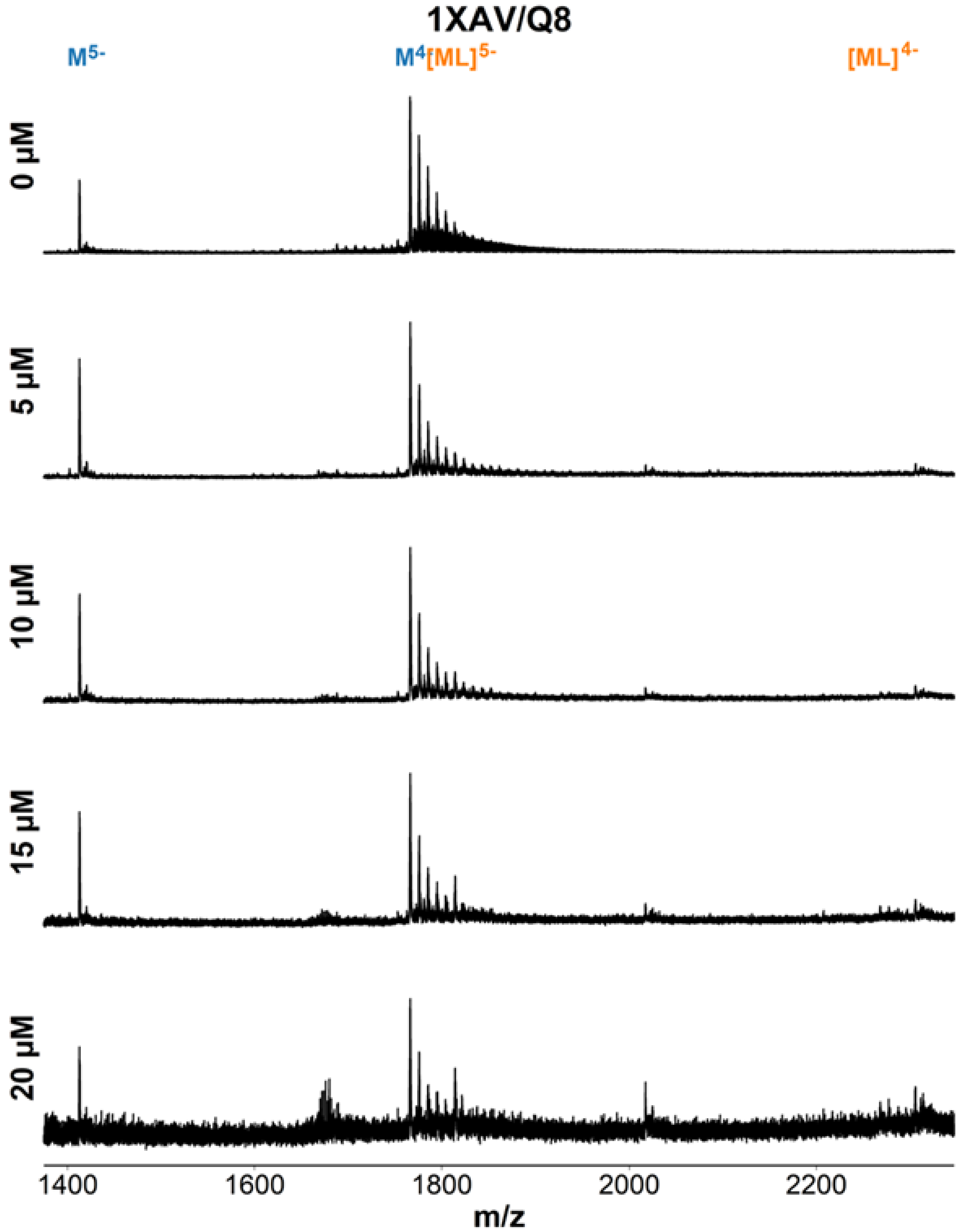

*Figure S76. ESI-MS titration of 1XAV (dT**GA**GGG**T**GGG**TA**GGG**T**GGG**TAA) with foldamer QQQQQQQQ. Samples contain 10 µM DNA, 0-20 µM ligand, 0.5 mM KCl, 100 mM TMAA (pH 6.8).*

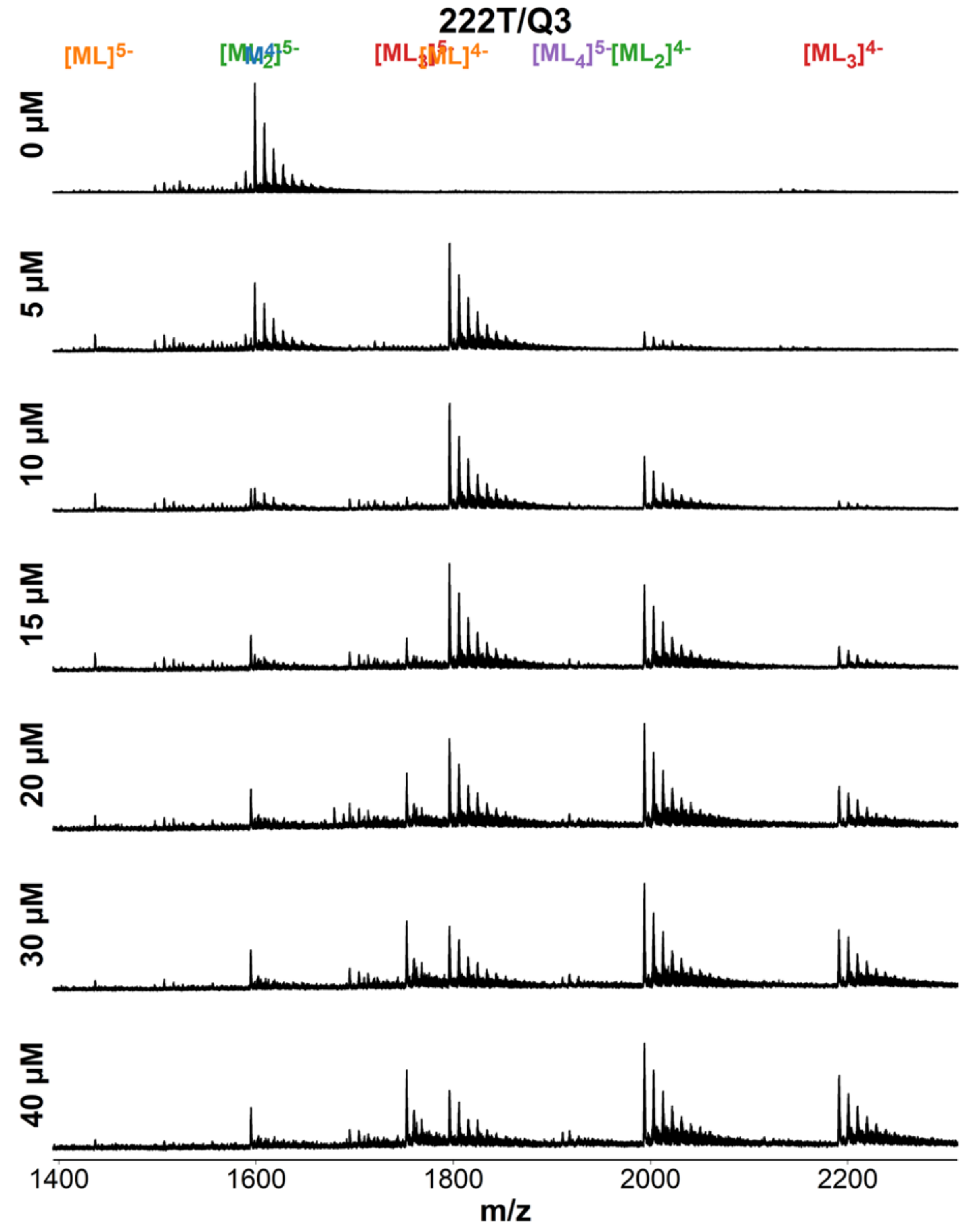

*Figure S77. ESI-MS titration of 222T (dT**GGG**TT**GGG**TT**GGG**TT**GGG**T) with foldamer QQQ. Samples contain 10 µM DNA, 0-40 µM ligand, 0.5 mM KCl, 100 mM TMAA (pH 6.8).*

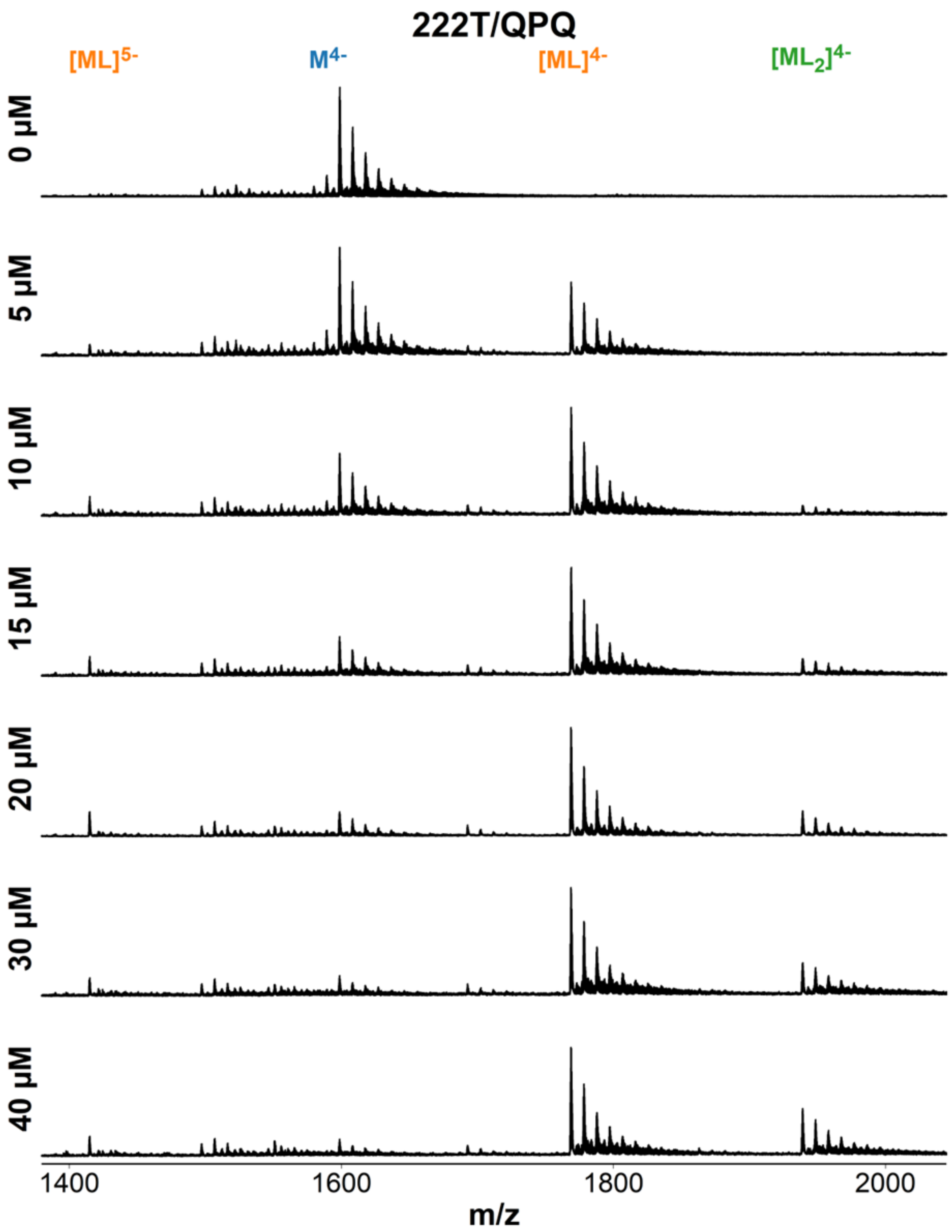

*Figure S78. ESI-MS titration of 222T (dT**GGG**TT**GGG**TT**GGG**TT**GGG**T) with foldamer QPQ. Samples contain 10 µM DNA, 0-40 µM ligand, 0.5 mM KCl, 100 mM TMAA (pH 6.8).*

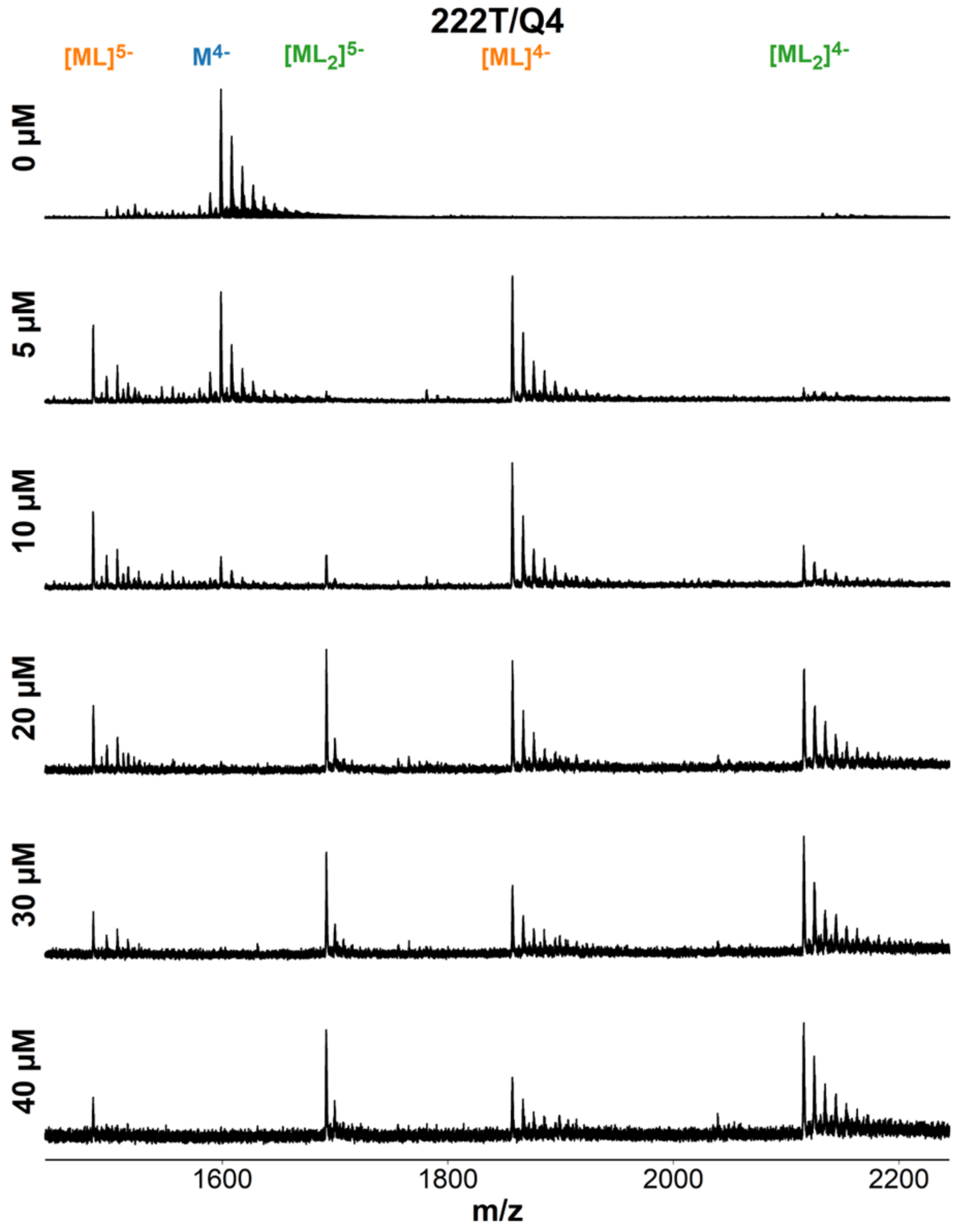

*Figure S79. ESI-MS titration of 222T (dT**GGG**TT**GGG**TT**GGG**TT**GGG**T) with foldamer QQQQ. Samples contain 10 µM DNA, 0-40 µM ligand, 0.5 mM KCl, 100 mM TMAA (pH 6.8).*

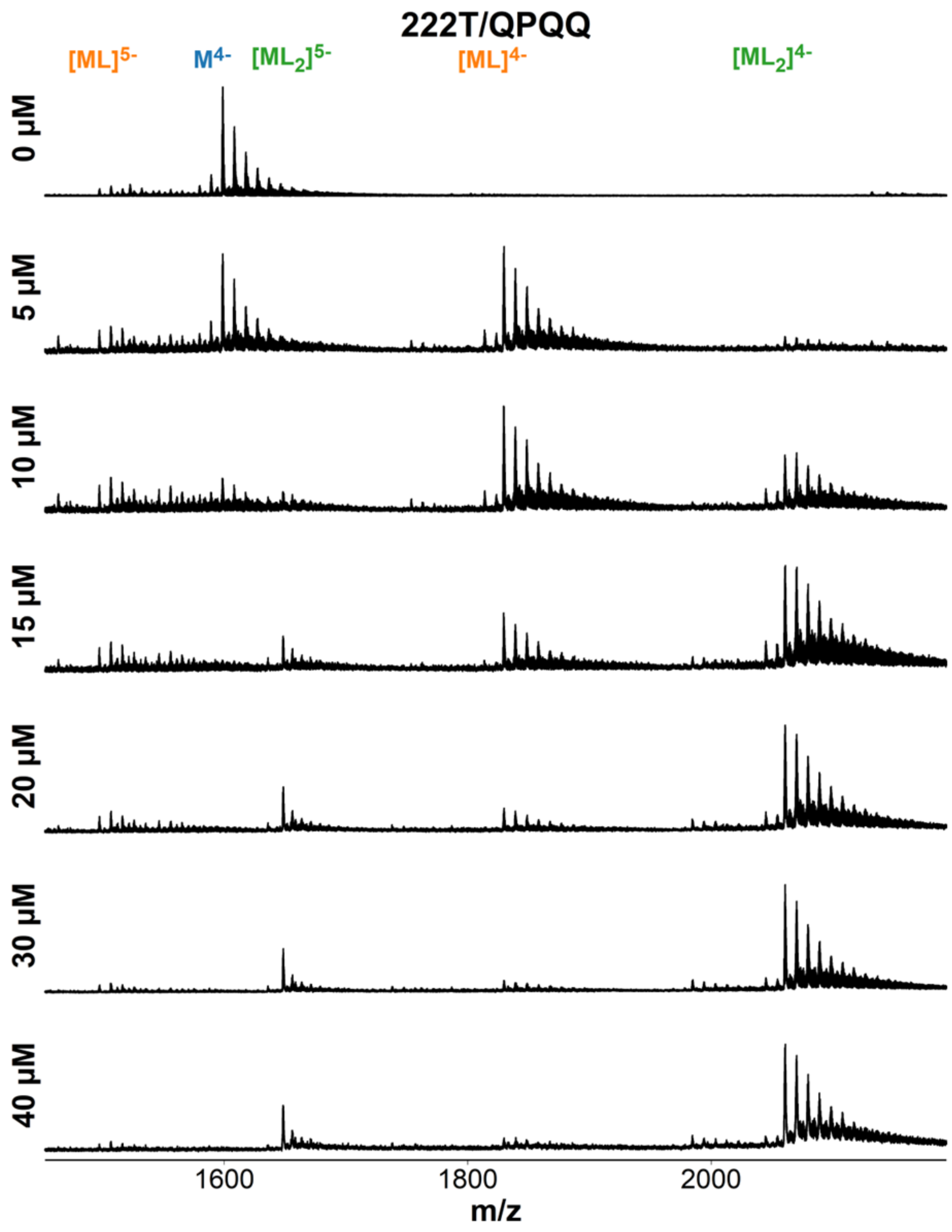

*Figure S80. ESI-MS titration of 222T (dT**GGG**TT**GGG**TT**GGG**TT**GGG**T) with foldamer QQPQ. Samples contain 10 µM DNA, 0-40 µM ligand, 0.5 mM KCl, 100 mM TMAA (pH 6.8).*

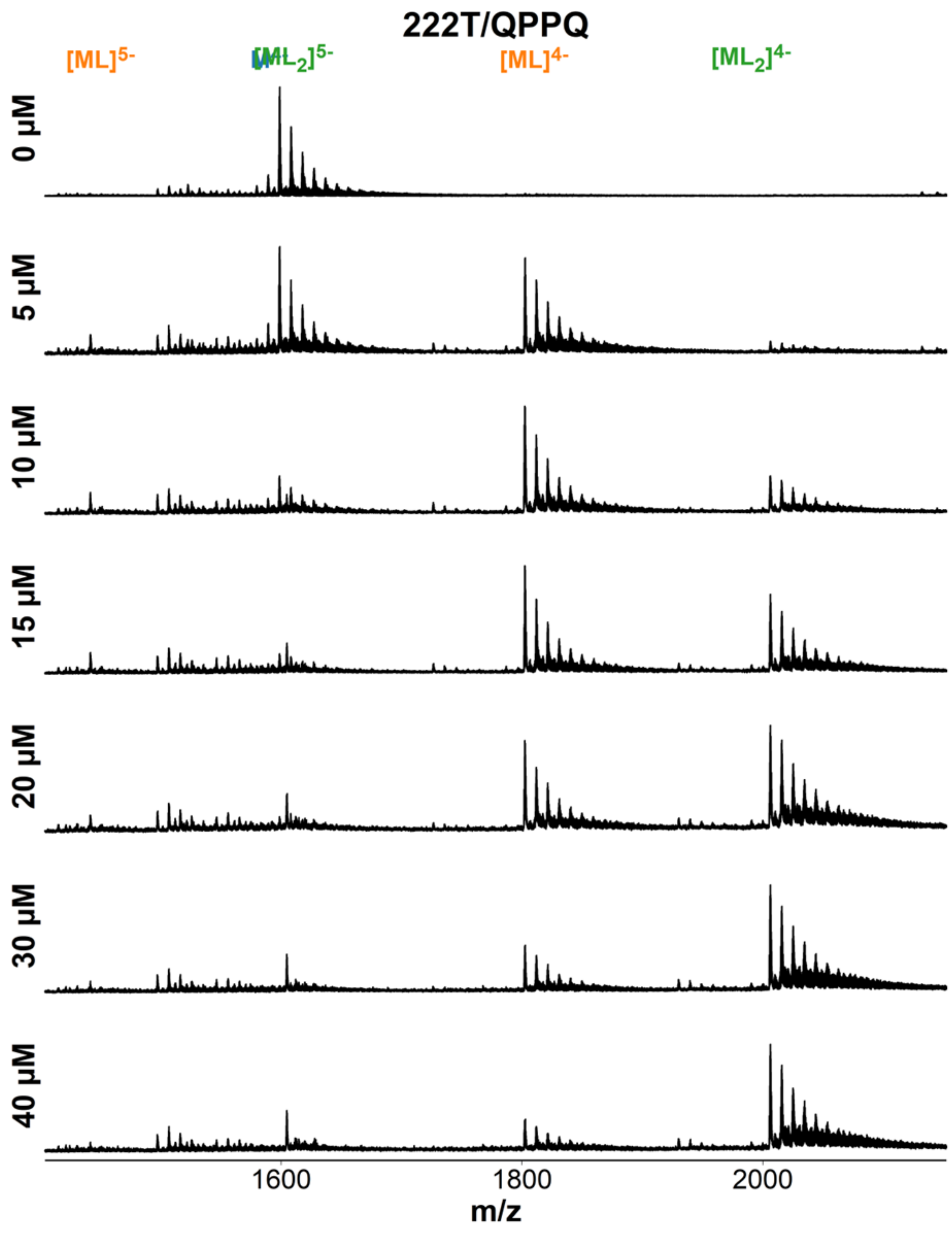

*Figure S81. ESI-MS titration of 222T (dT**GGG**TT**GGG**TT**GGG**TT**GGG**T) with foldamer QPPQ. Samples contain 10 µM DNA, 0-40 µM ligand, 0.5 mM KCl, 100 mM TMAA (pH 6.8).*

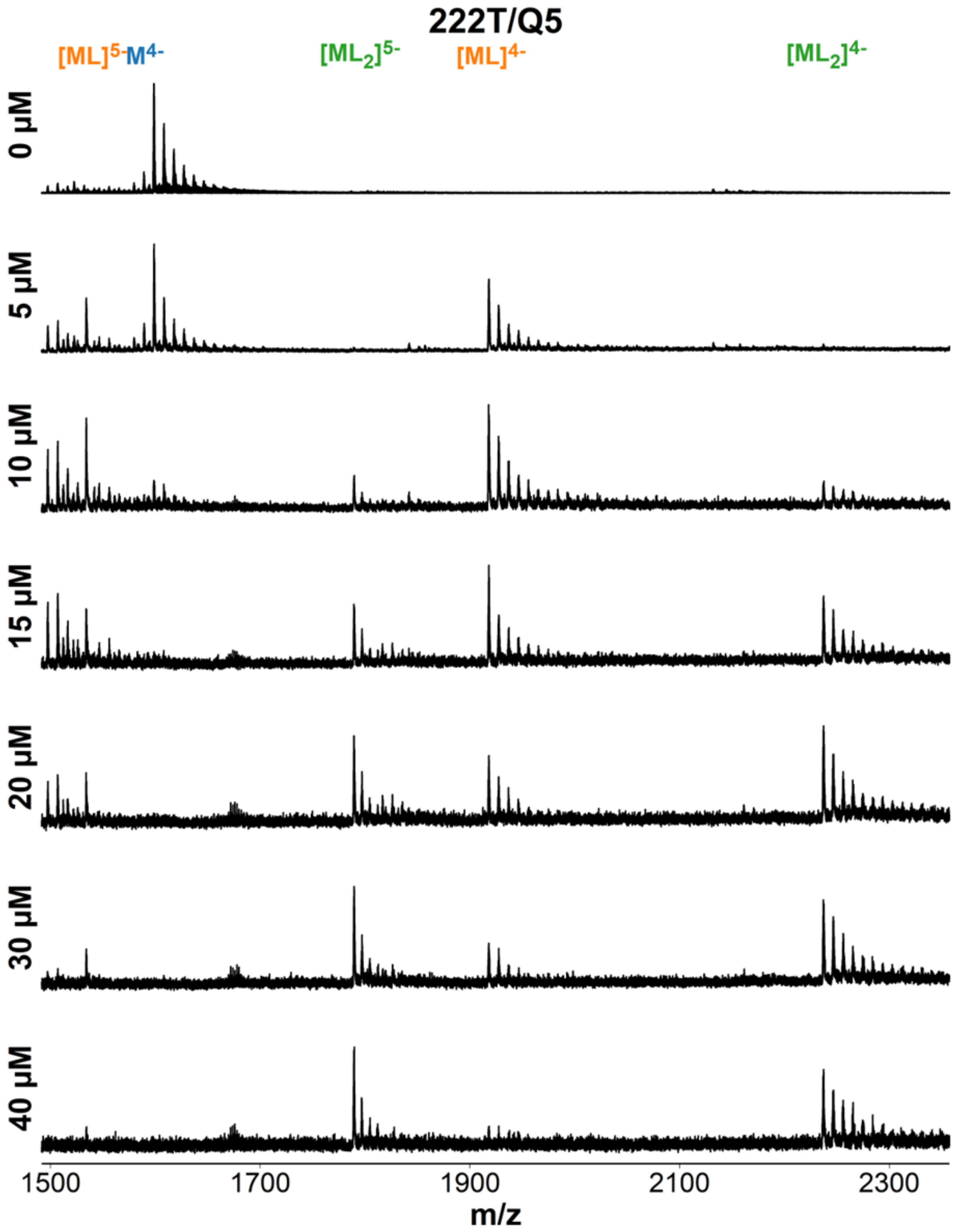

*Figure S82. ESI-MS titration of 222T (dT**GGG**TT**GGG**TT**GGG**TT**GGG**T) with foldamer QQQQQ. Samples contain 10 µM DNA, 0-40 µM ligand, 0.5 mM KCl, 100 mM TMAA (pH 6.8).*

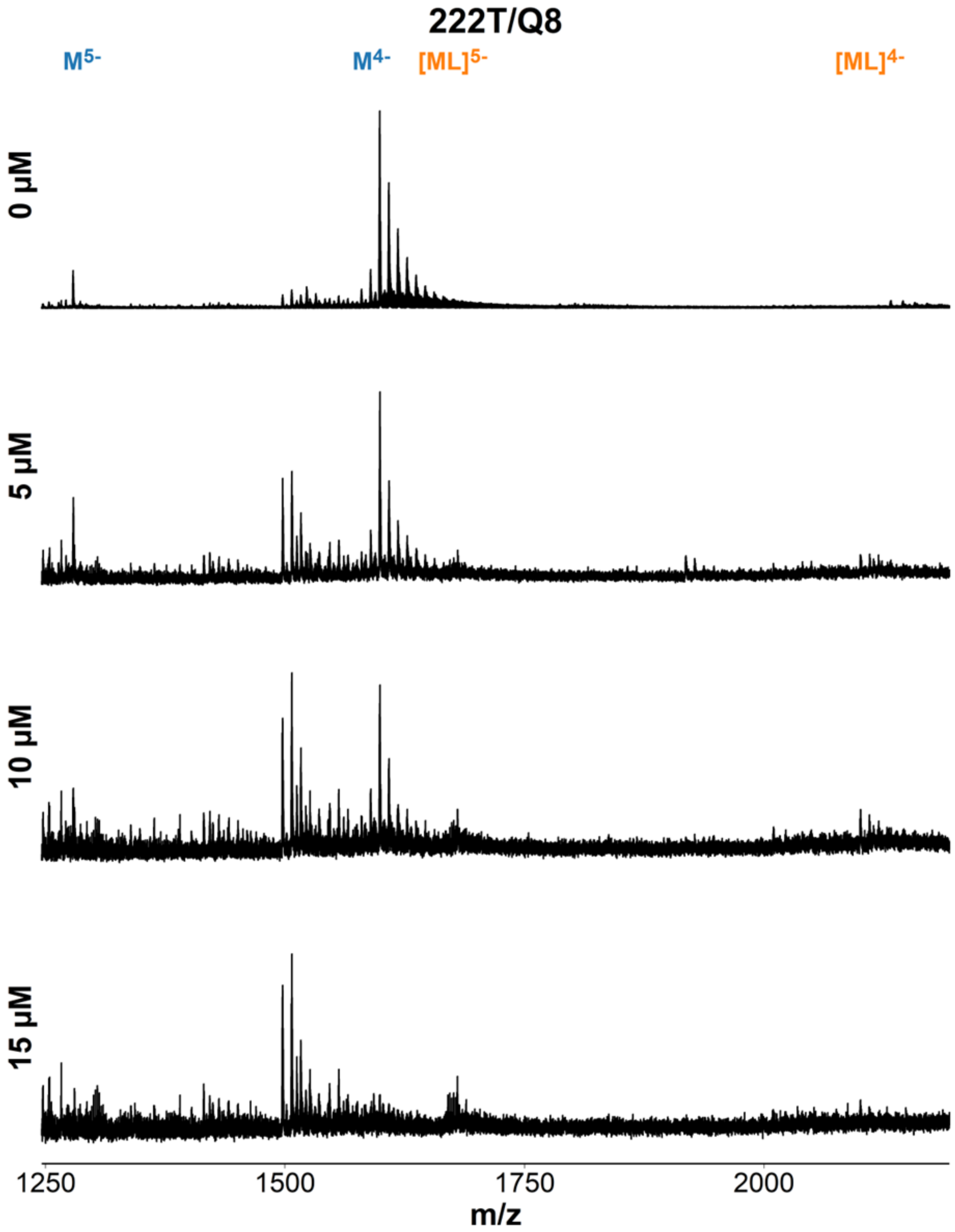

*Figure S83. ESI-MS titration of 222T (dT**GGG**TT**GGG**TT**GGG**TT**GGG**T) with foldamer QQQQQQQQ. Samples contain 10 µM DNA, 0-15 µM ligand, 0.5 mM KCl, 100 mM TMAA (pH 6.8).*

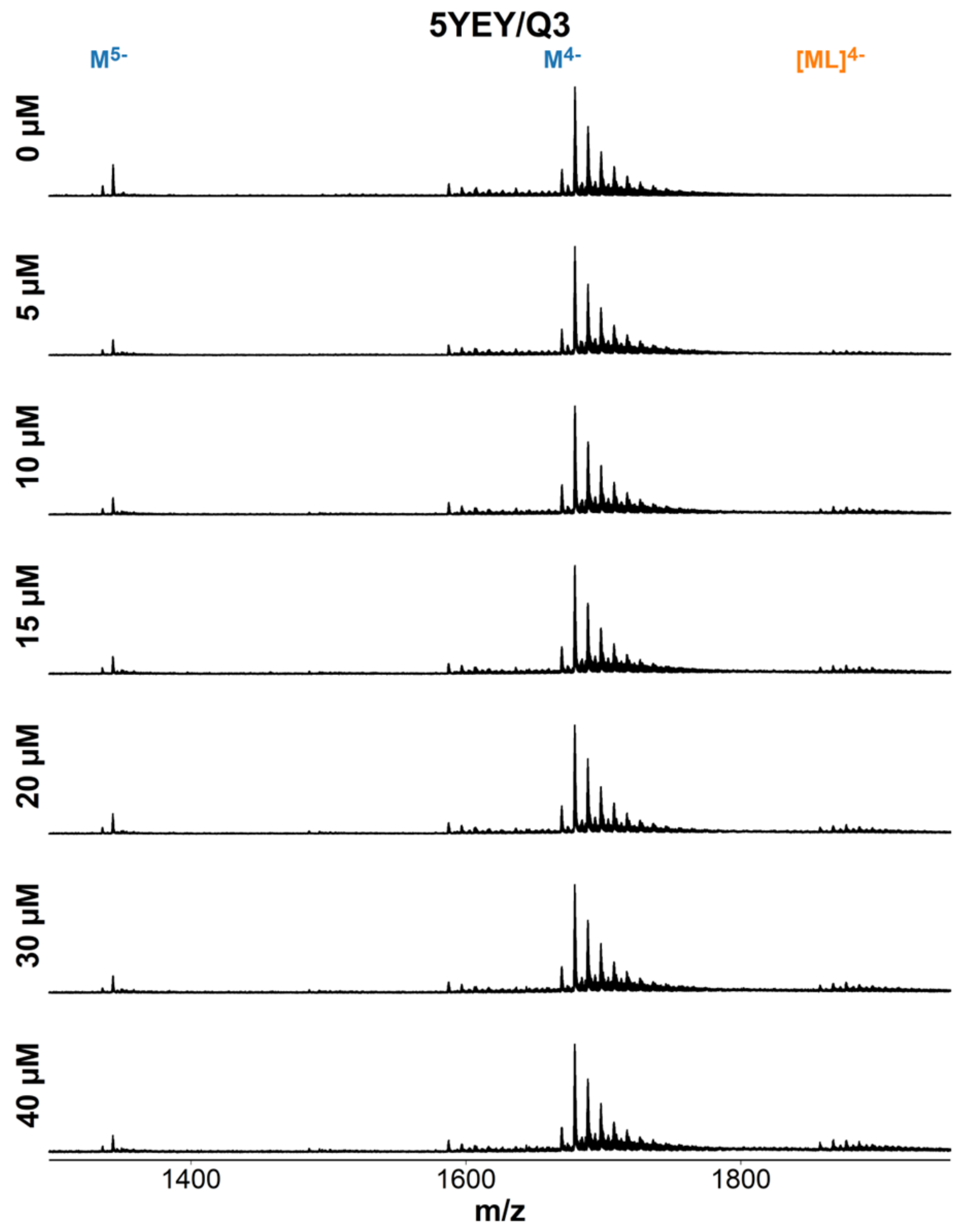

*Figure S84. ESI-MS titration of 5YEY (d**GGG**TTA**GGG**TTA**GGG**TTT**GGG**) with foldamer QQQ. Samples contain 10 µM DNA, 0-40 µM ligand, 0.5 mM KCl, 100 mM TMAA (pH 6.8).*

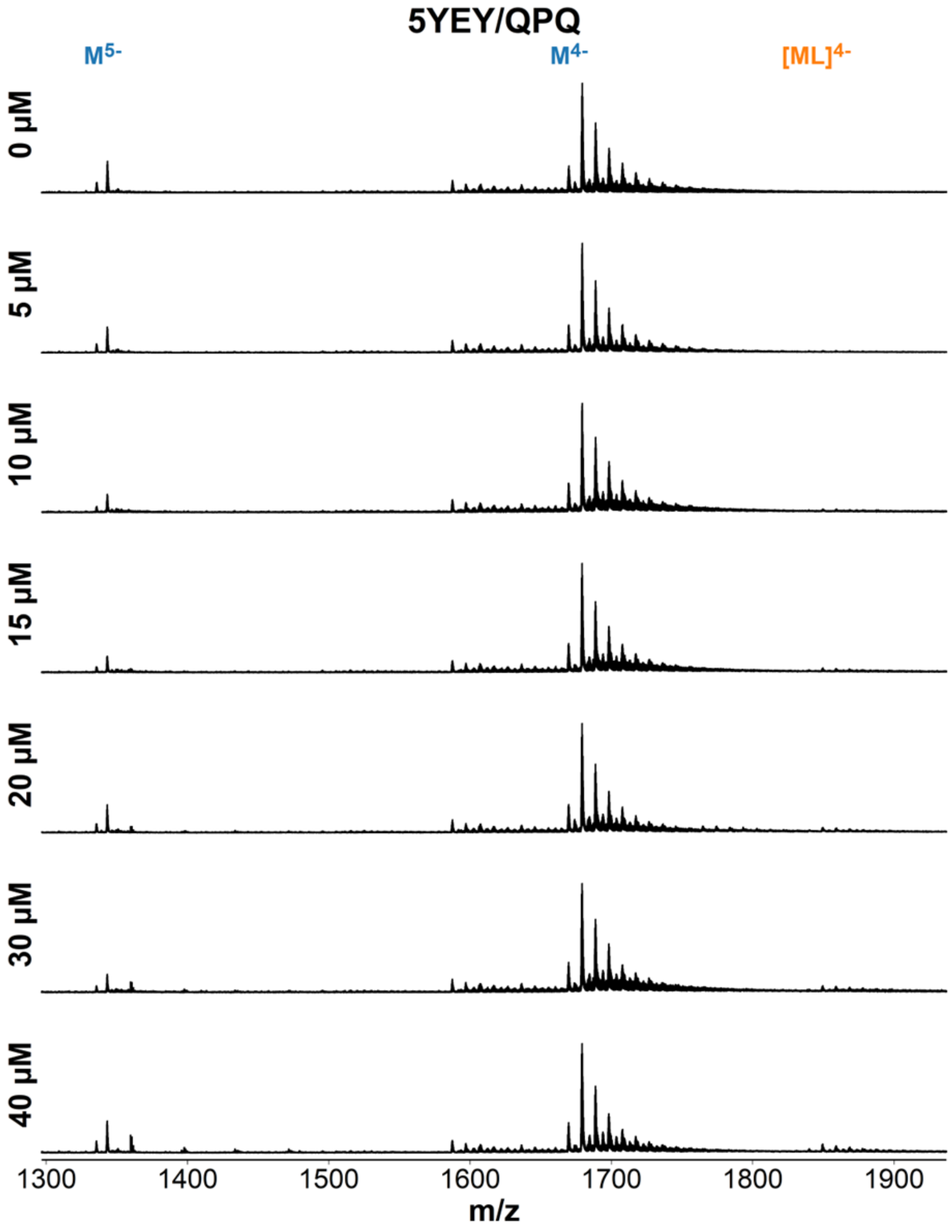

*Figure S85. ESI-MS titration of 5YEY (d**GGG**TTA**GGG**TTA**GGG**TTT**GGG**) with foldamer QPQ. Samples contain 10 µM DNA, 0-40 µM ligand, 0.5 mM KCl, 100 mM TMAA (pH 6.8).*

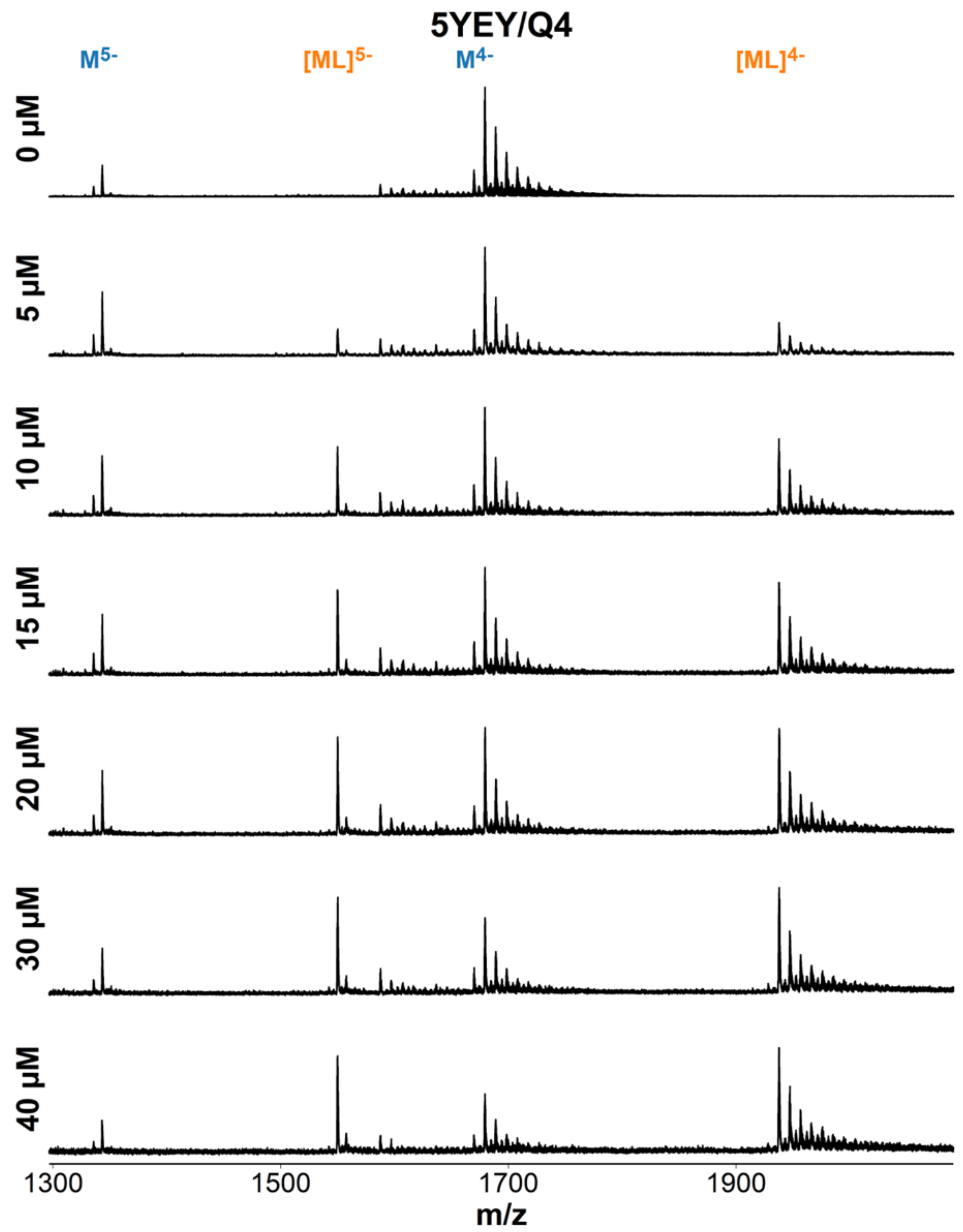

*Figure S86. ESI-MS titration of 5YEY (d**GGG**TTA**GGG**TTA**GGG**TTT**GGG**) with foldamer QQQQ. Samples contain 10 µM DNA, 0-40 µM ligand, 0.5 mM KCl, 100 mM TMAA (pH 6.8).*

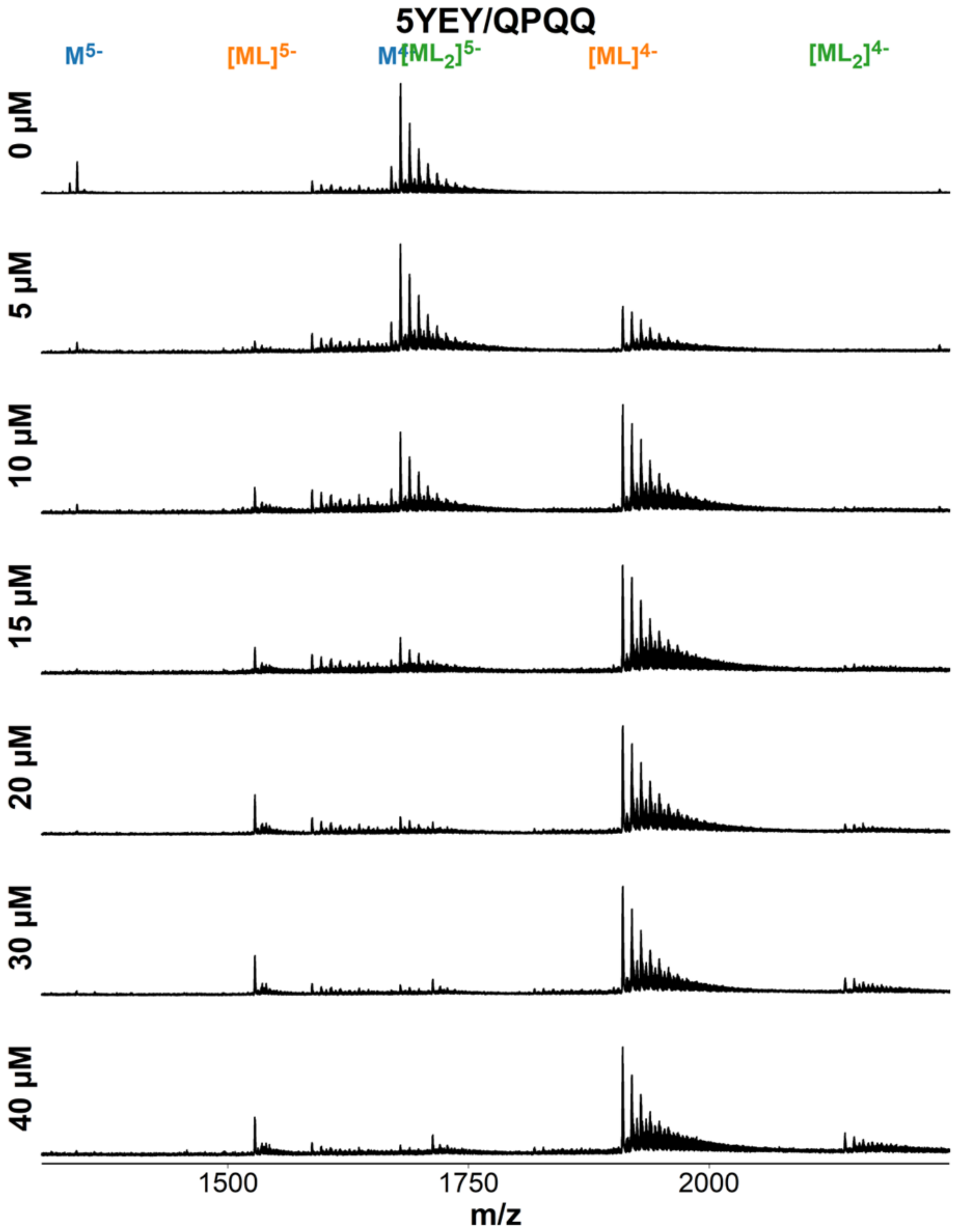

*Figure S87. ESI-MS titration of 5YEY (d**GGG**TTA**GGG**TTA**GGG**TTT**GGG**) with foldamer QQPQ. Samples contain 10 µM DNA, 0-40 µM ligand, 0.5 mM KCl, 100 mM TMAA (pH 6.8).*

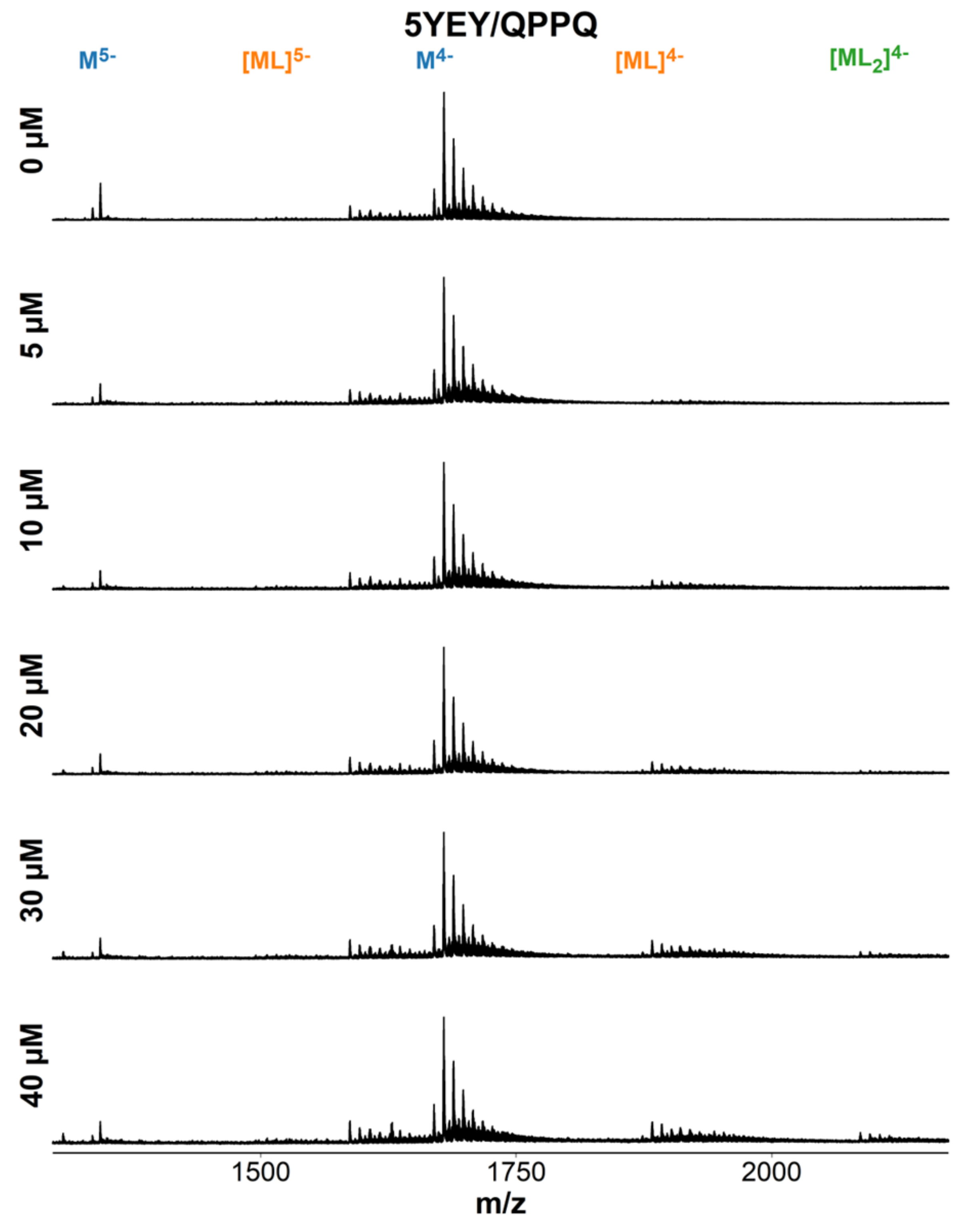

*Figure S88. ESI-MS titration of 5YEY (d**GGG**TTA**GGG**TTA**GGG**TTT**GGG**) with foldamer QPPQ. Samples contain 10 µM DNA, 0-40 µM ligand, 0.5 mM KCl, 100 mM TMAA (pH 6.8).*

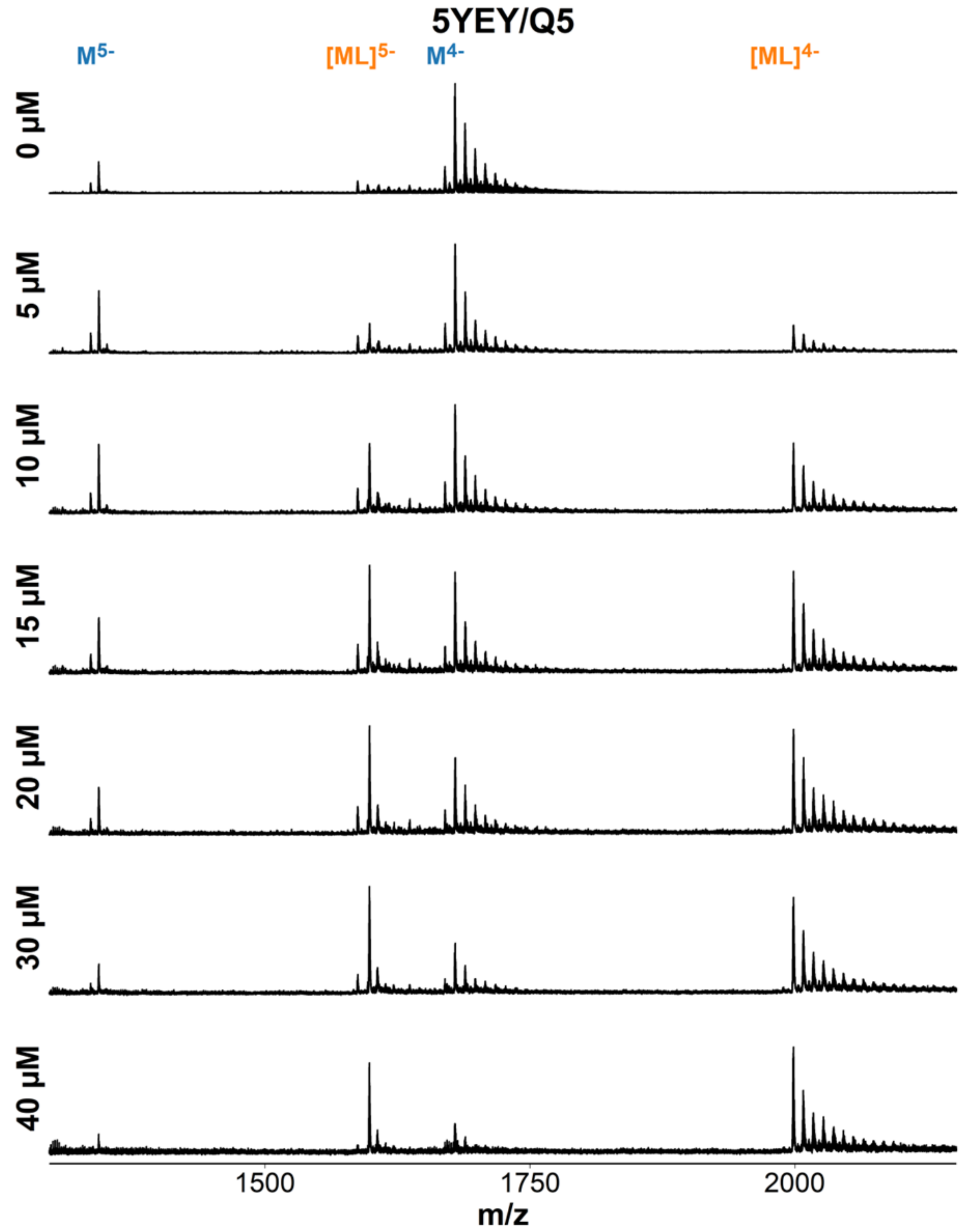

*Figure S89. ESI-MS titration of 5YEY (d**GGG**TTA**GGG**TTA**GGG**TTT**GGG**) with foldamer QQQQQ. Samples contain 10 µM DNA, 0-40 µM ligand, 0.5 mM KCl, 100 mM TMAA (pH 6.8).*

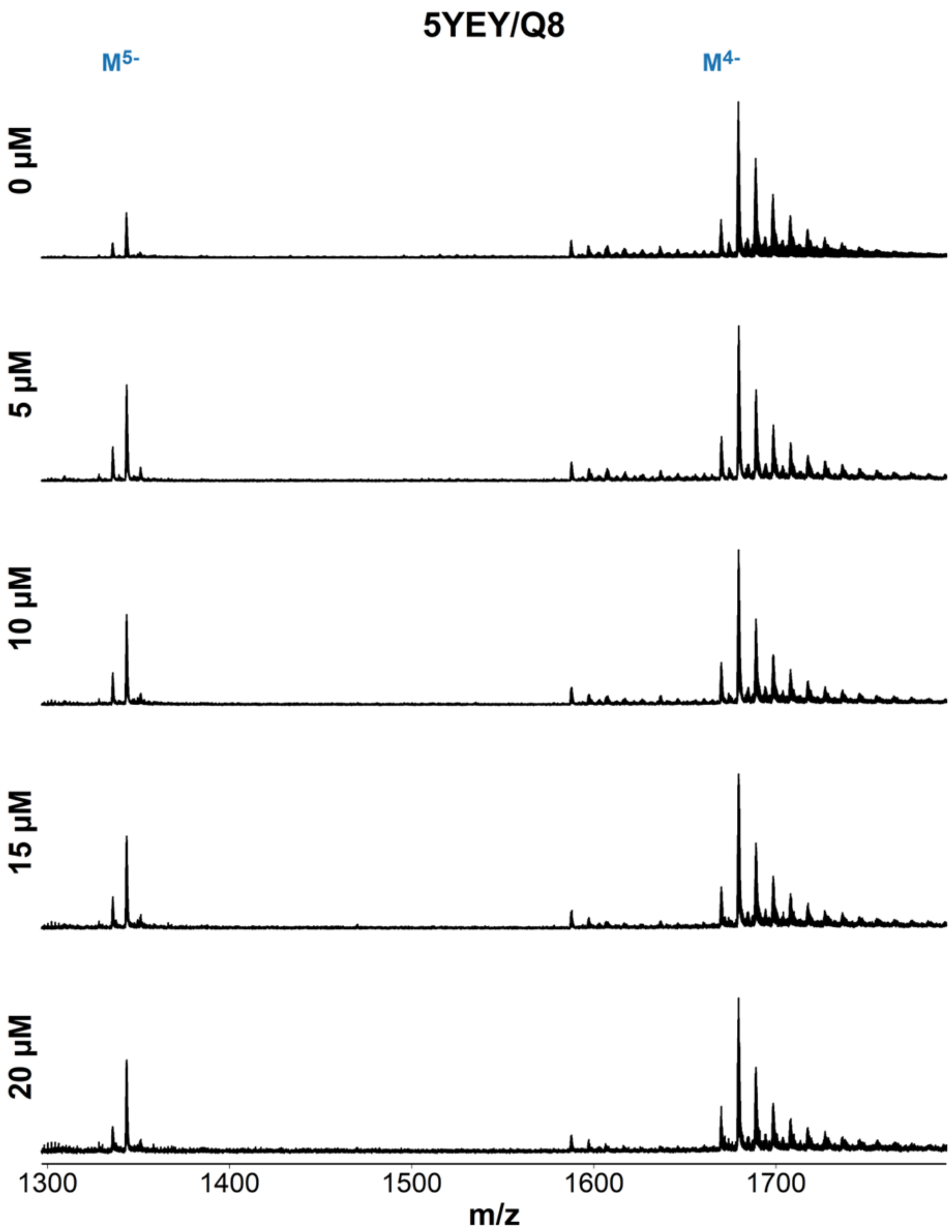

*Figure S90. ESI-MS titration of 5YEY (d**GGG**TTA**GGG**TTA**GGG**TTT**GGG**) with foldamer QQQQQQQQ. Samples contain 10 µM DNA, 0-20 µM ligand, 0.5 mM KCl, 100 mM TMAA (pH 6.8).*

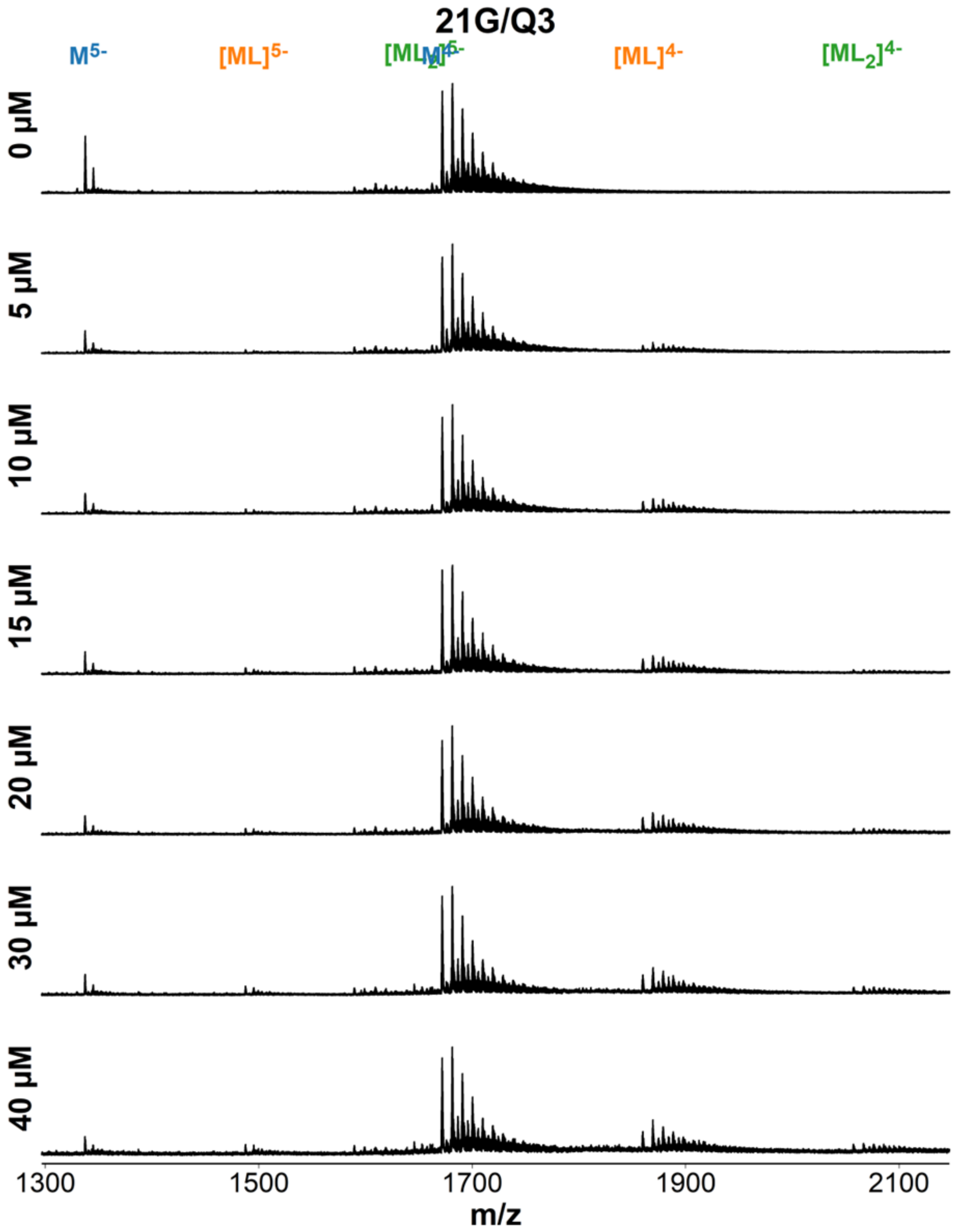

*Figure S91. ESI-MS titration of 21G (d**GGG**TTA**GGG**TTA**GGG**TTA**GGG**) with foldamer QQQ. Samples contain 10 µM DNA, 0-40 µM ligand, 0.5 mM KCl, 100 mM TMAA (pH 6.8).*

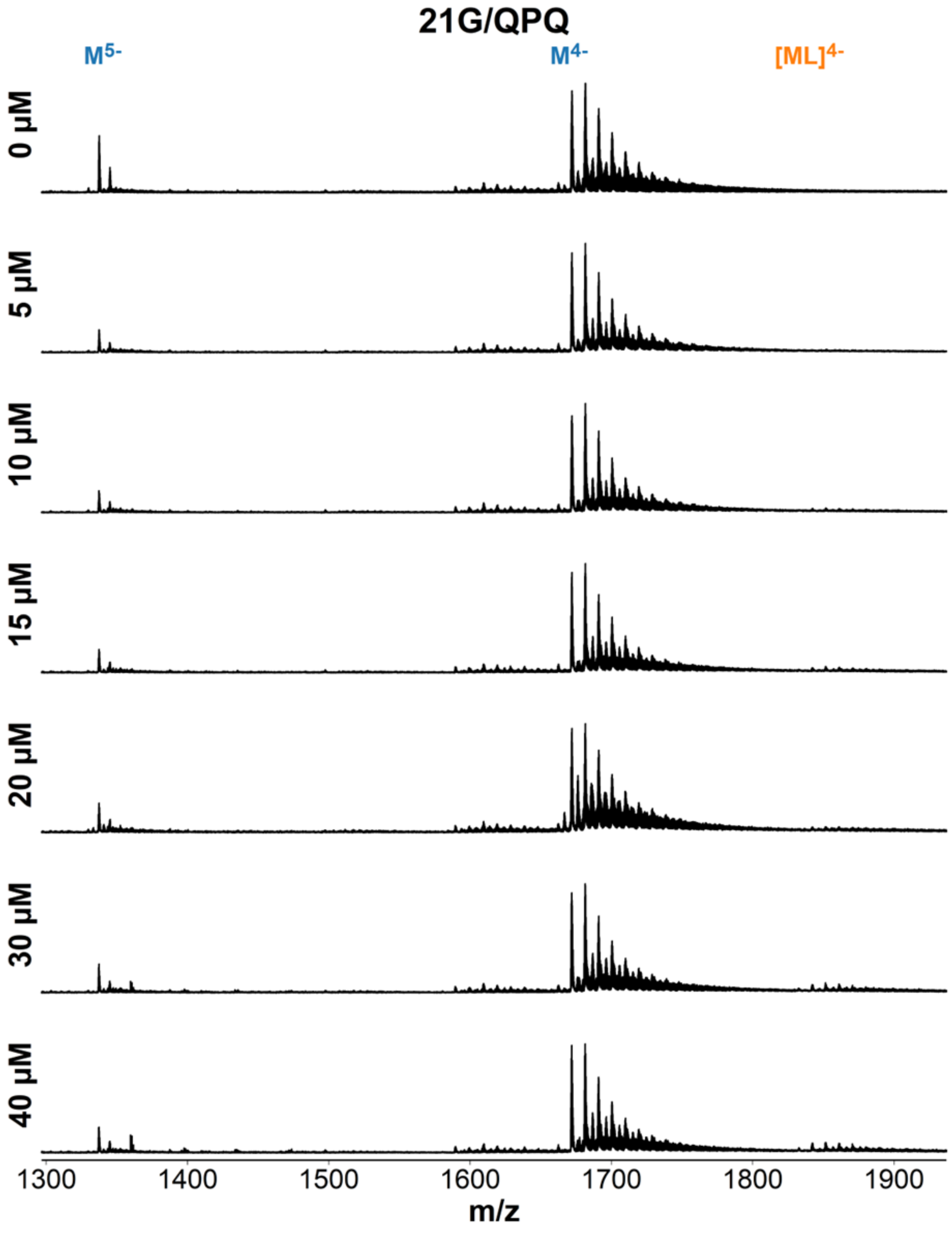

*Figure S92. ESI-MS titration of 21G (d**GGG**TTA**GGG**TTA**GGG**TTA**GGG**) with foldamer QPQ. Samples contain 10 µM DNA, 0-40 µM ligand, 0.5 mM KCl, 100 mM TMAA (pH 6.8).*

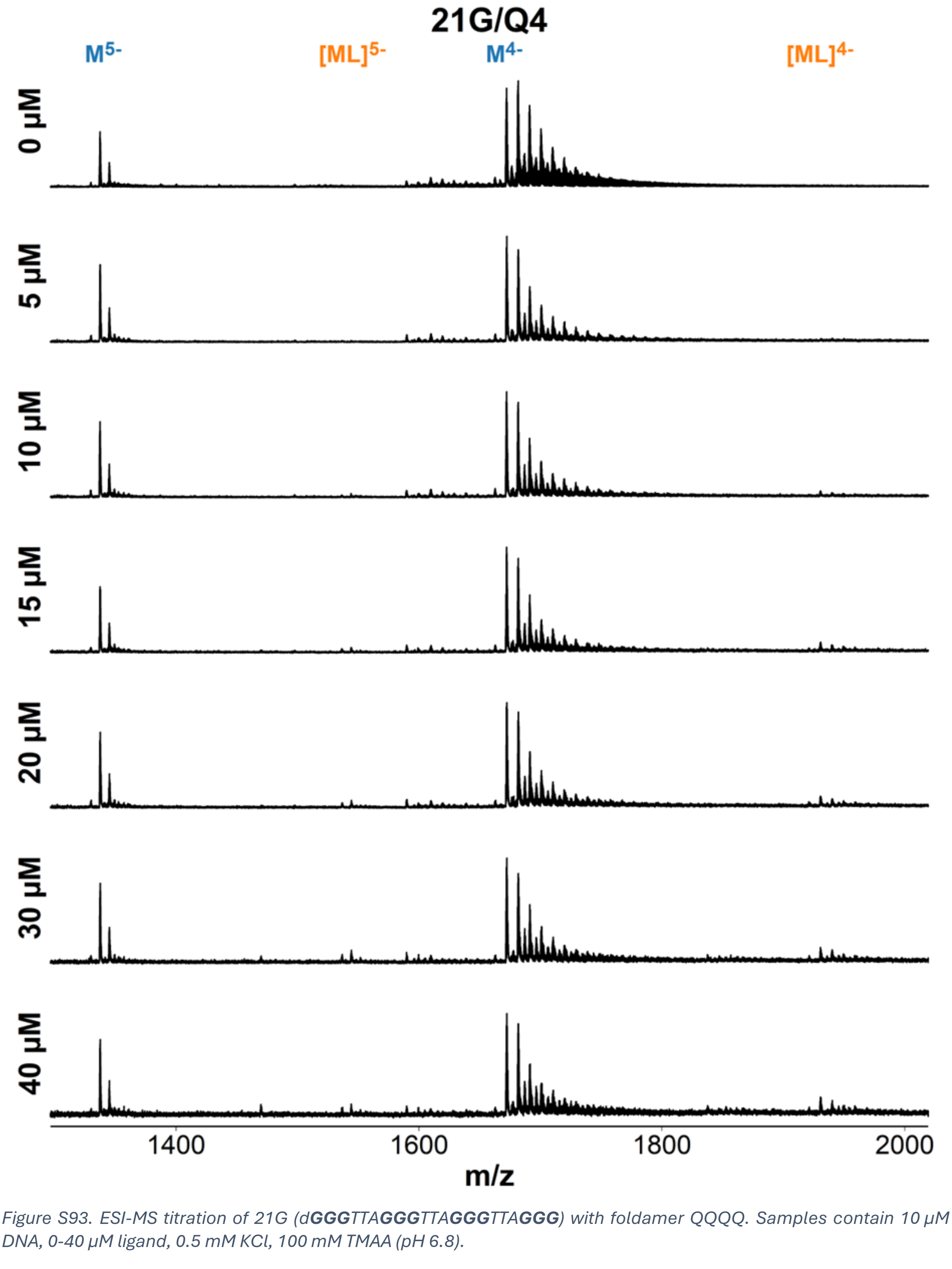

*Figure S93. ESI-MS titration of 21G (d**GGG**TTA**GGG**TTA**GGG**TTA**GGG**) with foldamer QQQQ. Samples contain 10 µM DNA, 0-40 µM ligand, 0.5 mM KCl, 100 mM TMAA (pH 6.8).*

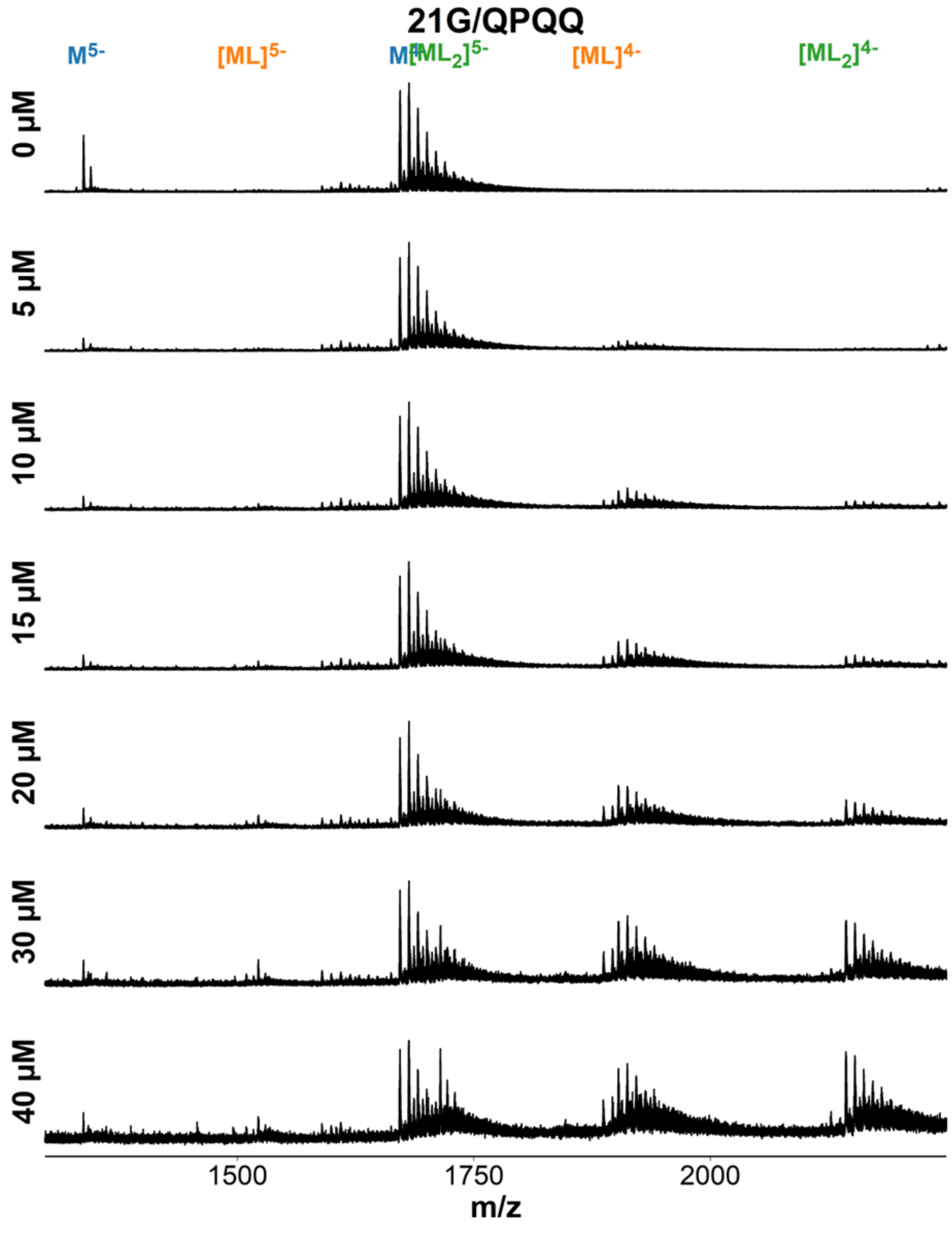

*Figure S94. ESI-MS titration of 21G (d**GGG**TTA**GGG**TTA**GGG**TTA**GGG**) with foldamer QQPQ. Samples contain 10 µM DNA, 0-40 µM ligand, 0.5 mM KCl, 100 mM TMAA (pH 6.8).*

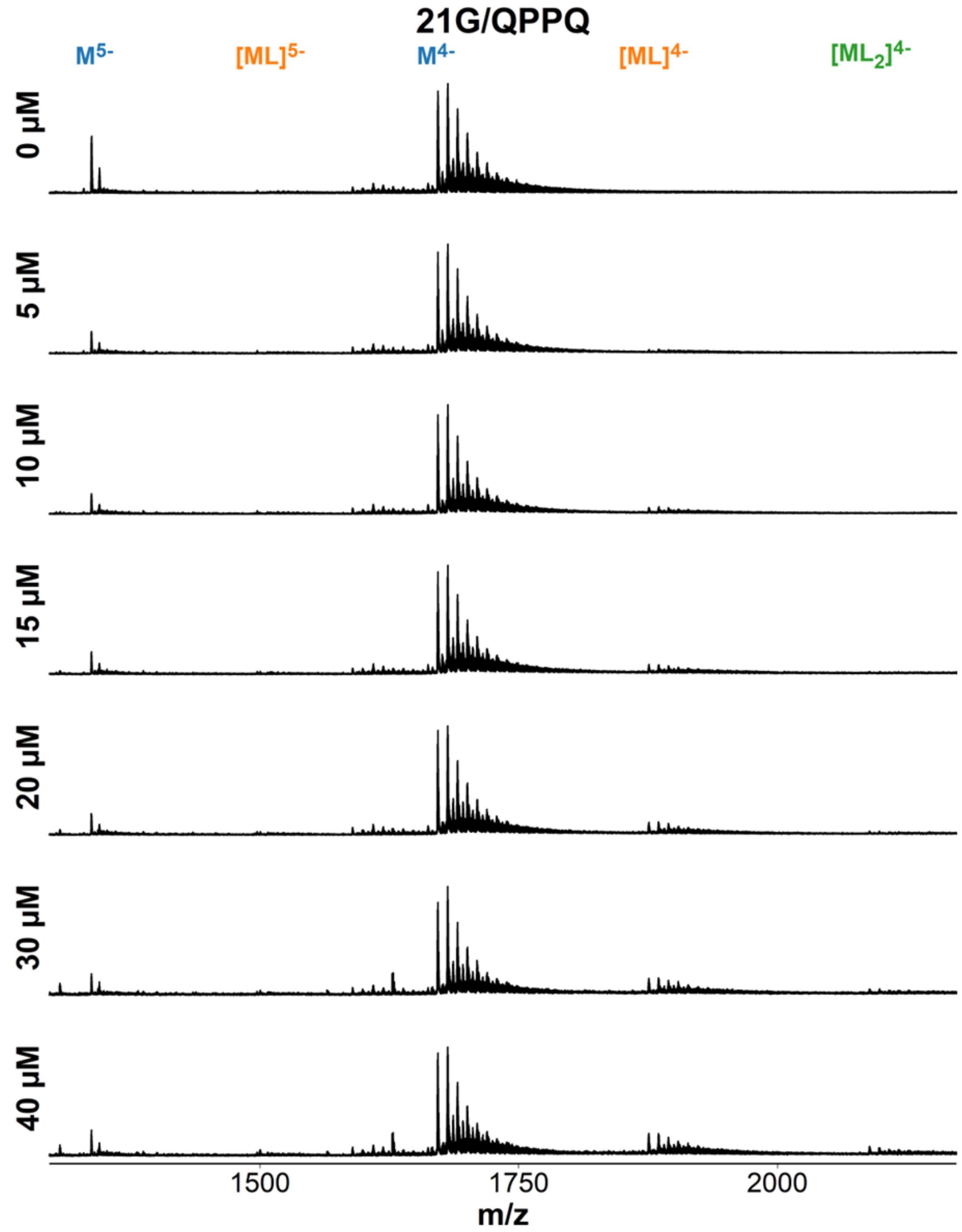

*Figure S95. ESI-MS titration of 21G (d**GGG**TTA**GGG**TTA**GGG**TTA**GGG**) with foldamer QPPQ. Samples contain 10 µM DNA, 0-40 µM ligand, 0.5 mM KCl, 100 mM TMAA (pH 6.8).*

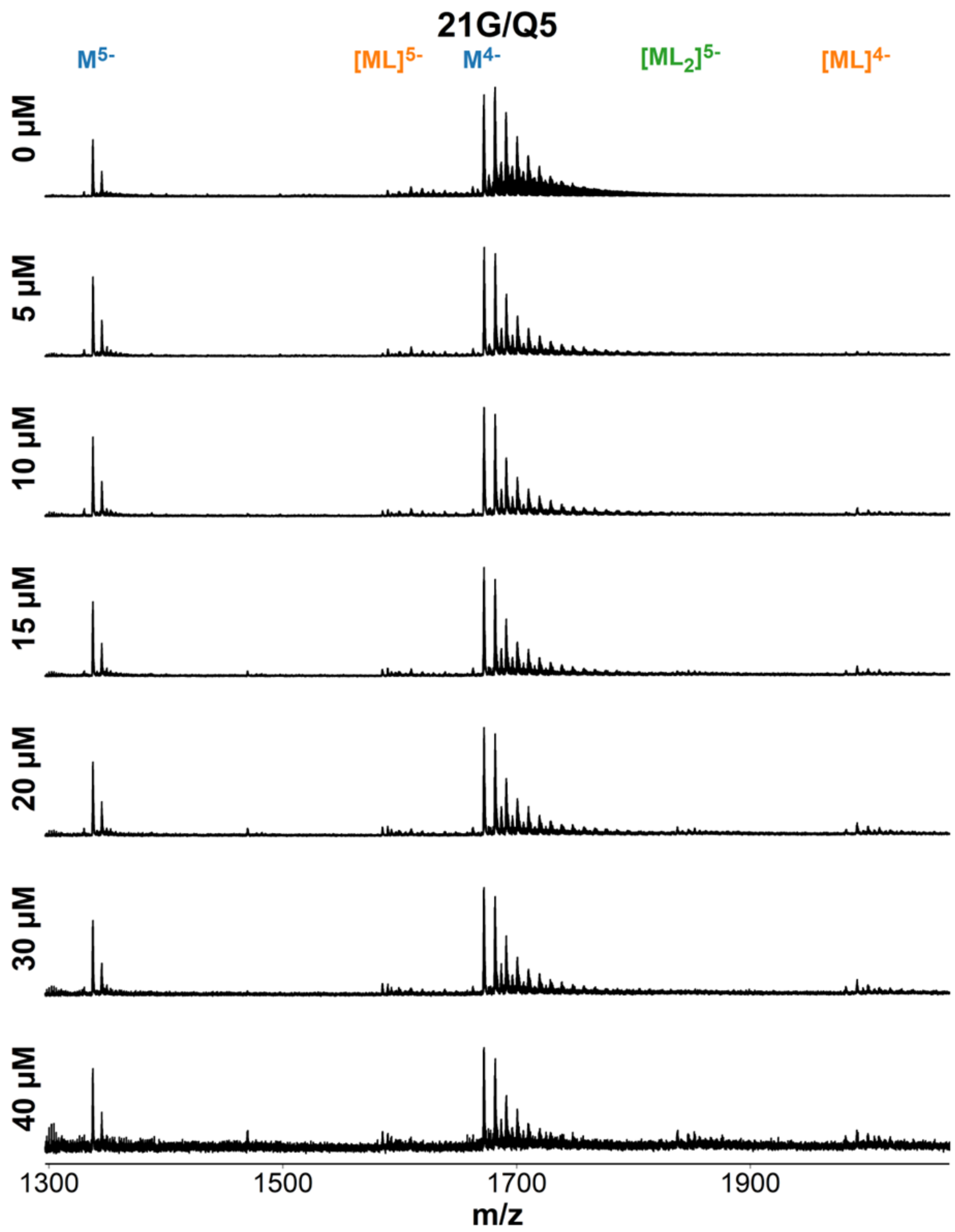

*Figure S96. ESI-MS titration of 21G (d**GGG**TTA**GGG**TTA**GGG**TTA**GGG**) with foldamer QQQQQ. Samples contain 10 µM DNA, 0-40 µM ligand, 0.5 mM KCl, 100 mM TMAA (pH 6.8).*

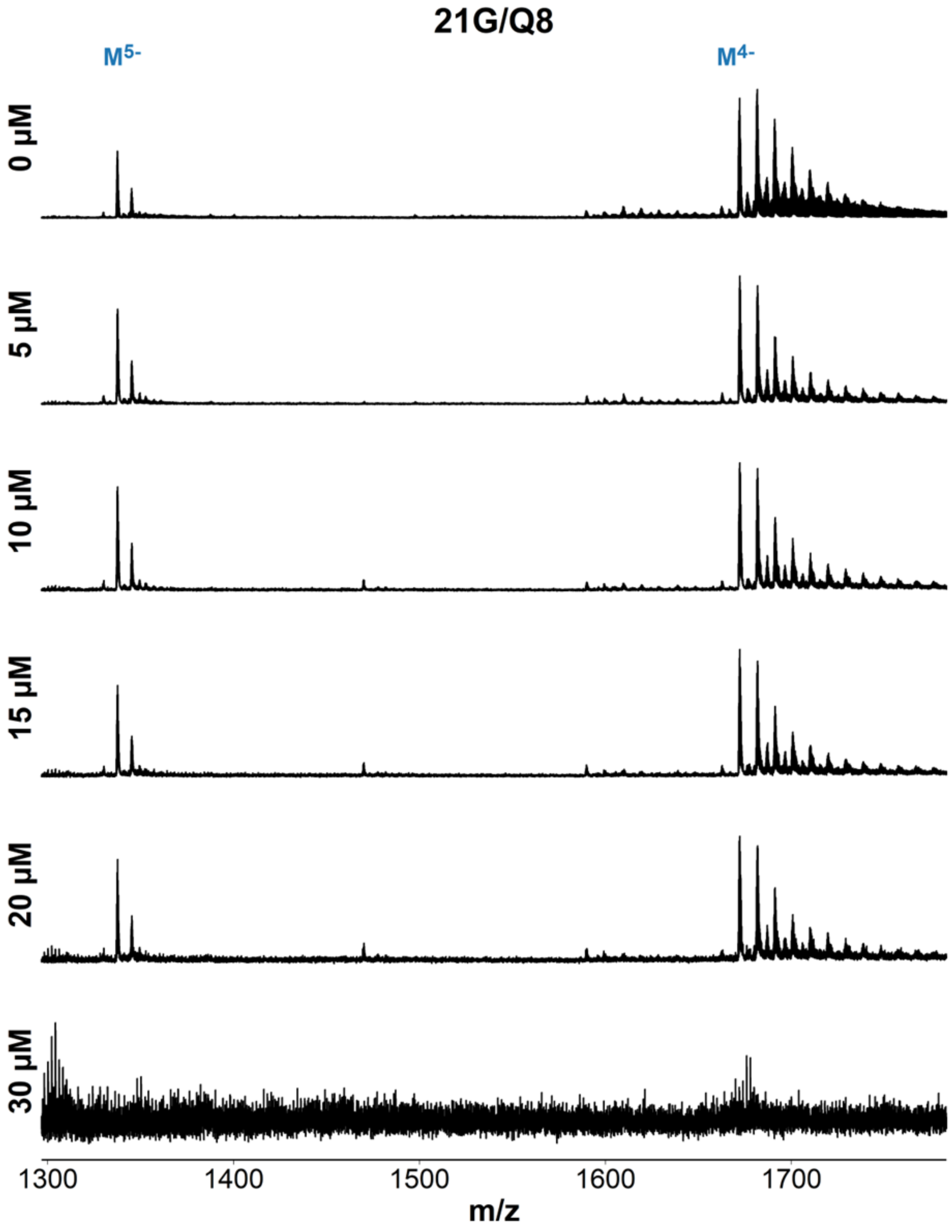

*Figure S97. ESI-MS titration of 21G (d**GGG**TTA**GGG**TTA**GGG**TTA**GGG**) with foldamer QQQQQQQQ. Samples contain 10 µM DNA, 0-30 µM ligand, 0.5 mM KCl, 100 mM TMAA (pH 6.8).*

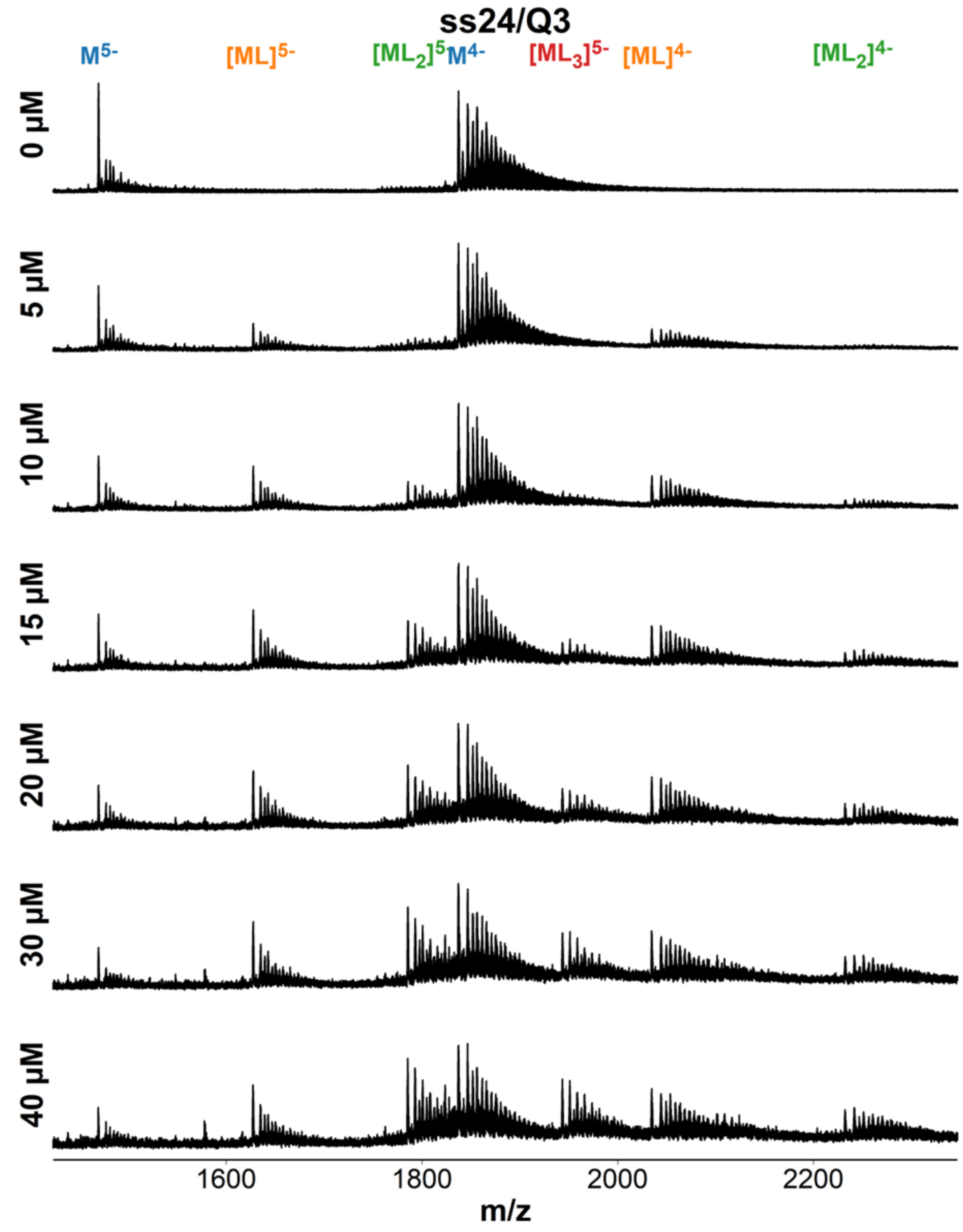

Figure S98. ESI-MS titration of ss24 (dTGCCATGCTACTGAGATGACGCTA) with foldamer QQQ. Samples contain 10 µM DNA, 0-40 µM ligand, 0.5 mM KCl, 100 mM TMAA (pH 6.8).

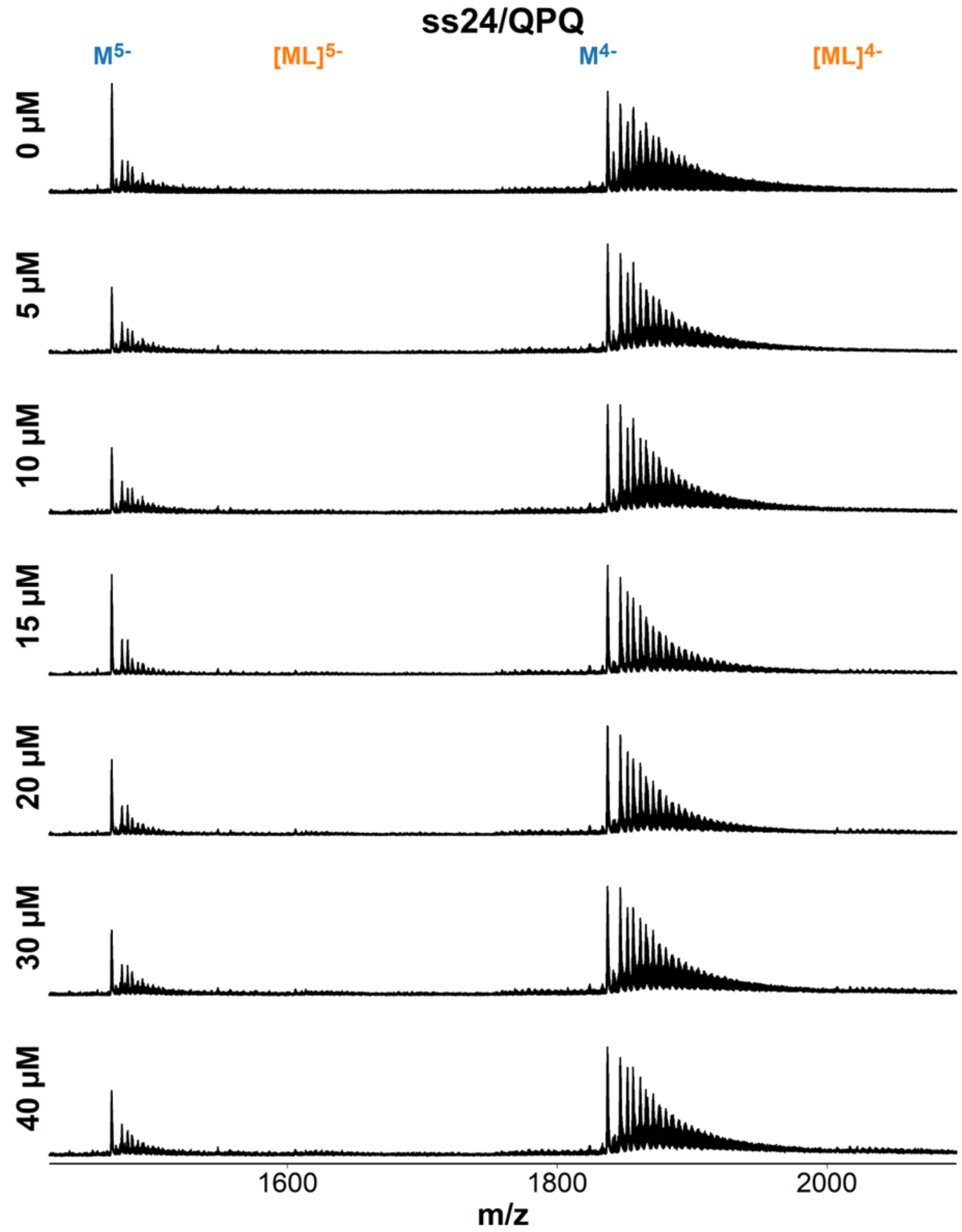

Figure S99. ESI-MS titration of ss24 (dTGCCATGCTACTGAGATGACGCTA) with foldamer QPQ. Samples contain 10 µM DNA, 0-40 µM ligand, 0.5 mM KCl, 100 mM TMAA (pH 6.8).

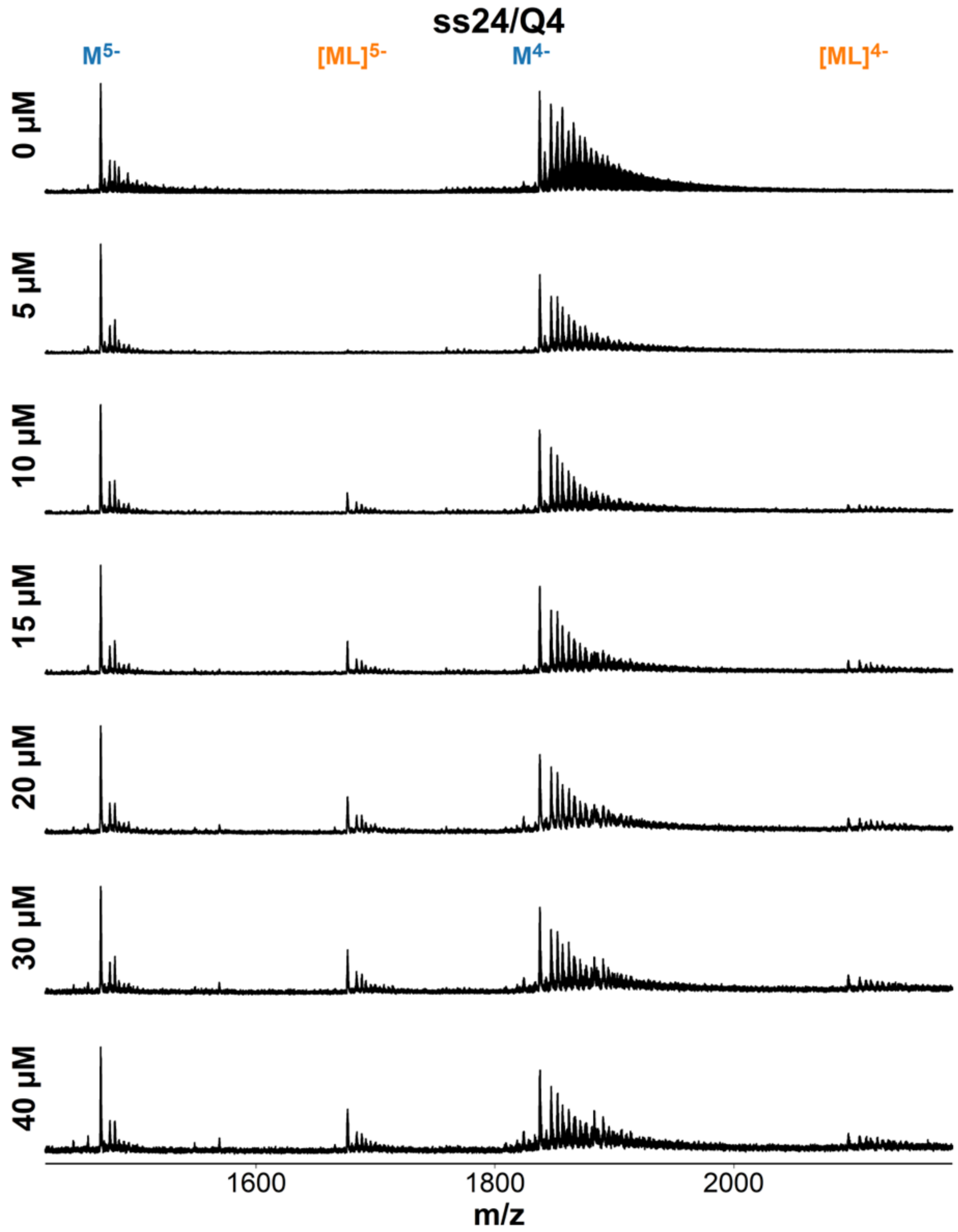

Figure S100. ESI-MS titration of ss24 (dTGCCATGCTACTGAGATGACGCTA) with foldamer QQQQ. Samples contain 10 µM DNA, 0-40 µM ligand, 0.5 mM KCl, 100 mM TMAA (pH 6.8).

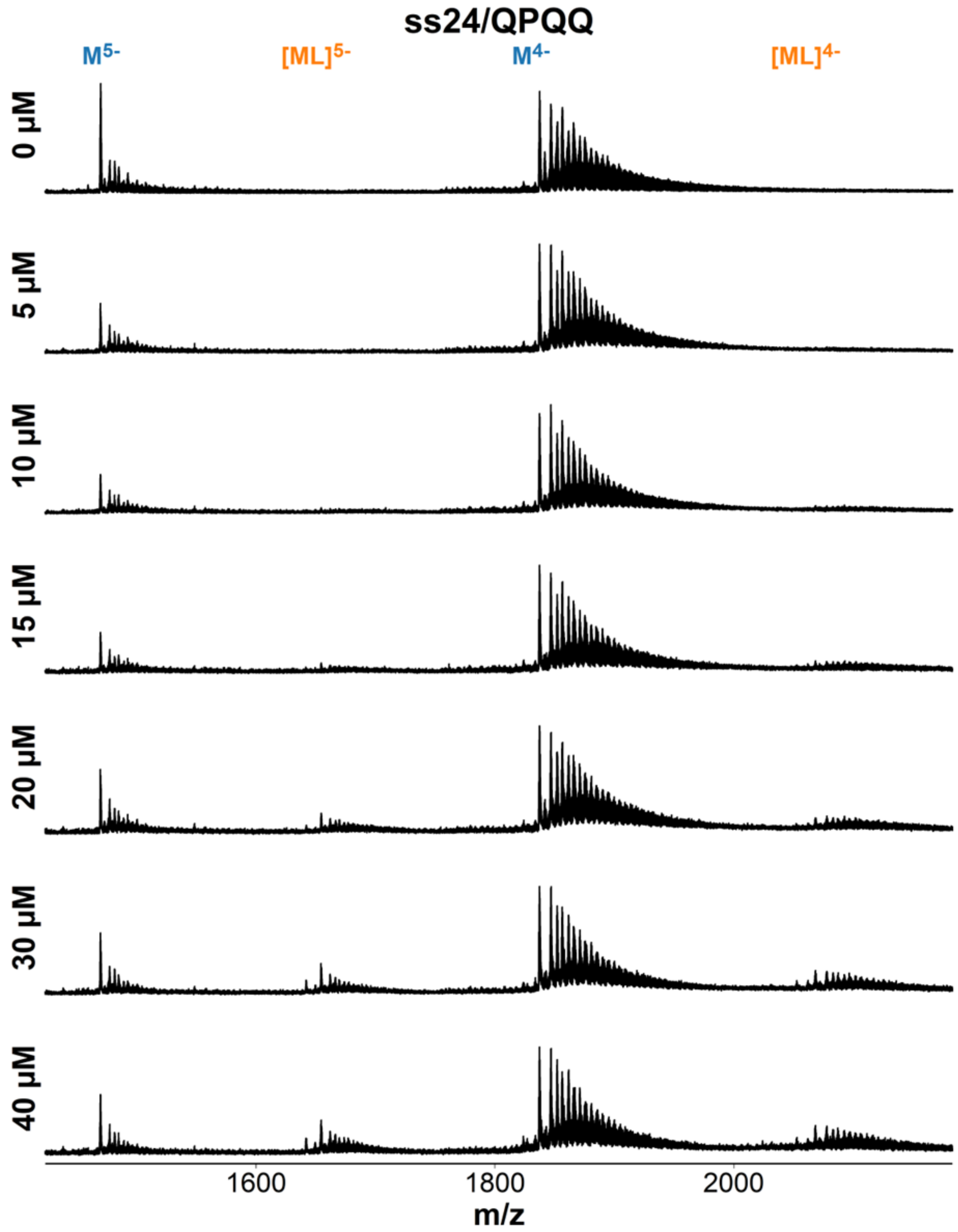

Figure S101. ESI-MS titration of ss24 (dTGCCATGCTACTGAGATGACGCTA) with foldamer QQPQ. Samples contain 10 µM DNA, 0-40 µM ligand, 0.5 mM KCl, 100 mM TMAA (pH 6.8).

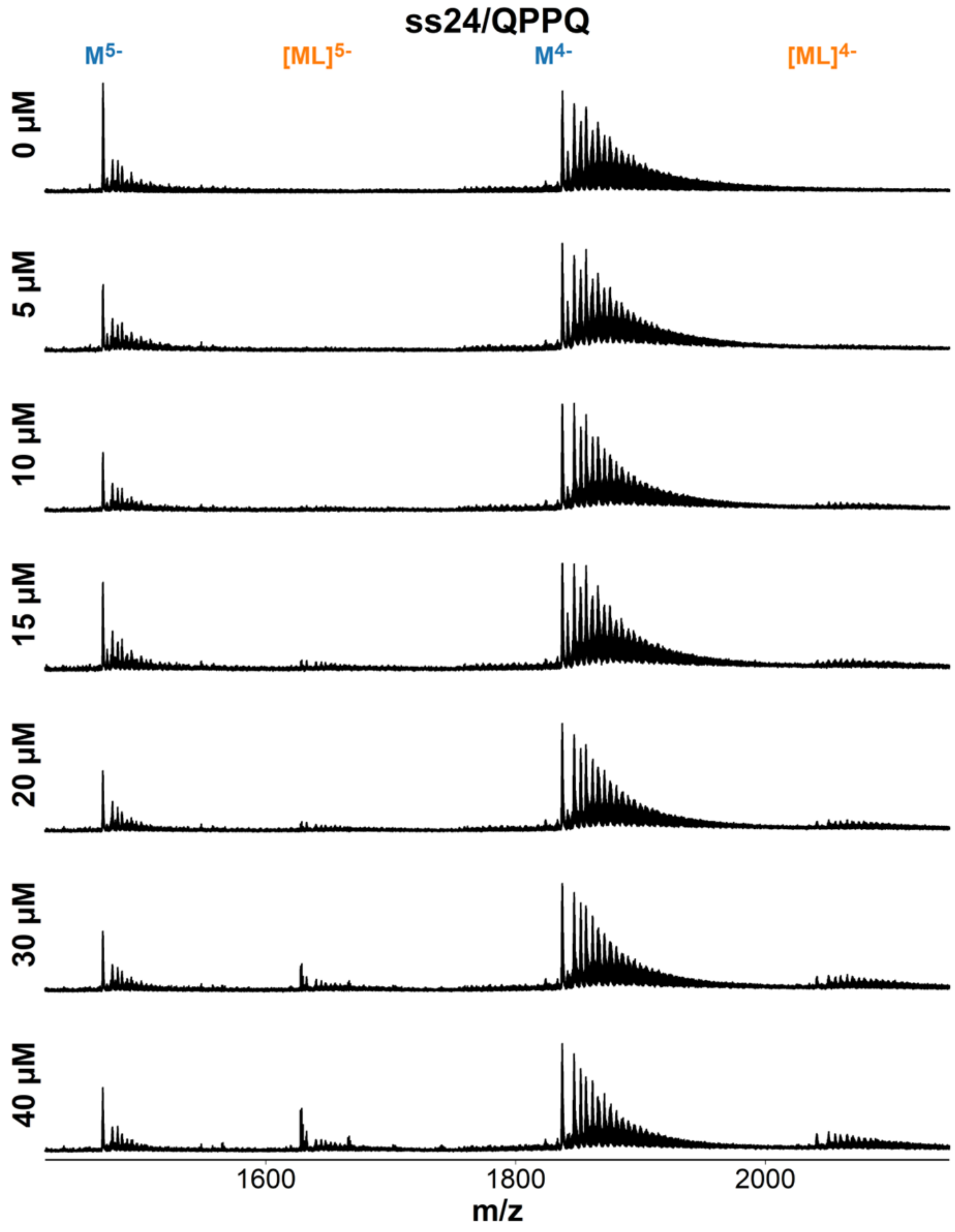

Figure S102. ESI-MS titration of ss24 (dTGCCATGCTACTGAGATGACGCTA) with foldamer QPPQ. Samples contain 10 µM DNA, 0-40 µM ligand, 0.5 mM KCl, 100 mM TMAA (pH 6.8).

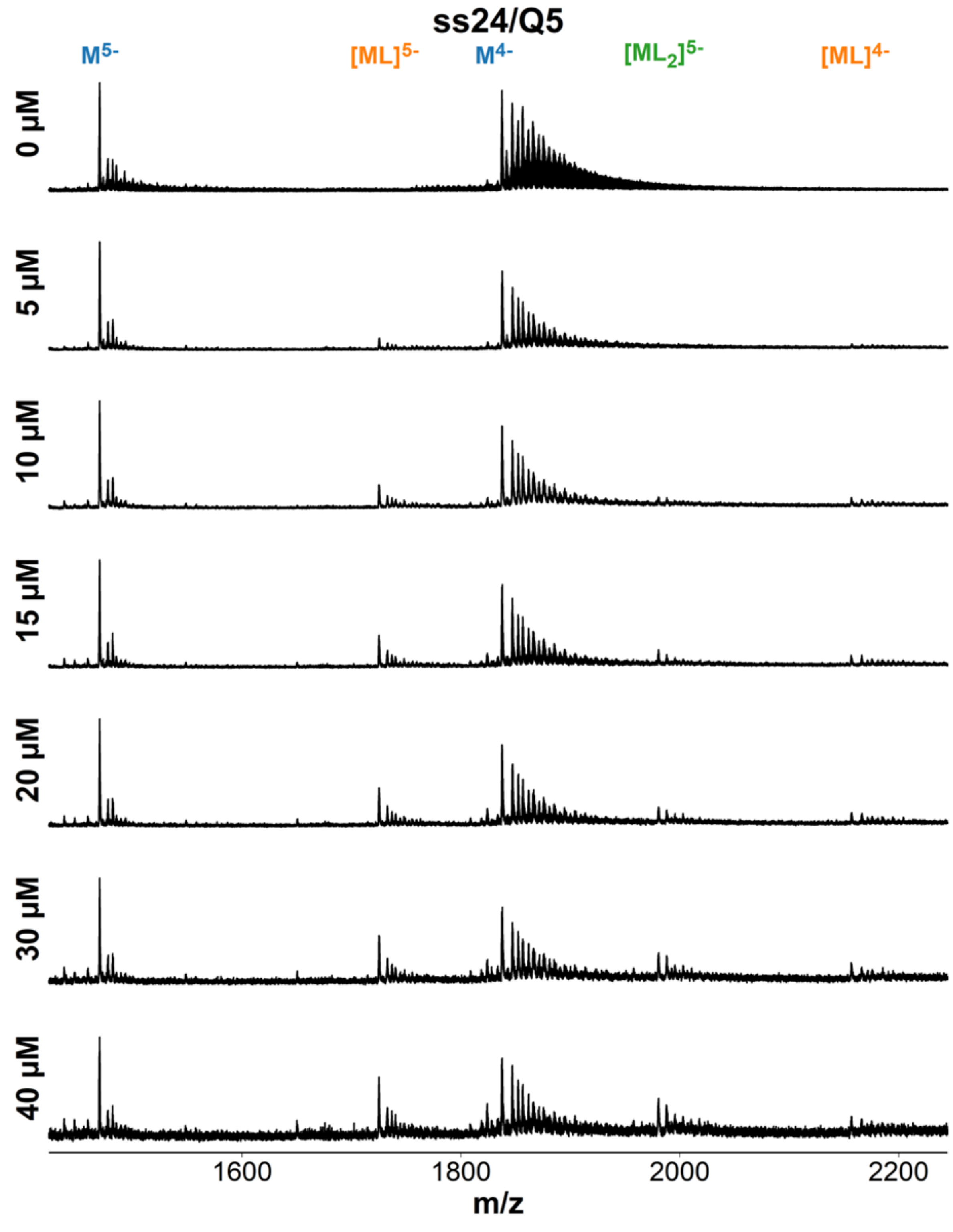

Figure S103. ESI-MS titration of ss24 (dTGCCATGCTACTGAGATGACGCTA) with foldamer QQQQQ. Samples contain 10 µM DNA, 0-40 µM ligand, 0.5 mM KCl, 100 mM TMAA (pH 6.8).

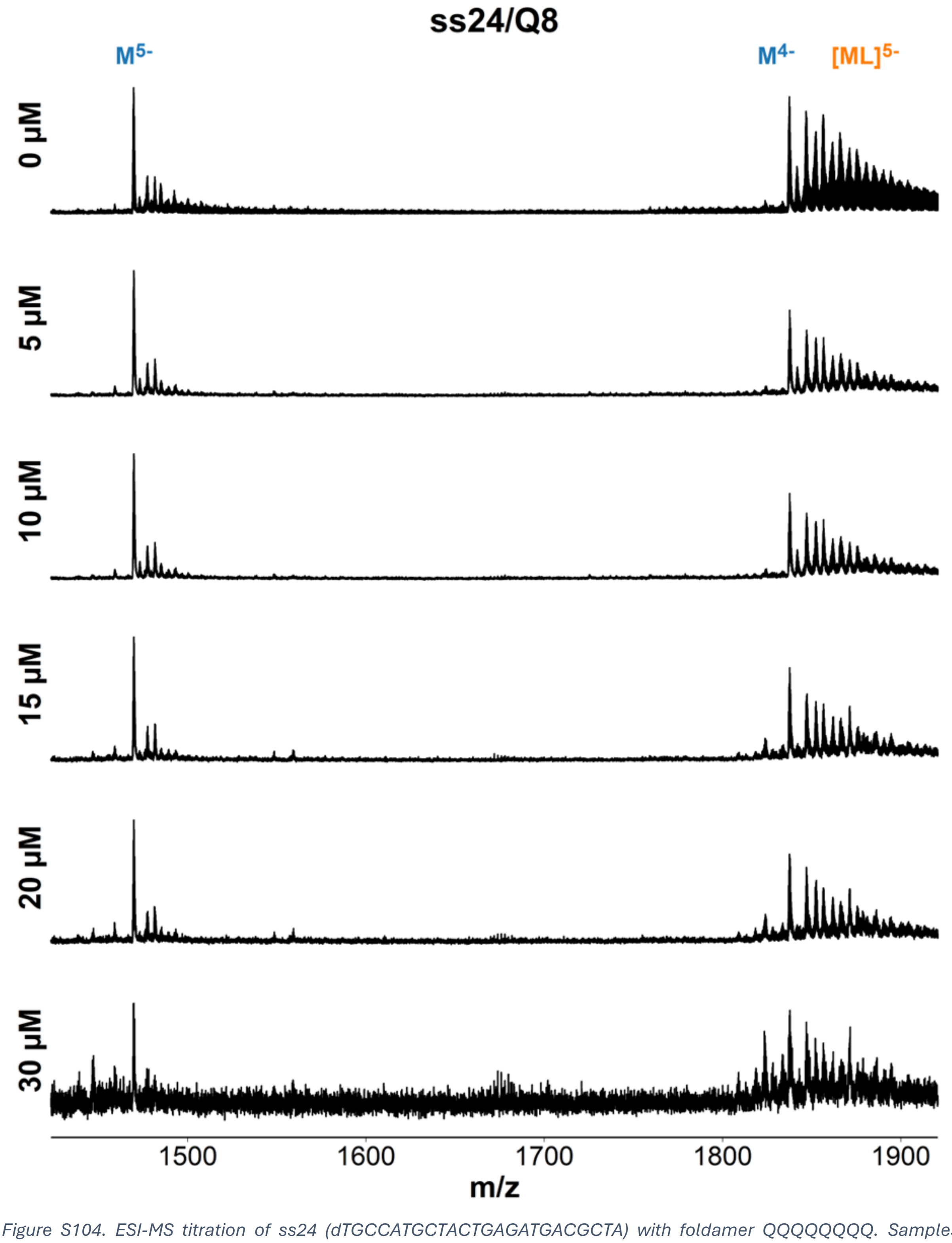

Figure S104. ESI-MS titration of ss24 (dTGCCATGCTACTGAGATGACGCTA) with foldamer QQQQQQQQ. Samples contain 10 µM DNA, 0-30 µM ligand, 0.5 mM KCl, 100 mM TMAA (pH 6.8).

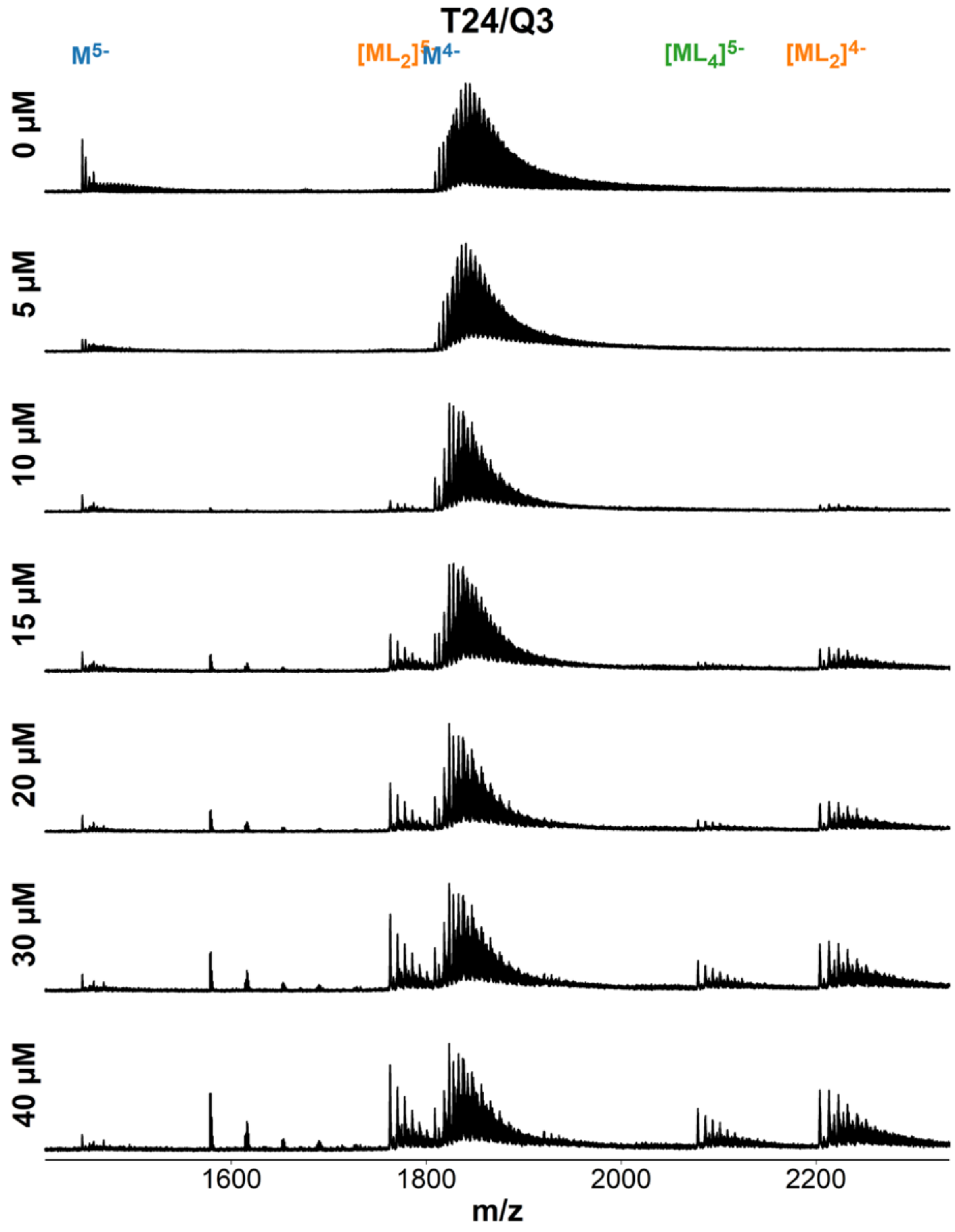

Figure S105. ESI-MS titration of T24 (dTTTTTTTTTTTTTTTTTTTTTTTT) with foldamer QQQ. Samples contain 10 µM DNA, 0-40 µM ligand, 0.5 mM KCl, 100 mM TMAA (pH 6.8).

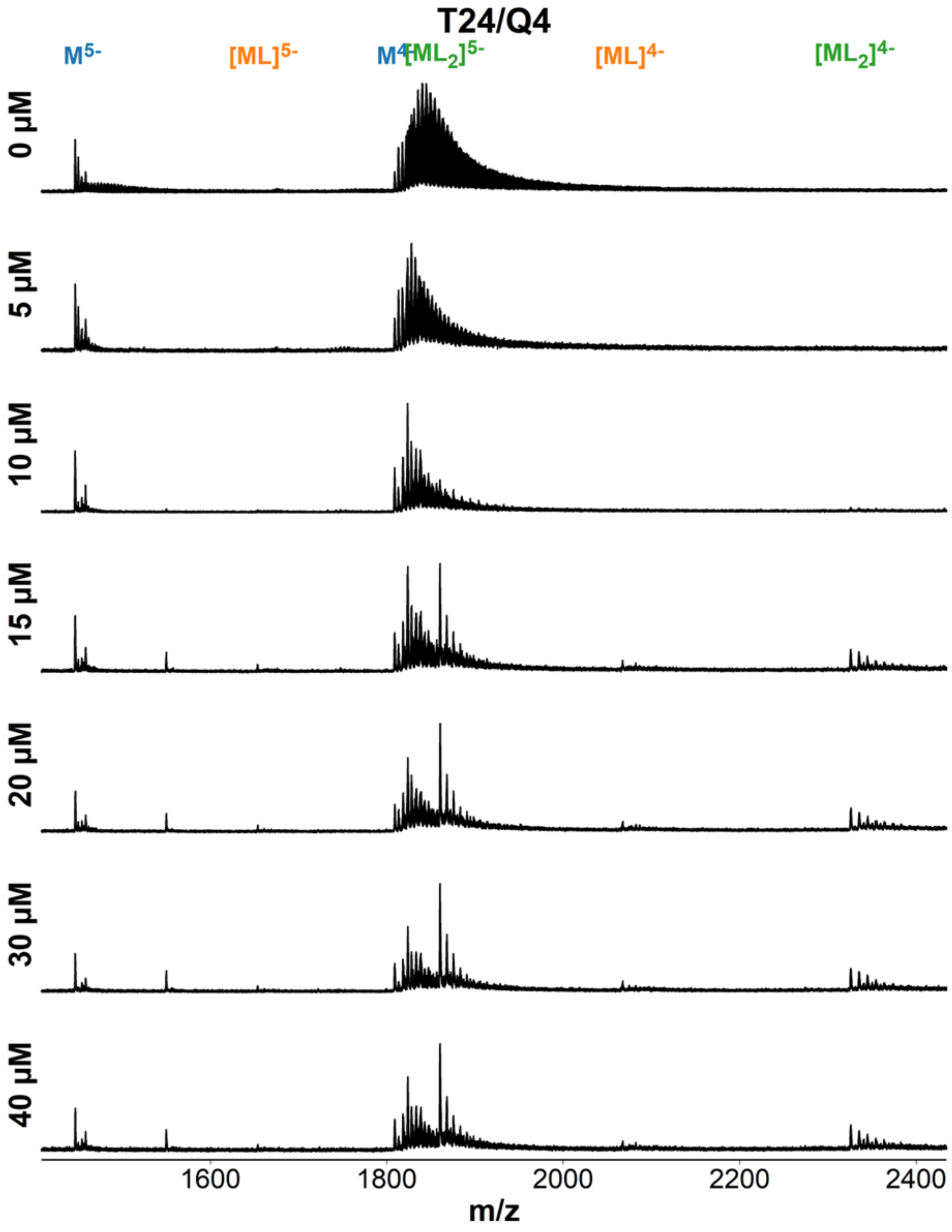

Figure S106. ESI-MS titration of T24 (dTTTTTTTTTTTTTTTTTTTTTTTT) with foldamer QQQQ. Samples contain 10 µM DNA, 0-40 µM ligand, 0.5 mM KCl, 100 mM TMAA (pH 6.8).

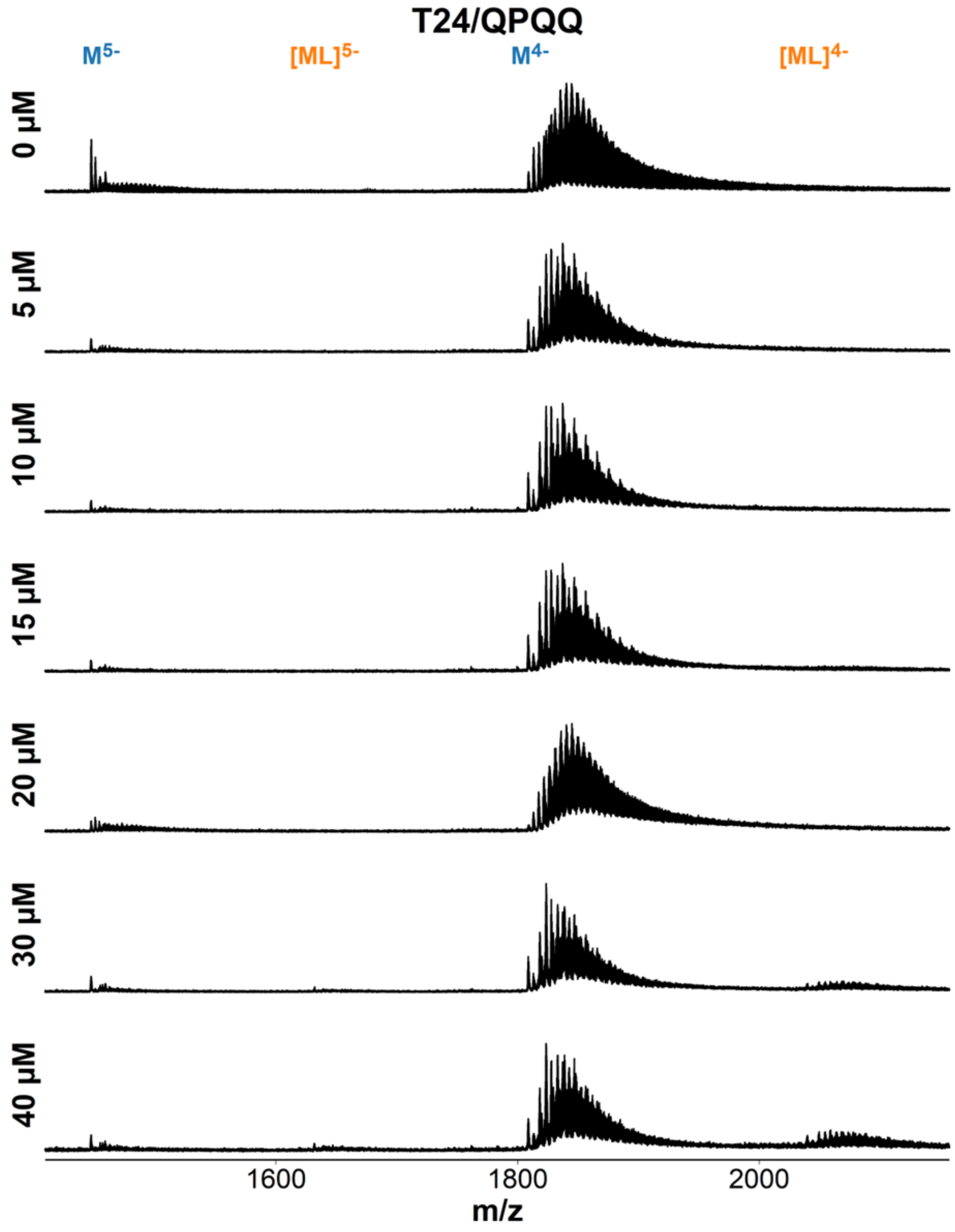

Figure S107. ESI-MS titration of T24 (dTTTTTTTTTTTTTTTTTTTTTTTT) with foldamer QQPQ. Samples contain 10 µM DNA, 0-40 µM ligand, 0.5 mM KCl, 100 mM TMAA (pH 6.8).

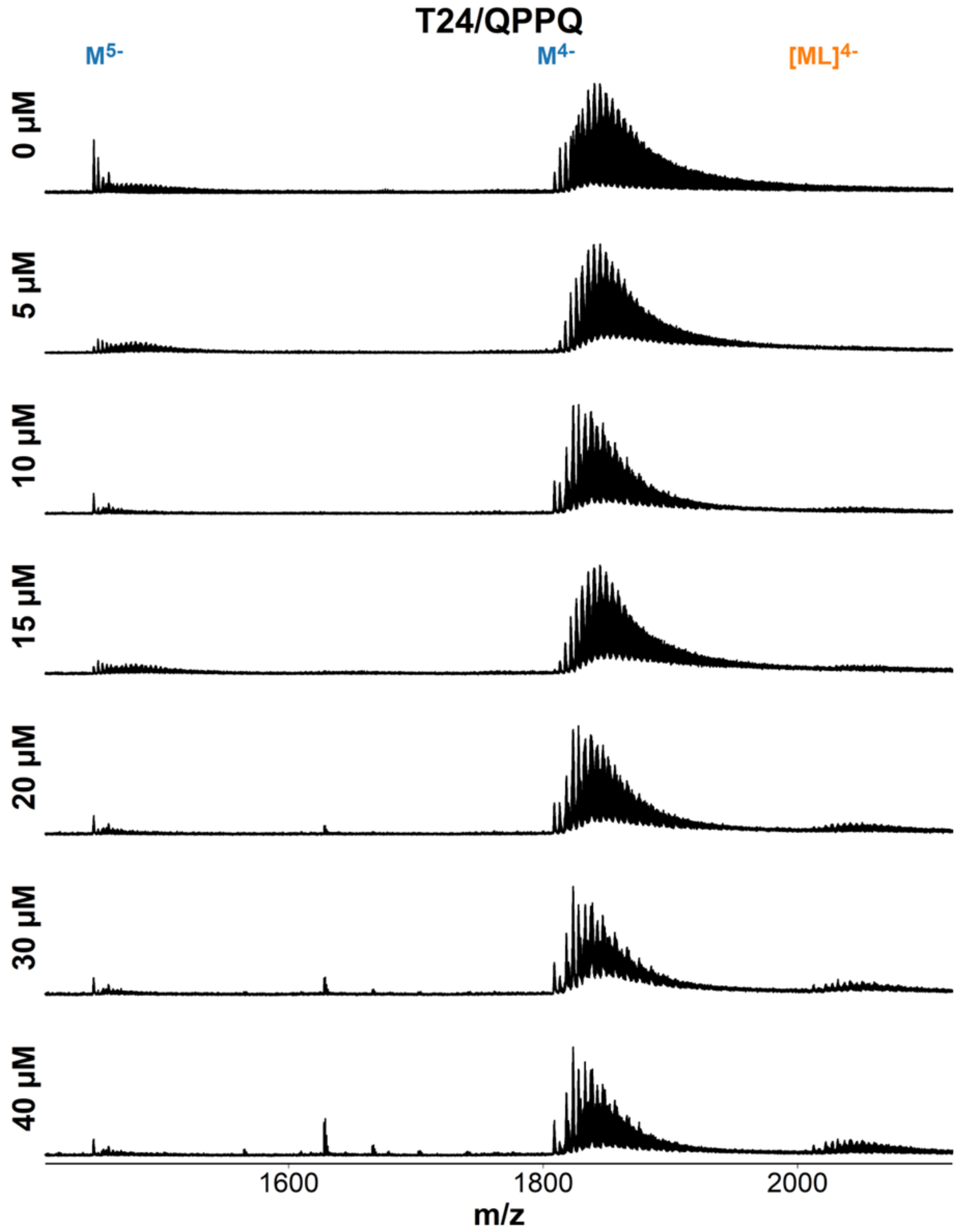

Figure S108. ESI-MS titration of T24 (dTTTTTTTTTTTTTTTTTTTTTTTT) with foldamer QPPQ. Samples contain 10 µM DNA, 0-40 µM ligand, 0.5 mM KCl, 100 mM TMAA (pH 6.8).

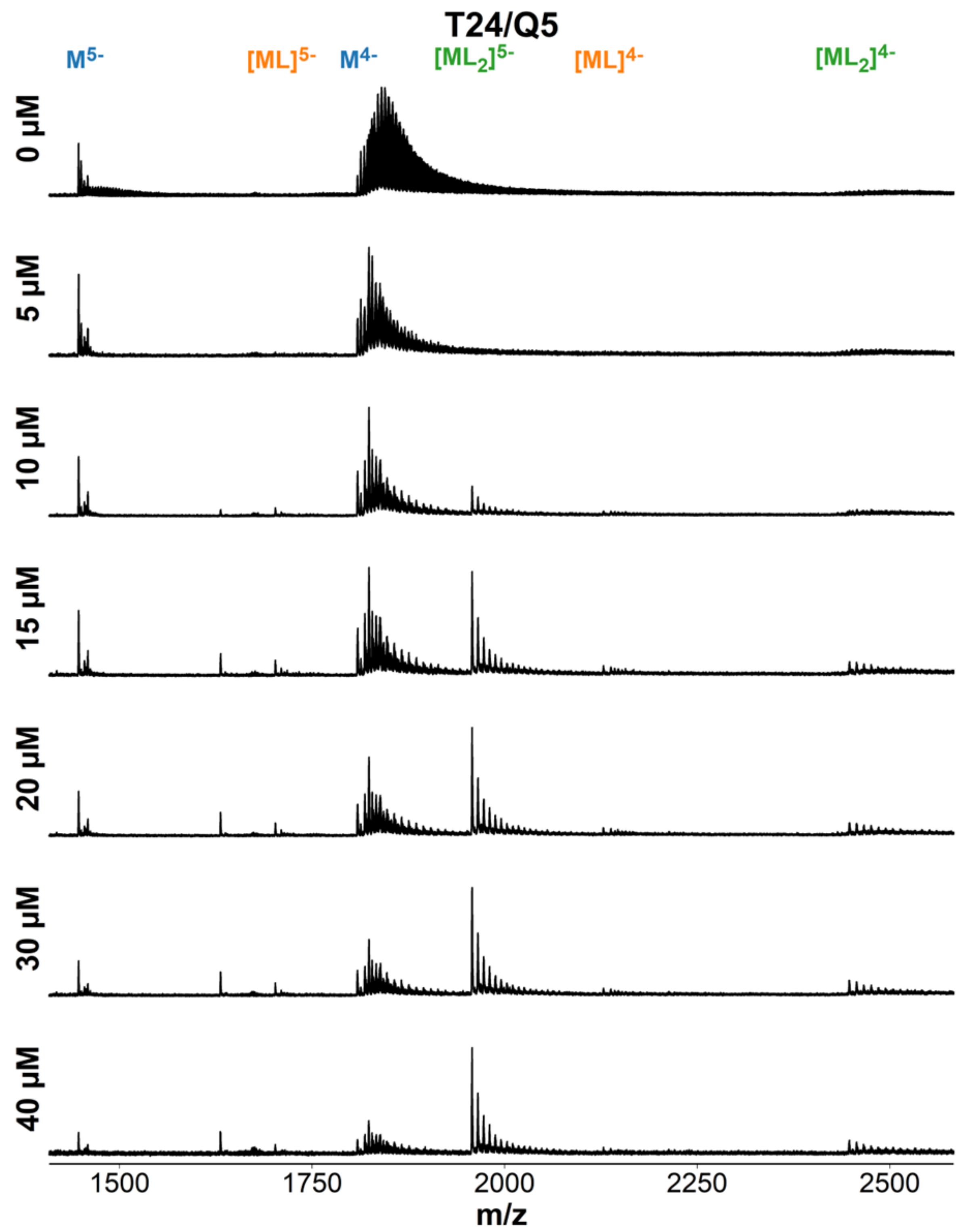

Figure S109. ESI-MS titration of T24 (dTTTTTTTTTTTTTTTTTTTTTTTT) with foldamer QQQQQ. Samples contain 10 µM DNA, 0-40 µM ligand, 0.5 mM KCl, 100 mM TMAA (pH 6.8).

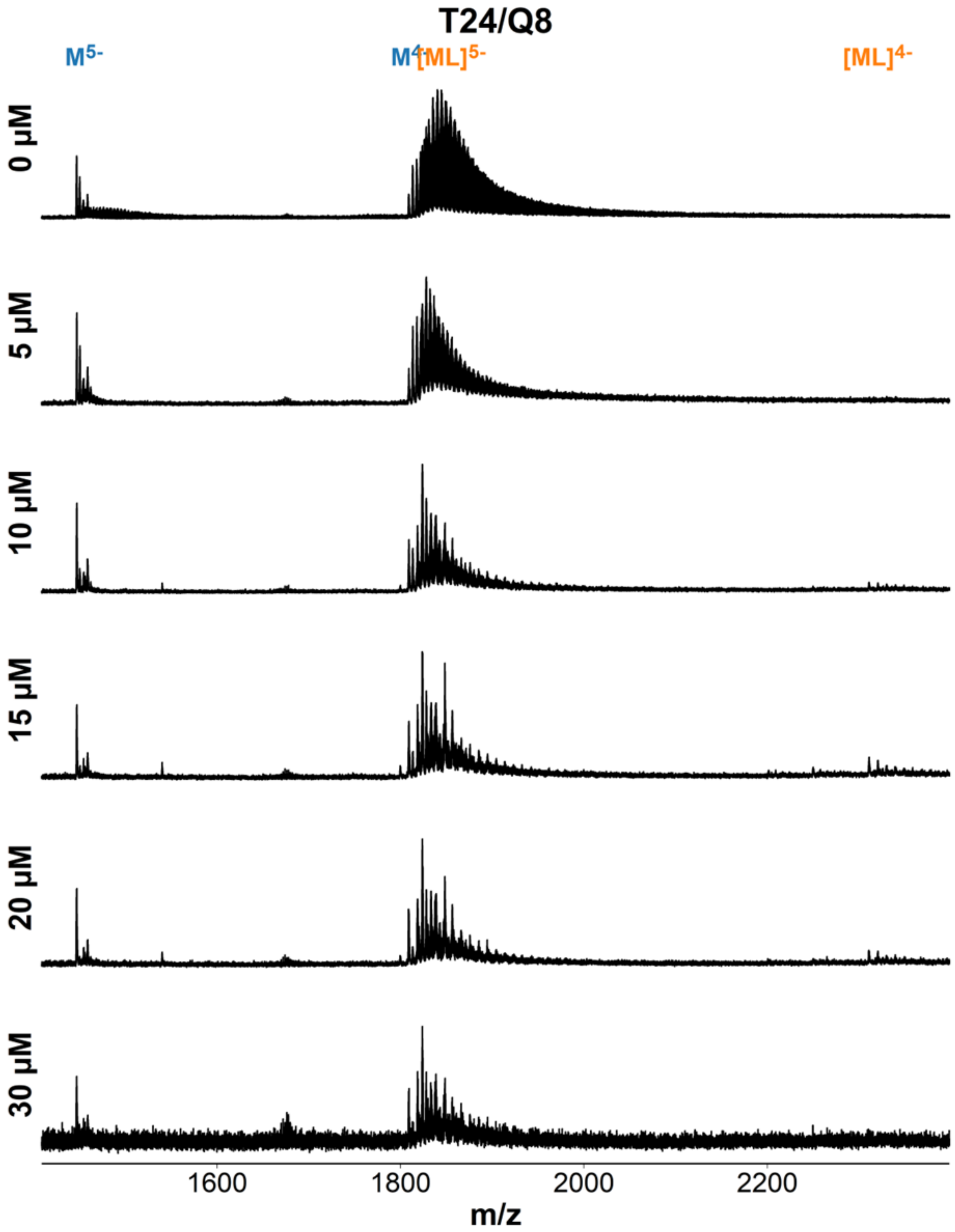

Figure S110. ESI-MS titration of T24 (dTTTTTTTTTTTTTTTTTTTTTTTT) with foldamer QQQQQQQQ. Samples contain 10 µM DNA, 0-30 µM ligand, 0.5 mM KCl, 100 mM TMAA (pH 6.8).

T24 induces CD on Q_n-type foldamers

Among our ligand screening results, we noticed that Q-mers (i.e. Q_3, Q_4, Q_5, Q_8) form high-affinity 2:1 complexes (low µM K_D) with single-stranded sequence T24. We investigated this phenomenon via CD (Figure S111).

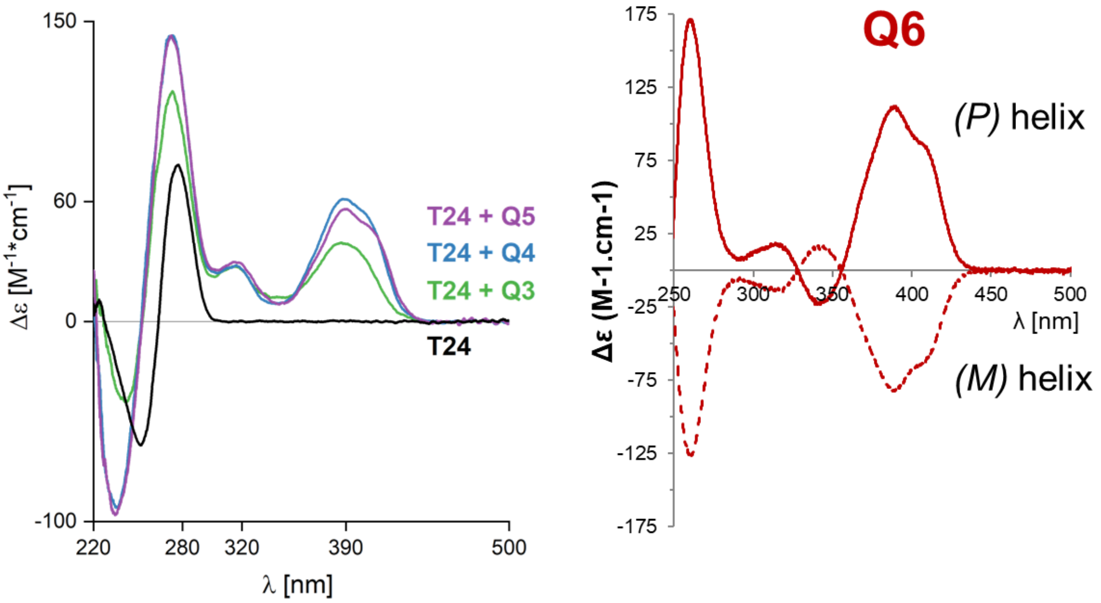

*Figure S111. **Left**: CD signatures of dT_{24} with different Q-mer foldamers. Samples contain: 10 µM T24, 40 µM Q_3/30 µM Q_4/24 µM Q_5 (normalized to 120 µM Q monomer) and 100 mM TMAA (pH 6.8). **Right**: Enantiomer-separated CD signatures of the (M) and (P) helix for Q6.*

In absence of T24, Q_3/Q_4/Q_5 have no CD signature, since they are racemic. But with T24, we see CD bands that are consistent with the *(P)*-helix. We do not know that kind of structure T24 and Q-mer foldamers form; our attempt of crystallizing a T24/Q_4 complex was unsuccessful. Based on the high 2:1 cooperativity and the induced CD on the (right-handed) *(P)*-helix we speculate that T24 and Q_n foldamers associate into some sort of double helix, with the foldamer dimerizing to accommodate the full length of the DNA sequence.

U-rich motifs play a role in RNA expression,[8,9] which is why we attempted to reproduce our results on U24 (Figure S112). Alas, we could not see the same induced CD when switching from dT to rU.

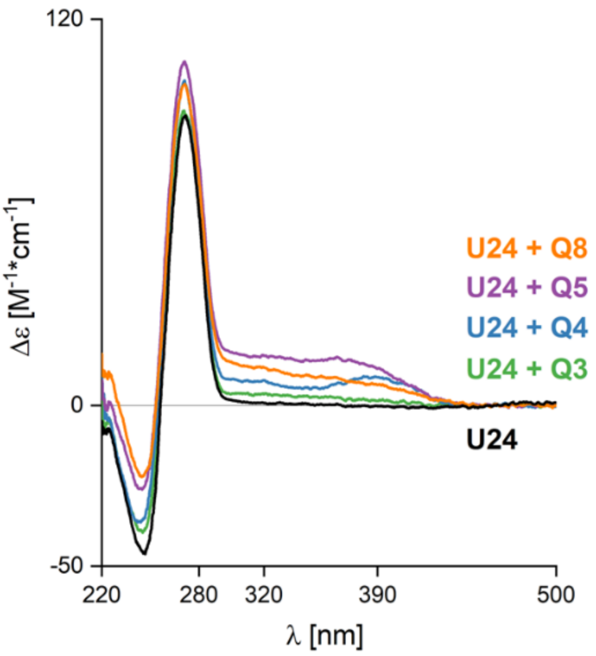

Figure S112. CD signatures of rU$_{24}$ with different Q-mer foldamers. Samples contain: 10 µM U24, 40 µM Q$_3$/30 µM Q$_4$/24 µM Q$_5$/15 µM Q$_8$ (normalized to 120 µM Q monomer) and 100 mM TMAA (pH 6.8).

Foldamer-induced disruption of G-quadruplex

Figure S32 shows that the decline in MS signal response of the G4:foldamer complexes is proportional to the number of Q units in the foldamer. Each Q unit carries a positive charge, so we suspect that the loss of signal is due to ligand-induced DNA aggregation, with the ligand acting as a sort of flocculant that helps overcome charge repulsions between DNA polyanions. To test this hypothesis, we picked T30177TT, a parallel G-quadruplex that is stable even in low K^+ and Q8, the ligand with the fastest decrease in response factor. If the ligand causes DNA aggregation, the CD signature of T30177TT should decrease the more ligand is added.

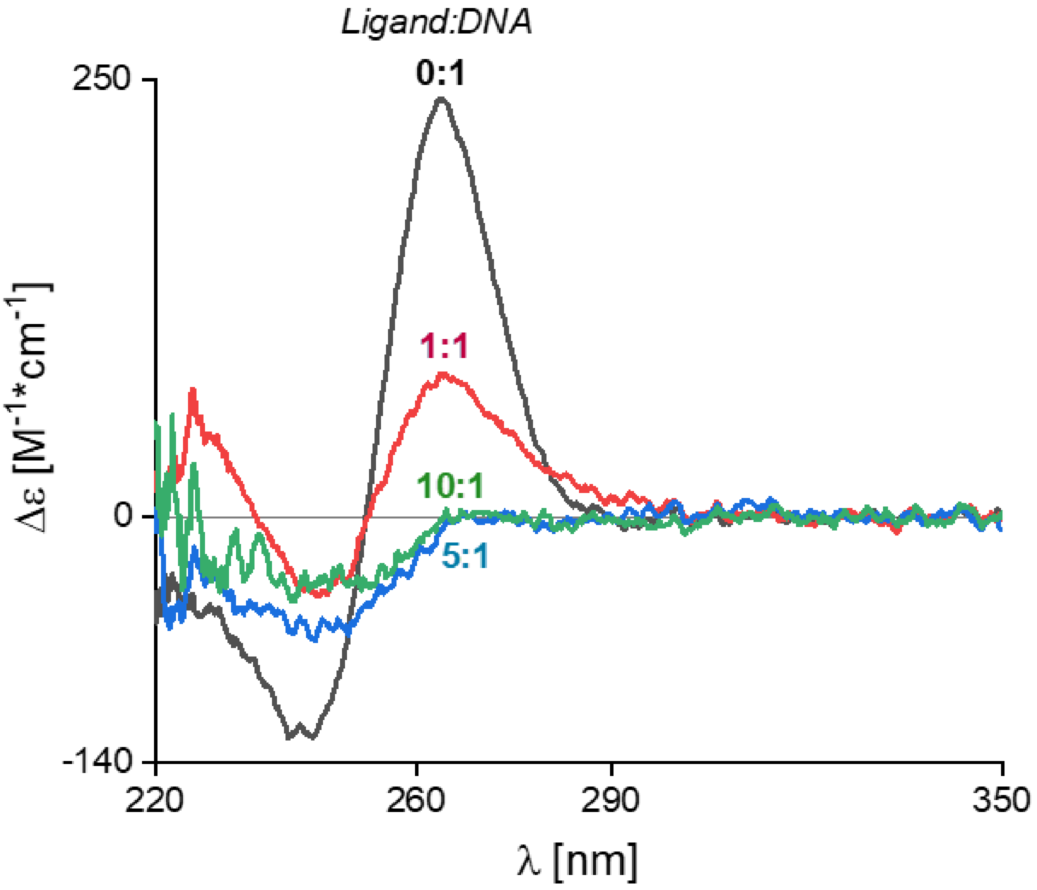

*Figure S113. Tracking the ligand-induced aggregation of T30177TT (dTT**G**T**GG**T**GGG**T**GGG**T**GGG**T) by adding more and more Q8. Samples contain 1 µM DNA, 0/1/5/10 µM Q_8, 1 mM KCl and 100 mM TMAA (pH 6.8).*

The experimental results are consistent with our hypothesis, indicating that G-quadruplexes with too many positive charges run the risk of causing DNA aggregation, or even precipitation (aka 'salting out' a biomolecule by neutralizing all of its charges).

X-ray crystallography

Table S5. Data acquisition and refinement parameters for the 222T/QQPQ crystal. Parenthesis show statistics for the highest-resolution shell.

Space group	P42
Unit cell (Å)	a = b = 32.71, c = 61.01, α = β = γ = 90.000°
Wavelength (Å)	0.979
Resolution range (Å)	30.5-2.512 (2.602-2.512)
Completeness (%)	99.10 (100.0)
Total reflections	29748 (2985)
Unique reflections	2226 (218)
Multiplicity	13.4 (13.7)
Average I/σ	21.61 (4.09)
Wilson B-Factor	58.79
R-merge	0.1469 (1.054)
R-means	0.1531 (1.095)
R-pim	0.0426 (0.2952)
CC ½	0.999 (0.872)
CC*	1 (0.965)
Reflections used in refinement	2207 (218)
Reflections used for R-free	221 (22)
R-work	0.2563 (0.2933)
R-free	0.3037 (0.4455)
CC (work)	0.902 (0.773)
CC (free)	0.990 (0.336)
r.m.s. bond deviation (Å)	0.018
r.m.s. angle deviation (°)	1.84
Clashscore	23.19
Number of non-hydrogen atoms	531
DNA atoms	388
QQPQ atoms	243
Other atoms (K^+ and Mg^{2+})	2
Average B factors	66.24
DNA atoms	61.87
QQPQ atoms	78.21
Other atoms (K^+ and Mg^{2+})	69.63

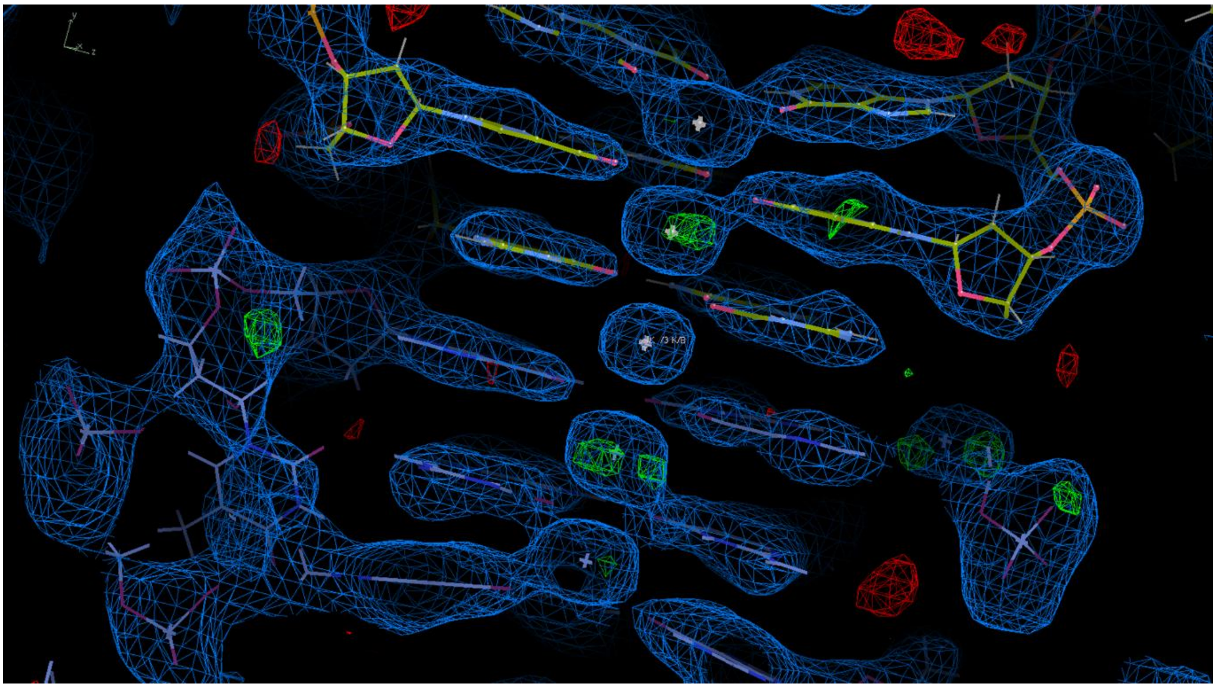

Figure S114. Electron density map (standard 2mFo-DFc contoured at 1 sigma; blue) of the G-quadruplex core structure.

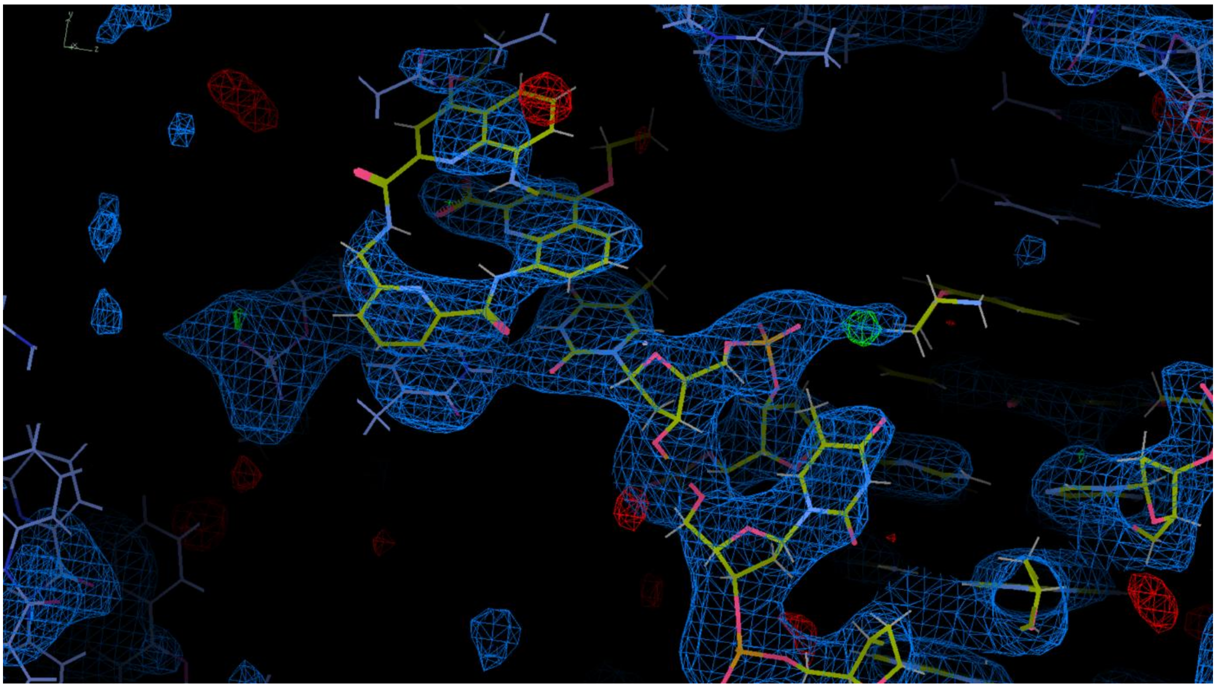

Figure S115. Electron density map (standard 2mFo-DFc contoured at 1 sigma; blue) of the QQPQ foldamer stacked on top of a thymine in the 222T loop region.

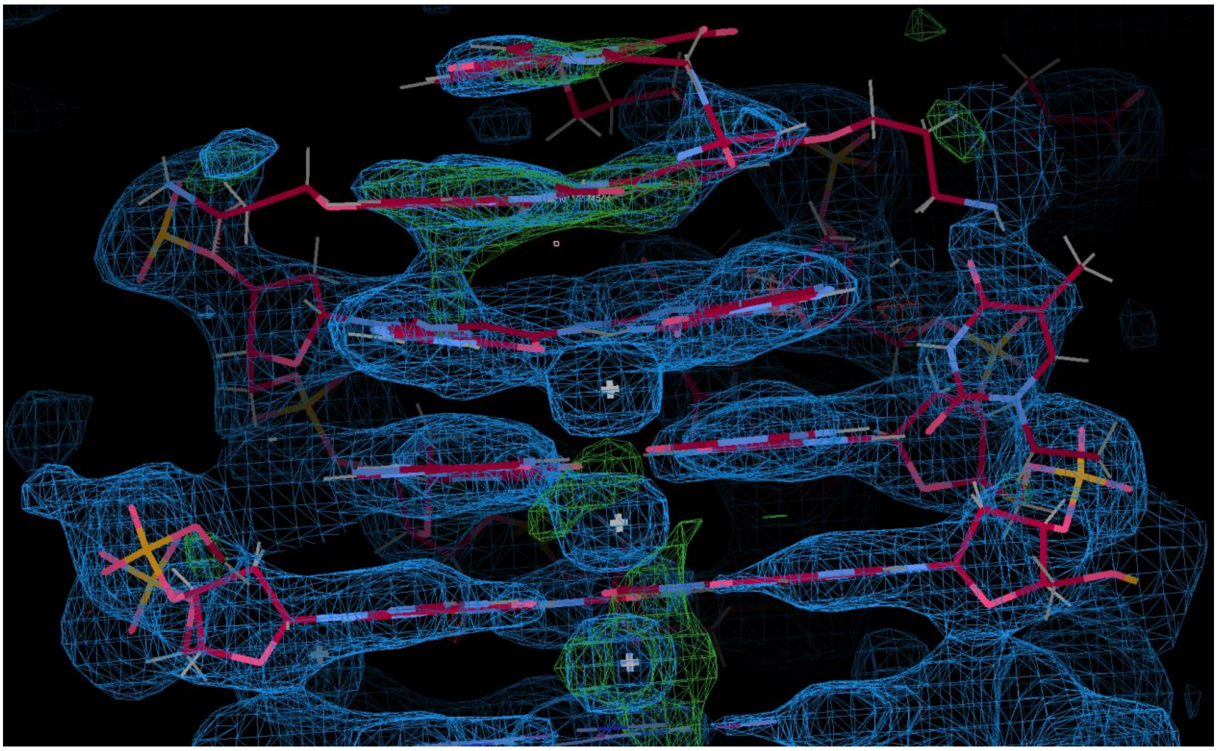

Figure S116. G4/QQPQ complex structure with two electron density maps superimposed: standard 2mFo-DFc contoured at 1 sigma (blue) and Polder omit map as calculated in the routine phenix_polder contoured at 2.7 sigma (green), i.e. an omit map excluding the bulk solvent around the omitted region, here the QQPQ molecule

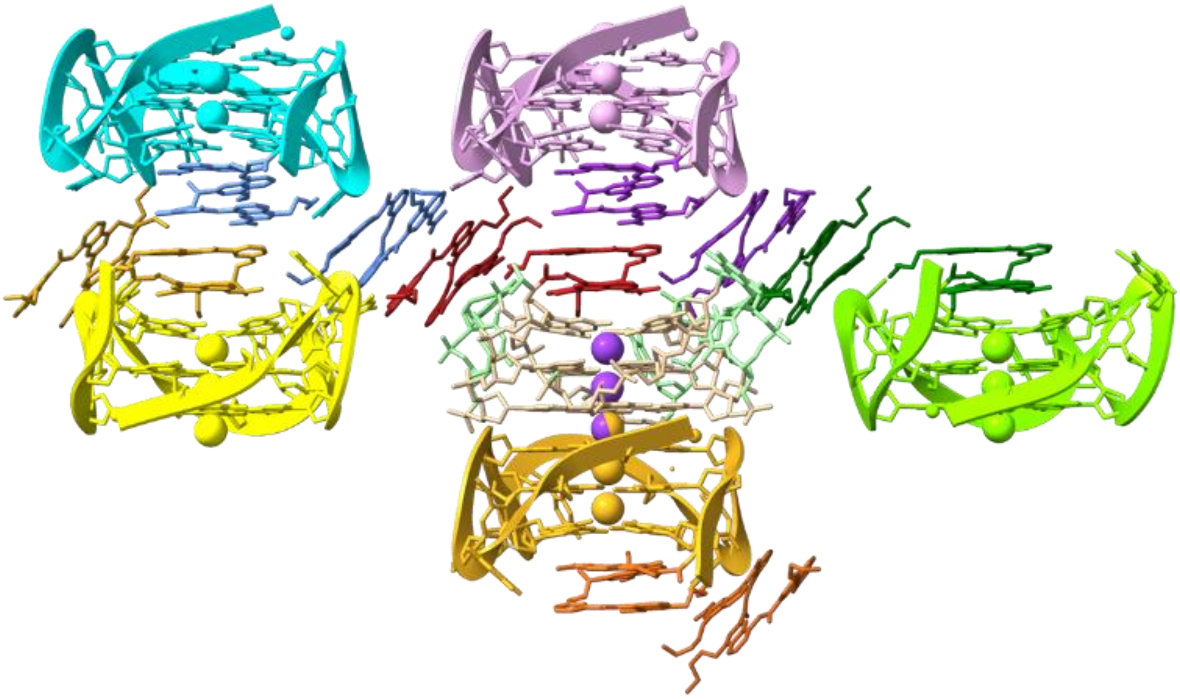

Figure S117. Crystal packing of the 222T/QQPQ crystal. Different colors show different unit cells.

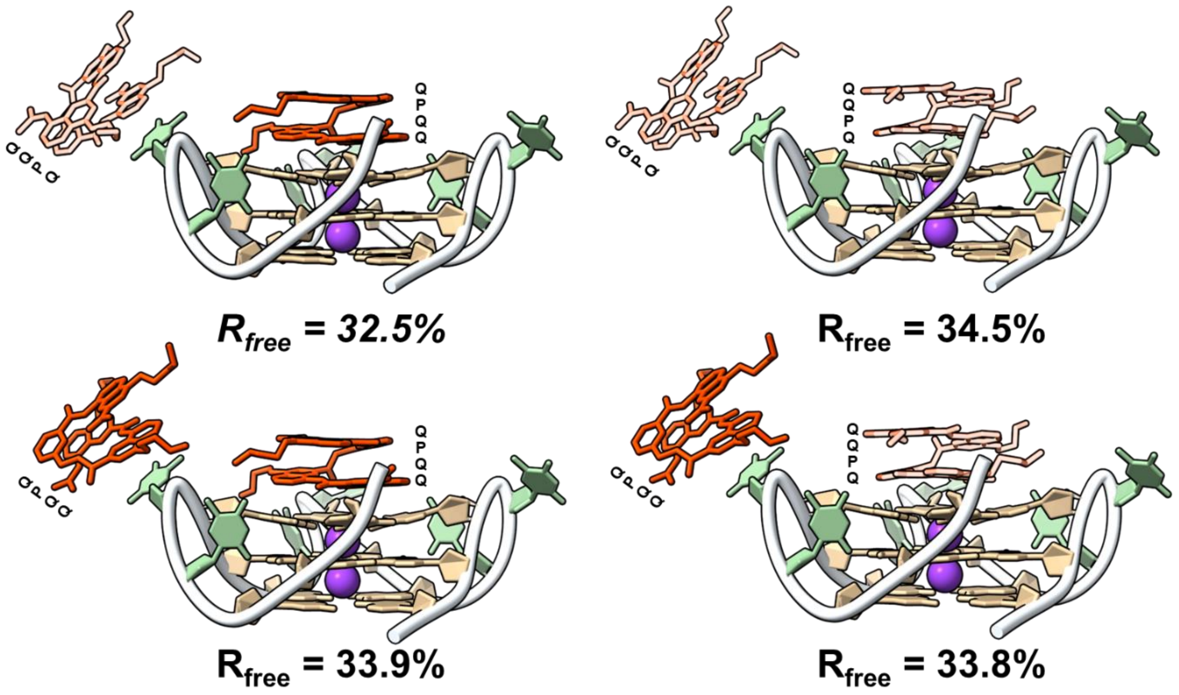

Figure S118. Flipping the foldamer between C- and N-terminal binding to test whether any conformation is statistically preferential. Schemes represent the four different models and their resulting R_{free} values.

1D and 2D NMR spectra

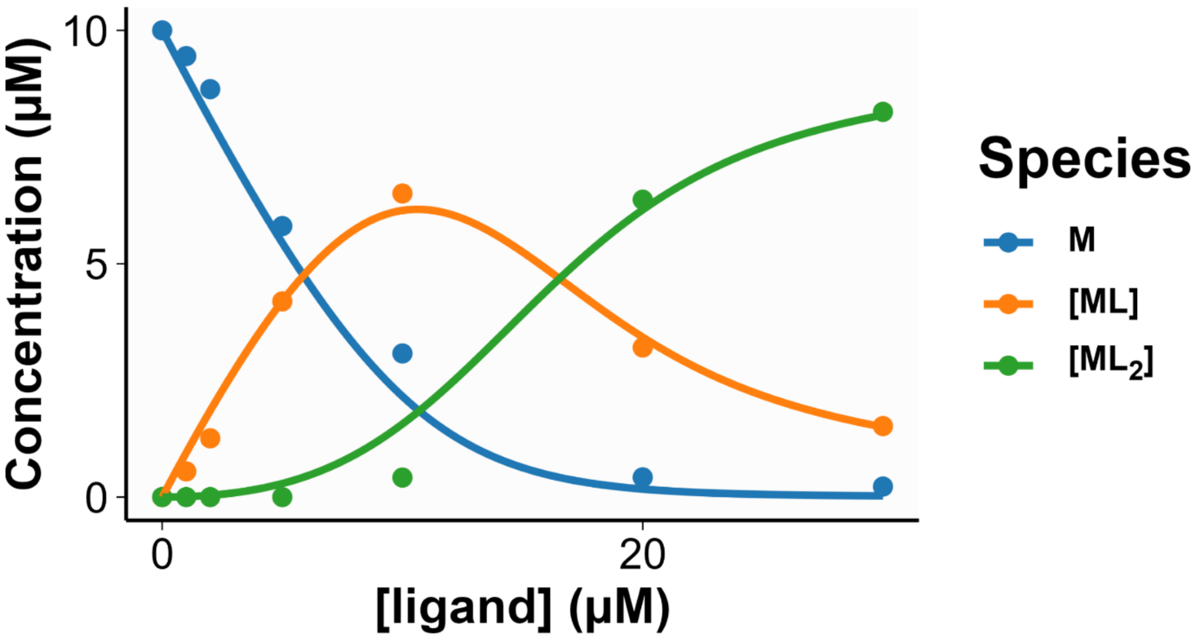

*Figure S119. Dynamic fitting of MS titration data for 2LK7 (dTT**G**$_3$T**G**$_3$T**G**$_3$T**G**$_3$T) and foldamer QQPQ with K_{D1} = 0.18 ± 0.08 µM and K_{D2} = 2.1 ± 0.7 µM. Samples contain 10 µM DNA, 0/1/2/5/10/20/30 µM QQPQ, 0.5 mM KCl and 100 mM TMAA (pH 6.8).*

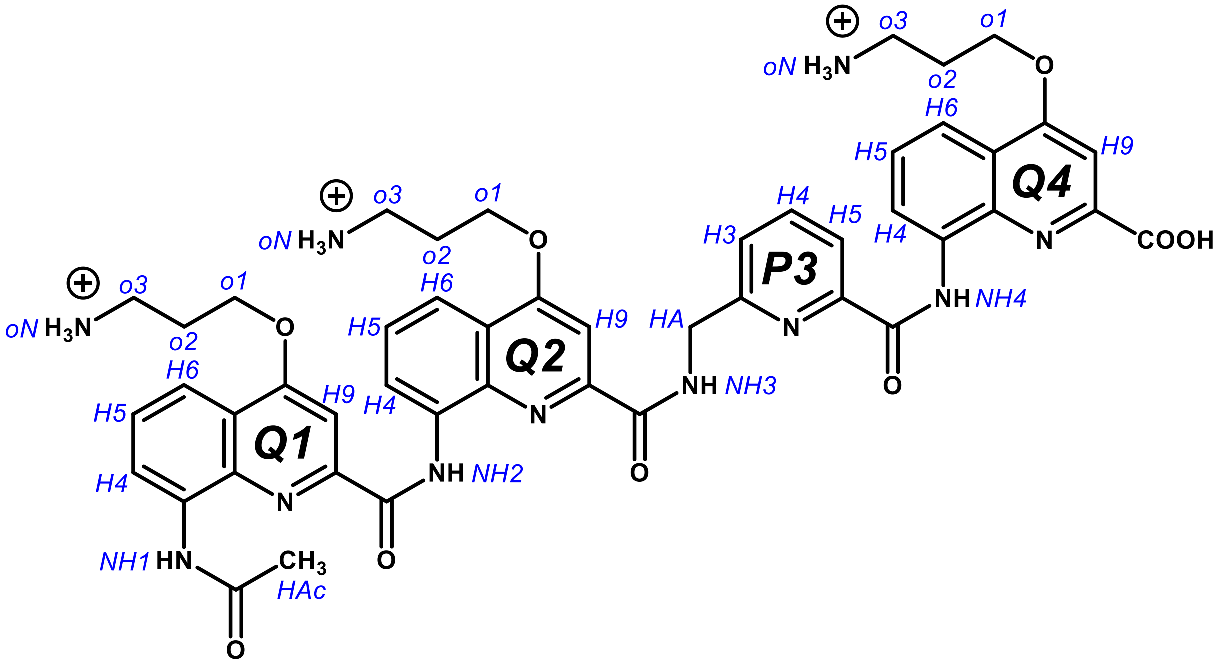

Figure S120. Proton labels in the QQPQ molecule.

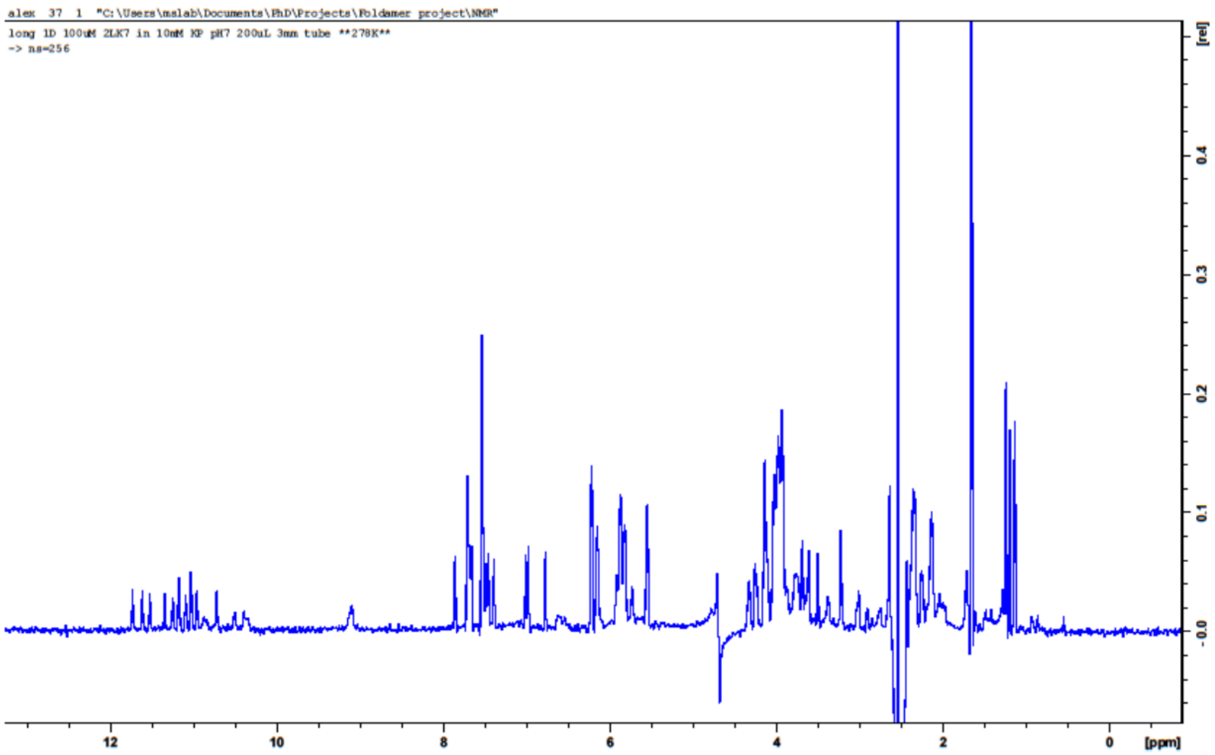

*Figure S121. Full 1D NMR spectrum of 2LK7 (dTT**G_3**T**G_3**T**G_3**T**G_3**T) on a Bruker Avance 700 MHz at 278 K. Sample matrix: 100 µM DNA, 10 mM potassium phosphate buffer (pH 7), 90/10 H_2O/D_2O. Water signal was suppressed by excitation sculpting.*

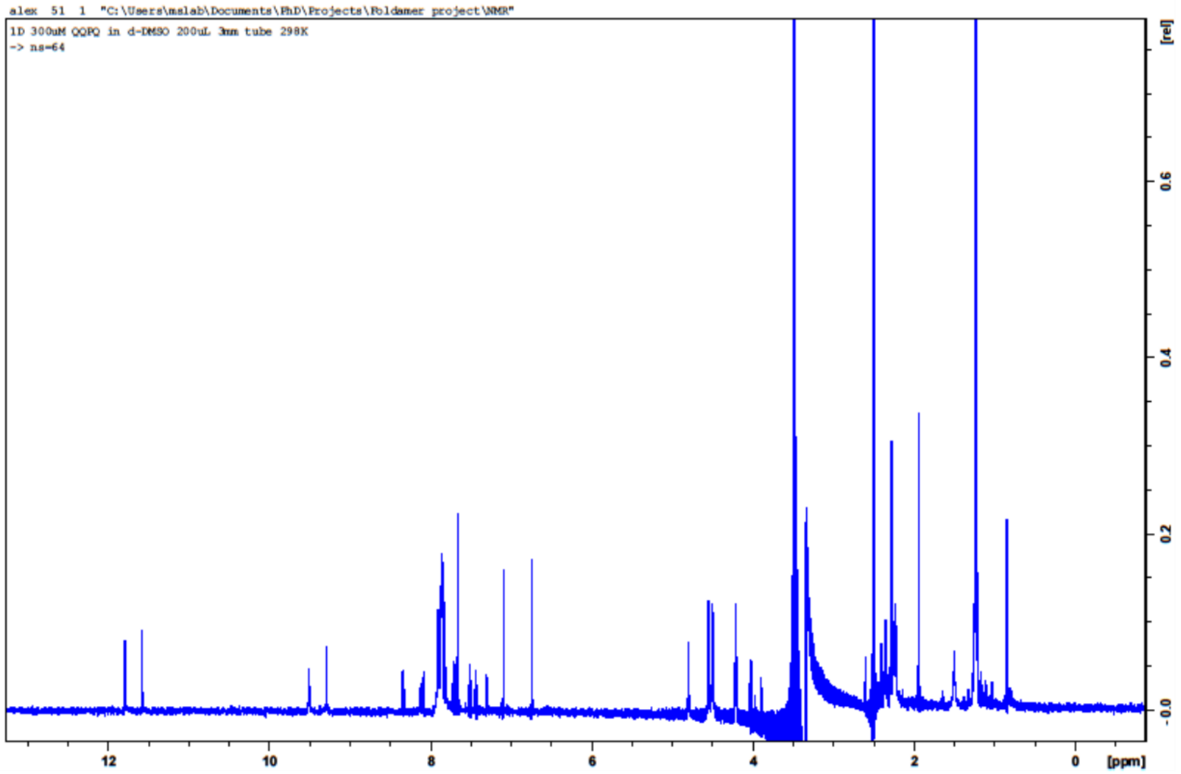

Figure S122. Full 1D NMR spectrum of Foldamer QQPQ on a Bruker Avance 700 MHz at 298 K. Sample matrix: 300 µM ligand in d6-DMSO.

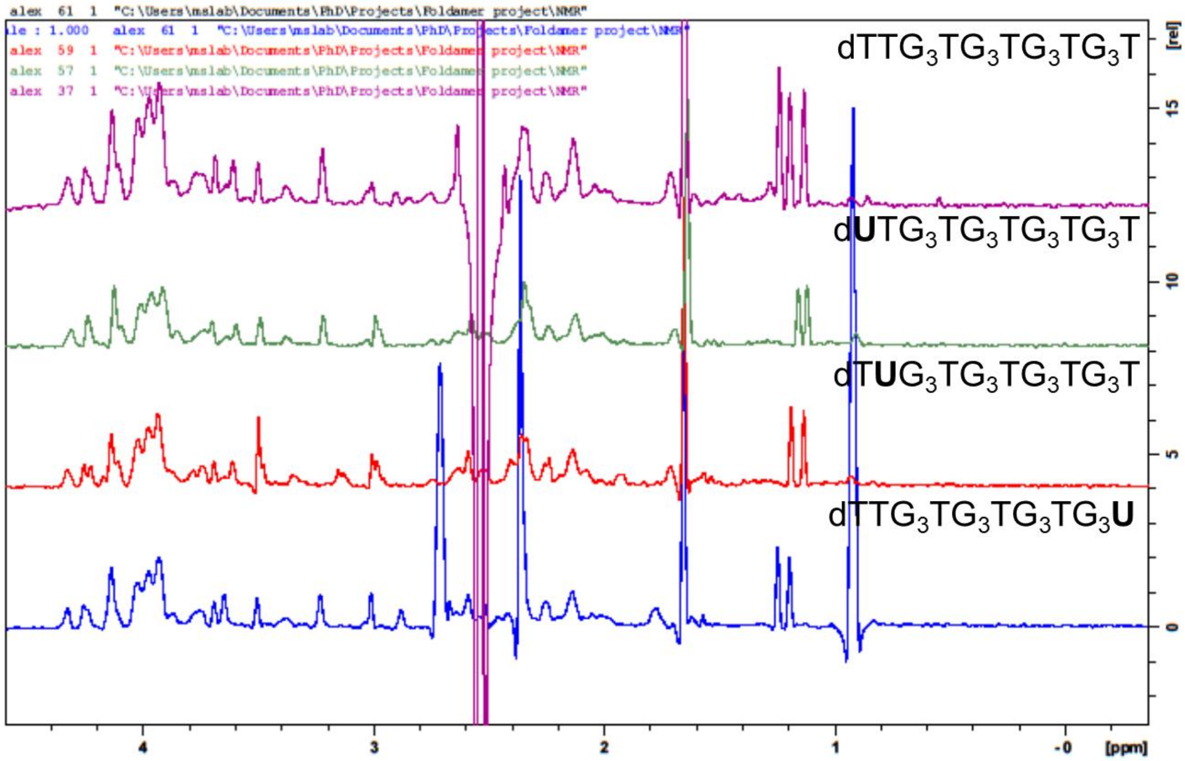

Figure S123. 1D NMR spectra of 2LK7 and labeled derivatives on a Bruker Avance 700 MHz at 278 K, showing the low shift region. Sample matrix: 100 µM DNA, 10 mM potassium phosphate buffer (pH 7), 90/10 H_2O/D_2O. Water signal was suppressed by excitation sculpting.

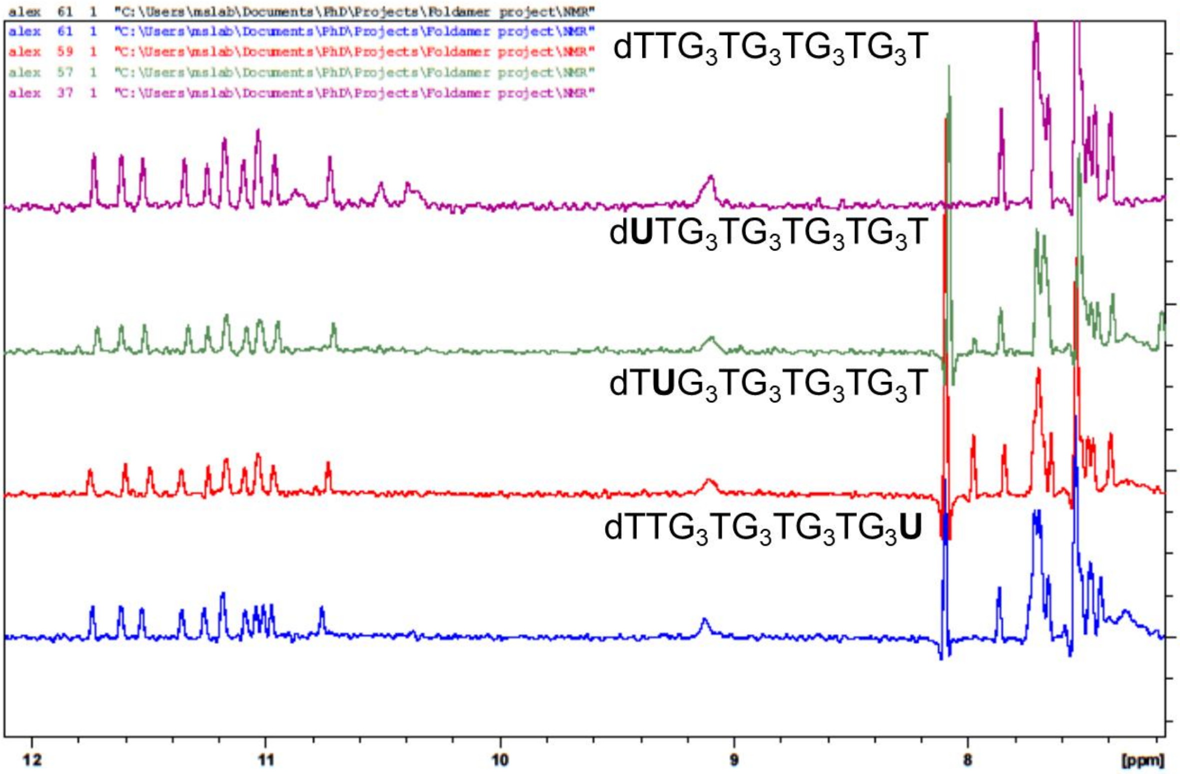

Figure S124. 1D NMR spectra of 2LK7 and labeled derivatives on a Bruker Avance 700 MHz at 278 K, showing the high shift region. Sample matrix: 100 µM DNA, 10 mM potassium phosphate buffer (pH 7), 90/10 H_2O/D_2O. Water signal was suppressed by excitation sculpting.

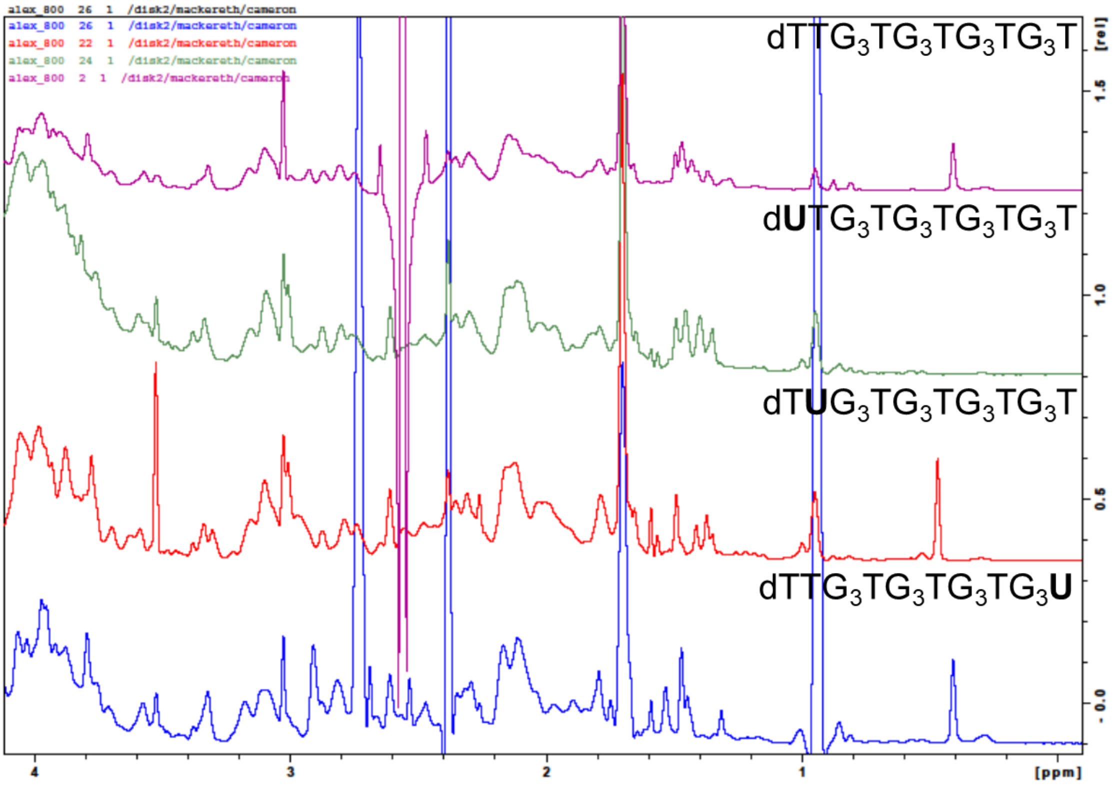

Figure S125. 1D NMR spectra of 2LK7 and labeled derivatives with QQPQ on a Bruker Avance 700 MHz at 278 K, showing the low shift region. Sample matrix: 100 μM DNA,300 μM ligand. 10 mM potassium phosphate buffer (pH 7), 90/10 H_2O/D_2O.

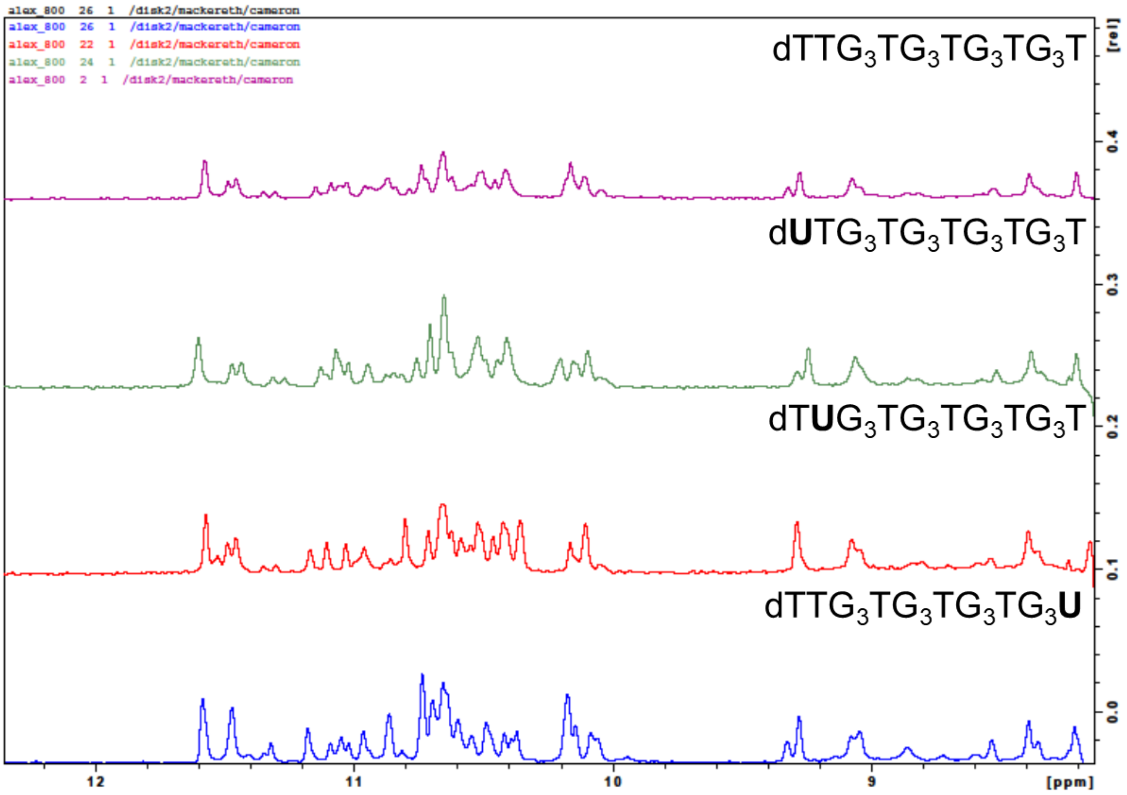

Figure S126. 1D NMR spectra of 2LK7 and labeled derivatives with QQPQ on a Bruker Avance 700 MHz at 278 K, showing the high shift region. Sample matrix: 100 μM DNA,300 μM ligand. 10 mM potassium phosphate buffer (pH 7), 90/10 H_2O/D_2O.

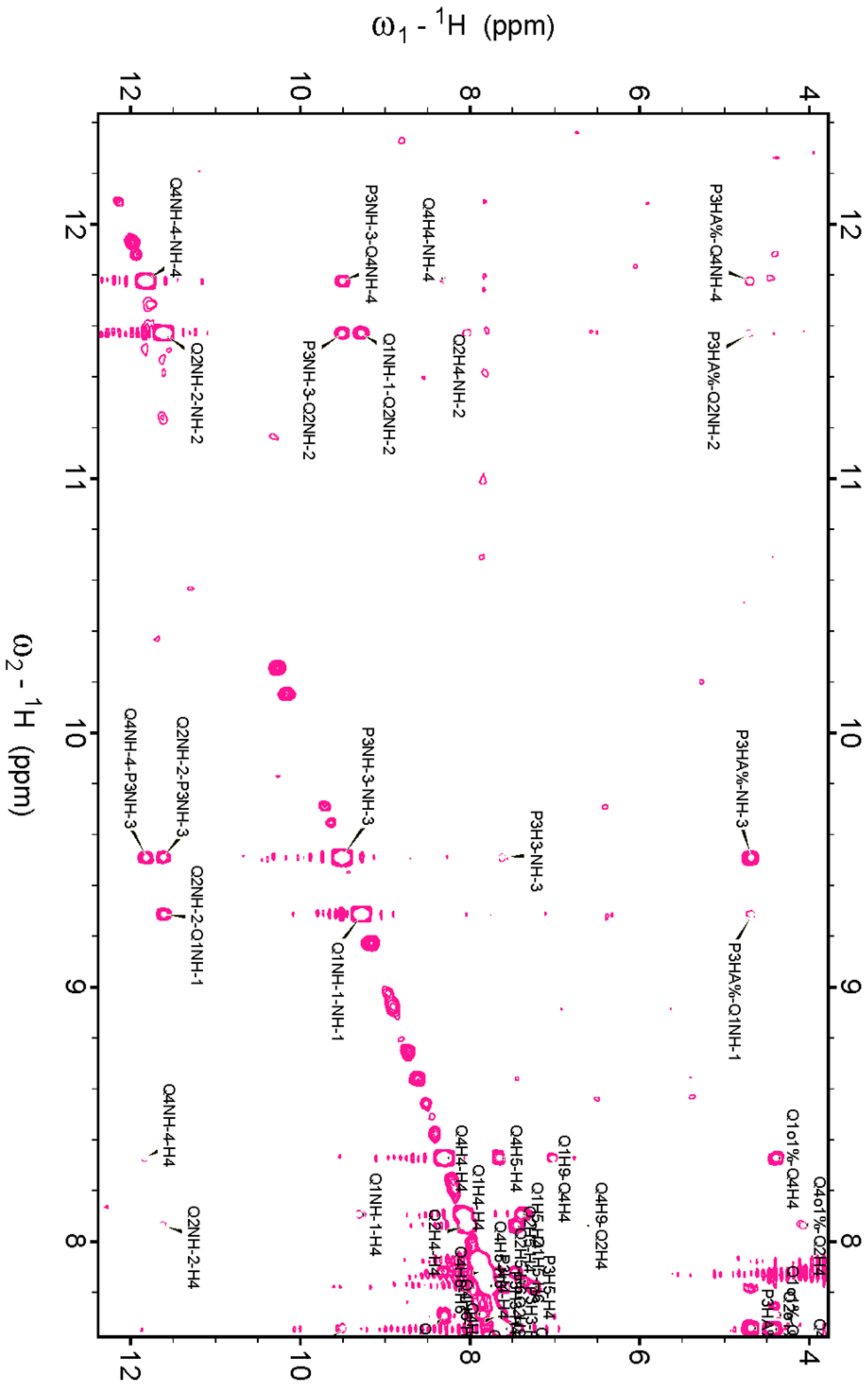

Figure S127. ¹H-¹H NOESY of QQPQ on a Bruker Avance-III 800 MHz; high shift region containing amide protons (NH). Sample matrix: 300 µM QQPQ in d6-DMSO.

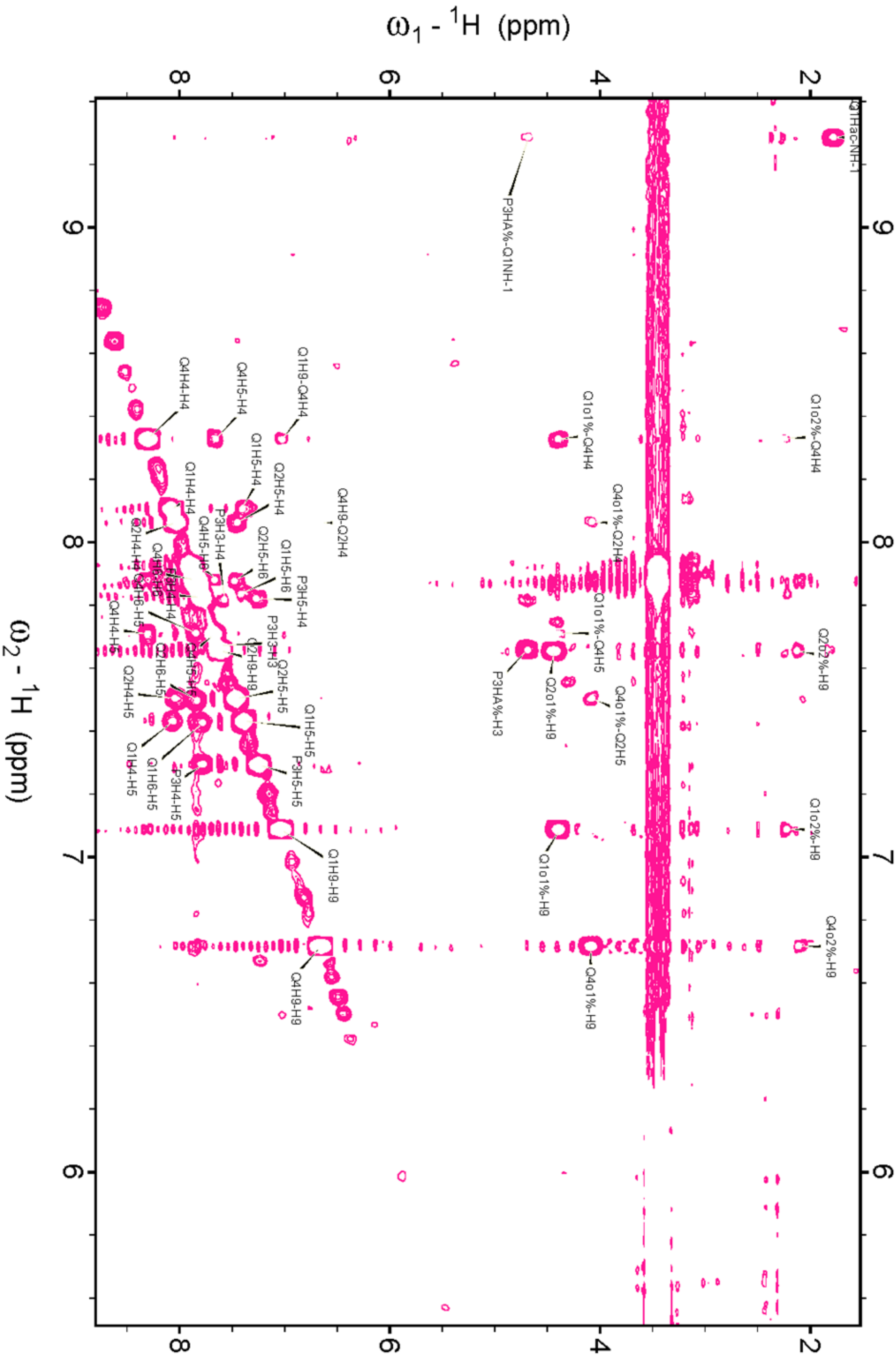

Figure S128. 1H-1H NOESY of QQPQ on a Bruker Avance-III 800 MHz; middle region containing the aromatic protons in the foldamer core (H4, H5, H6, H9). Sample matrix: 300 µM QQPQ in d6-DMSO.

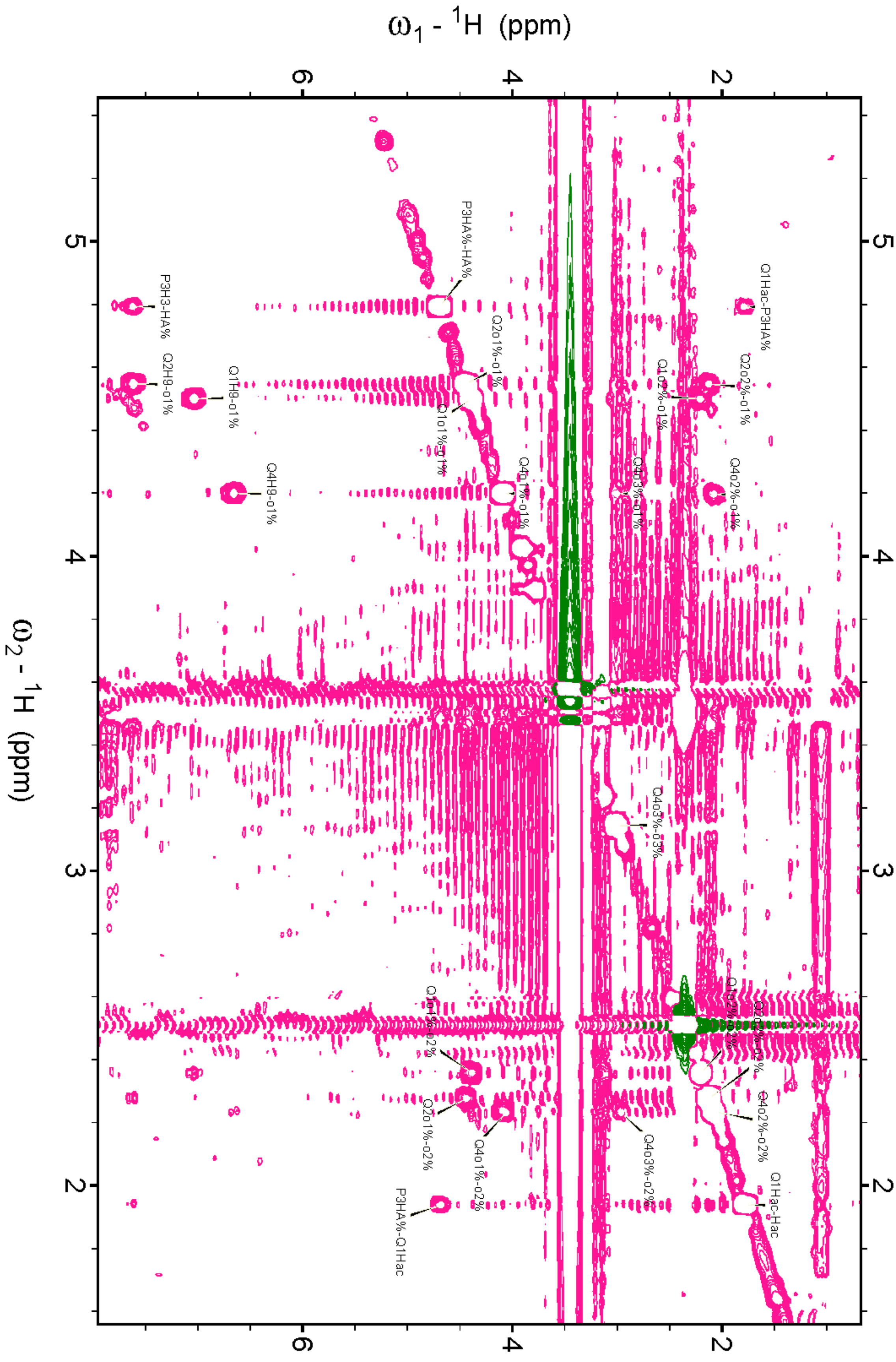

Figure S129. ^{1}H-^{1}H NOESY of QQPQ on a Bruker Avance-III 800 MHz; low shift region containing the aliphatic protons, which are mostly in the side chains(o1, o2, o3, HA, HAc). Sample matrix: 300 μM QQPQ in d6-DMSO.

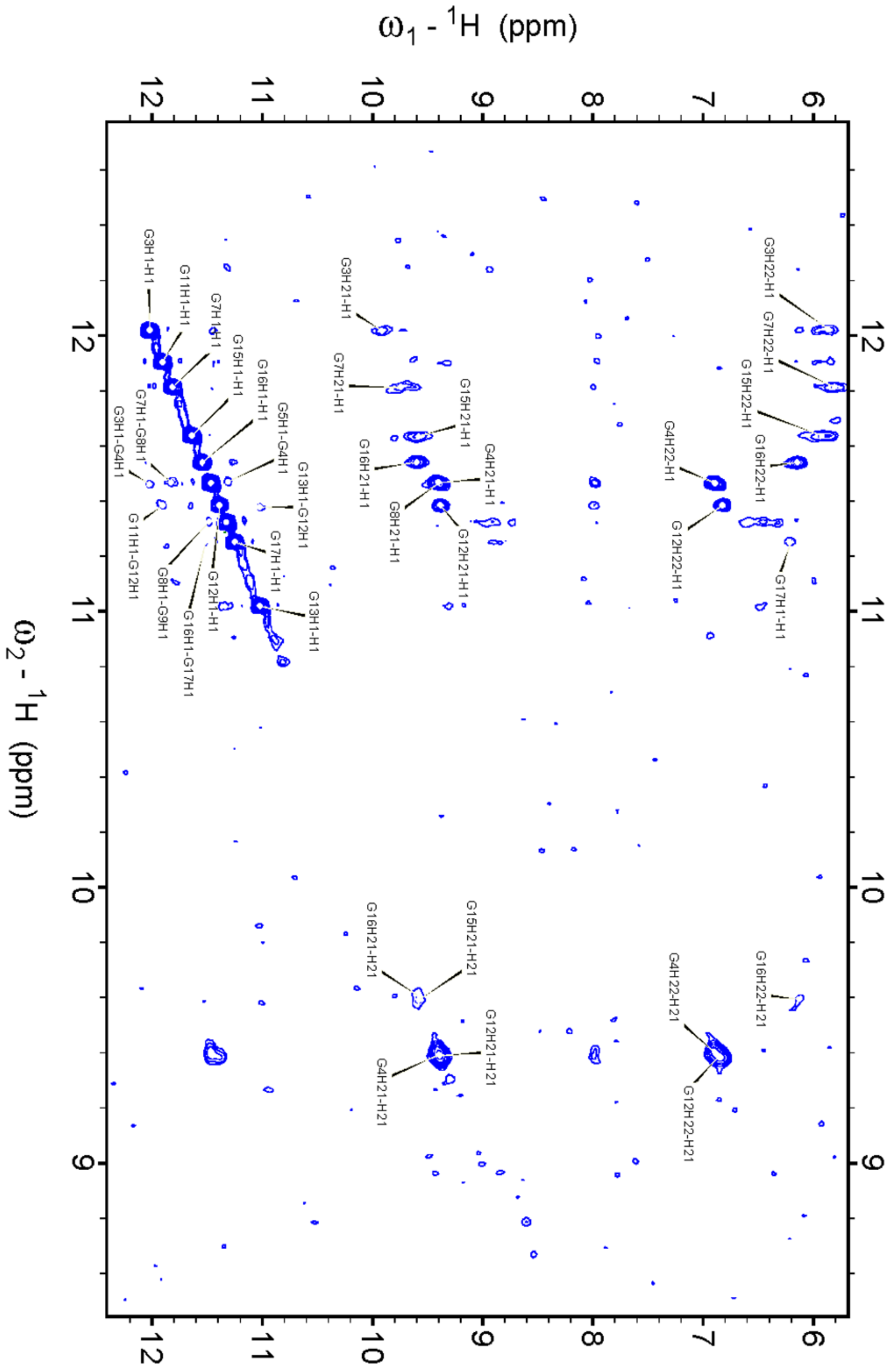

Figure S130. ^1H-^1H NOESY of 2LK7 on a Bruker Avance-III 800 MHz; high shift region showing the guanine H1/H2 protons. Sample matrix: 100 µM 2LK7, 10 mM potassium phosphate buffer (pH 7), 90/10 H_2O/D_2O.

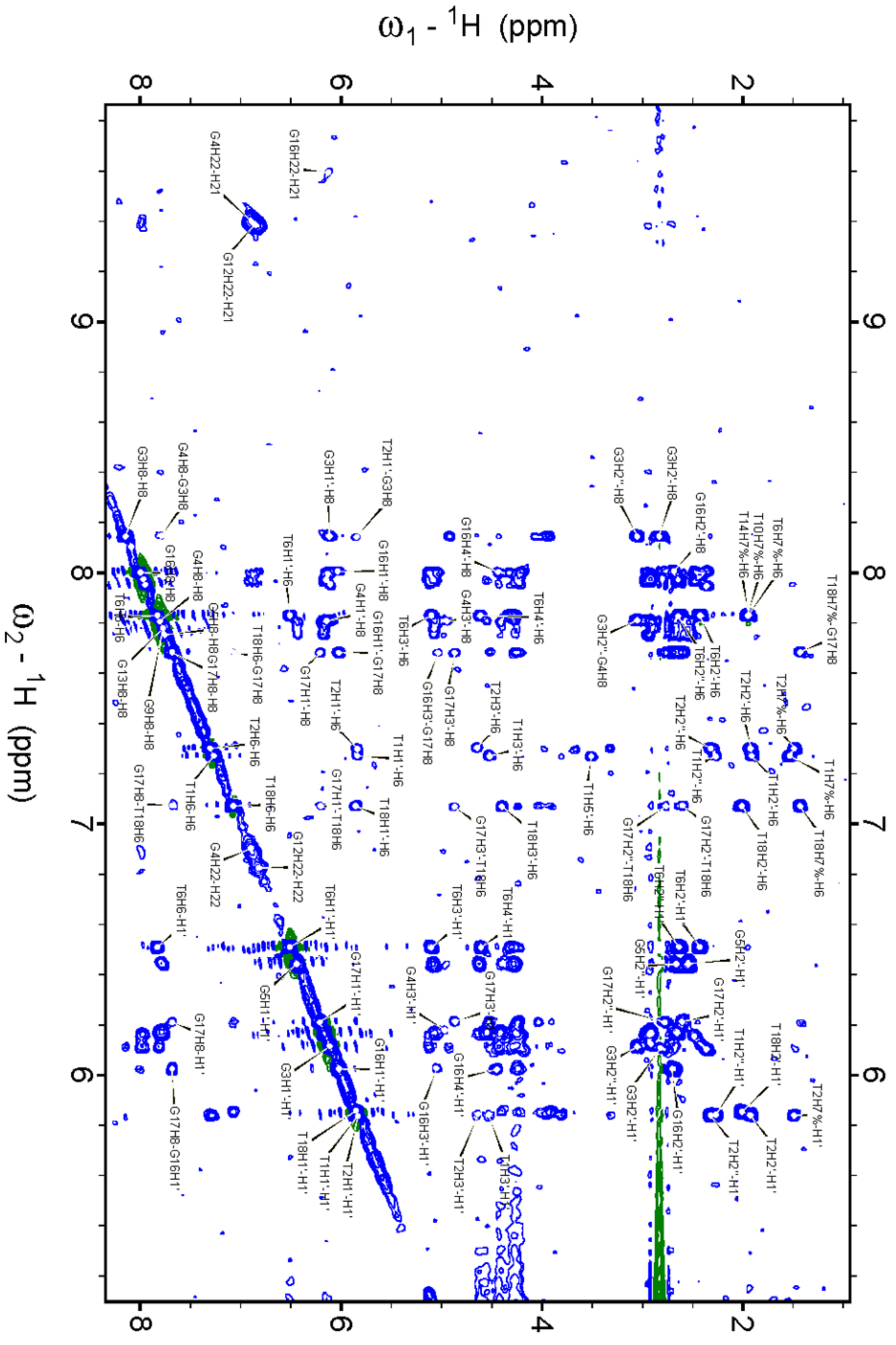

Figure S131. ^{1}H-^{1}H NOESY of 2LK7 on a Bruker Avance-III 800 MHz; middle region containing the guanine H8, thymine H6 and sugar H1' protons. Sample matrix: 100 μM 2LK7, 10 mM potassium phosphate buffer (pH 7), 90/10 H_2O/D_2O.

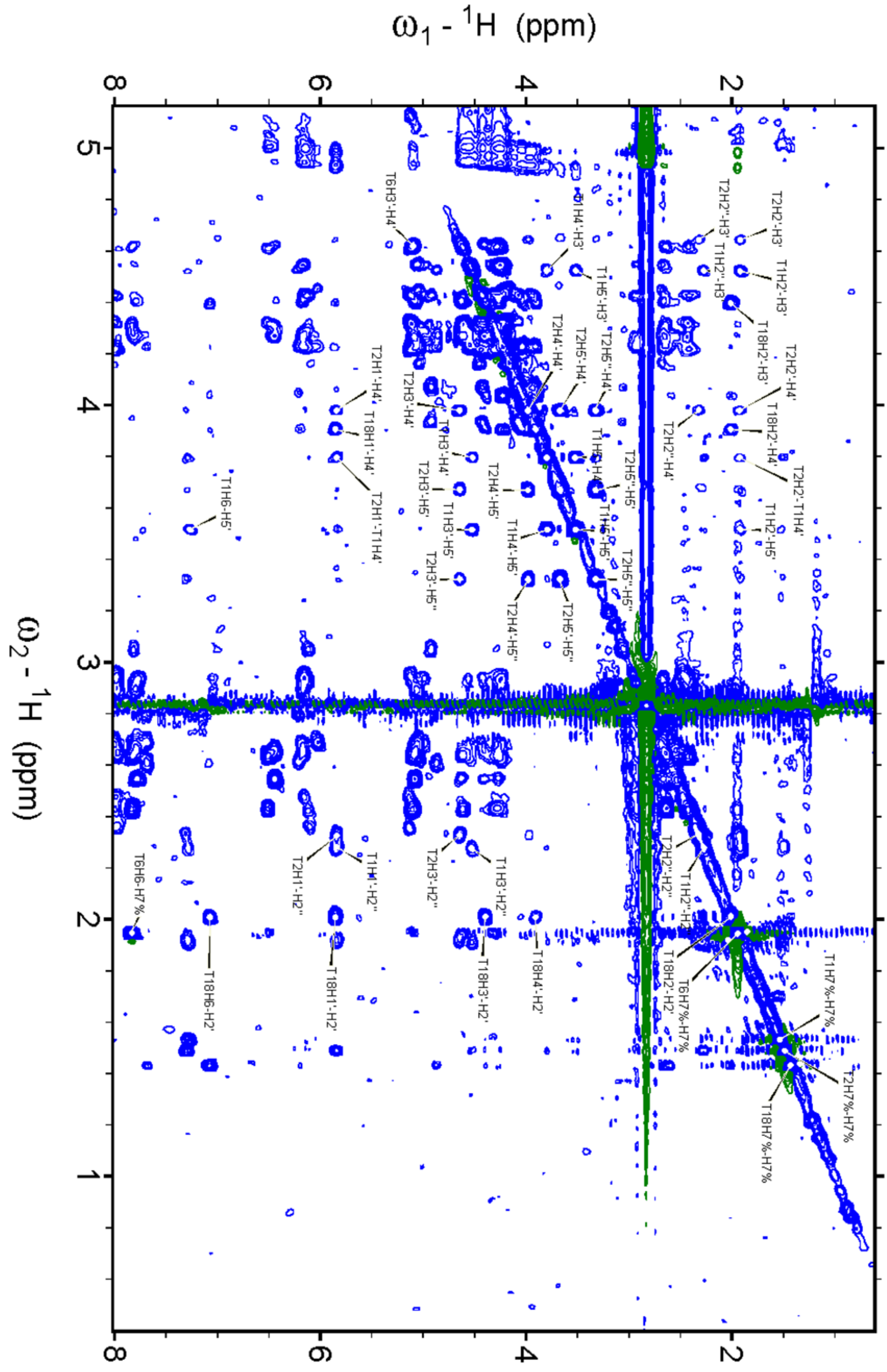

Figure S132. ^{1}H-^{1}H NOESY of 2LK7 on a Bruker Avance-III 800 MHz; low shift region containing sugar protons (H2' to H5') and the thymine methyl group (H7). Sample matrix: 100 µM 2LK7, 10 mM potassium phosphate buffer (pH 7), 90/10 H_2O/D_2O.

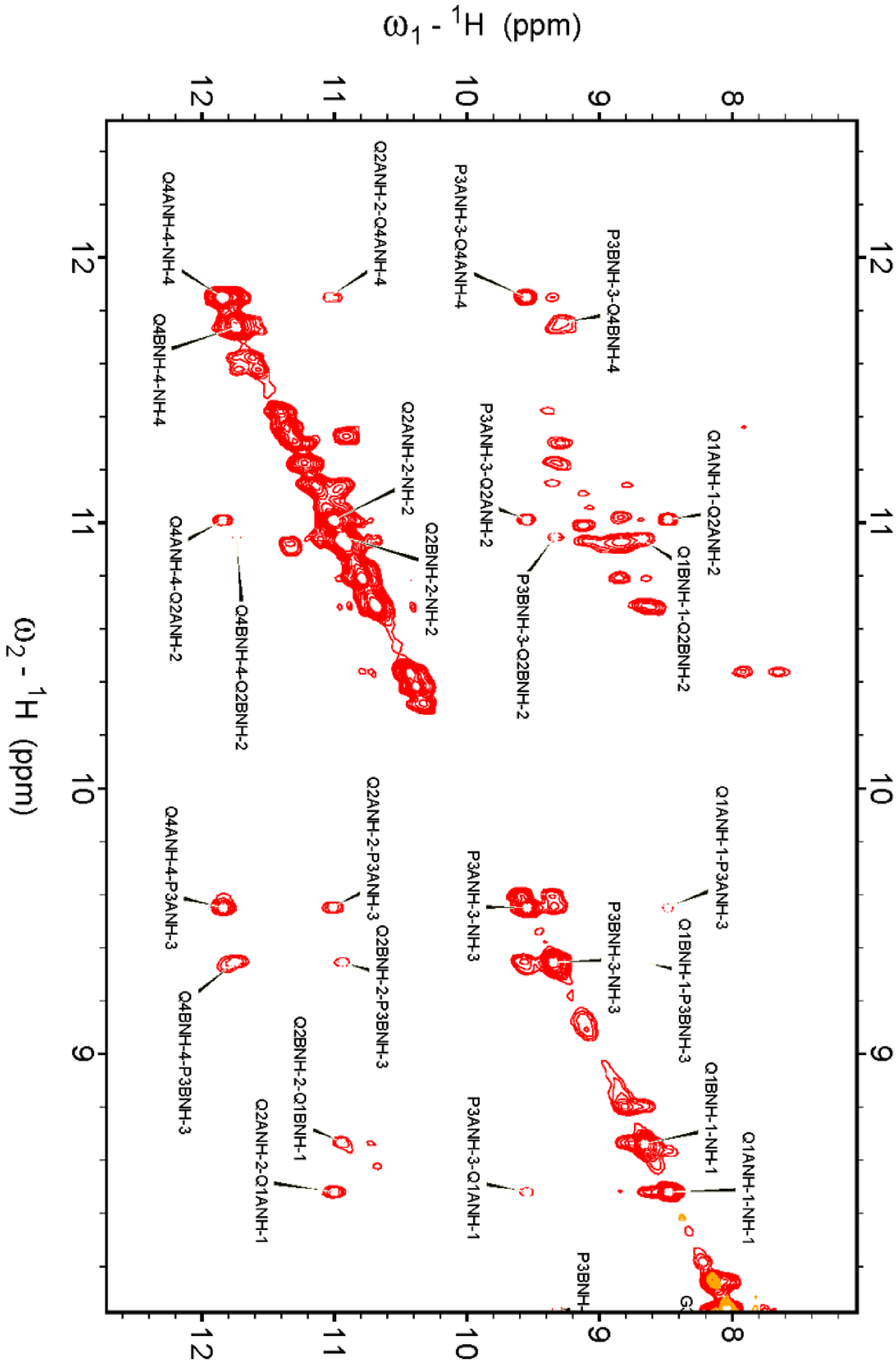

Figure S133. ^{1}H-^{1}H NOESY of 2LK7/QQPQ on a Bruker Avance-III 800 MHz; high shift region containing guanine H1 protons and foldamer NH protons. Sample matrix: 100 µM 2LK7, 300 µM QQPQ, 10 mM potassium phosphate buffer (pH 7), 90/10 H_2O/D_2O.

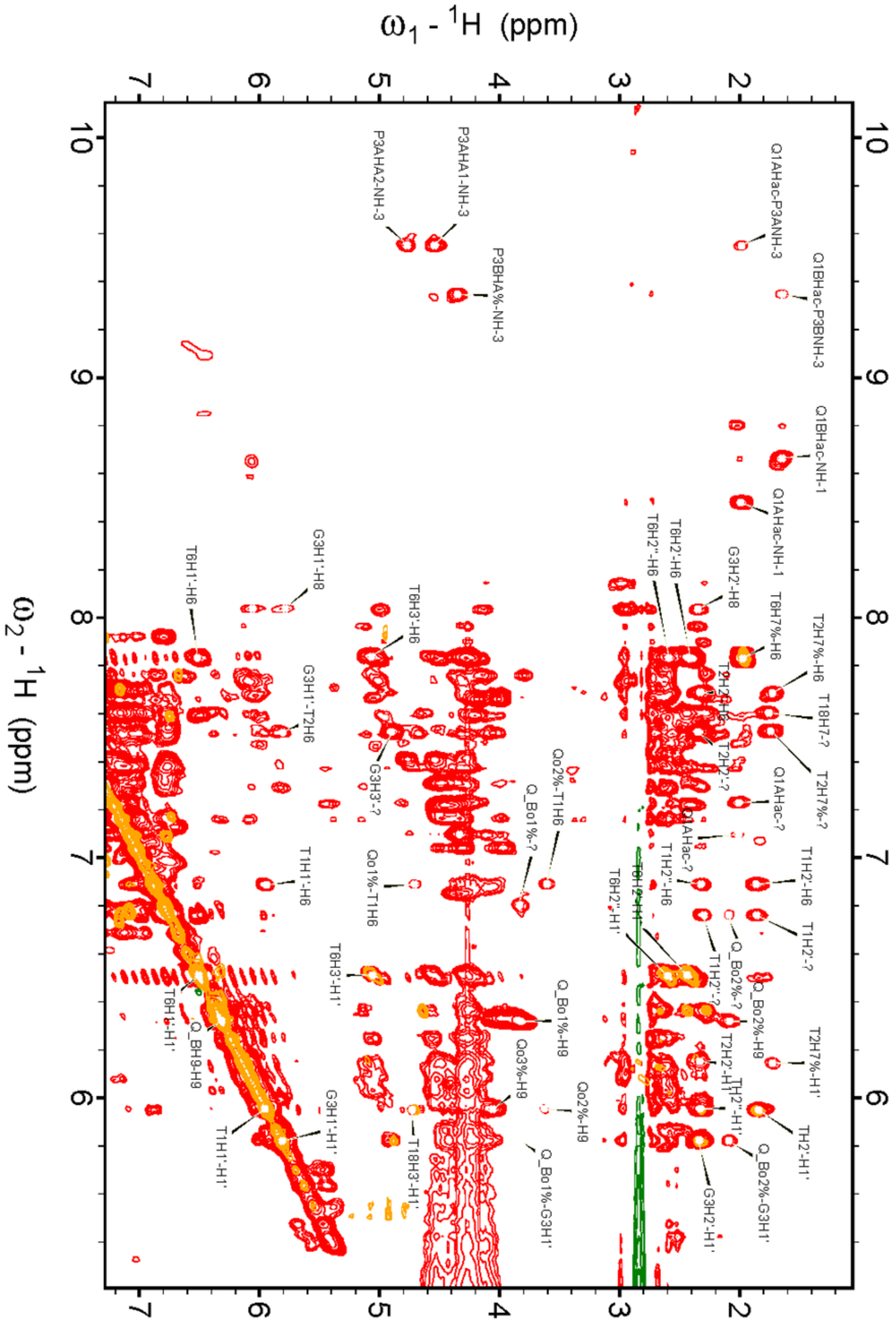

Figure S134. ^{1}H-^{1}H NOESY of 2LK7/QQPQ on a Bruker Avance-III 800 MHz; middle region containing aromatic protons of nucleobases and foldamer (as well as H1' sugar protons). Sample matrix: 100 µM 2LK7, 300 µM QQPQ, 10 mM potassium phosphate buffer (pH 7), 90/10 H_2O/D_2O. The orange overlay is a TOCSY spectrum of the same system.

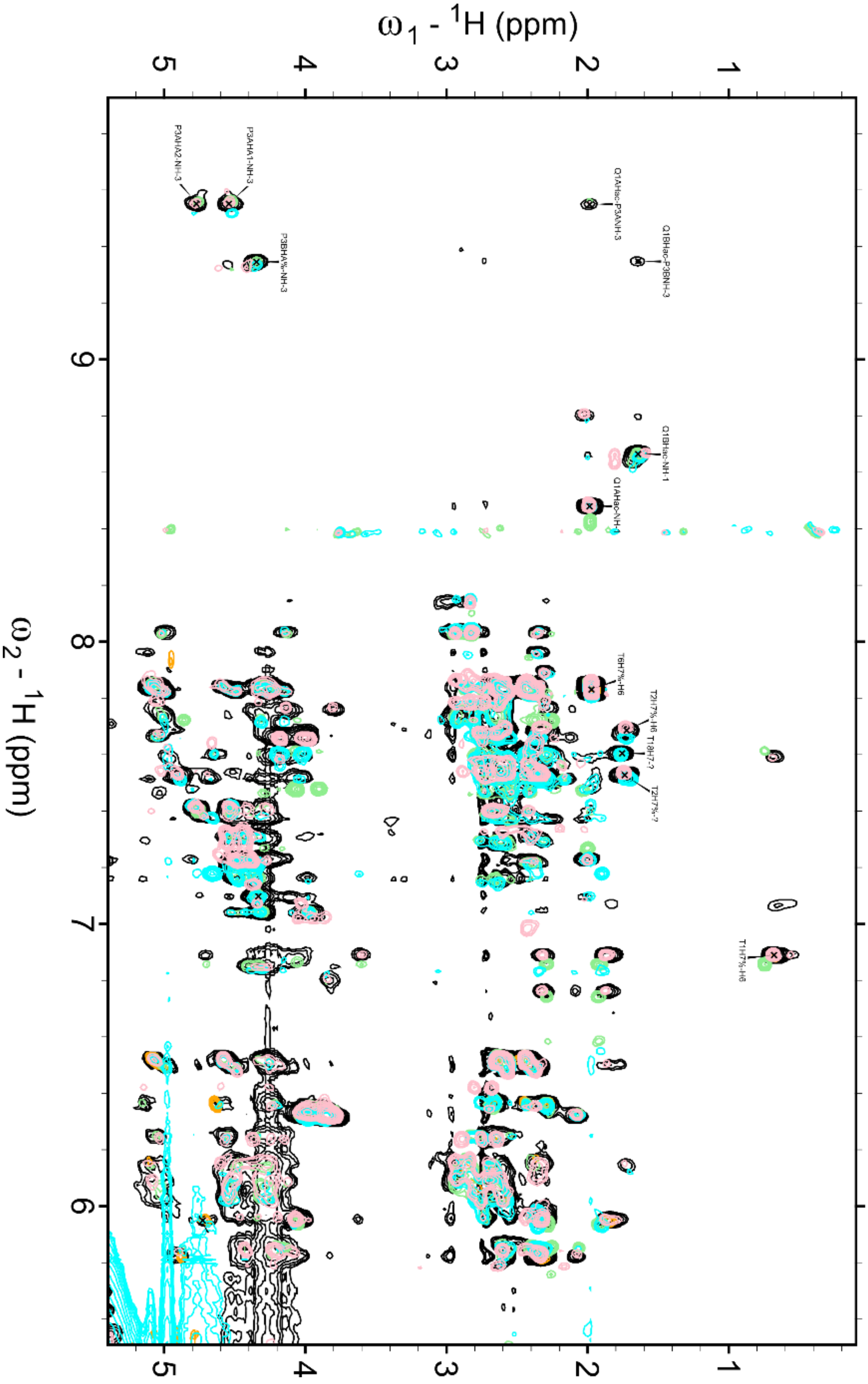

Figure S135. Full spectra for regions selected in Figure 7B. The color coding is the same, i.e.: cyan, green and pink for the 1U, 2U and 18U mutants, respectively, and the non-mutated 2LK7 sequence is shown in black.

CD kinetics of QQPQ-induced topology interconversion of 5YEY

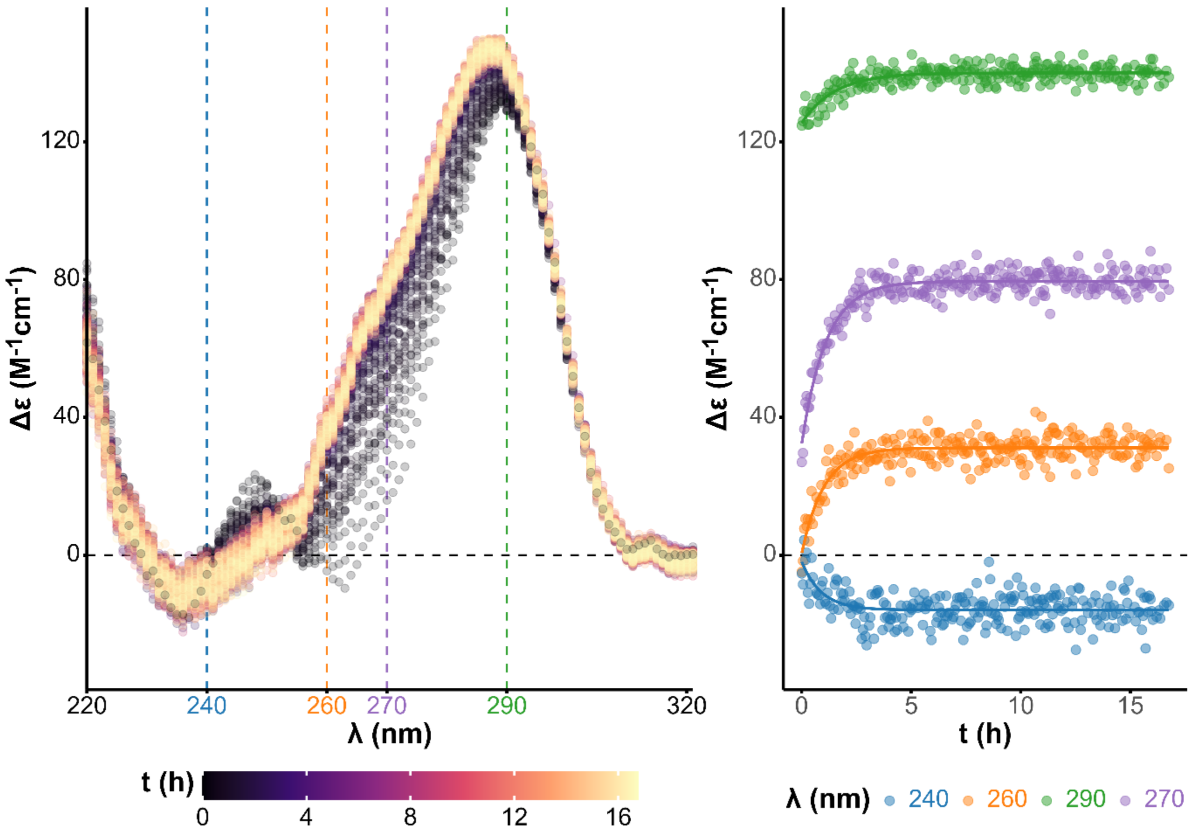

Figure S136. Kinetics of binding of QQPQ on 5YEY accompanied by structural rearrangement monitored by CD on a Jasco J-1500 instrument equipped with a Peltier temperature controller. Both partner were mixed at 10 µM in 100 mM TMAA, 1 mM KCl, at t=0 and 300 spectra (220—500 nm) were recorded with an interval of 180 s with the following parameters: 2 s integration time, 2.0 nm bandwidth, 200 nm/min scanning speed, 22°C, baseline correction. A control kinetics was recorded simultaneously in absence of QQPQ and the signal variations were subtracted from the QQPQ/5YEY kinetics. Data is shown here converted to molar ellipticity using Equation 1 (main text).

Quantum chemical and molecular dynamics calculations

Methods

Torsion scan

A simultaneous scan of three amide bond dihedral angles was performed with the ORCA 6.0 software package,[10–21] using the libxc and libint libraries.[22,23] The scan employed 10-degree increments across a ± 50° range around the initial torsion values, starting from the original angles. Geometry optimizations were carried out using the B3LYP density functional with the 6-31G(d) basis set and tight SCF convergence criteria.[24–27] Implicit water solvation was performed using the SMD solvation model.[28] The calculations were parallelized across sixteen cores with Open MPI 4.1.6.

Conformer search

To extend the search beyond discrete changes in dihedral angles of amide bonds only, we performed a determination of the ensemble around the global minimum using the Global Optimizer Algorithm (GOAT) implementation, in ORCA.[10–23] Briefly, a first local minimum is determined from the starting position on the potential energy surface. The energy is then increased ("uphill") until a barrier is crossed, and a new geometry optimization provides a new minimum. The process is repeated until no new minimum is found. Geometry optimizations were carried out with the ORCA implementation of the Semiempirical Extended Tight-Binding (xTB) Program Package,[29] using the semiempirical GFN2-xTB and GFN-FF (for uphill steps).[30,31] Implicit water solvation was performed using the analytical linearized Poisson–Boltzmann (ALPB) solvation model.[32] The search was set to stop when $\Delta S_{conf} < 0.1\ \mathrm{cal/(mol\ K)}$ to maximize the conformational entropy and be more exhaustive. The calculations were parallelized across sixteen cores with Open MPI 4.1.6.

Force field modifications for the foldamer

The single-point energy of the foldamer structure was calculated using quantum mechanical (QM) density functional theory (DFT) with the B3LYP functional and 6-31G(d) basis set, employing the ORCA 6.0 software package, then converted to the molden file format.[10,12–21] Two-stage Restrained Electrostatic Potential (RESP) atomic charge calculations were then performed in Multiwfn.[33,34] An initial antechamber file was generated with Amber atom types, -2 formal charge (accounting for two phosphates),[35,36] wherein AM1-BCC partial charges were then replaced with those of the RESP calculation. A prepin file was generated using prepgen to define the topology and molecular parameters of QQPQ, and the force field parameters were subsequently derived using parmchk2.[37]

Complex preparation

The complexes were prepared in PyMOL (3.0.0, Schrödinger, NY, USA) from the crystal structure. The latter was first cleaned up: a single 222T unit was kept, extra magnesium and potassium ions were removed (only the 2 tetrad-bound K^+ were conserved), as well as the second QQPQ bound on the loop at the interface with other units. Missing 3' and 5' thymine atoms were added at the system preparation step (*vide infra*).

Variant complexes were then generated by adding (on the 5'-tetrad) and/or flipping (binding through its C- instead of N-terminus) and/or mirroring (to its right-handed enantiomer) QQPQ, where necessary. A total of 8 complexes were prepared and named following the nomenclature:

- 3 or 5, to designate the 3'-tetrad or 5'-tetrad binding interface

- N or C, for N-terminus or C-terminus QQPQ binding interface
- L or R, for left-handed or right-handed enantiomer

Hence, the "original" crystal structure is 3NM, while e.g. 3NM/5CM refers to a 2:1 complex, with the L enantiomer bound through it N-terminus on the 3'-tetrad, and C-terminus on the 5' end.

For the Hybrid-1 structural variant of 5YEY, the structure of 2JSM was used as a base. The extra 5' residues were removed and the 5'O capped with an hydrogen. The residues were renumbered and A18 was mutated to a T. Potassium cations were positioned at their expected coordination sites, in between tetrads. QQPQ was stacked on top of the 5' tetrad with a random orientation.

MD preparation

The systems were then prepared for molecular dynamics using the Leap program from the Amber24 suite [37]. The OL21 force field was used for DNA.[38,39]

The structure was explicitly solvated in a truncated octahedral box of water molecules, using the OPC model with the ad hoc Li/Merz ion parameters of atomic ions (12-6 set),[40,41] with a minimum of 14 Å between the solute and the box edge. K^+ and Cl^- ions were added to neutralize the system and adjust the ionic strength to 100 mM. The number of ions was determined SLTCAP method, using Equation S1 simplified by Machado and Pantano into Equation S2, where ν_w is the water volume of the simulation box in reduced units, c_0 the salt concentration, Q the total charge of the complex, and $N_0 = \frac{N_w \times c_0}{55.5}$ with N_w the number of water molecules in the simulation box.[42]

$$N_\pm = \nu_w c_0 e^{\mp ArcSinh\left(\frac{Q}{2\nu_w c_0}\right)} \qquad \text{(Eq. S1)}$$

$$N_\pm = N_0\sqrt{1+\left(\frac{Q}{2N_0}\right)^2 \mp \frac{Q}{2}} \qquad \text{(Eq. S2)}$$

Note that the simpler SPLIT method described by Machado and Pantano cannot be applied as our system does not satisfy the $N_0 \gg Q$ condition; however it yields identical values.

Molecular dynamics

All simulation steps were performed with pmemd.cuda (v. 18.0) from the CUDA version of AMBER,[43–45] on an NVIDIA H100 PCIe Tensor core GPU (CUDA version: 12.4) from the DOREMI CALI v3 cluster of the *Mésocentre de Calcul Intensif Aquitain* (Université de Bordeaux, France). The system was minimized for 20000 cycles using the steepest descent algorithm for the first 4000 steps and the conjugate gradient for the next 16000 steps. The system was then heated at constant volume from 0 to 298 K over 18 ps then kept for 2 ps at the final temperature, using a time step of 2 fs, the Langevin thermostat with a 2.0 ps^{-1} collision frequency and a different seed for the pseudo-random number generation for every run to avoid synchronization artifacts,[46] an 8 Å non-bonded cutoff, and the bonds involving hydrogen were constrained with the SHAKE algorithm. The system was further equilibrated five times at 298 K with the parameters above and the pressure kept at 1.0 bar with the Berendsen barostat,[47] before production MD simulations were run for a microsecond.

Data analysis

Trajectory files cleanup, alignment, filtering and analysis was performed with R 4.4.2, using the bio3d package and custom scripts.[48] The determination of RMSD was performed with the rmsd function. All atom-atom were measured with the dist.xyz function, complemented with the com function to determine center of masses for ring-ring distances. Molecular structure images were created in PyMOL 3.0.

Interplanar angle calculations

All interplanar angles were computed by first defining a geometric plane for each aromatic ring and then measuring the angle between their normal whose length has been normalized to 1.

Three atoms were used to defined the planes: C7, C4a and C2 for quinoline, C2, C4 and C6 for pyridine, and C2, C5 and C8 for guanine. For each set of these three non-collinear points p_1, p_2, p_3 two edge vectors v_1 and v_2 were defined:

$$v_1 = p_2 - p_1$$
$$v_2 = p_3 - p_1$$

Each normal vector n^* was calculated from the cross product of edge vectors, then normalized to length 1 by dividing with the corresponding Euclidian length $\|n^*\|$.

$$n^* = v_1 \times v_2$$
$$n = \frac{n^*}{\|n^*\|} = \frac{n^*}{\sqrt{n^* \cdot n^*}}$$

The angle between the two planes θ characterized by length-normalized normal vectors n_1 and n_2 was then calculated with :

$$\theta = arccos(n_1 \cdot n_2)$$

Results were subsequently converted to degrees by multiplying with $180/\pi$.

These calculations were performed frame-by-frame over trajectories for each aromatics (Q1, Q2, P3, Q4) and 3' and/or 5' tetrad guanines (depending on where the foldamer is stacked) with a custom R script, parallelized with the *future.apply* package.[49]

PCA and cluster centroid determination

For each trajectory, principal component analysis (PCA) was performed using the pca.pdbs function from the bio3d R package.[48] The optimal number of clusters was determined via k-means clustering, based on Euclidean distances computed over the first four principal components. Subsequent components were excluded due to their low contribution to the total variance. Cluster number was constrained to a range of 3 to 5 to maintain interpretability. The NbClust function from the NbClust R package was used for this selection.[50] For each cluster, a representative conformation was defined as the structure closest (in Euclidean distance) to the cluster centroid, and its coordinates were exported to PDB format using the write.pdb function from bio3d.

Results

Torsion scan

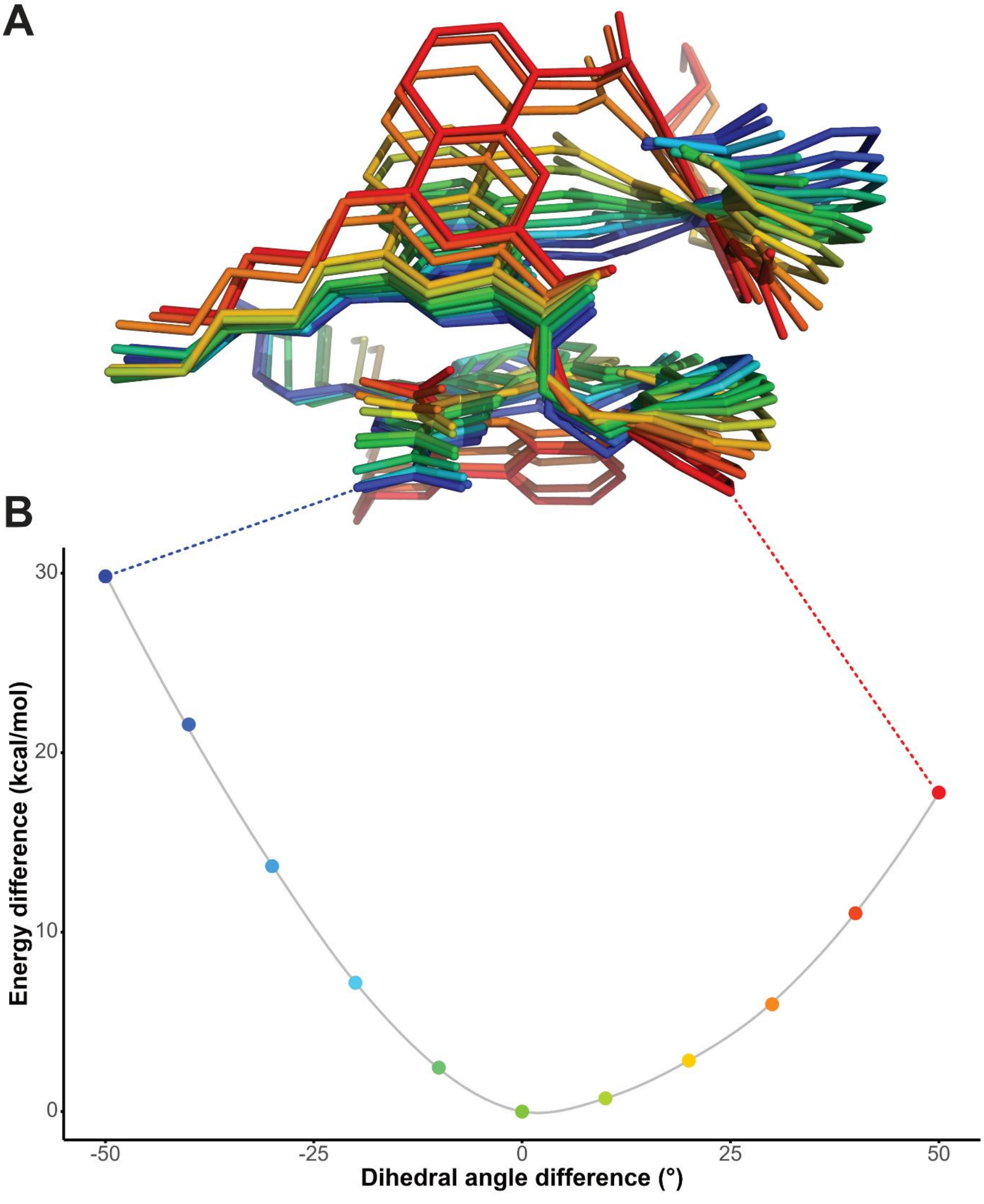

Figure S137. Torsion scan of QQPQ: simultaneous scan of the dihedral angles of the amide bonds in a ± 50° range around the original values, with a 10 degree step. The geometry optimization were performed with the B3LYP density functional, 6-31G(d) basis set and tight SCF convergence criteria, with implicit water solvation using the SMD model. Panel A shows the superimposed optimized coordinates whose energy relative to the initial conformation are shown in panel B.

Table S6. Final ensemble information. 133 conformers (#) were found with less than 6 kcal/mol difference (lowest conformer energy: -197.172519 Eh), among which 16 have less than 3 kcal/mol difference (green). Degeneracy indicates the number of rotamers.

#	Energy (kcal/mol)	Degeneracy	%total	#	Energy (kcal/mol)	Degeneracy	%total	#	Energy (kcal/mol)	Degeneracy	%total
**0**	0	8	49.34	**45**	4.609	2	0.01	**89**	5.511	2	0
**1**	0.192	1	4.46	**46**	4.693	2	0	**90**	5.512	2	0
**2**	0.741	1	1.77	**47**	4.711	5	0.01	**91**	5.525	4	0
**3**	0.776	16	26.62	**48**	4.774	2	0	**92**	5.531	1	0
**4**	1.562	21	9.27	**49**	4.858	2	0	**93**	5.544	1	0
**5**	1.775	1	0.31	**50**	4.892	1	0	**94**	5.565	2	0
**6**	2.141	7	1.16	**51**	4.906	5	0.01	**95**	5.567	1	0
**7**	2.211	2	0.3	**52**	4.912	2	0	**96**	5.574	5	0
**8**	2.413	4	0.42	**53**	4.955	2	0	**97**	5.585	1	0
**9**	2.433	14	1.42	**54**	4.98	1	0	**98**	5.608	1	0
**10**	2.547	2	0.17	**55**	4.984	3	0	**99**	5.608	5	0
**11**	2.737	11	0.67	**56**	5.022	1	0	**100**	5.609	1	0
**12**	2.842	18	0.92	**57**	5.052	7	0.01	**101**	5.618	8	0
**13**	2.844	8	0.41	**58**	5.081	1	0	**102**	5.62	7	0
**14**	2.871	1	0.05	**59**	5.117	2	0	**103**	5.63	2	0
**15**	2.99	1	0.04	**60**	5.124	2	0	**104**	5.636	2	0
**16**	3.028	2	0.07	**61**	5.128	5	0.01	**105**	5.642	1	0
**17**	3.098	19	0.63	**62**	5.137	3	0	**106**	5.677	4	0
**18**	3.128	3	0.09	**63**	5.145	9	0.01	**107**	5.691	3	0
**19**	3.131	12	0.38	**64**	5.152	1	0	**108**	5.71	2	0
**20**	3.447	11	0.2	**65**	5.153	1	0	**109**	5.714	1	0
**21**	3.467	7	0.12	**66**	5.158	7	0.01	**110**	5.724	3	0
**22**	3.508	9	0.15	**67**	5.179	1	0	**111**	5.744	2	0
**23**	3.593	2	0.03	**68**	5.247	7	0.01	**112**	5.769	1	0
**24**	3.65	5	0.07	**69**	5.25	6	0.01	**113**	5.77	1	0
**25**	3.689	10	0.12	**70**	5.25	3	0	**114**	5.782	5	0
**26**	3.763	11	0.12	**71**	5.252	4	0	**115**	5.786	1	0
**27**	3.77	1	0.01	**72**	5.263	2	0	**116**	5.794	1	0
**28**	3.772	11	0.12	**73**	5.271	1	0	**117**	5.802	4	0
**29**	3.805	9	0.09	**74**	5.29	5	0	**118**	5.816	1	0
**30**	3.831	1	0.01	**75**	5.32	6	0	**119**	5.82	13	0
**31**	3.845	1	0.01	**76**	5.337	3	0	**120**	5.822	2	0
**32**	3.875	2	0.02	**77**	5.343	2	0	**121**	5.846	2	0
**33**	3.945	4	0.03	**78**	5.367	1	0	**122**	5.85	1	0
**34**	4.057	9	0.06	**79**	5.371	1	0	**123**	5.85	1	0
**35**	4.11	4	0.02	**80**	5.392	11	0.01	**124**	5.88	1	0
**36**	4.111	2	0.01	**81**	5.397	6	0	**125**	5.885	7	0
**37**	4.115	1	0.01	**82**	5.425	2	0	**126**	5.886	3	0
**38**	4.222	1	0	**83**	5.427	6	0	**127**	5.957	1	0
**39**	4.254	3	0.01	**84**	5.437	1	0	**128**	5.985	3	0
**40**	4.299	5	0.02	**85**	5.45	1	0	**129**	5.989	2	0
**41**	4.34	3	0.01	**86**	5.469	1	0	**130**	5.991	1	0
**42**	4.414	2	0.01	**87**	5.486	4	0	**131**	5.993	2	0
**43**	4.556	10	0.03	**88**	5.499	11	0.01	**132**	5.998	1	0
**44**	4.572	6	0.02								

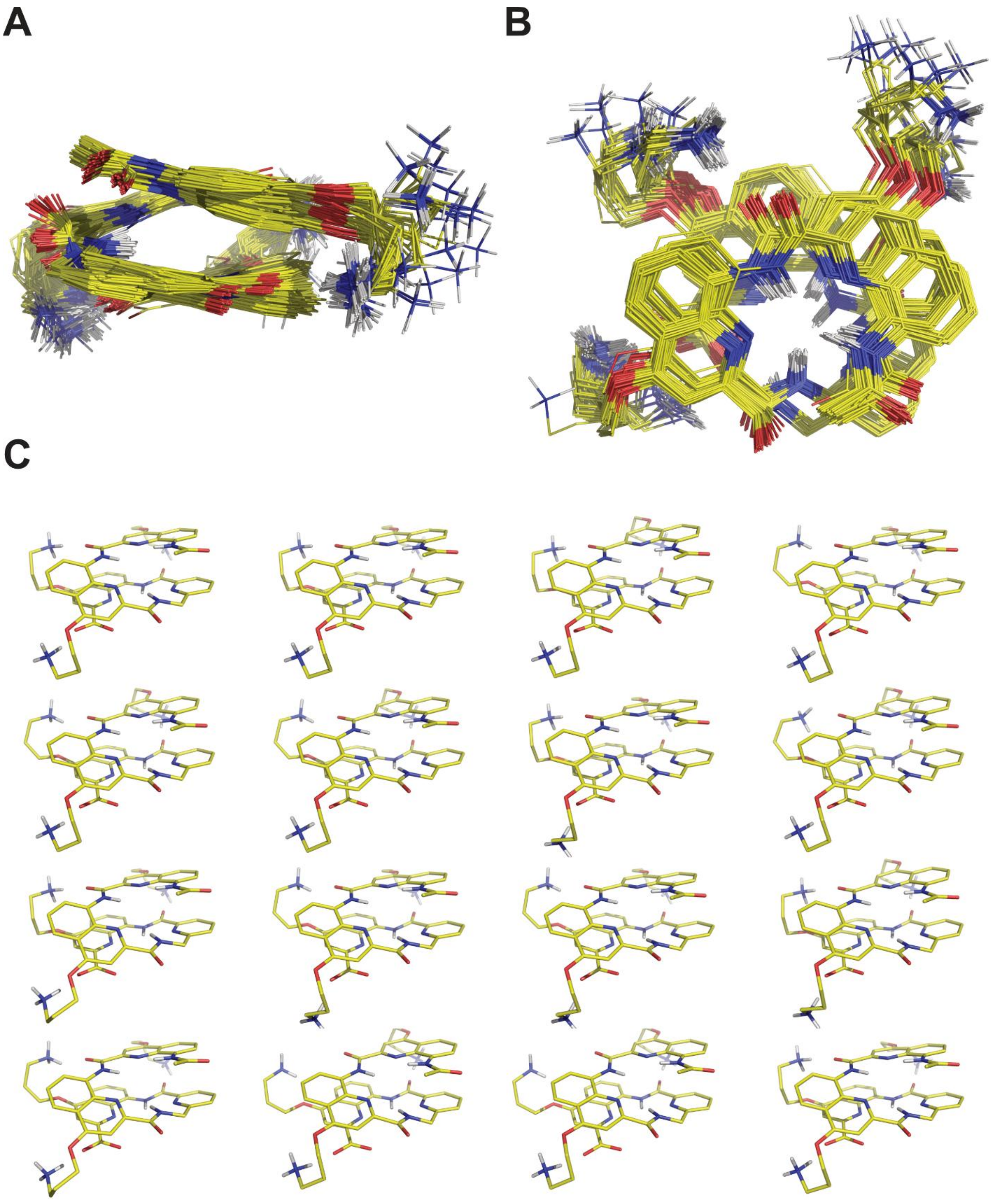

Figure S138. Conformational ensemble of QQPQ: Side (A) and Top (B) view of the 133 superimposed identified conformers, and comparison of the 16 lowest energy conformers (C; < 3 kcal/mol difference with the global minimum).

QQPQ/222T

Structures mid-production

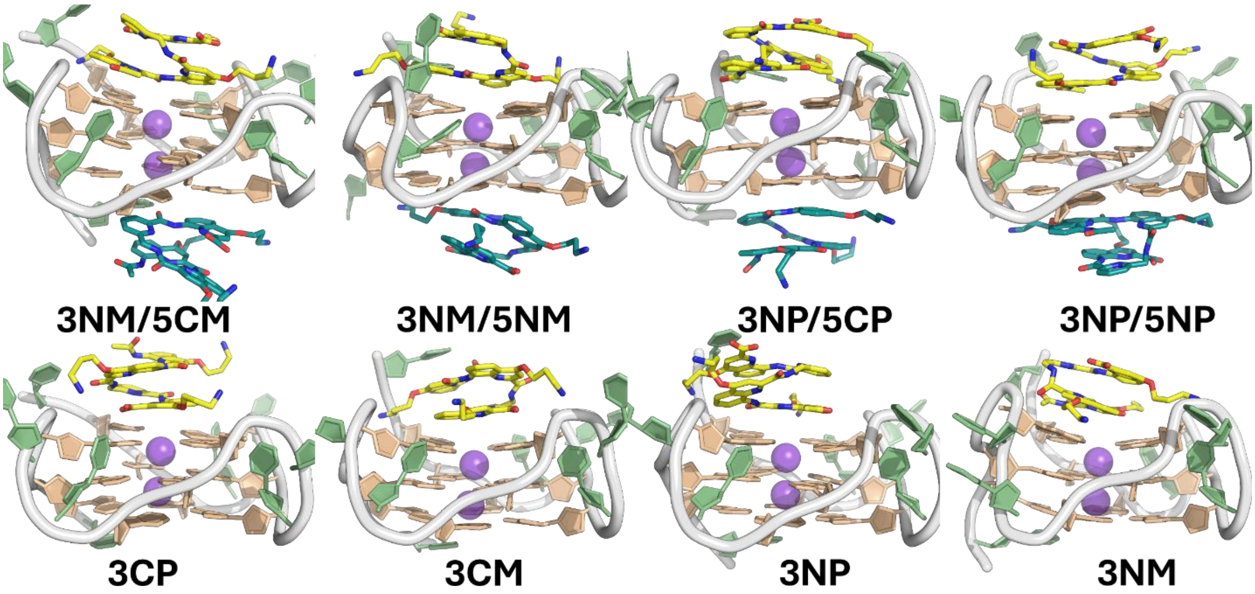

Figure S139. Minimized, heated and equilibrated structures of the 1:1 (bottom) and 2:1 (top) QQPQ:222T complexes submitted to 1 microsecond MD simulations, here shown after 500 ns.

RMSD

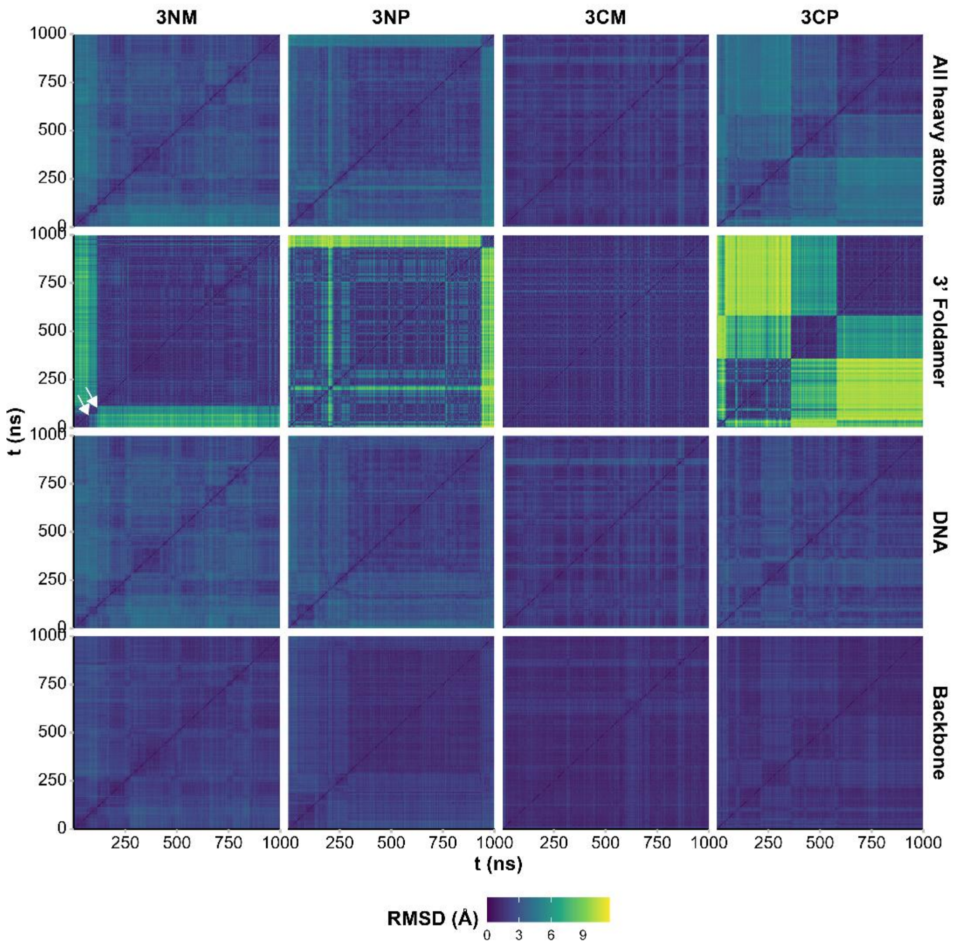

Figure S140. Pairwise RMSD of 1:1 QQPQ:222T complexes calculated on all heavy atoms, and only on the foldamer, the DNA, and the DNA backbone. Lighter colors points to structural changes, while similar-colored squares along the diagonal indicate a relative structural stability during the corresponding time range. An example of foldamer rotation events are indicated by white arrows for 3NM.

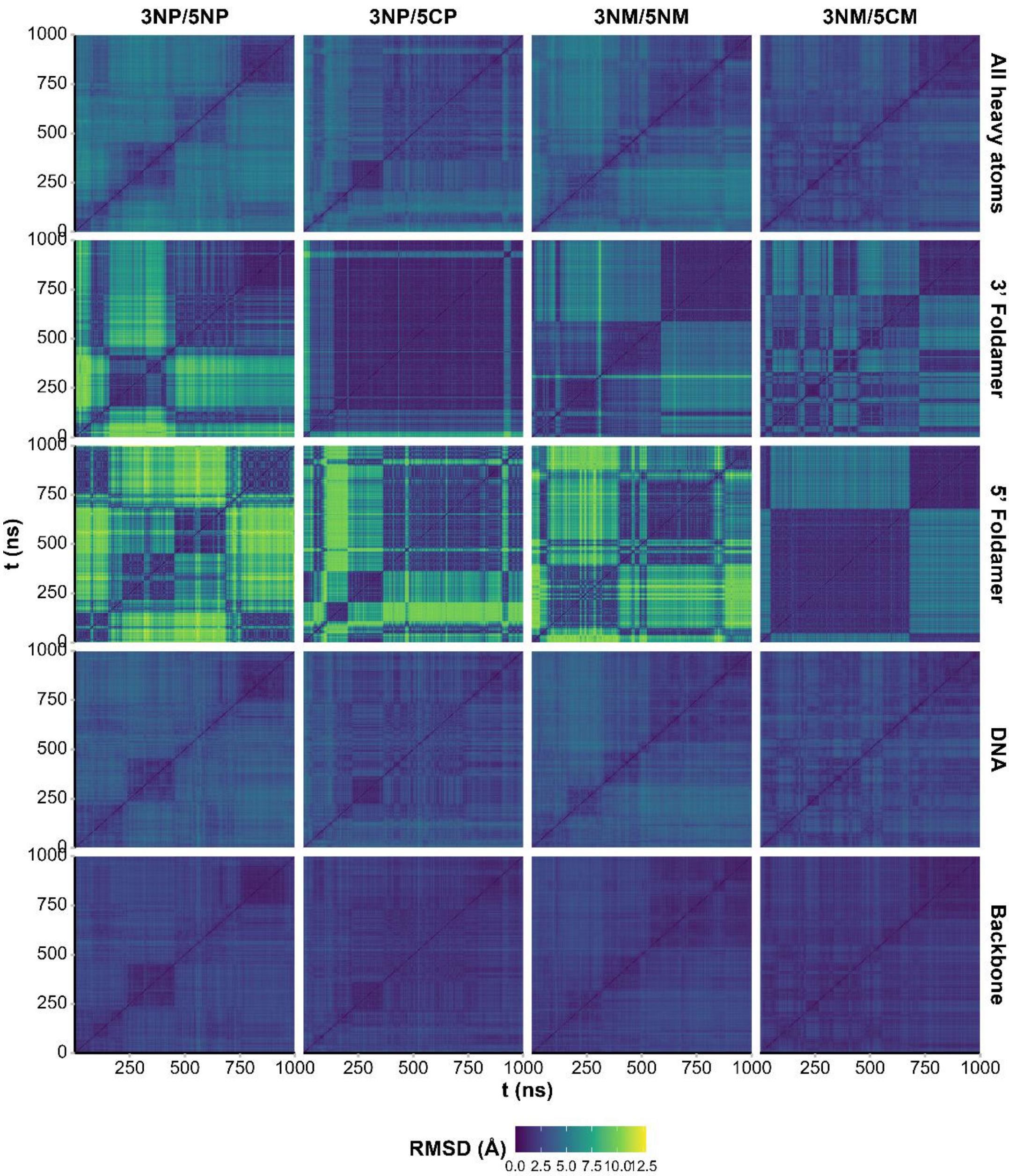

Figure S141. Pairwise RMSD of 2:1 QQPQ:222T complexes calculated on all heavy atoms, and only on either foldamer, the DNA, and the DNA backbone. Lighter colors points to structural changes, while similar-colored squares along the diagonal indicate a relative structural stability during the corresponding time range.

Tetrad stability

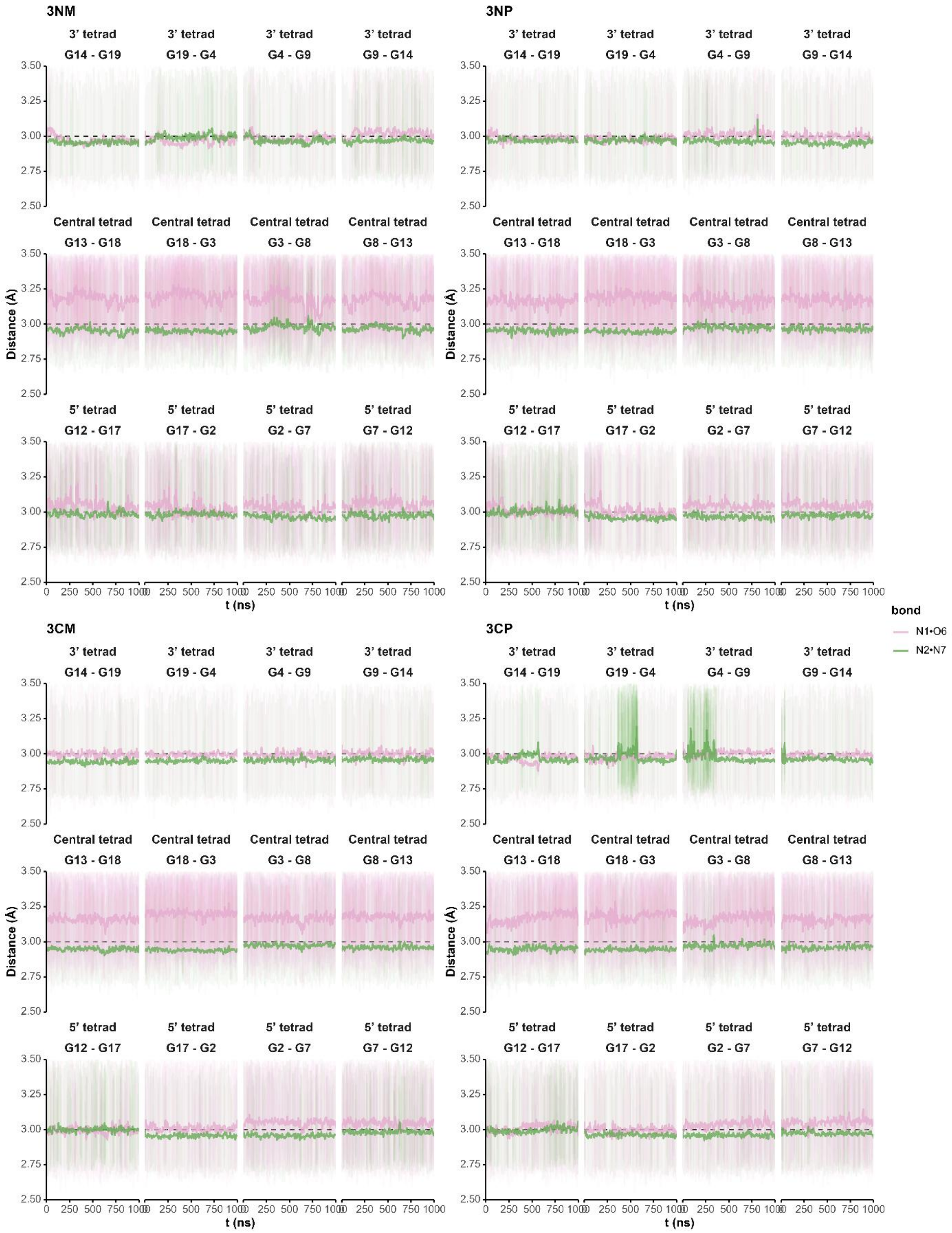

Figure S142. H-bonds within tetrads are stable for all 1:1 complexes, here shown through the donor-acceptor distance during the trajectory.

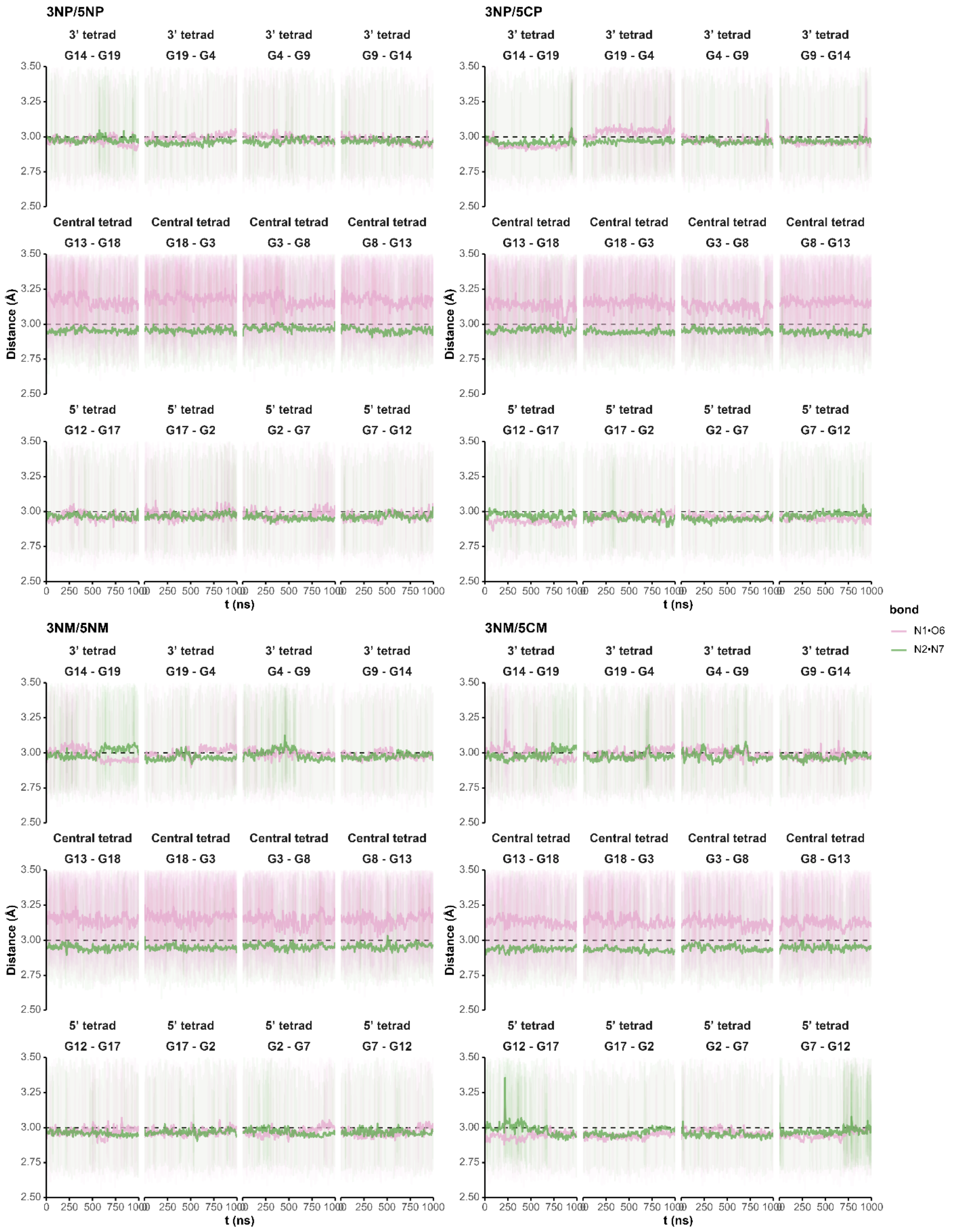

Figure S143. H-bonds within tetrads are stable for all 2:1 complexes, here shown through the donor-acceptor distance during the trajectory.

Principal component analysis

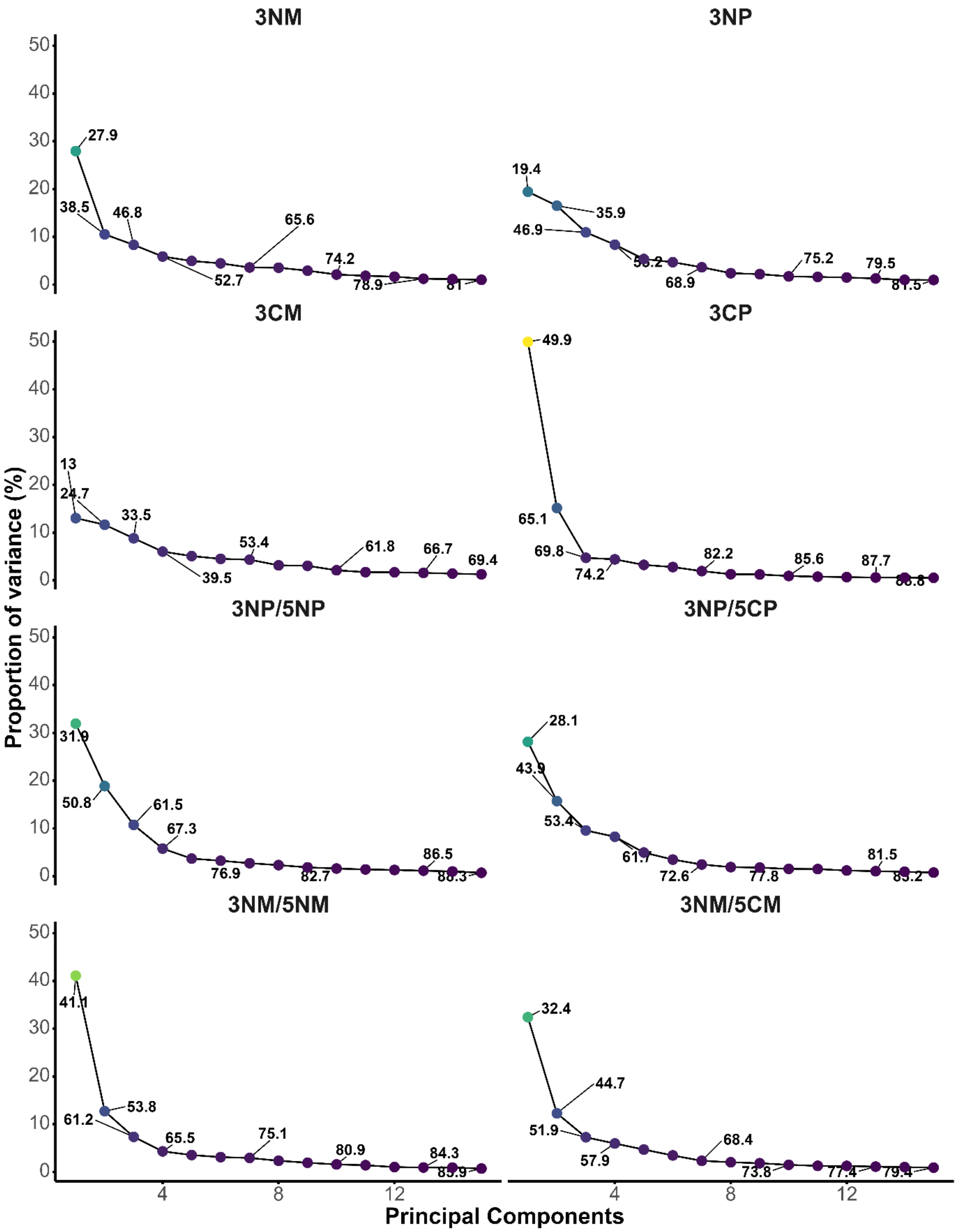

Figure S144. Scree plots for the PCA of the 8 molecular dynamics trajectory. The labels indicate the cumulative proportion of variance. Only the first 2—4 principal components account for significant proportions of the variance.

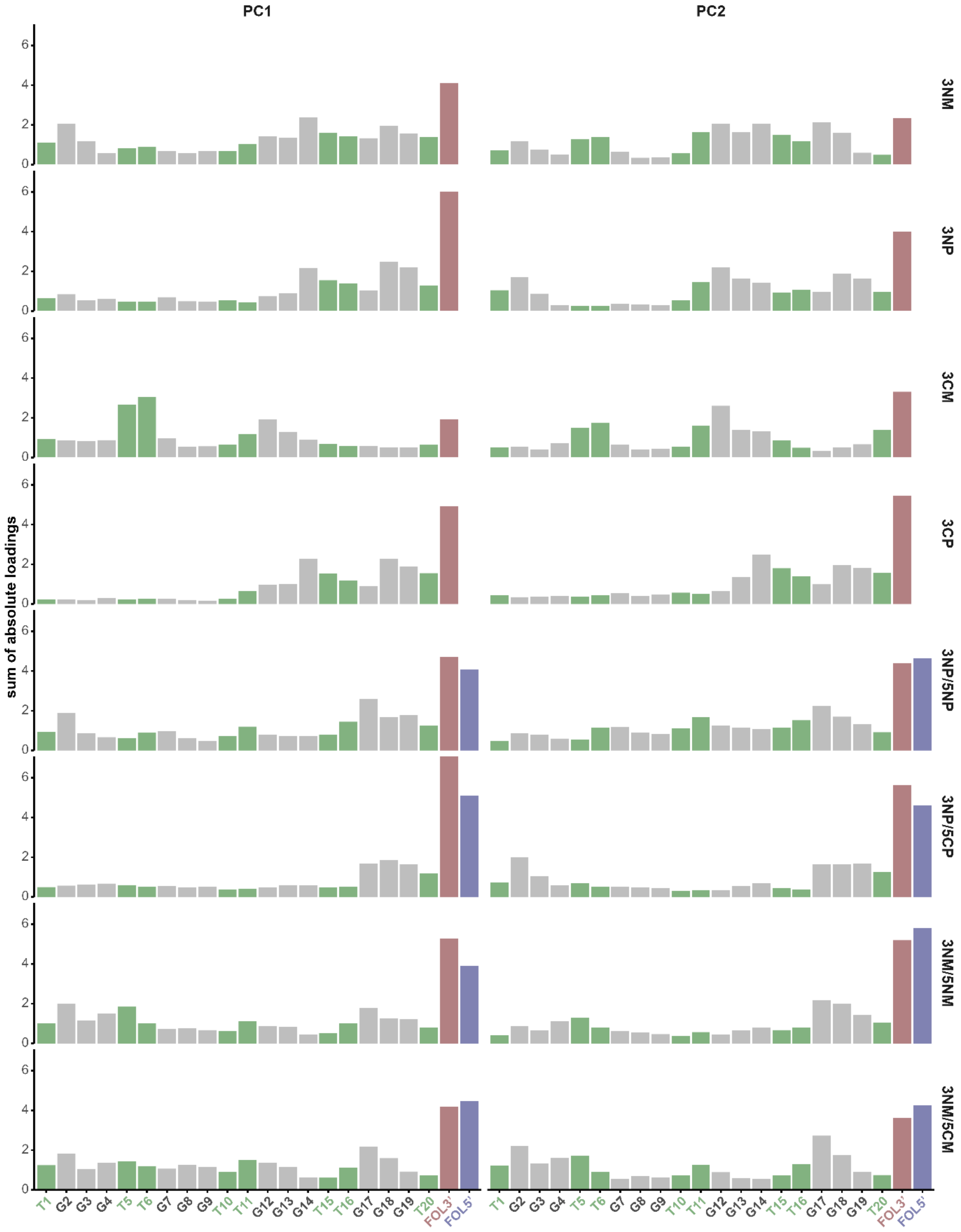

Figure S145. Sum of absolute loadings for the PCA of the 8 molecular dynamics trajectory for the first two components. The contribution of QQPQ bound in 3' (red), 5' (blue), and thymine residues (green) are colored. QQPQ contribute the most to these principal components.

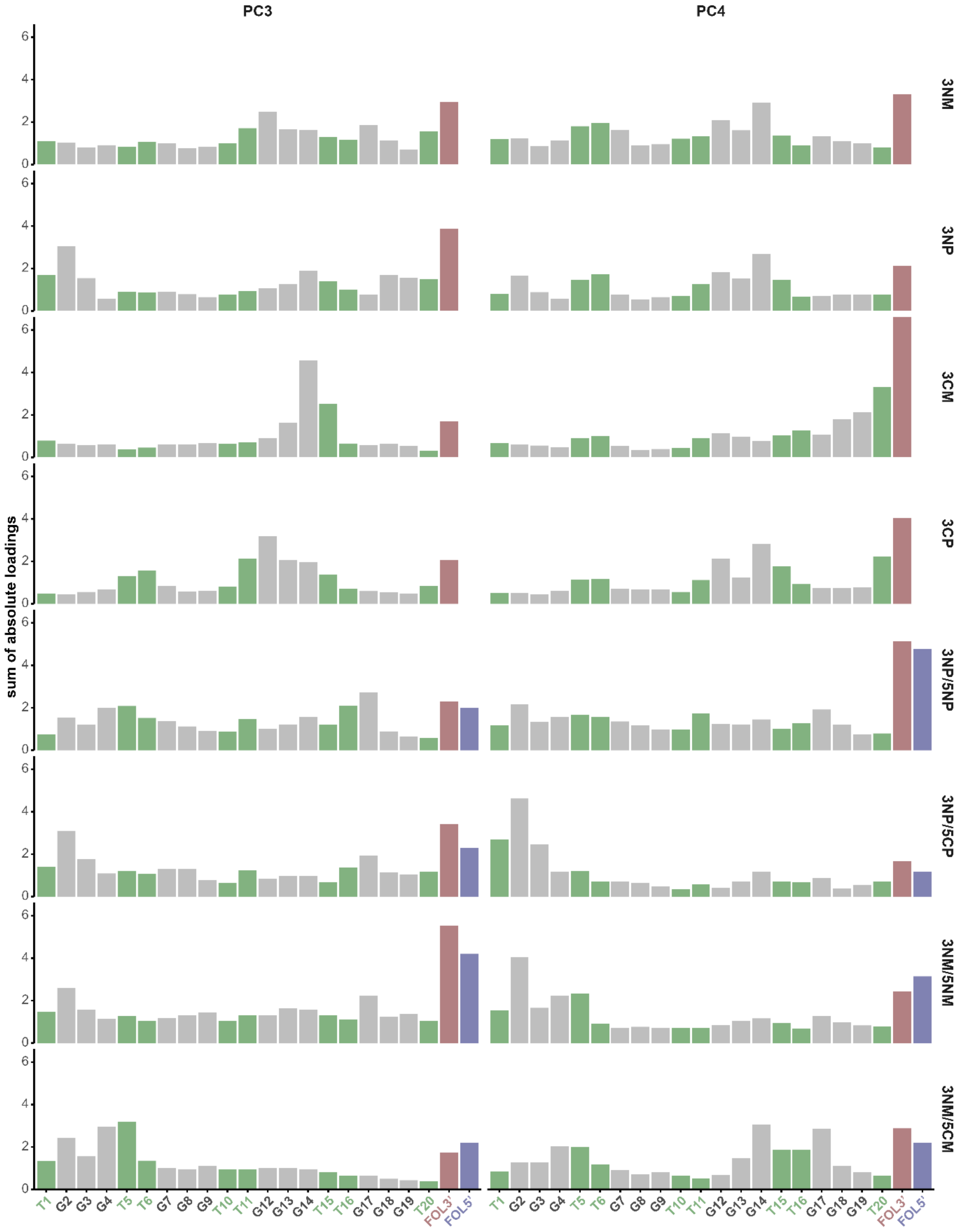

Figure S146. Sum of absolute loadings for the PCA of the 8 molecular dynamics trajectory for the third and fourth components. The contribution of QQPQ bound in 3' (red), 5' (blue), and thymine residues (green) are colored. QQPQ contribute the most to these principal components.

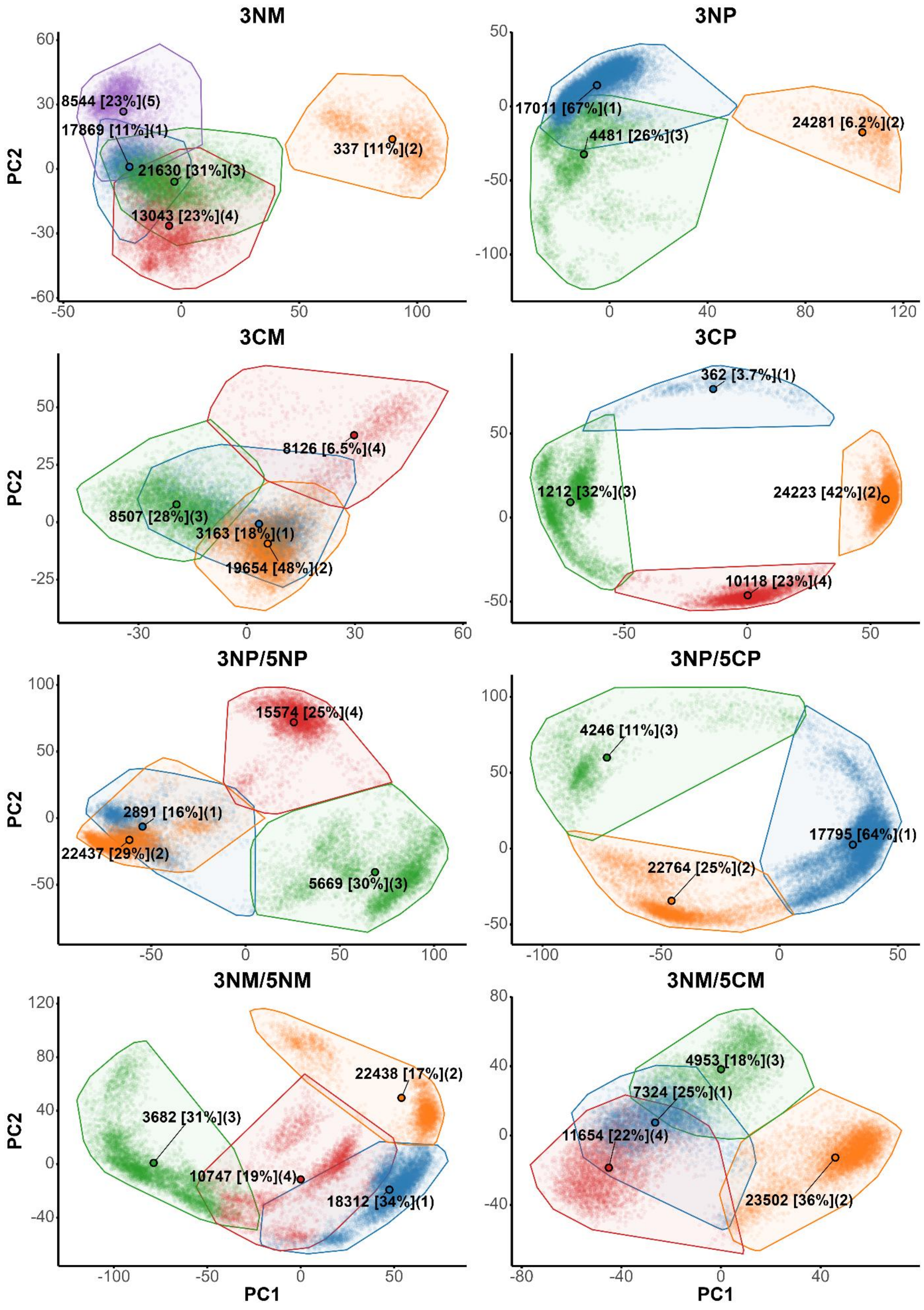

Figure S147. PCA plots for the 8 molecular dynamics trajectories. Each point represents a frame of the trajectory and is colored by k-mean cluster. The centroid of each cluster is labelled with the frame number, percentage of frames belonging to this cluster and cluster number in brackets.

PCA clusters

3NM

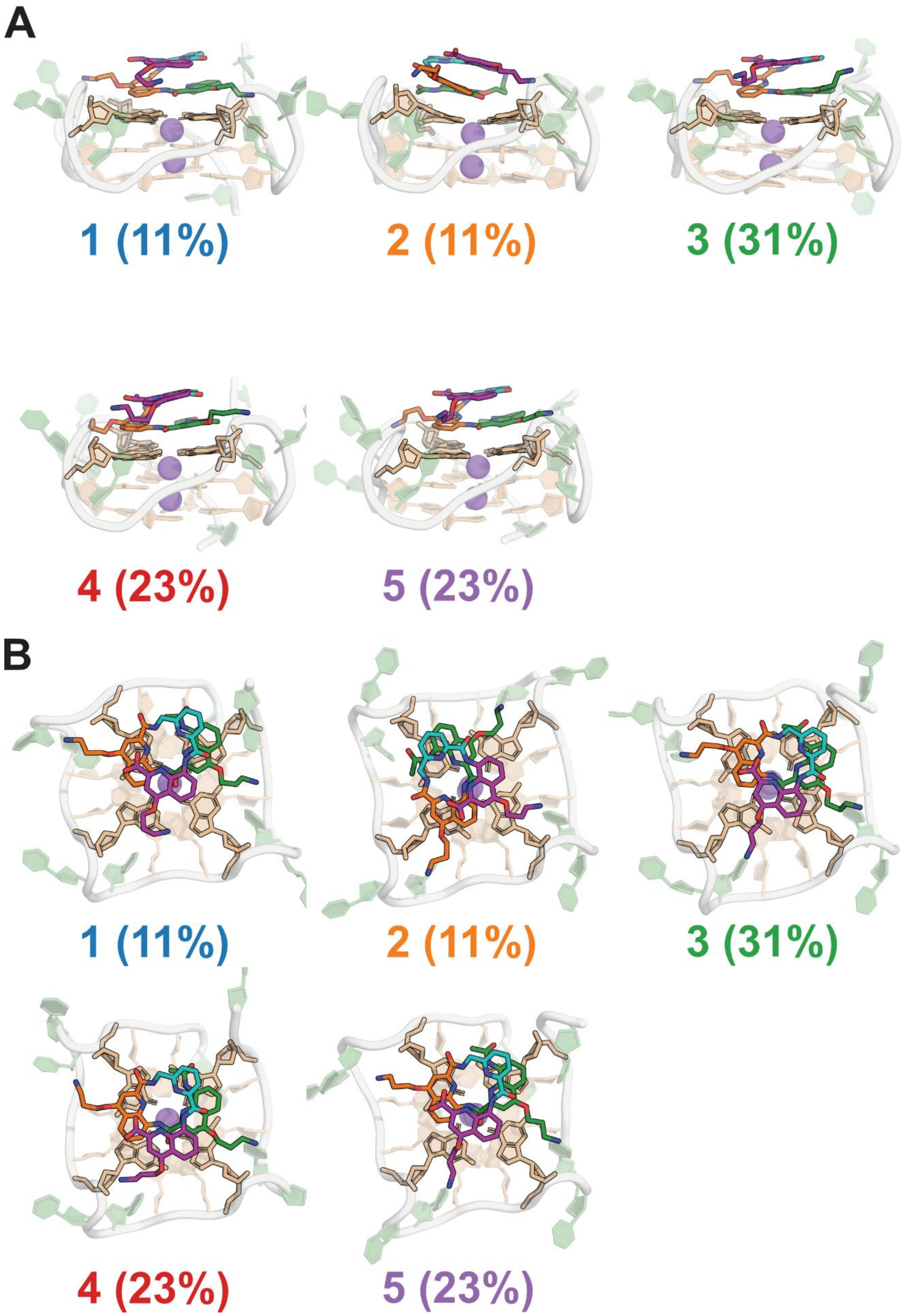

Figure S148. Side (A) and top (B) views of the 3NM model clusters (percentage of frame belonging to clusters indicated in brackets). The aromatic cycles of QQPQ are colored in green (Q1), orange (Q2), blue (P3) and and purple (Q4).

3NP

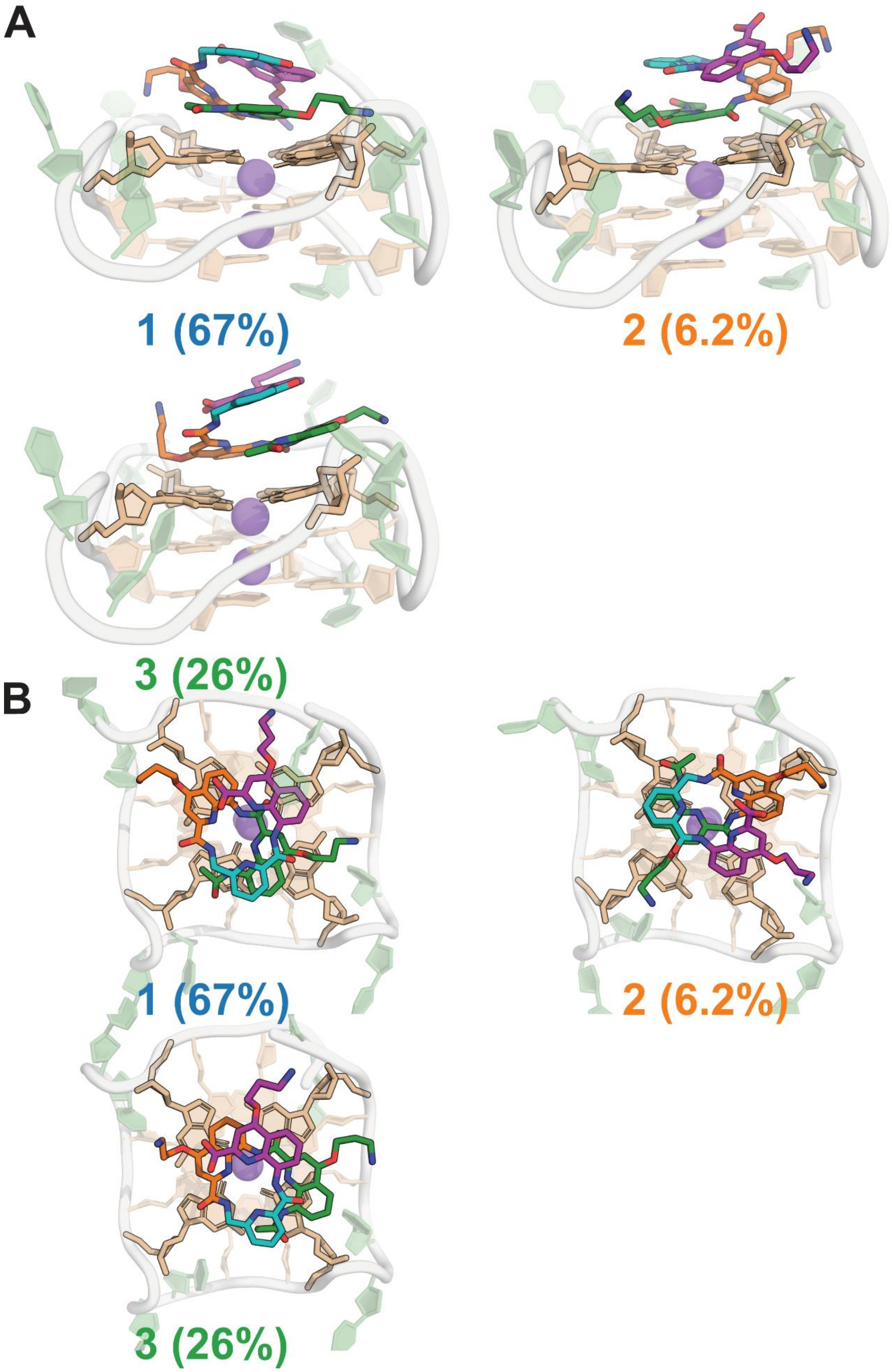

Figure S149. Side (A) and top (B) views of the 3NP model clusters (percentage of frame belonging to clusters indicated in brackets). The aromatic cycles of QQPQ are colored in green (Q1), orange (Q2), blue (P3) and and purple (Q4).

3CM

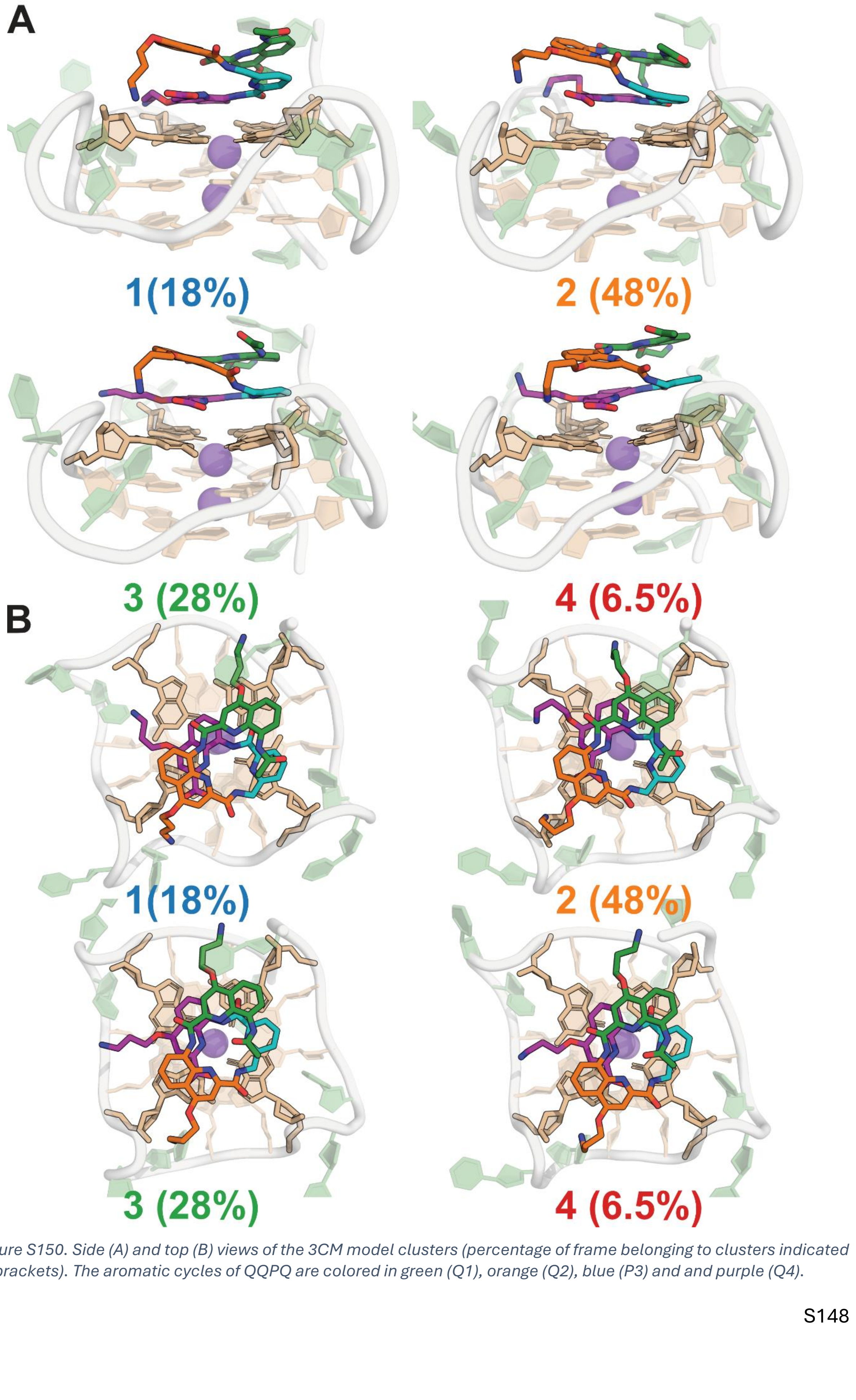

Figure S150. Side (A) and top (B) views of the 3CM model clusters (percentage of frame belonging to clusters indicated in brackets). The aromatic cycles of QQPQ are colored in green (Q1), orange (Q2), blue (P3) and and purple (Q4).

3CP

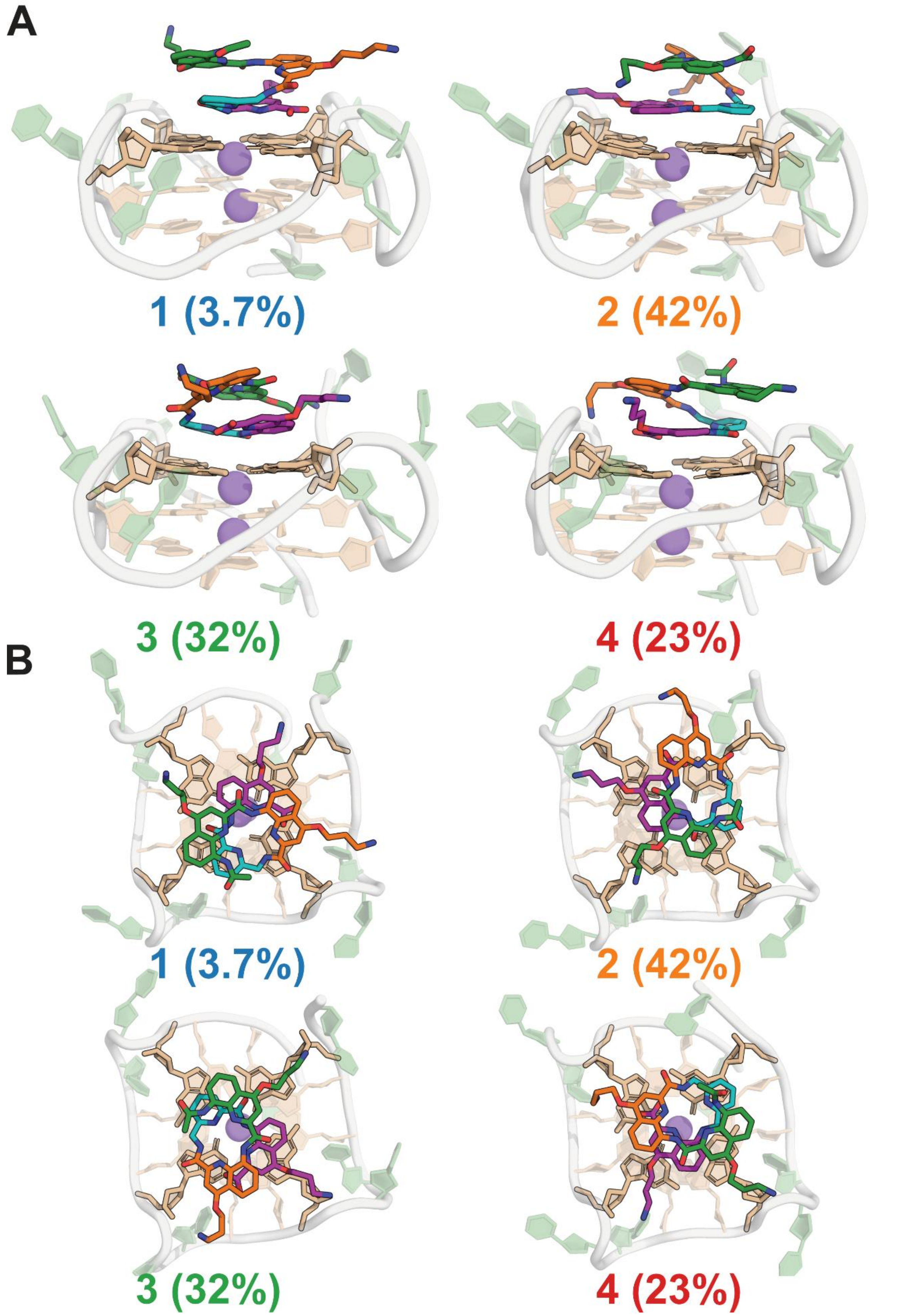

Figure S151. Side (A) and top (B) views of the 3CP model clusters (percentage of frame belonging to clusters indicated in brackets). The aromatic cycles of QQPQ are colored in green (Q1), orange (Q2), blue (P3) and and purple (Q4).

3NP/5NP

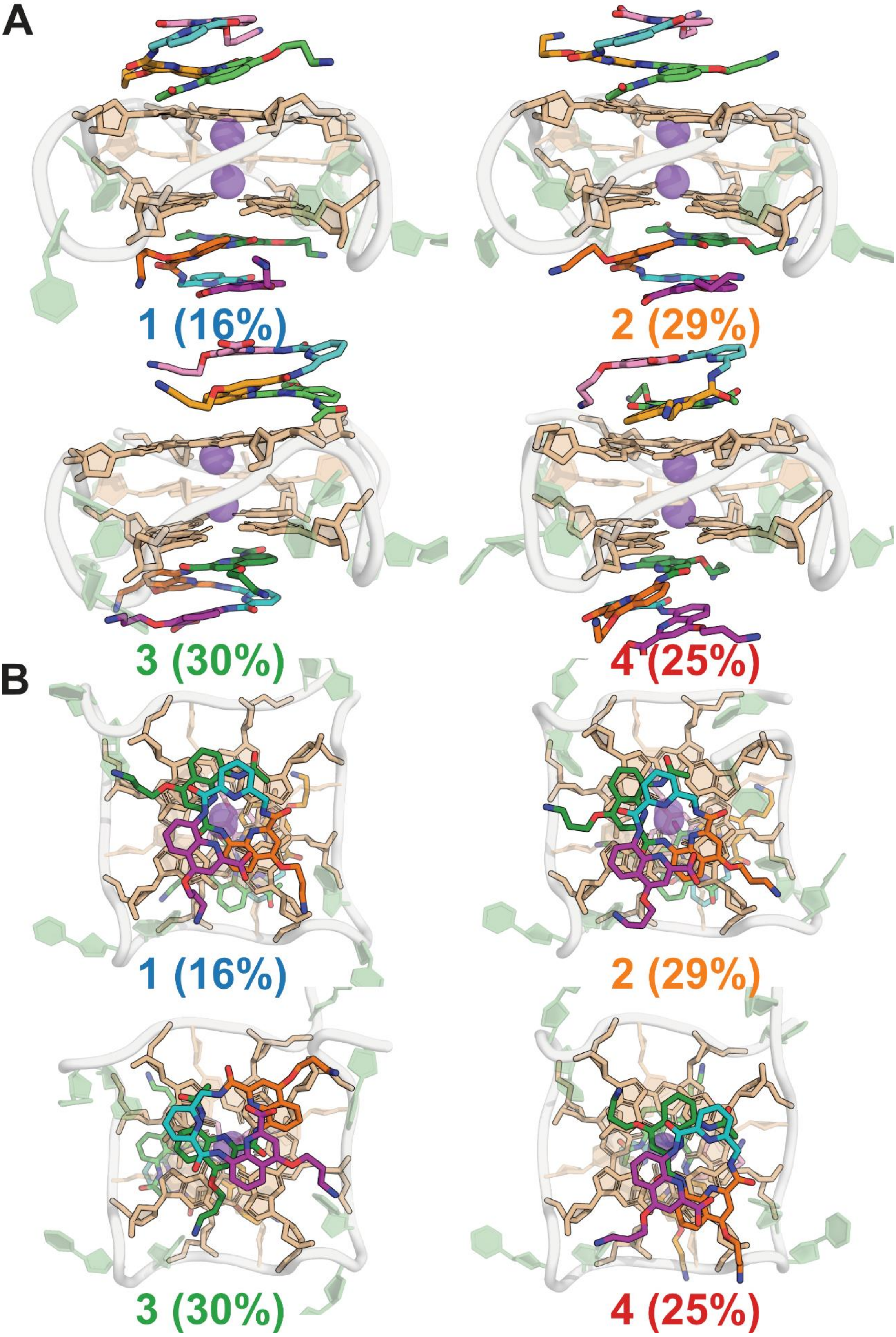

Figure S152. Side (A; 5' end on top) and top (B; 5' end) views of the 3NP/5NP model clusters (percentage of frame belonging to clusters indicated in brackets). The aromatic cycles of QQPQ are colored in green (Q1), orange (Q2), blue (P3) and and purple (Q4), with lighter colors for the 5' binding site.

3NP/5CP

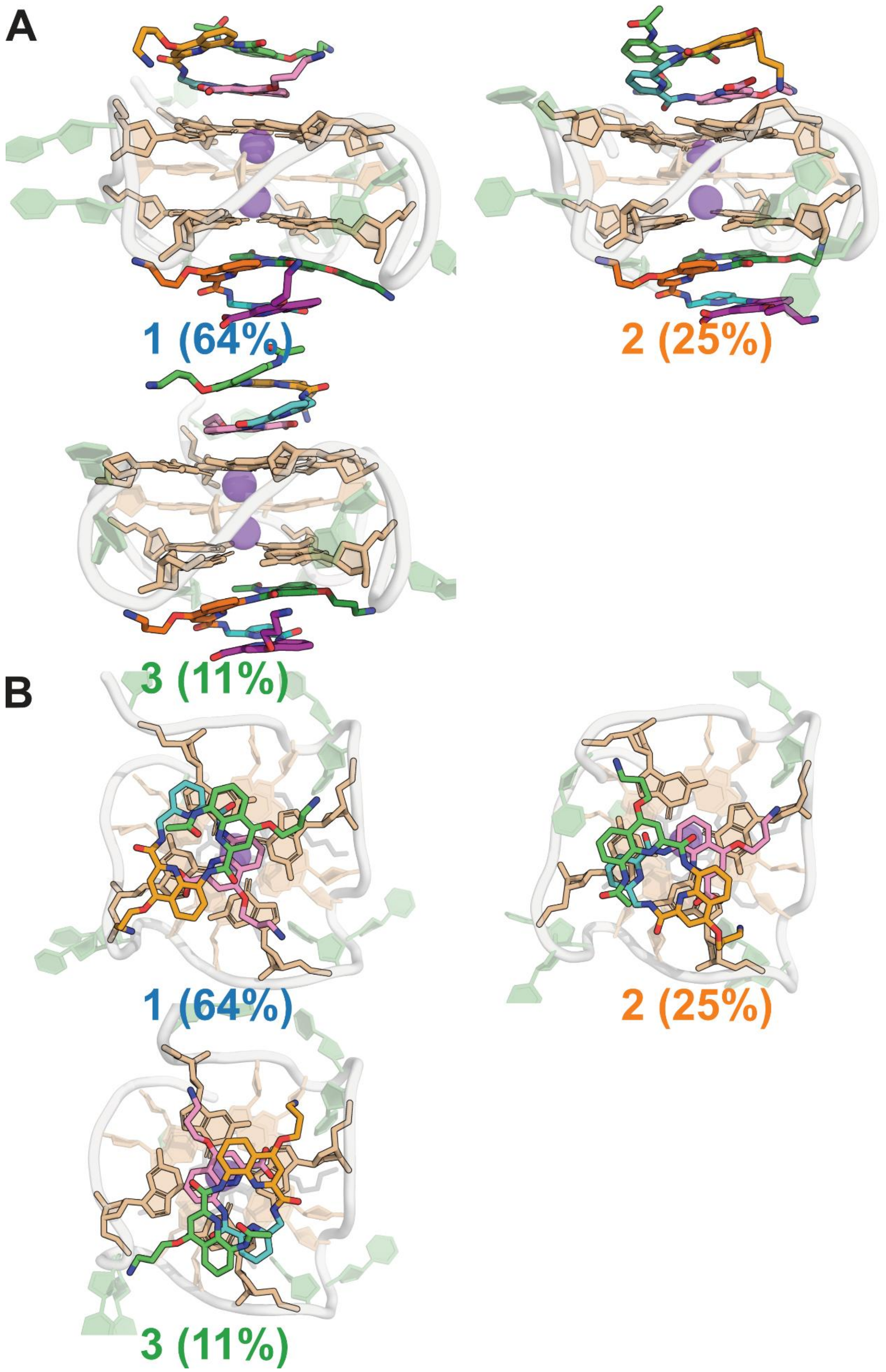

Figure S153. Side (A; 5' end on top) and top (B; 5' end) views of the 3NP/5CP model clusters (percentage of frame belonging to clusters indicated in brackets). The aromatic cycles of QQPQ are colored in green (Q1), orange (Q2), blue (P3) and and purple (Q4), with lighter colors for the 5' binding site.

3NM/5NM

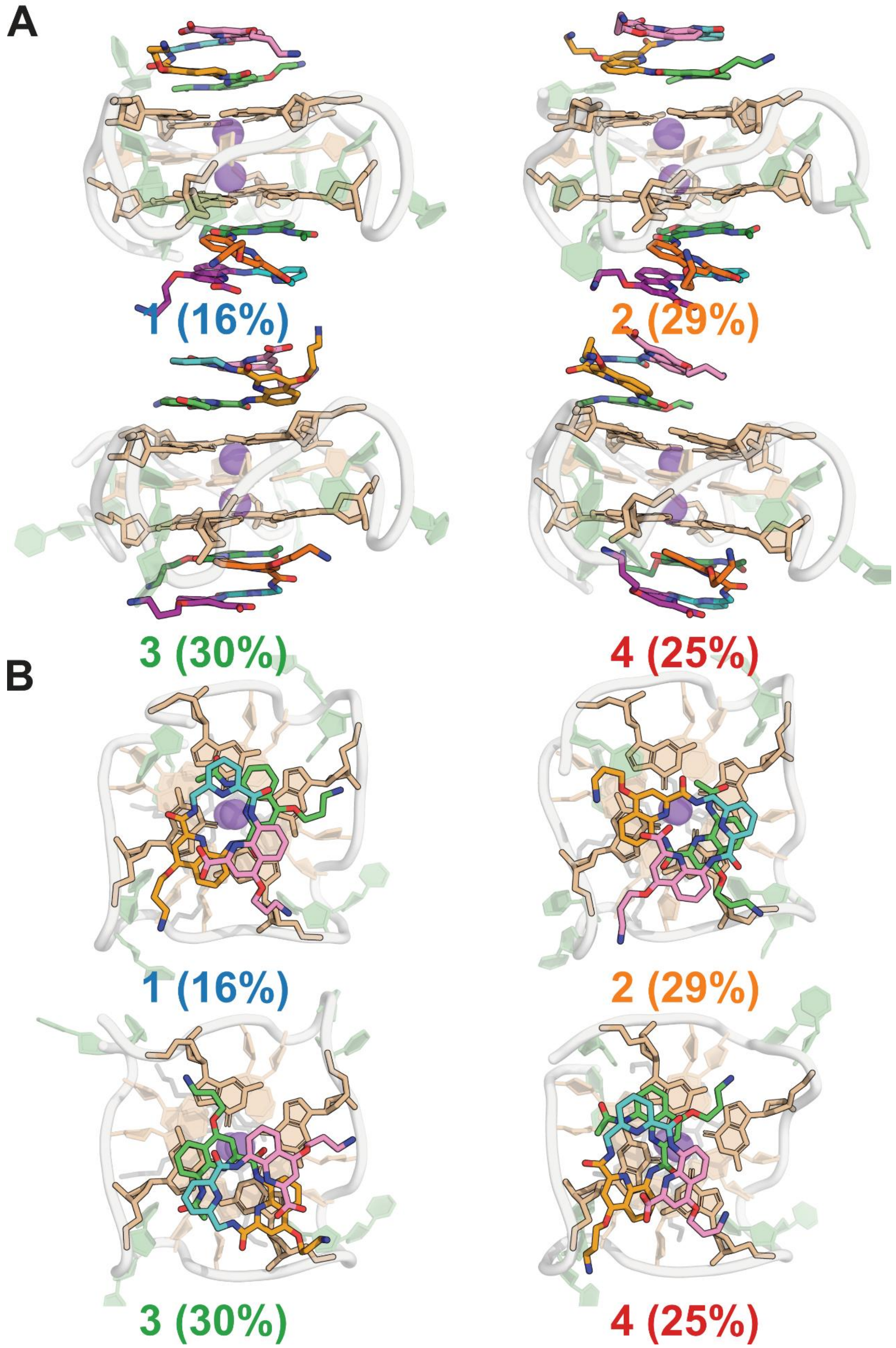

Figure S154. Side (A; 5' end on top) and top (B; 5' end) views of the 3NM/5NM model clusters (percentage of frame belonging to clusters indicated in brackets). The aromatic cycles of QQPQ are colored in green (Q1), orange (Q2), blue (P3) and and purple (Q4), with lighter colors for the 5' binding site.

3NM/5CM

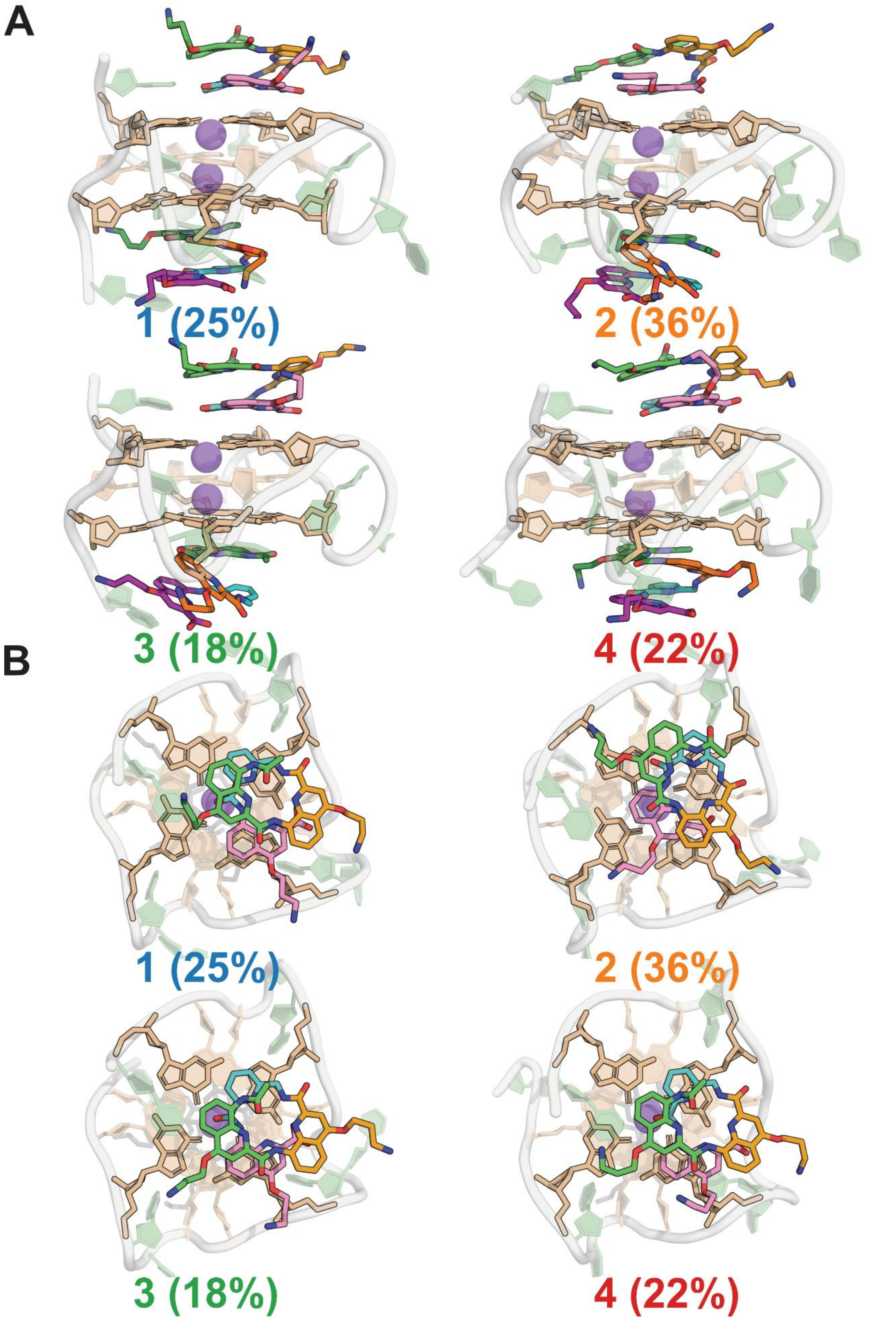

Figure S155. Side (A; 5' end on top) and top (B; 5' end) views of the 3NM/5CM model clusters (percentage of frame belonging to clusters indicated in brackets). The aromatic cycles of QQPQ are colored in green (Q1), orange (Q2), blue (P3) and and purple (Q4), with lighter colors for the 5' binding site.

Side-chain H-bonding

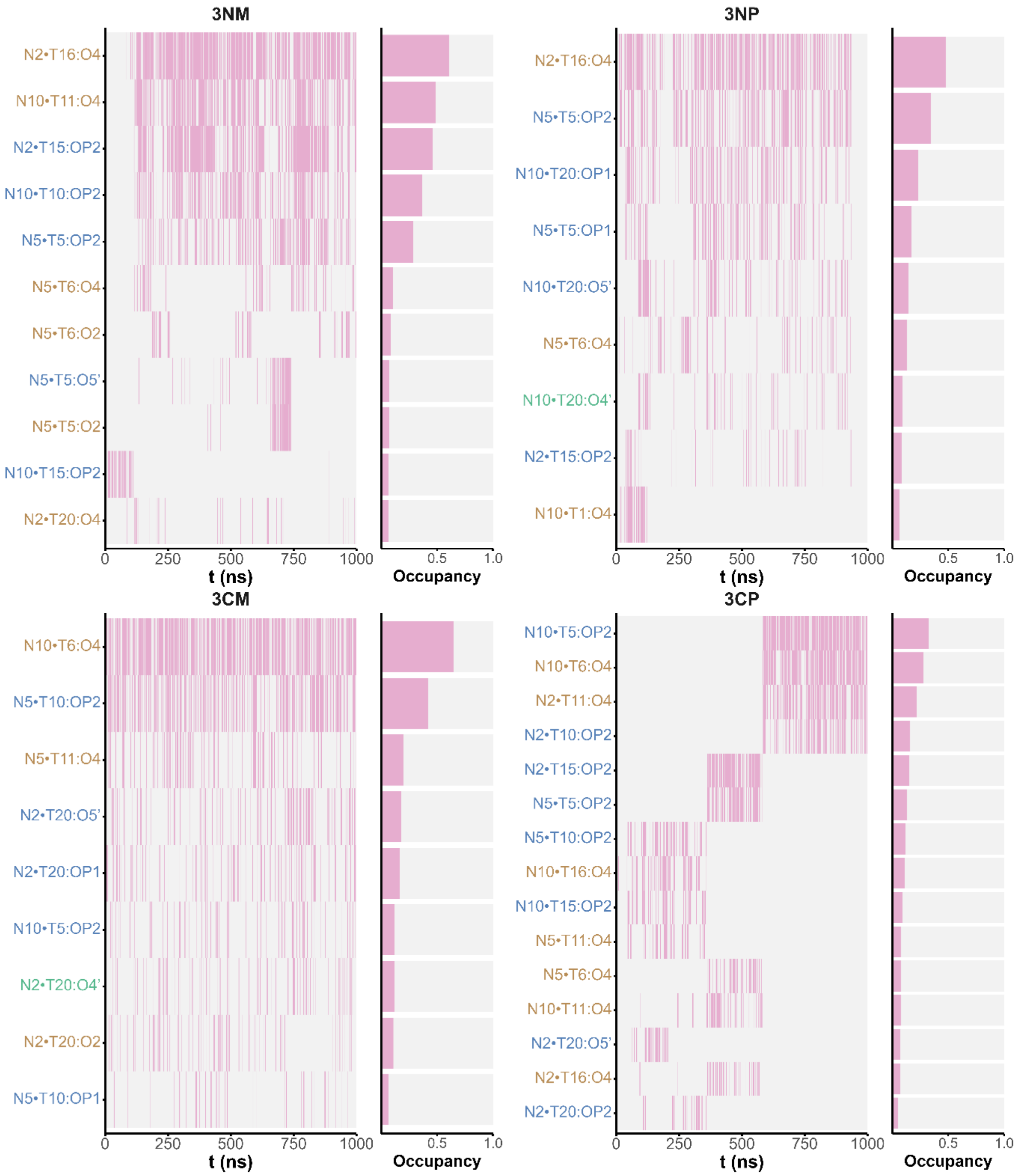

Figure S156. Frequency of side-chain H-bond formation during the simulations of 1:1 complexes. In left panels, pink lines indicate the formation of an H-bond; time range with stable bond formation produce pink rectangles. Right panels summarize the frequency of formation of the corresponding bonds. Pairs are colored in blue, green and brown if they involve the backbone, sugar and bases of DNA, respectively.

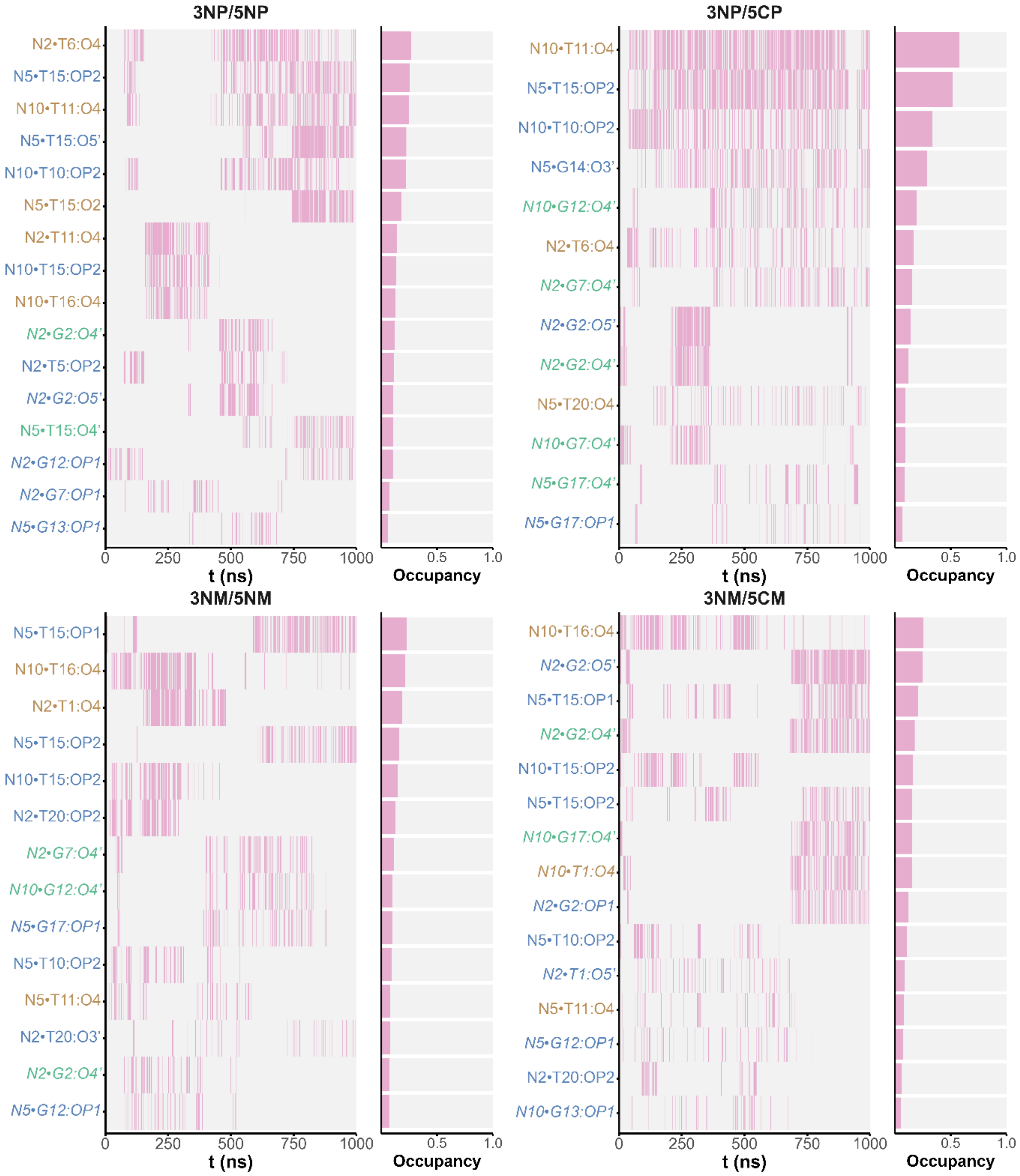

Figure S157. Frequency of side-chain H-bond formation during the simulations of 2:1 QQPQ:DNA complexes. In left panels, pink lines indicate the formation of an H-bond; time range with stable bond formation produce pink rectangles. Right panels summarize the frequency of formation of the corresponding bonds. Pairs are colored in blue, green and brown if they involve the backbone, sugar and bases of DNA, respectively. Pairs in italics denote the binding in 5', others are in 3'.

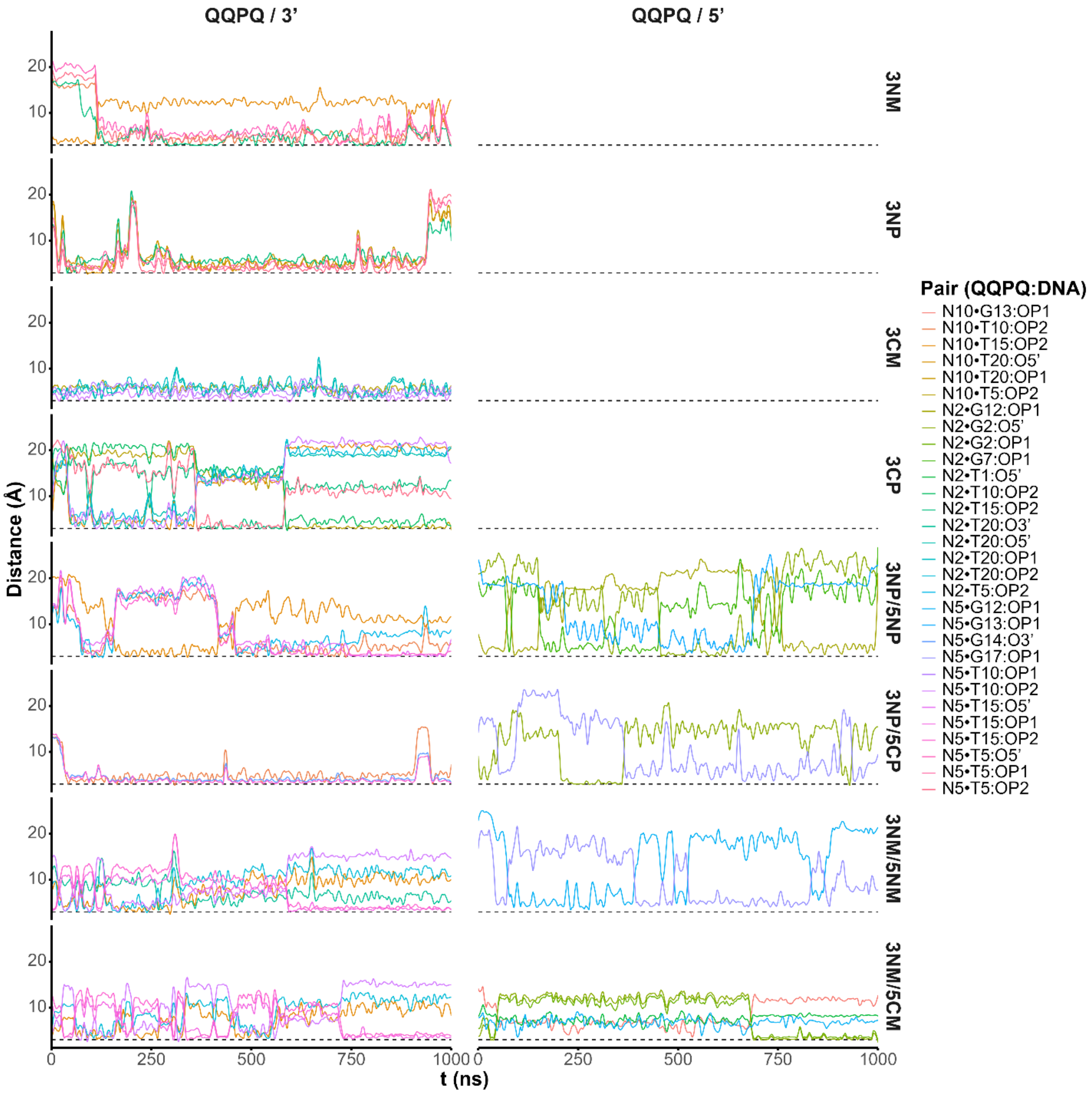

Figure S158. Donor-acceptor distances of H-bonds involving the ammonium groups of quinoline side chains and the DNA backbone. Only contacts for which the distance is below 3.1 Å with a frequency > 0.1 are shown for the sake of clarity.

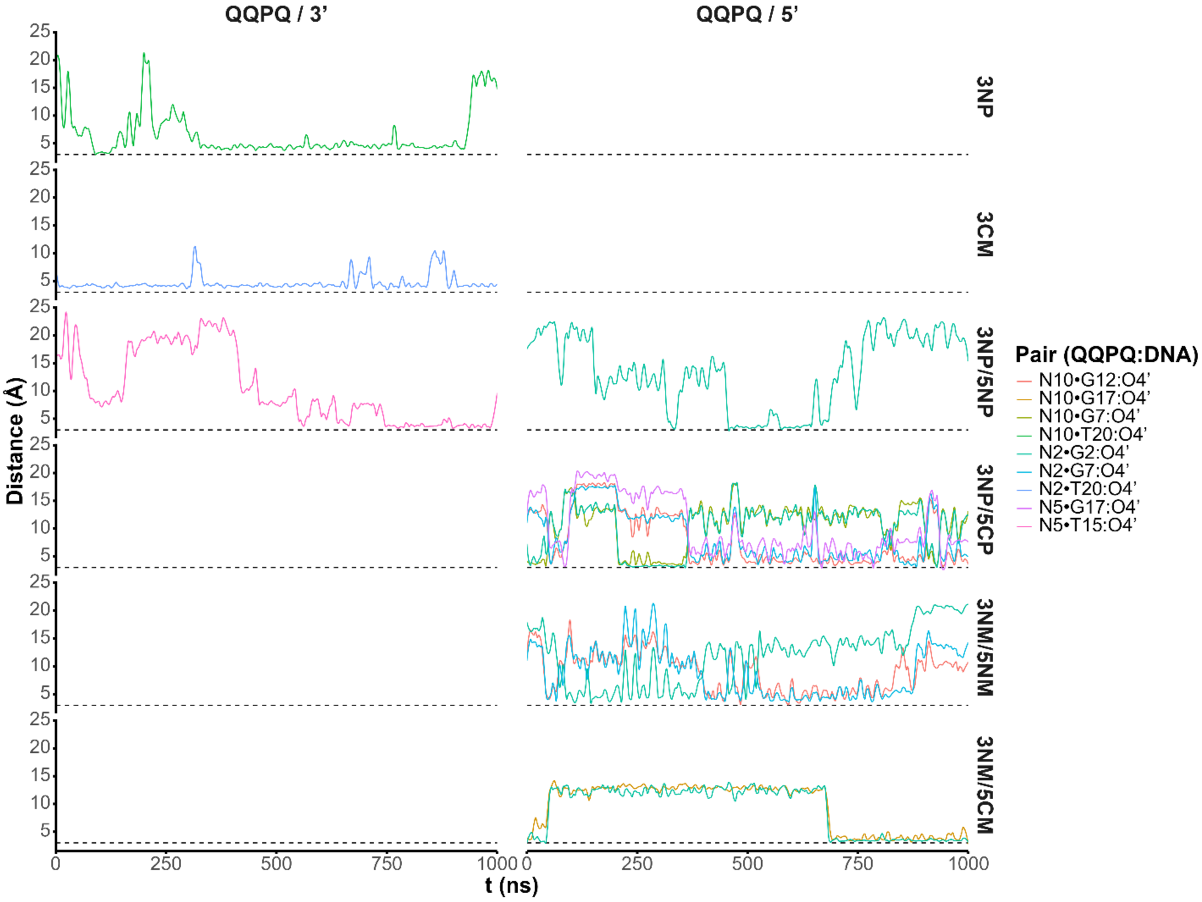

Figure S159. Donor-acceptor distances of H-bonds involving the ammonium groups of quinoline side chains and the DNA sugars. Only contacts for which the distance is below 3.1 Å with a frequency > 0.1 are shown for the sake of clarity.

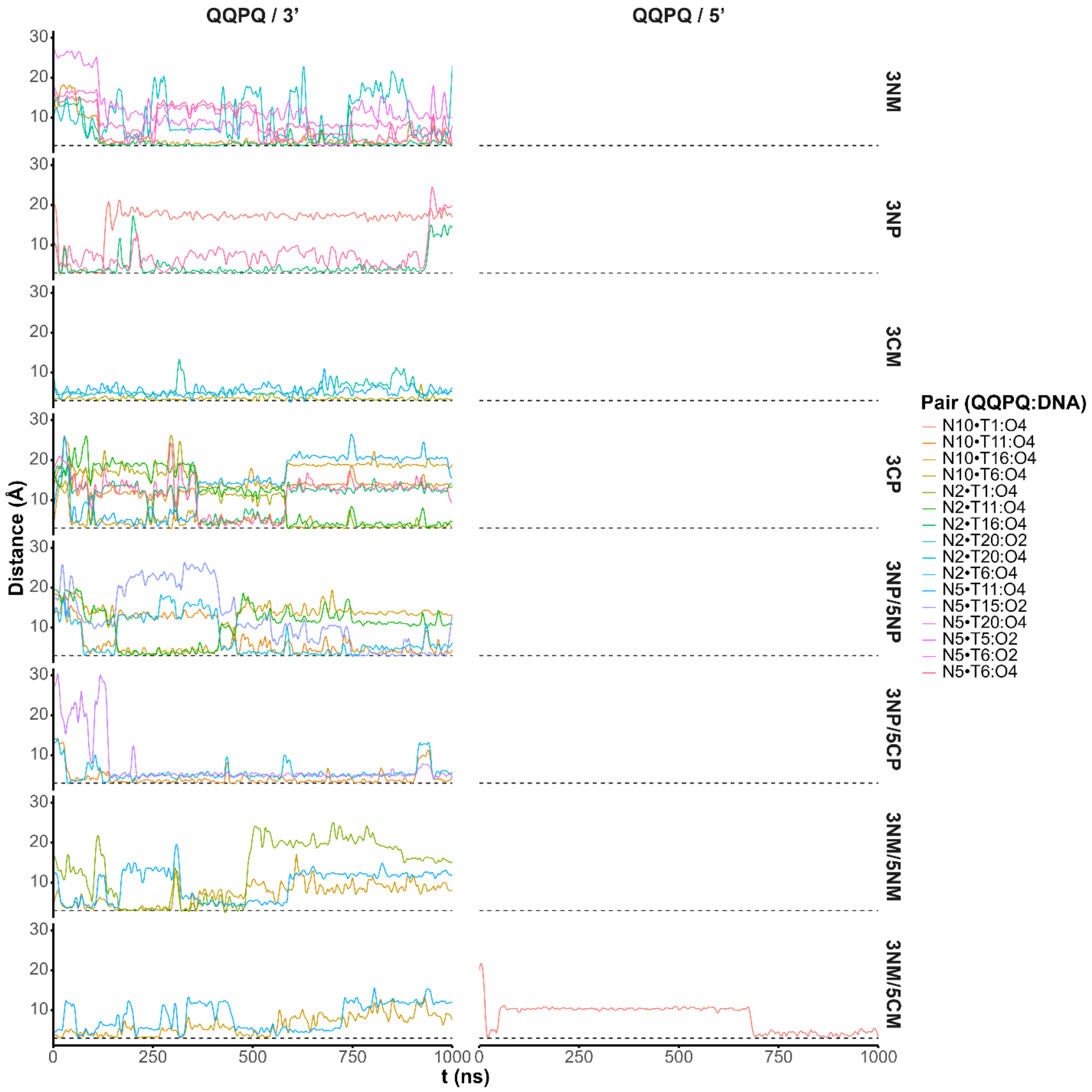

Figure S160. Donor-acceptor distances of H-bonds involving the ammonium groups of quinoline side chains and the DNA thymines from loop and termini (T1 and T20). Only contacts for which the distance is below 3.1 Å with a frequency > 0.1 are shown for the sake of clarity.

Stacking distances

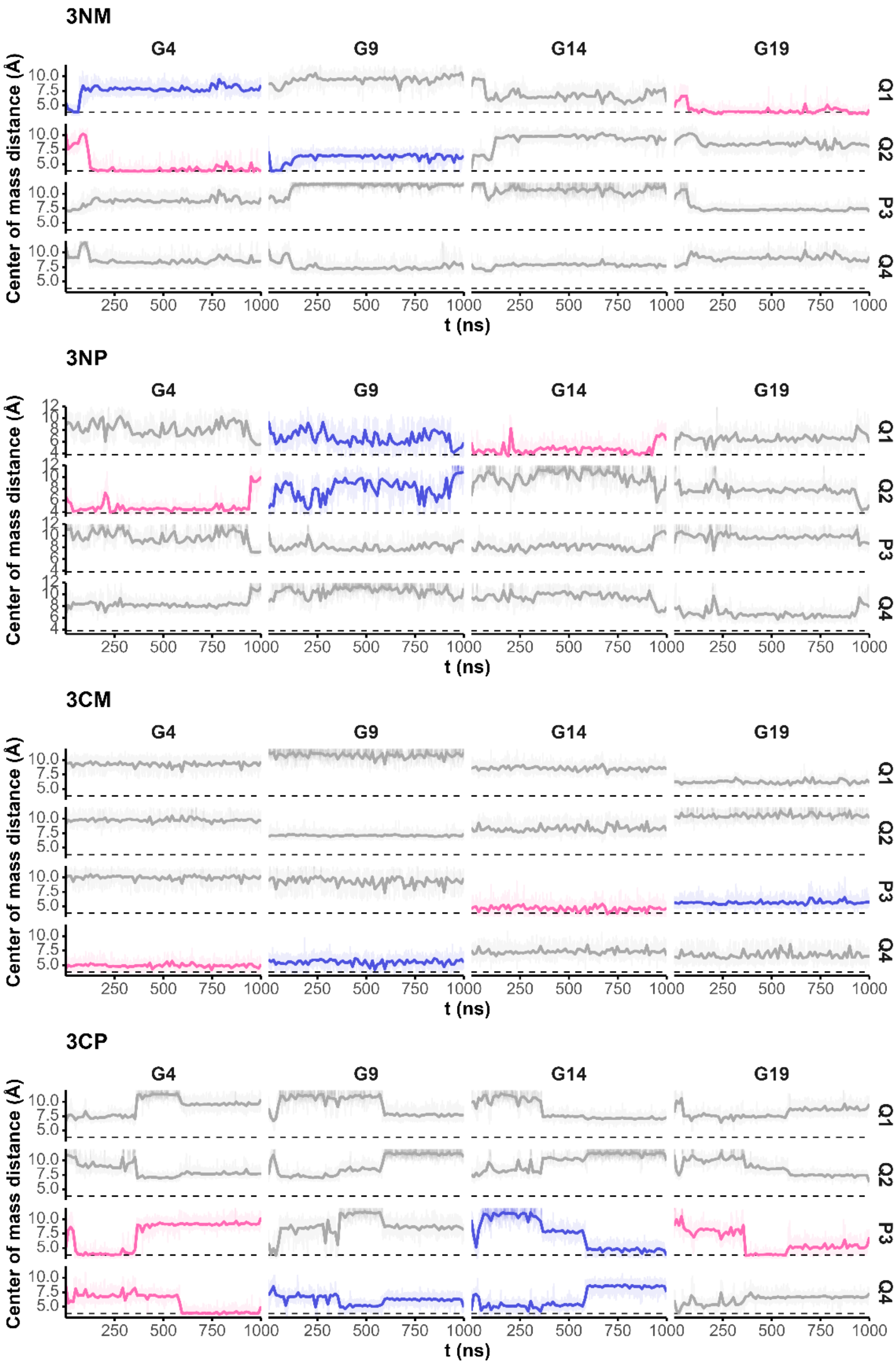

Figure S161. Distances between the aromatic cycles of QQPQ and the 3' tetrad guanines, for the 1:1 complexes. Aromatic cycles are numbered from 1 to 4 from the N- to C-termini. The data is colored by frequency of stacking (pink: often stacked, purple: occasionally stacked, grey: generally not stacked), estimated with the distance between G18 and G19 (dashed line) used as reference of 'proper' π-stacking

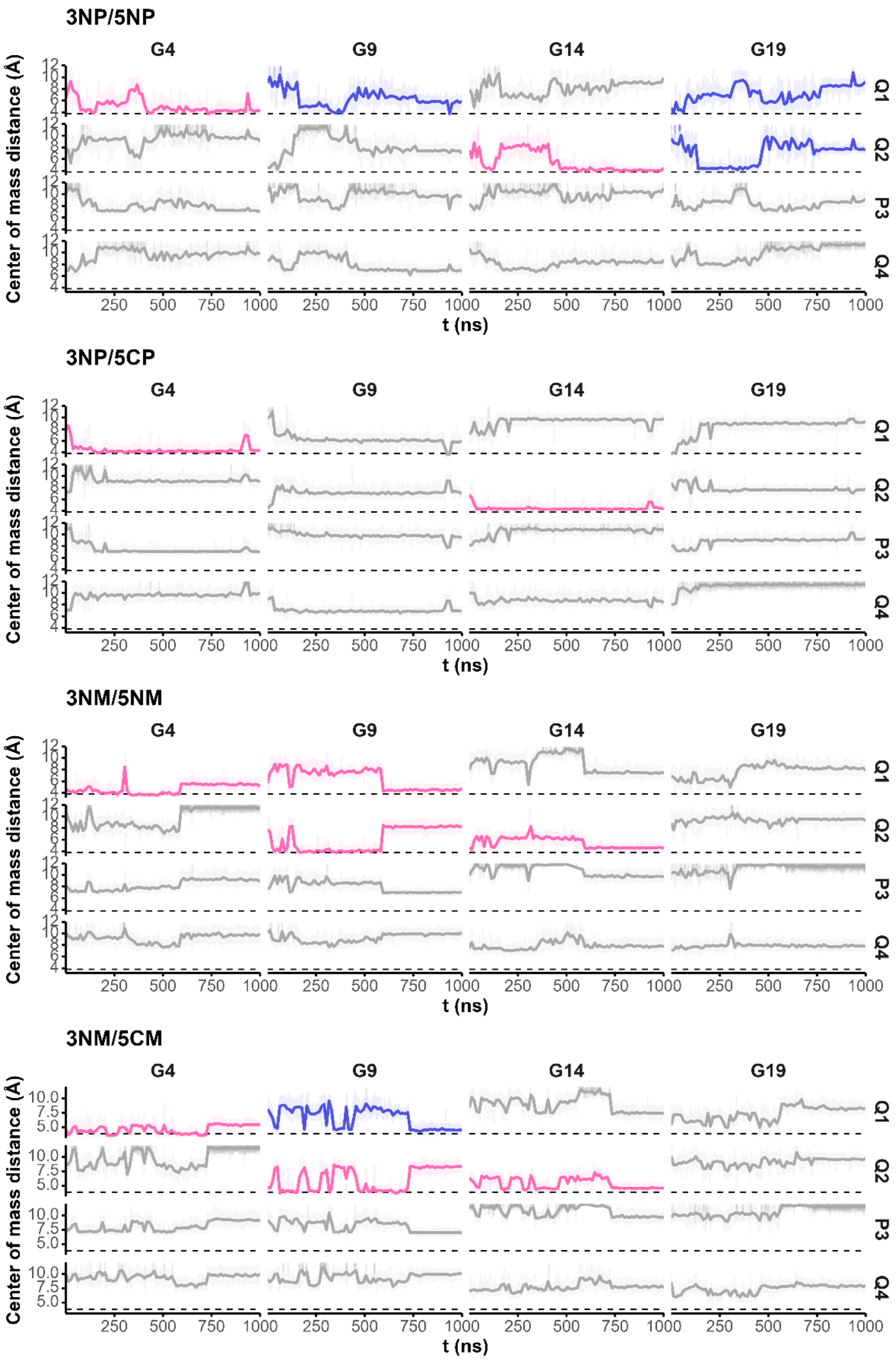

Figure S162. Distances between the aromatic cycles of QQPQ and the 3' tetrad guanines, for the 2:1 complexes. Aromatic cycles are numbered from 1 to 4 from the N- to C-termini. The data is colored by frequency of stacking (pink: often stacked, purple: occasionally stacked, grey: generally not stacked), estimated with the distance between G18 and G19 (dashed line) used as reference of 'proper' π-stacking

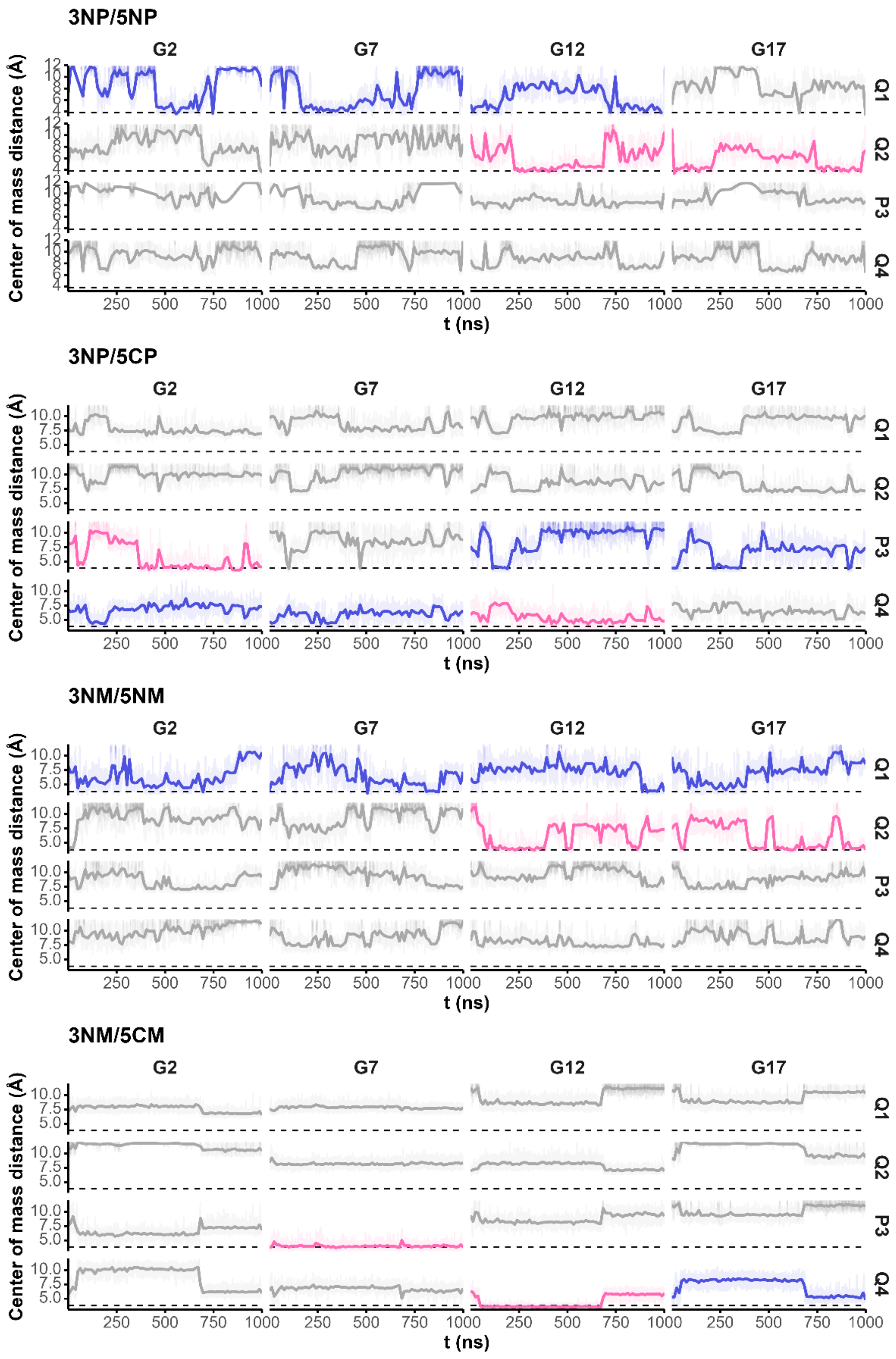

Figure S163. Distances between the aromatic cycles of QQPQ and the 5' tetrad guanines, for the 2:1 complexes. Aromatic cycles are numbered from 1 to 4 from the N- to C-termini. The data is colored by frequency of stacking (pink: often stacked, purple: occasionally stacked, grey: generally not stacked), estimated with the distance between G18 and G19 (dashed line) used as reference of 'proper' π-stacking

QQPQ rotation

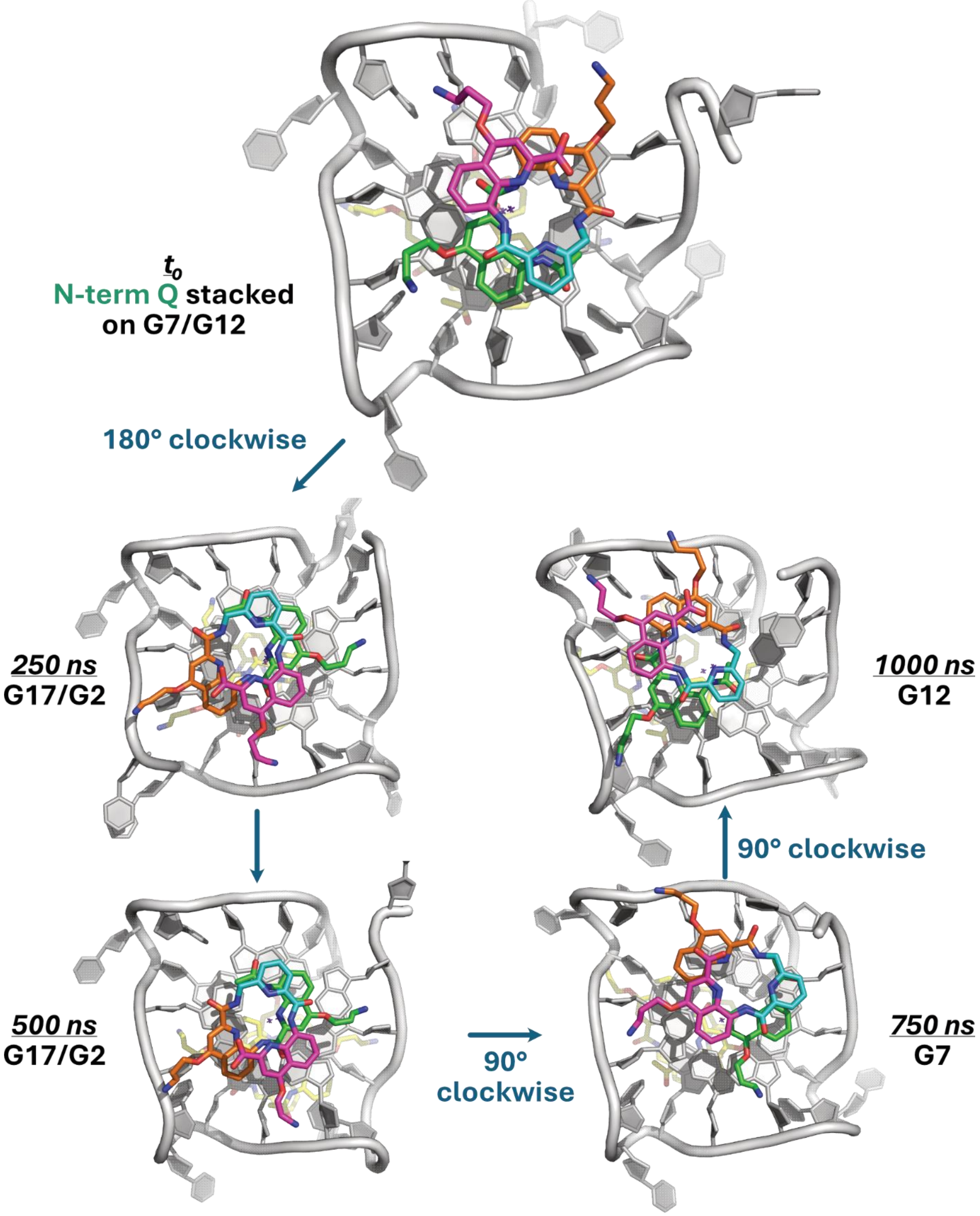

Figure S164. Example of a complete clockwise rotation of QQPQ (M enantiomer), here stacked by its N-terminal quinoline (green) on the 5'-end of 222T (complex 3NM/5NM). Note the very significant dynamics of 5' and 3' dTs during the simulation. Corresponding cycle-cycle distances are given in Figure S163 (see the sequential stacking for 3NM/5NM: Q1).

QQPQ stacking

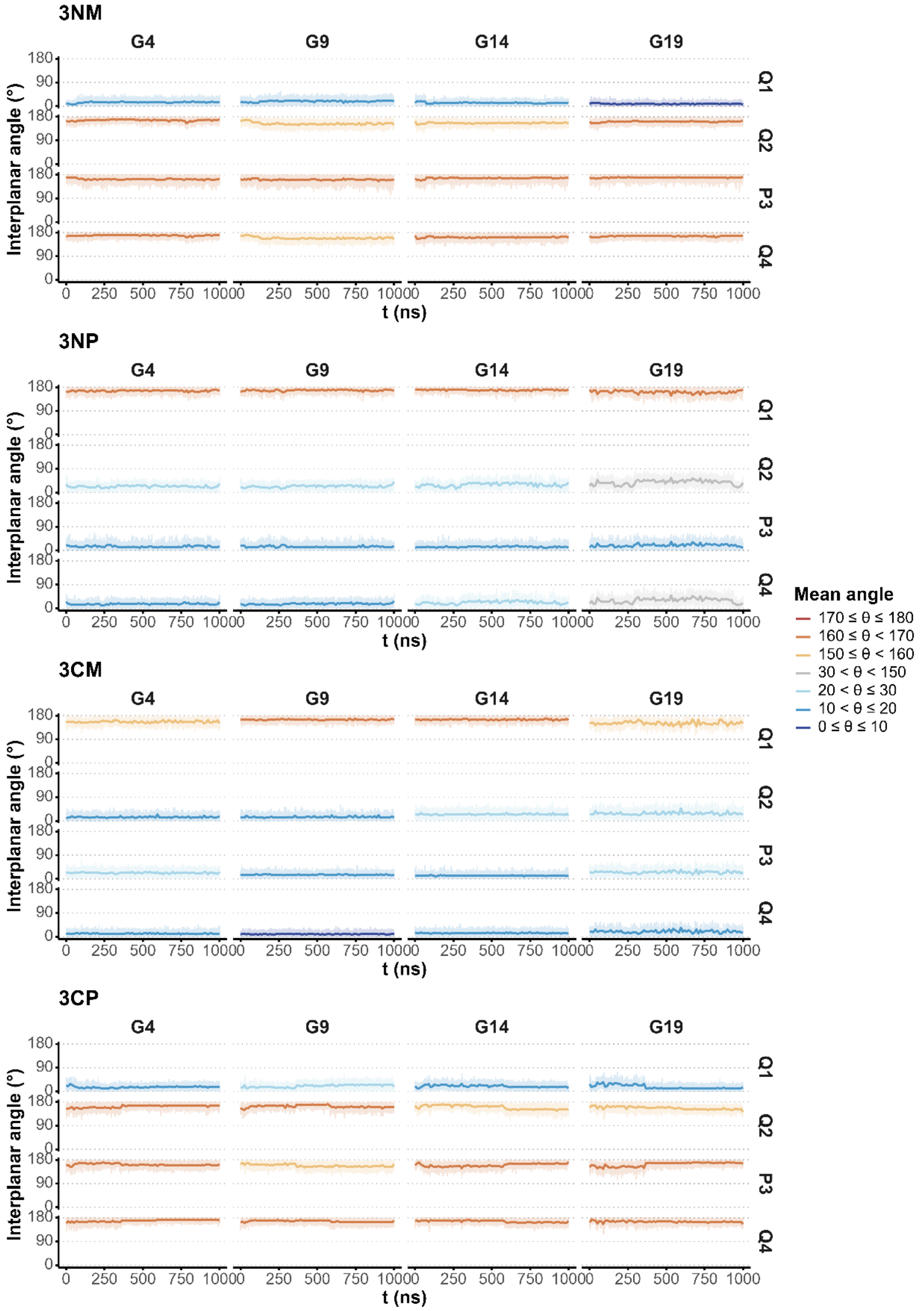

Figure S165. Angles between the planes defined by the aromatic cycles of QQPQ and the 3' tetrad guanines, for the 1:1 complexes. Aromatic cycles are numbered from 1 to 4 from the N- to C-termini. The data is colored by mean angle across the simulation: blue and orange colors denote similar and opposite orientations of the normal vectors, respectively, both being good stacking to perfect stacking (light to dark colors). Grey denotes an average twist greater than 30 degrees that is not compatible with a correct stacking.

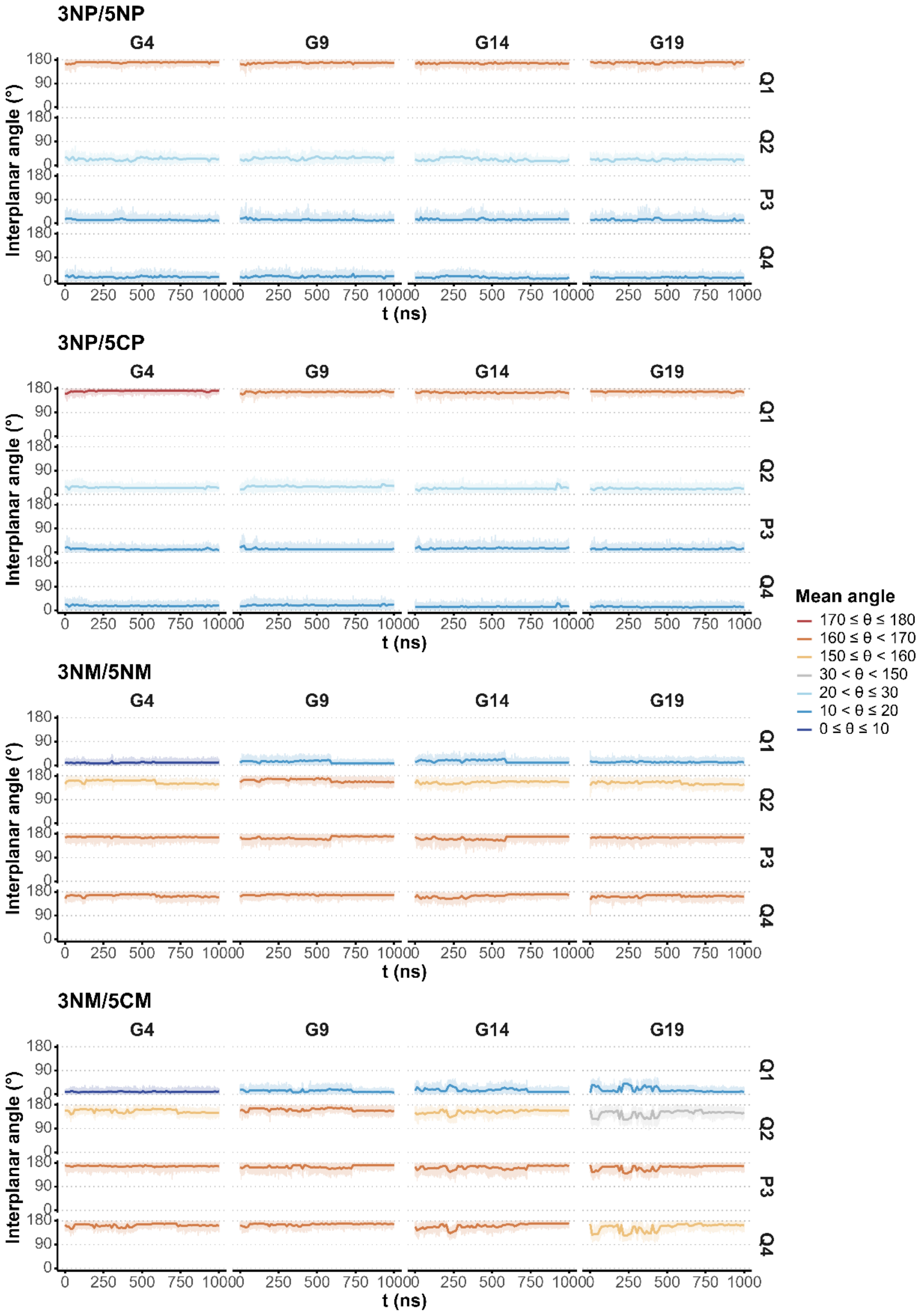

Figure S166. Angles between the planes defined by the aromatic cycles of QQPQ and the 3' tetrad guanines, for the 2:1 complexes. Aromatic cycles are numbered from 1 to 4 from the N- to C-termini. The data is colored by mean angle across the simulation: blue and orange colors denote similar and opposite orientations of the normal vectors, respectively, both being good stacking to perfect stacking (light to dark colors). Grey denotes an average twist greater than 30 degrees that is not compatible with a correct stacking.

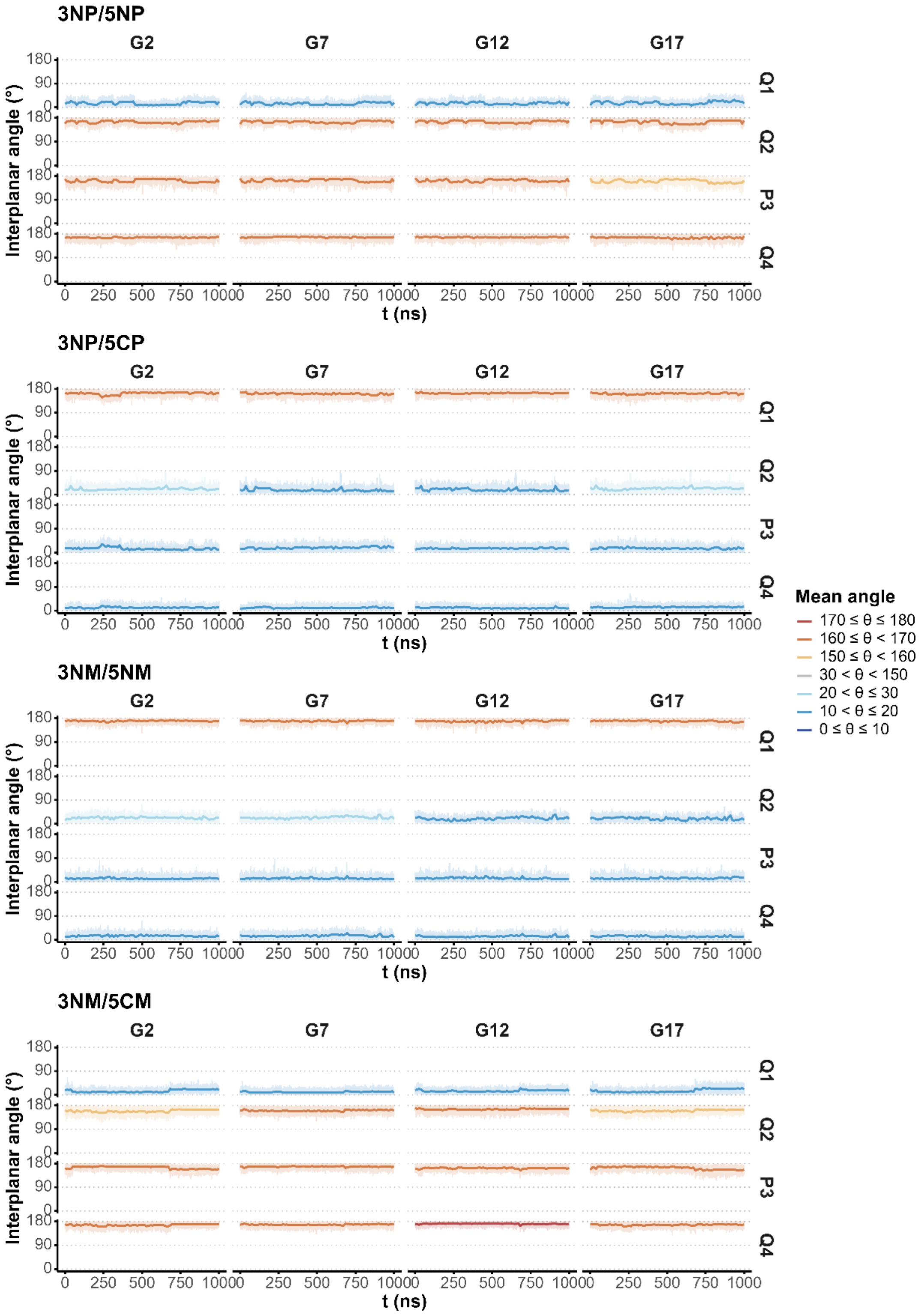

Figure S167. Angles between the planes defined by the aromatic cycles of QQPQ and the 5' tetrad guanines, for the 2:1 complexes. Aromatic cycles are numbered from 1 to 4 from the N- to C-termini. The data is colored by mean angle across the simulation: blue and orange colors denote similar and opposite orientations of the normal vectors, respectively, both being good stacking to perfect stacking (light to dark colors). Grey denotes an average twist greater than 30 degrees that is not compatible with a correct stacking.

Thymine binding

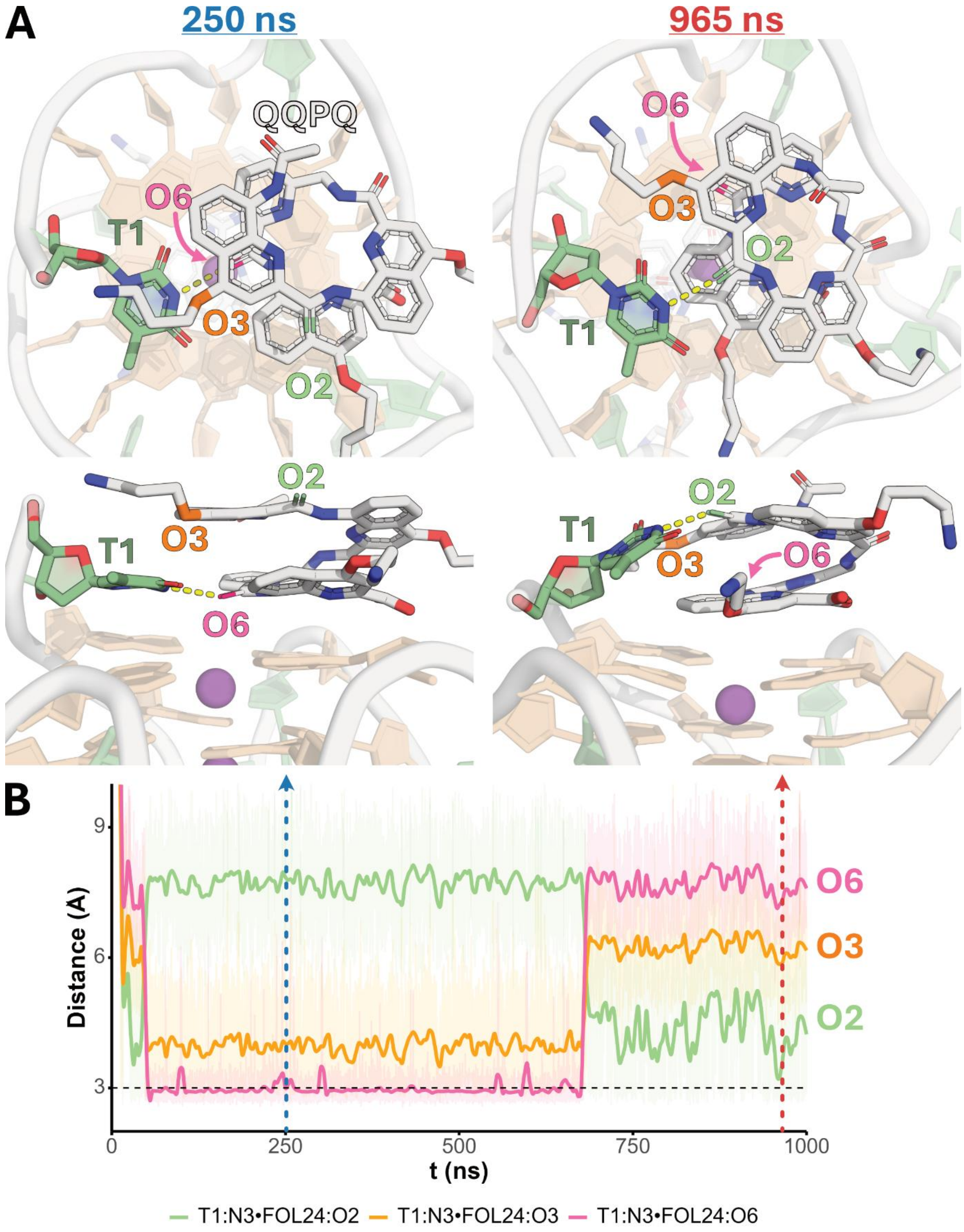

Figure S168. Example of discrete binding mode of QQPQ involving binding to a thymine. In 3NM5CM, QQPQ binds on the 5'-end by establishing an H-bond between its O6 (pink) and the N3 of dT1 (Panel A, left, 250 ns). This interaction has a significant lifetime, being maintained here for more than half a microsecond (Panel B). This leads to an inhibition of the rotation of QQPQ, as visible in Figure S163 (see 3NM/5CM: Q4/G12). Disruption of this specific interaction re-establishes the rotational freedom of QQPQ, yielding different binding arrangements (Panel A, right, 965 ns).

QQPQ/5YEY

RMSD

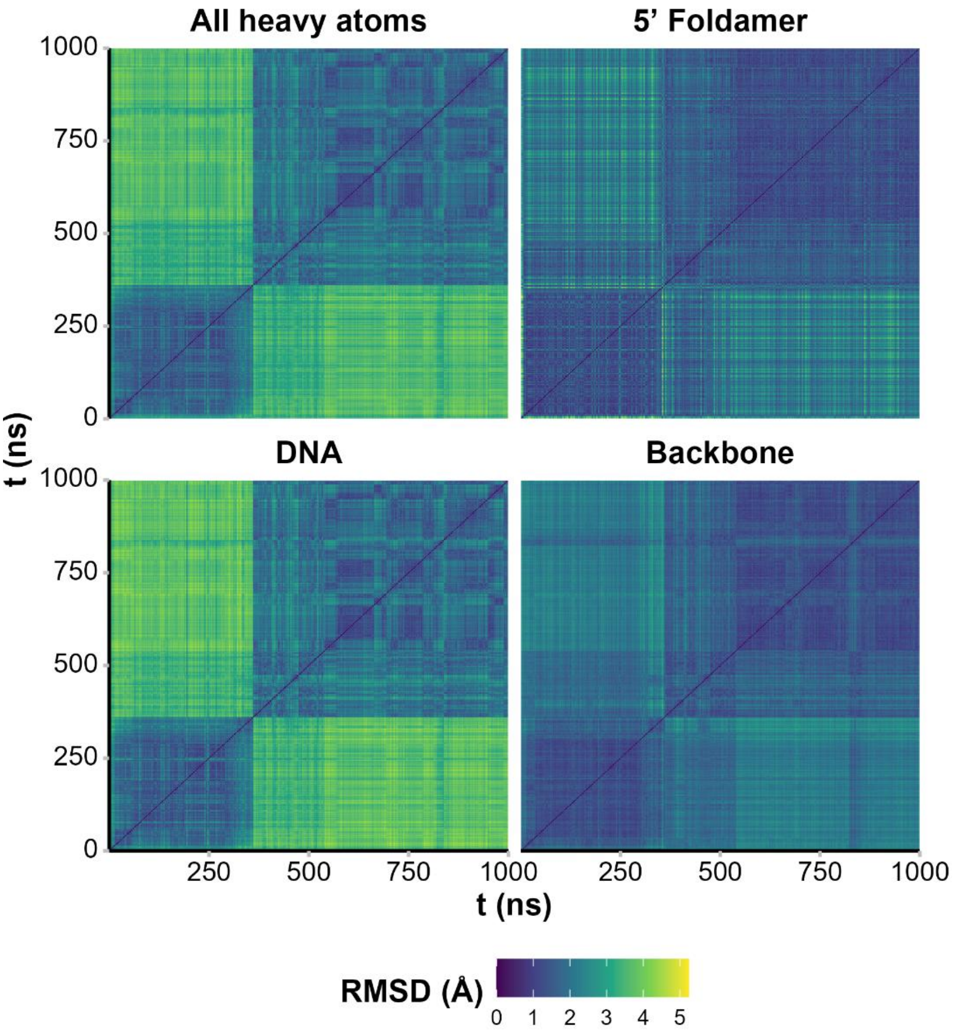

Figure S169. Pairwise RMSD of the 1:1 QQPQ:5YEY complexes calculated on all heavy atoms, and only on either foldamer, the DNA, and the DNA backbone. Lighter colors points to structural changes, while similar-colored squares along the diagonal indicate a relative structural stability during the corresponding time range.

QQPQ/5YEY T18 interactions

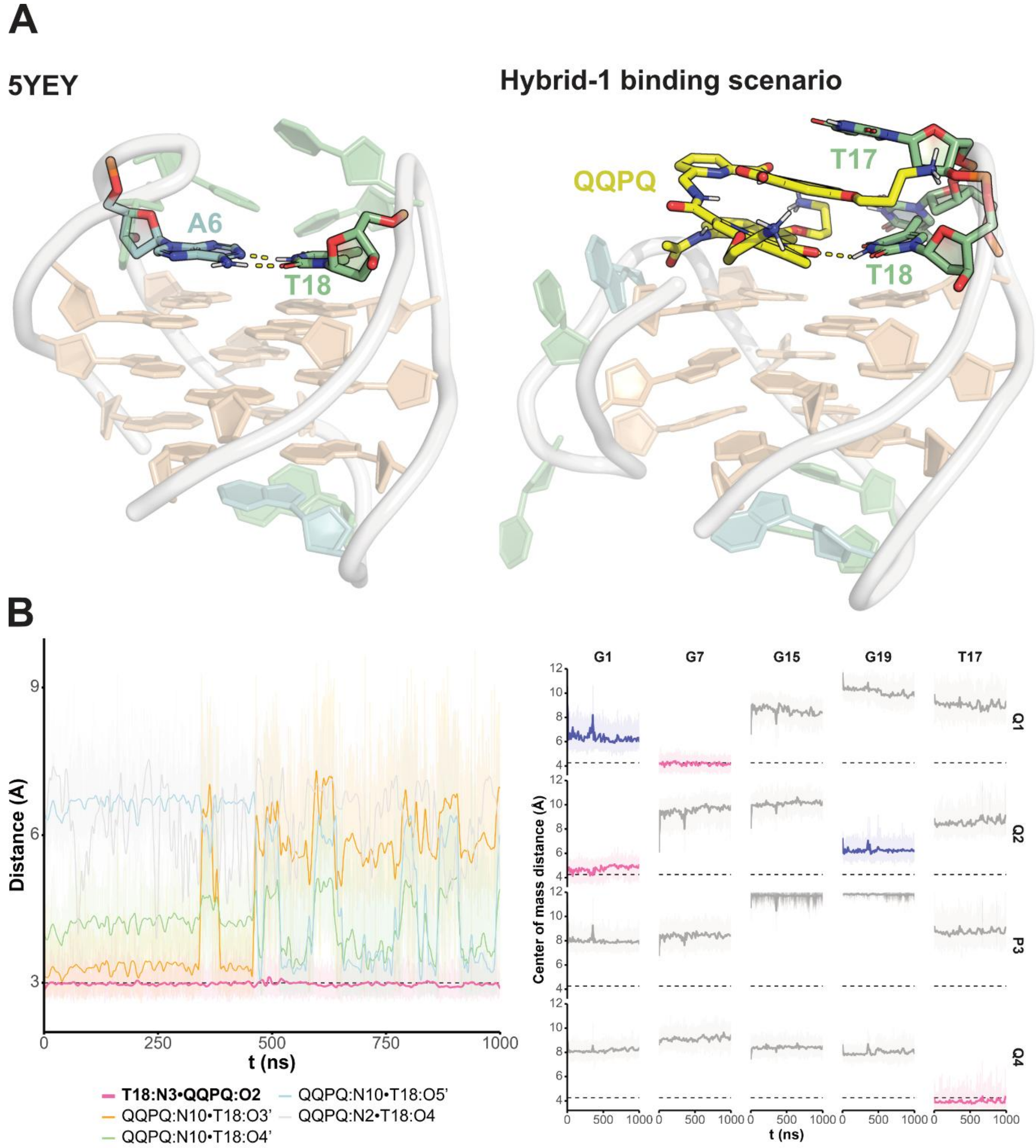

Figure S170. Exploring the binding of QQPQ to 5YEY converted to a hybrid-1 type loop arrangement. A. The deposited structure of 5YEY (left),[51] and a random frame extracted from the MD simulation of its hybrid-1 topology complexed by QQPQ. In this scenario, the A6:T18 base pair is disrupted giving space above the 5' tetrad for QQPQ to stack. Further, T18 is available to bind the amide oxygen at the N-terminus of QQPQ, reminiscent of the binding mode depicted in Figure S168A. T17 stacks on top of the C-terminus of QQPQ. B. Distances between H-bond donor and acceptor groups of QQPQ and T18, showing the stability of the pairing between QQPQ and the N3 of T18. Only bonds formed (i.e. distance < 3.1 Å) more than 5% of the time are shown. C. Distances between the aromatic cycles of QQPQ and the 5' tetrad guanines and T17 of 5YEY. Aromatic cycles are numbered from 1 to 4 from the N- to C-termini. The data is colored by frequency of stacking (pink: often stacked, purple: occasionally stacked, grey: generally not stacked), estimated with the distance between G20 and G21 (dashed line) used as reference of 'proper' π-stacking. The ligand is clearly locked in place, stacking with its first quinoline to G7, and being capped by T17, which stacks on Q4.

References

(1) Vallade, M.; Sai Reddy, P.; Fischer, L.; Huc, I. Enhancing Aromatic Foldamer Helix Dynamics to Probe Interactions with Protein Surfaces. *Eur. J. Org. Chem.* **2018**, *2018* (40), 5489–5498. https://doi.org/10.1002/ejoc.201800855.

(2) Lenarčič Živković, M.; Rozman, J.; Plavec, J. Adenine-Driven Structural Switch from a Two- to Three-Quartet DNA G-Quadruplex. *Angew. Chem. Int. Ed.* **2018**, *57* (47), 15395–15399. https://doi.org/10.1002/anie.201809328.

(3) Ghosh, A.; Largy, E.; Gabelica, V. DNA G-Quadruplexes for Native Mass Spectrometry in Potassium: A Database of Validated Structures in Electrospray-Compatible Conditions. *Nucleic Acids Res.* **2021**, *49* (4), 2333–2345. https://doi.org/10.1093/nar/gkab039.

(4) Mergny, J.-L. Kinetics of Tetramolecular Quadruplexes. *Nucleic Acids Res.* **2005**, *33* (1), 81–94. https://doi.org/10.1093/nar/gki148.

(5) Marchand, A.; Livet, S.; Rosu, F.; Gabelica, V. Drift Tube Ion Mobility: How to Reconstruct Collision Cross Section Distributions from Arrival Time Distributions? *Anal. Chem.* **2017**, *89* (23), 12674–12681. https://doi.org/10.1021/acs.analchem.7b01736.

(6) Gabelica, V.; Rosu, F.; Pauw, E. D. A Simple Method to Determine Electrospray Response Factors of Noncovalent Complexes. *Anal. Chem.* **2009**, *81* (16), 6708–6715. https://doi.org/10.1021/ac900785m.

(7) Rosu, F.; De Pauw, E.; Gabelica, V. Electrospray Mass Spectrometry to Study Drug-Nucleic Acids Interactions. *Biochimie* **2008**, *90* (7), 1074–1087. https://doi.org/10.1016/j.biochi.2008.01.005.

(8) Akashi, M.; Shaw, G.; Hachiya, M.; Elstner, E.; Suzuki, G.; Koeffler, P. Number and Location of AUUUA Motifs: Role in Regulating Transiently Expressed RNAs. *Blood* **1994**, *83* (11), 3182–3187.

(9) Munoz-Tello, P.; Rajappa, L.; Coquille, S.; Thore, S. Polyuridylation in Eukaryotes: A 3'-End Modification Regulating RNA Life. *BioMed Res. Int.* **2015**, *2015*, 968127. https://doi.org/10.1155/2015/968127.

(10) Neese, F. Software Update: The ORCA Program System—Version 5.0. *WIREs Comput. Mol. Sci.* **2022**, *12* (5), e1606. https://doi.org/10.1002/wcms.1606.

(11) Neese, F. An Improvement of the Resolution of the Identity Approximation for the Formation of the Coulomb Matrix. *J. Comput. Chem.* **2003**, *24* (14), 1740–1747. https://doi.org/10.1002/jcc.10318.

(12) Neese, F.; Wennmohs, F.; Hansen, A.; Becker, U. Efficient, Approximate and Parallel Hartree–Fock and Hybrid DFT Calculations. A 'Chain-of-Spheres' Algorithm for the Hartree–Fock Exchange. *Chem. Phys.* **2009**, *356* (1–3), 98–109. https://doi.org/10.1016/j.chemphys.2008.10.036.

(13) Helmich-Paris, B.; De Souza, B.; Neese, F.; Izsák, R. An Improved Chain of Spheres for Exchange Algorithm. *J. Chem. Phys.* **2021**, *155* (10), 104109. https://doi.org/10.1063/5.0058766.

(14) Neese, F. The SHARK Integral Generation and Digestion System. *J. Comput. Chem.* **2023**, *44* (3), 381–396. https://doi.org/10.1002/jcc.26942.

(15) Izsák, R.; Neese, F. An Overlap Fitted Chain of Spheres Exchange Method. *J. Chem. Phys.* **2011**, *135* (14), 144105. https://doi.org/10.1063/1.3646921.

(16) Izsák, R.; Hansen, A.; Neese, F. The Resolution of Identity and Chain of Spheres Approximations for the LPNO-CCSD Singles Fock Term. *Mol. Phys.* **2012**, *110* (19–20), 2413–2417. https://doi.org/10.1080/00268976.2012.687466.

(17) Neese, F. The ORCA Program System. *WIREs Comput. Mol. Sci.* **2012**, *2* (1), 73–78. https://doi.org/10.1002/wcms.81.

(18) Izsák, R.; Neese, F.; Klopper, W. Robust Fitting Techniques in the Chain of Spheres Approximation to the Fock Exchange: The Role of the Complementary Space. *J. Chem. Phys.* **2013**, *139* (9), 094111. https://doi.org/10.1063/1.4819264.

(19) Neese, F. Software Update: The ORCA Program System, Version 4.0. *WIREs Comput. Mol. Sci.* **2018**, *8* (1), e1327. https://doi.org/10.1002/wcms.1327.

(20) Neese, F.; Wennmohs, F.; Becker, U.; Riplinger, C. The ORCA Quantum Chemistry Program Package. *J. Chem. Phys.* **2020**, *152* (22), 224108. https://doi.org/10.1063/5.0004608.

(21) Neese, F. Approximate Second-Order SCF Convergence for Spin Unrestricted Wavefunctions. *Chem. Phys. Lett.* **2000**, *325* (1–3), 93–98. https://doi.org/10.1016/S0009-2614(00)00662-X.

(22) Lehtola, S.; Steigemann, C.; Oliveira, M. J. T.; Marques, M. A. L. Recent Developments in Libxc — A Comprehensive Library of Functionals for Density Functional Theory. *SoftwareX* **2018**, *7*, 1–5. https://doi.org/10.1016/j.softx.2017.11.002.

(23) E. F. Valeev. Libint: A Library for the Evaluation of Molecular Integrals of Many-Body Operators over Gaussian Functions, 2025. http://libint.valeyev.net/.

(24) Hehre, W. J.; Ditchfield, R.; Pople, J. A. Self—Consistent Molecular Orbital Methods. XII. Further Extensions of Gaussian—Type Basis Sets for Use in Molecular Orbital Studies of Organic Molecules. *J. Chem. Phys.* **1972**, *56* (5), 2257–2261. https://doi.org/10.1063/1.1677527.

(25) Francl, M. M.; Pietro, W. J.; Hehre, W. J.; Binkley, J. S.; Gordon, M. S.; DeFrees, D. J.; Pople, J. A. Self-Consistent Molecular Orbital Methods. XXIII. A Polarization-Type Basis Set for Second-Row Elements. *J. Chem. Phys.* **1982**, *77* (7), 3654–3665. https://doi.org/10.1063/1.444267.

(26) Rassolov, V. A.; Pople, J. A.; Ratner, M. A.; Windus, T. L. 6-31G* Basis Set for Atoms K through Zn. *J. Chem. Phys.* **1998**, *109* (4), 1223–1229. https://doi.org/10.1063/1.476673.

(27) Weigend, F. Accurate Coulomb-Fitting Basis Sets for H to Rn. *Phys. Chem. Chem. Phys.* **2006**, *8* (9), 1057. https://doi.org/10.1039/b515623h.

(28) Marenich, A. V.; Cramer, C. J.; Truhlar, D. G. Universal Solvation Model Based on Solute Electron Density and on a Continuum Model of the Solvent Defined by the Bulk Dielectric Constant and Atomic Surface Tensions. *J. Phys. Chem. B* **2009**, *113* (18), 6378–6396. https://doi.org/10.1021/jp810292n.

(29) Bannwarth, C.; Caldeweyher, E.; Ehlert, S.; Hansen, A.; Pracht, P.; Seibert, J.; Spicher, S.; Grimme, S. Extended TIGHT-BINDING Quantum Chemistry Methods. *WIREs Comput. Mol. Sci.* **2021**, *11* (2), e1493. https://doi.org/10.1002/wcms.1493.

(30) Bannwarth, C.; Ehlert, S.; Grimme, S. GFN2-xTB—An Accurate and Broadly Parametrized Self-Consistent Tight-Binding Quantum Chemical Method with Multipole Electrostatics and Density-Dependent Dispersion Contributions. *J. Chem. Theory Comput.* **2019**, *15* (3), 1652–1671. https://doi.org/10.1021/acs.jctc.8b01176.

(31) Spicher, S.; Grimme, S. Robust Atomistic Modeling of Materials, Organometallic, and Biochemical Systems. *Angew. Chem. Int. Ed.* **2020**, *59* (36), 15665–15673. https://doi.org/10.1002/anie.202004239.

(32) Ehlert, S.; Stahn, M.; Spicher, S.; Grimme, S. Robust and Efficient Implicit Solvation Model for Fast Semiempirical Methods. *J. Chem. Theory Comput.* **2021**, *17* (7), 4250–4261. https://doi.org/10.1021/acs.jctc.1c00471.

(33) Lu, T.; Chen, F. Multiwfn: A Multifunctional Wavefunction Analyzer. *J. Comput. Chem.* **2012**, *33* (5), 580–592. https://doi.org/10.1002/jcc.22885.

(34) Zhang, J.; Lu, T. Efficient Evaluation of Electrostatic Potential with Computerized Optimized Code. *Phys. Chem. Chem. Phys.* **2021**, *23* (36), 20323–20328. https://doi.org/10.1039/D1CP02805G.

(35) Wang, J.; Wolf, R. M.; Caldwell, J. W.; Kollman, P. A.; Case, D. A. Development and Testing of a General Amber Force Field. *J. Comput. Chem.* **2004**, *25* (9), 1157–1174. https://doi.org/10.1002/jcc.20035.

(36) Wang, J.; Wang, W.; Kollman, P. A.; Case, D. A. Automatic Atom Type and Bond Type Perception in Molecular Mechanical Calculations. *J. Mol. Graph. Model.* **2006**, *25* (2), 247–260. https://doi.org/10.1016/j.jmgm.2005.12.005.

(37) Case, D. A.; Aktulga, H. M.; Belfon, K.; Cerutti, D. S.; Cisneros, G. A.; Cruzeiro, V. W. D.; Forouzesh, N.; Giese, T. J.; Götz, A. W.; Gohlke, H.; Izadi, S.; Kasavajhala, K.; Kaymak, M. C.; King, E.; Kurtzman, T.; Lee, T.-S.; Li, P.; Liu, J.; Luchko, T.; Luo, R.; Manathunga, M.; Machado, M. R.; Nguyen, H. M.; O'Hearn, K. A.; Onufriev, A. V.; Pan, F.; Pantano, S.; Qi, R.; Rahnamoun, A.; Risheh, A.; Schott-Verdugo, S.; Shajan, A.; Swails, J.; Wang, J.; Wei, H.; Wu, X.; Wu, Y.; Zhang, S.; Zhao, S.; Zhu, Q.; Cheatham, T. E.; Roe, D. R.; Roitberg, A.; Simmerling, C.; York, D. M.; Nagan, M. C.; Merz, K. M. AmberTools. *J. Chem. Inf. Model.* **2023**, *63* (20), 6183–6191. https://doi.org/10.1021/acs.jcim.3c01153.

(38) Zgarbová, M.; Šponer, J.; Jurečka, P. Z-DNA as a Touchstone for Additive Empirical Force Fields and a Refinement of the Alpha/Gamma DNA Torsions for AMBER. *J. Chem. Theory Comput.* **2021**, *17* (10), 6292–6301. https://doi.org/10.1021/acs.jctc.1c00697.

(39) Love, O.; Galindo-Murillo, R.; Zgarbová, M.; Šponer, J.; Jurečka, P.; Cheatham, T. E. Assessing the Current State of Amber Force Field Modifications for DNA–2023 Edition. *J. Chem. Theory Comput.* **2023**, *19* (13), 4299–4307. https://doi.org/10.1021/acs.jctc.3c00233.

(40) Izadi, S.; Anandakrishnan, R.; Onufriev, A. V. Building Water Models: A Different Approach. *J. Phys. Chem. Lett.* **2014**, *5* (21), 3863–3871. https://doi.org/10.1021/jz501780a.

(41) Li, Z.; Song, L. F.; Li, P.; Merz, K. M. Systematic Parametrization of Divalent Metal Ions for the OPC3, OPC, TIP3P-FB, and TIP4P-FB Water Models. *J. Chem. Theory Comput.* **2020**, *16* (7), 4429–4442. https://doi.org/10.1021/acs.jctc.0c00194.

(42) Machado, M. R.; Pantano, S. Split the Charge Difference in Two! A Rule of Thumb for Adding Proper Amounts of Ions in MD Simulations. *J. Chem. Theory Comput.* **2020**, *16* (3), 1367–1372. https://doi.org/10.1021/acs.jctc.9b00953.

(43) Götz, A. W.; Williamson, M. J.; Xu, D.; Poole, D.; Le Grand, S.; Walker, R. C. Routine Microsecond Molecular Dynamics Simulations with AMBER on GPUs. 1. Generalized Born. *J. Chem. Theory Comput.* **2012**, *8* (5), 1542–1555. https://doi.org/10.1021/ct200909j.

(44) Salomon-Ferrer, R.; Götz, A. W.; Poole, D.; Le Grand, S.; Walker, R. C. Routine Microsecond Molecular Dynamics Simulations with AMBER on GPUs. 2. Explicit Solvent Particle Mesh Ewald. *J. Chem. Theory Comput.* **2013**, *9* (9), 3878–3888. https://doi.org/10.1021/ct400314y.

(45) Le Grand, S.; Götz, A. W.; Walker, R. C. SPFP: Speed without Compromise—A Mixed Precision Model for GPU Accelerated Molecular Dynamics Simulations. *Comput. Phys. Commun.* **2013**, *184* (2), 374–380. https://doi.org/10.1016/j.cpc.2012.09.022.

(46) Sindhikara, D. J.; Kim, S.; Voter, A. F.; Roitberg, A. E. Bad Seeds Sprout Perilous Dynamics: Stochastic Thermostat Induced Trajectory Synchronization in Biomolecules. *J. Chem. Theory Comput.* **2009**, *5* (6), 1624–1631. https://doi.org/10.1021/ct800573m.

(47) Berendsen, H. J. C.; Postma, J. P. M.; Van Gunsteren, W. F.; DiNola, A.; Haak, J. R. Molecular Dynamics with Coupling to an External Bath. *J. Chem. Phys.* **1984**, *81* (8), 3684–3690. https://doi.org/10.1063/1.448118.

(48) Grant, B. J.; Rodrigues, A. P. C.; ElSawy, K. M.; McCammon, J. A.; Caves, L. S. D. Bio3d: An R Package for the Comparative Analysis of Protein Structures. *Bioinformatics* **2006**, *22* (21), 2695–2696. https://doi.org/10.1093/bioinformatics/btl461.

(49) Bengtsson, H. A Unifying Framework for Parallel and Distributed Processing in R Using Futures. *R J.* **2021**, *13* (2), 208. https://doi.org/10.32614/RJ-2021-048.

(50) Charrad, M.; Ghazzali, N.; Boiteau, V.; Niknafs, A. NbClust: An R Package for Determining the Relevant Number of Clusters in a Data Set. *J. Stat. Softw.* **2014**, *61* (6), 1–36.

(51) Liu, C.; Zhou, B.; Geng, Y.; Yan Tam, D.; Feng, R.; Miao, H.; Xu, N.; Shi, X.; You, Y.; Hong, Y.; Tang, B. Z.; Kwan Lo, P.; Kuryavyi, V.; Zhu, G. A Chair-Type G-Quadruplex Structure Formed by a Human Telomeric Variant DNA in K^+solution. *Chem. Sci.* **2019**, *10* (1), 218–226. https://doi.org/10.1039/c8sc03813a.
